# Analysis of the First Spectrum of Ruthenium (Ru I)

**DOI:** 10.6028/jres.063A.017

**Published:** 1959-12-01

**Authors:** K. G. Kessler

## Abstract

The analysis of the first spectrum of ruthenium has been extended with the aid of digital computers. A total of 105 even and 206 odd levels are listed with observed Landé *g*-factors for 54 even and 148 odd levels. A complete list of approximately 3,400 classified lines in the range 2013 to 11484 A is presented. The ionization limit calculated from a two member series is 59410 cm^−1^ or 7.364 v.

## 1. Introduction

Constant differences between wave numbers of line pairs in the arc spectrum of ruthenium (Ru I) were found by Kayser [1]^1^ in 1897. Paulson [2], in 1915, reported 17 recurring differences among the reciprocals of the ruthenium wavelengths in air. The first multiplets and energy levels were found by Meggers and Laporte [3, 4] who investigated the absorption spectrum to identify the ground-state transitions and assigned quantum numbers to 16 even and 24 odd levels. Simultaneously, Sommer [5, 6] published 44 even and 71 odd levels and identified spectral terms with the aid of Zeeman-effect data.

The next contribution to the analysis was by Harrison and McNally [7] who observed Zeeman patterns for 450 lines and published *g*-values for 38 even and 102 odd levels. With the aid of an interval sorter, McNally later expanded this analysis [8] to include 61 even and 188 odd levels, accounting for 2,380 classified lines. This latter work has never been published, and was not made available to us until the present investigation was well under way.

## 2. Experimental Procedure

Line lists containing 4,492 ruthenium arc and 1,527 ruthenium spark lines compiled from spectrograms made at NBS [9] were used in this investigation. The observed wavelengths were converted to vacuum wave numbers by digital computers (the IBM 604, SEAC, and IBM 704 at various times) coded to compute the index of refraction of air by means of Edlen’s formula [10]. An IBM 604 computer was then utilized to calculate all permitted transitions predicted by the known energy levels. These permitted transitions were compared with the observed lines in order to check the validity of these energy levels. All lines thus classified were removed from the line list to reduce this list to one containing only unclassified lines. Predictions which fell within ±0.3 cm^−1^ were accepted as coincidences by the computer. Those which fell between ±0.2 and ±0.3 cm^−1^ were examined by the author and considered to be valid if adjacent lines in the multiplet were observed. The remaining list of unclassified Ru I lines was then searched for significant differences.

The search for new energy levels was carried out with the aid of electronic digital computers. The major portion of this work was done prior to 1957 with the NBS computer, the SEAC. The technique used is described elsewhere [11]. More recently, the faster IBM 704 computer has become available to us, and a revised and greatly expanded version of the technique used on the SEAC was developed for the newer computer by Coleman and Bozman [12]. The fundamental difference between the two computer techniques is that the earlier search was accomplished with a selected list of level differences, whereas the greater speed of the later machine permitted the use of all possible differences in the search procedure.

The list of unclassified lines remaining after the SEAC search has recently been investigated by means of the 704 routine. This search has yielded only five new even levels and one new odd level. It appears unlikely that more levels will be found with the present line list. More Zeeman data, particularly in the red and infrared regions, are needed for further progress on the classification of lines in this spectrum. New spectrograms devoid of underlying band spectra would also be helpful.

The energy levels, *g*-values, and percentage compositions for the low even levels calculated by Trees [13] have been very helpful in this analysis. For details of this, his paper in this issue (p. 255) should be consulted. For the rest of the assignments the author is indebted to Russell and Moore for the guidelines laid down in their analysis of the homologous spectrum, Fe I [14], and to C. E. Moore for her invaluable aid in grouping levels into terms and in assigning configurations.

The energy levels listed in table 1 have been adjusted to fit the new line list, but differ only slightly from those reported by McNally [8] in the case of those lines that occur in both lists. The estimated errors in the value of the levels range from ±0.01 cm^−1^ for the low oven levels to ±0.05 cm^−1^ for those high terms that are determined by only a few transitions.

The array of observed terms is presented as table 2. To facilitate comparison, this array is given in the same form as the array of predicted terms published in A.E.L. [15].

The complete list of classified lines is given in table 3. The intensity of the observed line on an arbitrary scale of 1 to 10,000, is listed in column 1. The wavelength in column 2 and the corresponding wave number is listed in column 3. The classification of the observed transition in terms of the energy levels listed in table 1 is given in column 4. A partial list of the 40 most intense remaining unclassified lines is given in table 4.

## 3. Discussion of Results

### 3.1. Even Terms

The known parent terms in Ru II have proven a useful guide in assigning the 4*d*^7^ 5*s* configurations in Ru I. Terms can be assigned on the basis of the intensity of the combinations with a fair degree of certainty for the limits *a* ^4^F, *a* ^4^P, *a* ^2^G, *a* ^2^P, *a* ^2^D, and *a* ^2^H in Ru II. A striking analogy exists with Fe I, although the term intervals are more irregular in Ru I. For these configurations, the levels are roughly 6000 to 12000 cm^−1^ lower than similar terms in Fe I.

All energy levels of Ru I which have been found are listed in table 1. Term designations have been assigned when possible. The notation is identical with that used by C. E. Moore in the Atomic Energy Level Volumes [15], henceforth referred to as A.E.L. The configuration is given in the first column, term designation in the second, *J*-value in the third, energy level in the fourth, the term-intervals in the fifth and the observed *g*-value in the sixth column. The five miscellaneous even levels are designated with letters B through F. Energy levels of odd parity are given in italics. Miscellaneous odd levels are designated by numbers 1° through 63°. The initials in the last column indicate the earliest reference for each level. The meaning of these initials is as follows: P, Paulson [2]; ML, Meggers and Laporte [3,4]; S, Sommer [5,6]; HM, Harrison and McNally [7]; Mc, McNally [8]; and K, this paper.

This table is identical with that published in A.E.L. [15] except for the following changes: The levels *d* ^3^F_2_, *a* ^1^F_3_, *a* ^1^I_6_, *e* ^5^G_4_, *e* ^3^P_2_, F_1_ and 63°_3_ have been added, minor typographical errors in the energy level of *y* ^3^H°_5_ and in the *g*-value for *b* ^3^H_6_ have been corrected and the level formerly designated as A_4_ has been designated *f* ^3^F_4_. The majority of the *g*-values are quoted from Harrison and McNally [7] and McNally [8]. The hitherto unpublished *g*-values from McNally’s thesis together with a few additions contributed by the present author are included in the following paper. More Zeeman observations are needed in the red and infrared regions, where the present spectrograms record only very few lines.

The writer has benefited from consultations with Trees who has furnished theoretical predicted values for all of the low even terms. These calculations are based on parameters derived from known levels and are an excellent guide to the analysis. The general agreement of observation with his predictions is good. His paper [13] should be consulted for further details.

The LS coupling designations represent in general the major composition of each level, but some of the levels are mixed to such an extent that the LS notation should be used with caution. In particular, the designations of the levels *a* ^3^D_2_ at 15054.07 cm^−1^ and *a* ^1^D_2_ at 17045.97 cm^−1^ should, according to Trees’ calculations be interchanged. The relative intensities of the observed combinations and the term intervals, however, favor the designations assigned in table 1.

The level at 25200.97 cm^−1^ has been designated as 4*d*^7^ (*a* ^2^F)5*s a* ^1^F_3_ despite the absence of any observed combinations with singlet levels. This assignment was made on the strength of Trees’ calculations which predict a level at 25210 cm^−1^ composed of 58 percent 4*d*^7^ (*a* ^2^F) 5*s* ^1^F_3_ and 16 percent 4*d*^6^5*s*^2 3^G_3_. The odd terms of similar parentage are unfortunately not well known.

The *a* ^5^D term having the configuration 4*d*^6^ 5*s*^2^ is about 8000 cm^−1^ higher than the corresponding terms in Fe I. The level *a* ^5^D_4_, corresponding to the ground state of Fe I, occurs at 7483 cm^−1^ in Ru I. The remaining terms of this configuration are only slightly higher (0 to 3,000 cm^−1^) than the corresponding terms in Fe I. Of the 16 terms predicted for this configuration, 8 have been identified but *d* ^3^P and *b* ^3^D are each missing one level and ƒ ^3^F is missing two levels. Of the 8 singlets predicted, only *a* ^1^I has been found. According to Trees’ predictions, the higher ^1^S and ^1^D terms will lie above the ionization limit. The other missing singlet terms are ^1^S predicted near 33411 cm^−1^, ^1^D near 33905 cm^−1^, ^1^F near 39308 cm^−1^ and two ^1^G terms, one near 31719, the other near 48417 cm^−1^. A high ^3^P term predicted near 43571 cm^−1^ is also missing.

The *b* ^3^D_3_ level deviates sufficiently, by 582 cm^−1^, from Trees’ value to east some doubt upon its validity. A level at 29617.15 cm^−1^ which might possibly be designated as 4*d*^6^ 5*s^2^ b* ^3^D_1_ has not been included in table 1. The agreement with the predicted position, 29621 cm^−1^, is good, but only three weak combinations with $w{\;}^{3}D_{2}^{{}^{\circ}}$, $z{\;}^{1}D_{2}^{{}^{\circ}}$, and $y{\;}^{1}P_{1}^{{}^{\circ}}$ have been observed and further confirmation is needed.

The *c* ^3^F_2_ and *c* ^3^F_3_ levels deviate by 149 and 173 cm^−1^ from their predicted positions and both are strongly mixed with 4*d*^7^ (*a* ^2^F)5*s*, *d* ^3^F_2_ and *d* ^3^F_3_. The average deviation of the remaining 18 levels of the 4*d*^6^ 5*s*^2^ configuration from their predicted positions is 45 cm^−1^.

All terms of the 4*d*^8^ configuration have been found with the exception of ^1^S This term should occur at about 38892 cm^−1^. The *c* ^3^P_o_ level does not agree well with Trees’ calculations. The evidence for this level consists of only three transitions, but since extensive searching revealed no other level with *J*=0 in this region, it was kept in the list will a question mark. The remaining seven levels deviate by less than 37 cm^−1^ from Trees’ predictions. The only term from this configuration known in Fe I lies 23500 cm^−1^ above the corresponding term, *b* ^3^F, in Ru I.

Only the terms *e* ^7^D and *f* ^5^D from the 4*d*^6^ 5*^s^* (*a* ^6^D) 6*s* configuration are known, and the latter is incomplete. They are about 6000 cm^−1^ higher than the corresponding terms in Fe I.

The remaining even configurations observed involve a 5*d* electron. The terms with this configuration are found above 47000 cm^−1^ and are difficult to find with certainty because of the limited number of expected transitions. Six of the ten predicted terms with the configuration 4*d*^7^ (*a* ^4^F) 5*d* are represented in the tables, but only one, *e* ^3^G, is complete. They lie about 6000 cm^−1^ below those in Fe I. Data on the remaining 5*d* configurations are extremely fragmentary. Two terms, *e* ^5^S and part of ƒ ^7^D are known and tentatively assigned to 4*d*^6^ 5*s* (*a* ^6^D) 5*d*. They lie almost coincident with the similar terms in Fe I.

A possible level at 55405.64 cm^−1^ may be the missing 4*d*^7^ (*a* ^4^P) 5*_p_* $f{\;}^{3}D_{3}^{{}^{\circ}}$ level. A transition from a level at this position to $y{\;}^{3}D_{3}^{{}^{\circ}}$ would coincide with the position of the strongest remaining unclassified line at 5361 A. Very weak lines at the wavelengths corresponding to combinations with three otherlevels, $y{\;}^{5}P_{3}^{{}^{\circ}}$, $y{\;}^{5}P_{2}^{{}^{\circ}}$, and $6_{3}^{{}^{\circ}}$, have been found. These combinations may, however, be coincidental and the level has not been listed in table 1.

The strongest remaining unclassified lines lie between 4700 and 7500 A. Many of these probably represent transitions between unidentified terms with a 4*d*^7^ (*a* ^4^F) 5*d*, 4*d*^7^ (*a* ^4^P) 5*d*, or a 4*d*^6^ 5*s* (*a* ^6^D) 5*d* configuration and odd terms from the same limits. These multiplets are characterized by strong transitions on the main diagonal with very weak off-diagonal transitions. The corresponding energy levels are, therefore, difficult to find.

### 3.2. Odd Terms

All terms of the 4*d*^7^ 5*p* configuration having as limits in Ru II *a* ^4^F, *a* ^4^P, *a* ^2^G, *a* ^2^D, and *a* ^2^H have been found. The intervals are approximately three times as large as those in Fe I. Those from *a* ^4^F lie about 5500 cm^−1^ below the corresponding terms in Fe I. The two levels $z{\;}^{5}G_{3}^{{}^{\circ}}$ and $z{\;}^{5}G_{2}^{{}^{\circ}}$ appear to be perturbed downward.

In the next group, i.e., 4*d*^7^(*a* ^4^*P*)5*p*, the lowest term, *z* ^5^S°, is 13326 cm^−1^ below the analogous term in Fe I; the rest are about 9000 cm^−1^ lower. Also, as in Fe I, the levels of *x* ^5^D*°* appear to be strongly perturbed.

For 4*d*^7^*(a* ^2^G)5*p*, the terms are about 8000 cm^−1^ below those in Fe I, and the intervals of the terms *y* ^3^G° and *z* ^3^H° show evidence of severe perturbation.

In the 4*d*^7^(*a* ^2^P)5*p* configuration, all levels lie about 9000 em^−1^ below those that have been found in Fe I. The intervals of the *x* ^3^D° term show evidence of strong perturbation.

The terms of the configuration 4*d*^7^(*a* ^2^H)5*p* lie about 8000 cm^−1^ below those in Fe I. The intervals of *z* ^3^I° indicate strong perturbation.

The levels $x{\;}^{3}P_{1}^{{}^{\circ}}$ and $x{\;}^{3}P_{0}^{{}^{\circ}}$ of the 4*d*^7^(*a*^2^D)5*p* configuration are missing. The remaining terms range 6000 to 15000 cm^−1^ below those in Fe I, and the intervals of the *x*^3^F° term indicate a strong perturbation.

Only two terms, *x* ^1^P° and *x* ^1^G° of the 4*d*^7^ (a ^2^F)5*p* configuration are suggested and these assignments are tentative. No levels of this configuration have been classified in Fe I.

For the configuration 4*d*^6^ 5*s*5*p* the two triads of septet and quintet terms having as limit *a* ^6^D in Ru II are well known. These terms are regular and lie from 6500 to 8500 cm^−1^ above the related terms in Fe I.

For the 4*d*^6^ 5*s*(*a*^4^D)5*p* configuration, only two terms, w ^5^D° and *x* ^5^F*°* have been designated and the level *x* ^5^F_1_ is missing. These terms lie about 5000 cm^−1^ above those in Fe I. Similarly, only two terms, *y* ^5^S° and *x* ^5^p° are ascribed to 4*d*^6^ 5*s*(*b*^4^P)5*p*. They are about 4000 cm^−1^ above those in Fe I. The unassigned term *t* ^3^D° may belong in this group.

One term, *x* ^1^H° is ascribed to 4*d*^6^ 5*s*(*b* ^2^H)5*p* because of its strong combination with *a* ^1^H.

Five terms are entered in table 1 without configuration assignments: *v* ^3^D°, *u* ^3^D°, *t* ^3^D°, *w* ^3^G*°*, and *v* ^3^G°. All of these are fragmentary but observed *g*-values are known for seven of the levels involved.

The remaining odd levels have been classed as miscellaneous. These levels lie above 40000 cm^−1^ and most of them are probably fragments of the various remaining 4*d*^6^ 5*s*5*p* or 4*d*^7^(*a* ^2^F)5*p* configurations. Some possible assignments, made on the basis of the intensities of the observed combinations, are given in parentheses. Very few Zeeman effect data are available in this range, and the overlapping of terms is so great that configuration and term assignments have little significance.

### 3.3. Ionization Limits

The ionization limit for Ru I was determined by fitting the observed term values for the two series members 4*d*^7^(*a* ^4^F)5*s* $a{\;}^{5}F_{5}(T_{1}^{\prime }=0.0\;{\text{cm}}^{-1})$ and 4*d*^7^(*a* ^4^F)6*s* $e{\;}^{5}F_{5}(T_{2}^{\prime }=41256.40\;{\text{cm}}^{-1})$ to the formulas below. The value of the constant *α*= − 2.43 ×10^−6^ cm used is an interpolated value obtained by Catalan and Rico [16]. This interpolation was obtained by drawing a smooth curve through the points given by the available data on a plot of *α* versus atomic number for the first spectra of the elements Rb through Ag.

$$\begin{array}{l} T_{n}=R/{\left(n+\mu +\alpha T_{n}\right)}^{2}\\ T_{n}=T_{\infty }-T_{n}^{\prime } \end{array}$$

where

*T_n_* is the energy level of the *n*th series member measured relative to the ionization limit, *T_∞_*
$T_{n}^{\prime }$ is the energy level of the *n*th series member measured relative to the ground state,
*μ* is the quantum defect and is assumed to be constant for the series,
*α* is a constant,
*R* is the Rydberg constant, and
*n* is 1 for the first term, 2 for the second term.

This set of equations is satisfied by a unique value of the ionization limit, *T_∞_*. The value of the ionization limit obtained in this manner is 59410 cm^−1^ or 7.364 ev. This value of the ionization limit agrees well with the value 59417 cm^−1^ obtained by Catalan and Rico by interpolation between data from spectra of neighboring elements in the periodic table.

## Figures and Tables

**Table 1:** Ru I

Config.	Desig.	*J*	Level	Interval	Obs. *g*	Earliest reference
						
4*d*^7^(*a* ^4^F)5*s*	*a* ^5^F	5	0.00	−1190.64 −900.90 −621.70 −392.25	1.397	P
		4	1190.64		1.349	P
		3	2091.54		1.249	P
		2	2713.24		1.000	P
		1	3105.49		0.000	ML, S
4*d*^7^(*a* ^4^F)5*s*	*a* ^3^F	4	6545.03	−1539.09 −1099.54	1.284	P
		3	8084.12		1.196	P
		2	9183.66		1.089	P
4*d*^6^ 5*s*^2^	*a* ^5^D	4	7483.07	−1092.35 −482.22 −15.34 −419.39	1.447	P
		3	8575.42		1.420	P
		2	9057.64		1.232	P
		1	9072.98		1.795	ML, S
		0	9492.37		0/0	HM
4*d*^7^ (*a* ^4^P)5*s*	*a* ^5^P	3	8770.93	727.24 −1576.60	1.624	P
		2	8043.69		1.536	P
		1	9620.29		1.985	ML, S
4*d*^8^	*b* ^3^F	4	9120.63	−1533.99 −792.69	1.255	P
		3	10654.62		1.086	P
		2	11447.31		0.764	P
4*d*^7^(*a* ^2^P)5*s*	*a* ^3^P	2	10623.53	−1162.52 33.43	1.534	P
		1	11786.05		1.684	S
		0	11752.62		0/0	S
4*d*^7^(*a* ^2^G)5*s*	*a* ^3^G	5	12207.05	−609.64	1.190	HM
		4	12816.69	−882.38	1.033	S
		3	13699.07		0.757	HM
4*d*^7^(*a* ^4^P)5*s*	*b* ^3^P	2	13645.75	−335.92 −845.83	1.315	P
		1	13981.67		1.441	S
		0	14827.50		0/0	HM
4*d*^7^(*a* ^2^G)5*s*	*a* ^1^G	4	14700.32		0.992	HM
4*d*^7^(*a* ^2^D)5*s*	*a* ^3^D	3	16190.61	1136.54 −1658.51	1.333	HM
		2	15054.07		1.162	S
		1	16712.58		0.676	HM
4*d*^7^(*a* 2H)5*s*	*a* ^3^H	6	15550.16	−689.97 −856.74	1.164	HM
		5	16240.13		1.041	HM
		4	17096.87		0.834	HM
4*d*^7^(*a* ^2^D)5*s*	*a* ^1^D	2	17045.97		1.175	HM
4*d*^7^(*a* ^2^H)5*s*	*a* ^1^H	5	20055.71		1.007	MC
4*d*^7^(*a* ^2^P)5*s*	*a* ^1^P	1	20242.01		0.927	HM
4*d*^8^	*c* ^3^P	2	20933.75	−1358.89 −1881.04?	1.343	HM
		1	22292.64		1.35	K
		0	24173.68?			K
4*d*^6^ 5*s*^2^	*c* ^3^F	4	21643.09	−776.37 76.32	1.08	K
		3	22419.46		1.070	K
		2	22343.14		0.697	K
4*d*^6^ 5*s*^2^	*b* ^3^H	6	22162.06	−356.82 −485.89	1.063	K
		5	22518.88		0.99	K
		4	23004.77		0.91?	K
4*d*^8^	*b* ^1^G	4	23392.60		0.950	K
4*d*^8^	*b* ^1^D	2	23453.47		1.162	K
4*d*^6^ 5*s*^2^	*d* ^3^P	2	24927.48	−2633.11		K
		1	27560.59			K
		0				
4*d*^7^(*a* ^2^F)5*s*	*a* ^1^F	3	25200.97			K
4*d*^6^ 5*s*(*a* ^6^D)5*p*	*z* ^7^D°	5	*25214.16*	−250.33 −571.07 −437.18 −307.72	1.592	HM
		4	*25464.49*		1.625	ML, S
		3	*26035.56*		1.737	S
		2	*26472.74*		1.992	MC
		1	*26780.46*			MC
4*d^6^* 5*s*^2^	*b* ^3^G	5	25602.60	−40.09 −433.01		K
		4	25642.69			K
		3	26075.70			K
4*d*^7^(*a* ^4^F)5*p*	*z* ^5^D°	4	*26312.83*	−1193.76 −959.10 −652.80 −451.41	1.486	ML, S
		3	*27506.59*		1.425	ML, S
		2	*28465.69*		1.324	ML, S
		1	*29118.49*		0.953	ML, S
		0	*29569.90*		0/0	ML
.4*d*^7^(*a* ^4^F)5*p*	*z* ^5^F°	5	*26816.23*	−1198.56 −875.68 −536.85 −266.25	1.394	ML, S
		4	*28014.79*		1.364	ML, S
		3	*28890.47*		1.293	ML, S
		2	*29427.32*		1.164	ML, S
		1	*29693.57*		0.567	ML, S
4*d*^7^(*a* ^2^F)5*s*	*d* ^3^F	4	27289.24	−227.33 700.90		K
		3	27516.57			K
		2	26815.67			K
4*d*^7^(*a* ^4^F)5*p*	*z* ^3^G°	5	*28495.10*	−1395.81 −1961.99	1.230	ML, S
		4	*29890.91*			ML, S
		3	*31852.90*		0.868	ML, S
4*d*^7^(*a* ^4^F)5*p*	*z* ^6^G°	6	*28571.89*	−1707.79 −1066.11 808.73 −421.74	1.379	ML, S
		5	*30279.68*		1.263	ML, S
		4	*31345.79*		1.111	ML, S
		3	*30537.06*		0.944	ML, S
		2	*30958.80*		0.375	ML, S
4*d*^6^ 5*s*(*a* ^6^D)5*p*	*z* ^7^F°	6	*29160.46*	−307.58 −126.52 −297.34 − 126.44 −67.04	1.462	MC
		5	*29468.04*		1.474	ML
		4	*29594.56*		1.370	HM
		3	*29891.90*			HM
		2	*30018.34*		1.497	HM
		1	*30086.38*			S
		0	*30115.25*	−29.87	0/0	MC
4*d*^6^ 5*s*^2^	*b* ^3^D	1				
		2	29352.41	626.59		K
		3	29979.00			K
4*d*^6^ 5*s*^2^	*a* ^1^I	6	29677.10			K
4*d*^6^ 5*s* (*a* ^6^D)5*p*	*z* ^7^P°	4	*30250.40*	−1134.24 −958.66	1.656	S
		3	*31384.64*		1.895	S
		2	*32343.30*		2.059	S
4*d*^7^ (*a* ^4^F)5*p*	*z* ^3^F°	4	*30348.45*	−2043.50 −780.07	1.276	ML, S
		3	*32391.95*		1.133	ML, S
		2	*33172.02*		1.026	S
4*d*^7^ (*a* ^4^F)5*p*	*z* ^3^D°	3	*31044*.*35*	−1163.30 −1372.57	1.204	S
		2	*32207.65*		1.032	S
		1	*33580.22*		0.522	S
4*d*^7^ (*a* ^4^P)5*p*	*z* ^6^S°	2	*31186.03*		2.034	S
4*d*^6^ *5s* (*a* ^6^D)5*p*	*y* ^5^D°	4	*33446.84*	16.19 −298.01 −362.40 −288.58	1.492	ML, S
		3	*33430.65*		1.496	ML, S
		2	*33728.66*		1.477	S
		1	*34091.06*		1.522	ML, S
		0	*34379.64*		0/0	HM
4*d*^6^ 5*s* (*a* ^6^D)5*p*	*z* ^5^P°	3	*34072.41*	−809.51 −164.85	1.646	S
		2	*34881.92*		1.808	S
		1	*35046.77*		2.385	HM
4*d*^6^ 5*s* (*a* ^6^D)5*p*	*y* ^5^F°	5	*34772.55*	−698.60 −335.47 −157.25 −274.90	1.402	HM
		4	*35471.15*		1.364	ML, S
		3	*35806.62*		1.276	ML; S
		2	*35963.87*		1.069	ML
		1	*36238.77*		0.145	ML
4*d*^7^(*a* ^4^P)5*p*	*x* ^5^D°	4	*36542.62*	−824.40 −300.84 −532.54 398.17	1.481	ML
		3	*37367.02*		1.379	ML, S
		2	*37667.86*		1.442	S
		1	*38200.40*		1.569	S
		0	*37802.23*		0/0	HM
4*d*^7^(*a* ^4^P)5*p*	*y* ^3^D°	3	*36760.34*	−204.94 −654.24	1.426	HM
		2	*36965.28*		1.173	ML, S
		1	*37619.52*		0.756	S
4*d*^7^(*a* ^4^P)5*p*	*z* ^3^P°	2	*37118.90*	−227.84 −126.14	1.469	ML, S
		1	*37346.74*		1.311	HM
		0	*37472.88*		0/0	HM
4*d*^7^(*a* ^2^G)5*p*	*y* ^3^F°	4	*38243.38*	−1190.32	1.107	HM
		3	*39433.70*	−999.53	0.968	S
		2	*40433.23*		0.889	HM
4*d*^7^ (*a* ^2^G)5*p*	*z* ^3^H°	6	*38897.50*	600.41 −976.19	1.174	MC
		5	*38297.09*		1.048	HM
		4	*39273.28*		0.895	HM
4*d*^7^ (*a* ^4^P)5*p*	*z* ^3^S°	1	*38587.14*		1.566	S
4*d*^7^(*a* ^4^P)5*p*	*y* ^5^P°	3	*38706.36*	−302.26 −764.87	1.631	ML, S
		2	*39008.62*		1.713	S
		1	*39773.49*		2.315	HM
4*d*^7^(*a* ^2^G)5*p*	*z* ^1^G°	4	*39037.18*		1.115	S
4*d*^7^(*a* ^2^G)5*p*	*y* ^3^G°	5	*39450.66*	−825.95 41.22	1.142	HM
		4	*40276.61*		1.035	S
		3	*40235.39*		0.890	S
*4d*^7^(*a* ^2^P)5*p*	*y* ^3^P°	2	*39742.03*	−174.51 22.04	1.299	S
		1	*39916.54*		1.606	S
		0	*39894.50*		0/0	HM
	1°(^3^F°)	4	*40439.25*		1.196	S
4*d*^7^(*a* ^2^G)5*p*	*z* ^1^H°	5	*40616.22*		1.020	MC
4*d*^7^ (*a* ^2^P)5*p*	*x* ^3^D°	3	*40768.15*	−1239.11 990.61	1.159	ML, S
		2	*42007.26*		1.007	S
		1	*41016.65*		0.895	HM
4*d*^7^(*a* ^2^G)5*p*	*z* ^1^F°	3	*40948.65*		1.137	S
4*d*^7^*(a* ^2^D)5*p*	*x* ^3^F°	2	*41182.94*	77.10 1086.86	0.877	S
		3	*41260.04*		1.235	S
		4	*42346.90*		1.247	HM
4*d*^7^(*a* ^4^F)6*s*	*e* ^5^F	5	41256.40	−1762.17		MC
		4	43018.57	−1157.66 284.14 −451.82		MC
		3	44176.23			MC
		2	43892.09			S
		1	44343.91			MC
4*d*^7^(*a* ^2^D)5*p*	*w* ^3^D°	3	*41482.66*	−1051.15	1.286	S
		2	*42533.81*	−360.61	1.025	ML
		1	*42894.42*		0.810	HM
4*d*^7^(*a* ^2^H)5*p*	*z* ^3^I°	7	*42260.53*	682.78 −1400.53	1.146	MC
		6	*41577.75*		1.013	HM
		5	*42978.28*		0.861	MC
4*d*^7^(*a* ^2^H)5*p*	*x* ^3^G°	5	*41739.30*	−1199.82	1.197	MC
		4	*42939.12*	−1036.67	1.07?	K
		3	*43975.79*		0.934	S
4*d*^7^(*a* ^2^D)5*p*	*z* ^1^D°	2	*41756.15*		1.182	HM
4*d*^7^(*a* ^4^F)6*s*	*e* ^3^F	4	41825.23	−1290.24 −1854.57		MC
		3	43115.47			S
		2	44970.04			S
	*v* ^3^D°	3	*41880.85*	−1016.38	1.163	S
		2	*42897.23*			K
		1				
4*d*^7^(*a* ^2^H)5*p*	*z* ^1^I°	6	*42404.14?*			K
4*d*^7^(*a* ^2^P)5*p*	*z* ^1^P°	1	*42415.81*		0.965	HM
4*d*^7^(*a* ^2^P)5*p*	*z* ^1^S°	0	*42620.80*		0/0	HM
4*d*^6^ 5*s*^2^	*f* ^3^F	4	42895.39			S
4*d*^7^(*a* ^2^D)5*p*	*y* ^1^F°	3	*42998.31*		0.995	HM
4*d*^7^(*a* ^2^P)5*p*	*y* ^3^S°	1	*43107.52*		1.533	MC
	*u* ^3^D°	3				
		2	*43509.17*	−332.36	1.158	S
		1	*43841.53*		0.800	HM
4*d*^7^(*a* ^2^H)5*p*	*y* ^3^H°	6	*43548.67*	−560.74	1.162	K
		5	*44109.41*	−552.60	1.033	MC
		4	*44662.01*		0.925	MC
4*d*^7^(*a* ^2^H)5*p*	*y* ^1^H°	5	*43596.58*		1.03	K
	*w* ^3^G°	5	*43742.81*	−120.10	1.272	ML
		4	*43862.91*			K
		3				
4*d*^7^(*a* ^2^P)5*p*	*y* ^1^D°	2	*43903.41*		1.026	MC
	2°(^5^G°)	6	*43998.60*		1.219	MC
4*d*^7^(*a* ^2^D)5*p*	*x* ^3^P°	2	*44234.68*		1.422	S
		1				
		0				
4*d*^6^ 5*s*(*a* ^4^D)5*p*	*w* ^5^D°	4	*44243.49*	−827.91 −719.01 −400.99	1.473	ML, S
		3	*45071.40*		1.449	ML, S
		2	*45790.41*		1.484	ML, S
		1	*46191.40*	−274.95	1.439	HM
		0	*46466.35*			K
	3°(^3^P°)	1	*44301.14*		1.350	HM
4*d*^6^ 5*s*(*a* ^4^D)5*p*	*x* ^5^F°	5	*44321.81*	−285.80 −193.20	1.303	ML
		4	*44607.61*			ML
		3	*44800.81*	−791.52		ML
		2	*45592.33*			K
		1				
	4°	3	*4444.59*		0.76	MC
	5°(^5^G°)	2	*44891.40*		0.383	ML
4*d*^6^ 5*s*(*b* ^4^P)5*p*	*y* ^5^S°	2	*45197.37*		2.224	K
4*d*^7^(*a* ^2^F)*5p*	*x* ^1^F*°*	3	*45021.98*		1.059	MC
4*d*^7^(*a* ^2^H)5*p*	*y* ^1^G°	4	*45364*.*72*		0.962	MC
	6°	3	*45475.77*		1.547	MC
4*d*^7^(*a* ^2^F)5*p*	*x* ^4^G°	4	*45528.61*		1.08	MC
	7°	3	*45549.51*			
4*d*^7^(*a* ^4^F)6*p*	*w* ^3^F°	4	*45755.55*	−1191.03		MC
		3	*46946.58*	−301.40	1.022	S
		2	*47247.98*		0.702	MC
	*t* ^3^D°	3				
		2	*45923.36*	−576.34	1.089	HM
		1	*46499.70*		0.72	MC
	8°	1	*45978.14*		0.44	K
	9°	1	*46056.23*		1.155	MC
	10°	4	*46067.24*			MC
	11°	0	*46102.93*		0/0	HM
	12°	4	*46273.20*			MC
	13°	4	*46400.58*			ML, S
4*d*^6^ 5*s*(*b* ^2^H)5*p*	*x* ^1^H°	5	*46495.05*		1.030	MC
4*d*^7^(*a* ^2^D)*5p*	*y* ^1^P°	1	*46528.26*		1.05	MC
	14°	4	*46695.02*			MC
4*d*^6^ *5s*(*b* ^4^P)5*p*	*x* ^5^P°	3	*46746.35*	−57.25		MC
		2	*46803.60*	−696.94		MC
		1	*47500.54*			K
	15°	1	*46789.23*			K
4*d*^7^(*a* ^4^F)5*d*	*f* ^5^F	5	46906.32	−1637.56 −265.40		S
		4	48543.88			K
		3	48809.28			K
		2				
		1				
4*d*^7^ (*a* ^4^F)5*d:*	*e* ^5^D	4	46972.42	−215.90 −1532.55 −792.46?	1.36	K
		3	47188.32			MC
		2	48720.87			S
		1	49513.33?			MC
		0				
4*d*^7^(*a* ^4^F)5*d:*	*e* ^5^G	6	46991.15	−1530.62 −51.20	1.35?	K
		5	48521.77			K
		4	49604.93			K
		3	49553.73			MC
		2				
	16°	3	*47046.54*		1.058	MC
4*d*^7^(*a* ^4^F)5*d:*	*e* ^3^G	5	47084.80	−1642.88 −948.29	1.19	S
		4	48727.68			K
		3	49675.97			K
	17°	4	*47157.28*		1.24	MC
	18°(^5^D°)	0	*47176.90*			K
	19°	4	*47261.52*		1.12	MC
4*d*^7^(*a* ^4^F)6*p:*	*s* ^3^D°	3	*47339.32*	−208.01	1.377	MC
		2	*47547.33*		1.057	MC
		1				
	20°(^5^D°)	2	*47345.10*		1.55	MC
4*d*^7^(*a* ^4^F)5*p:*	*e* ^3^P	2	47458.76			
	21°	5	*47425.17*			K
	B (^3^F)	4	47486.96			S
	22°	3	*47526.09*		1.134	MC
	23°	3	*47635.33*		1.06	MC
	24°	5	*47642.87*			K
	25°	3	*47788.72*			MC
	26°	1	*47809.11*			MC
	27°	4	*47817.84*		1.32?	MC
	28°	3	*47868.35*		1.309	MC
	29°	3	*48003.00*			MC
	30°	4	*48109.32*			MC
	31°	4	*48143.98*			MC
	32°	2	*48164.79*			MC
	33°	2	*48326.73*		1.08	K
4*d*^6^ 5*s* (*a* ^6^D)6*s*	*e* ^7^D	5	48386.33	−848.82 −781.55 −523.11 −79.09	1.602	K
		4	49235.15		1.649	K
		3	50016.70			K
		2	50539.81			K
		1	50618.88			K
	34°	3	*48405.09*		1.14	MC
4*d*^7^ (*a* ^4^F)5*d:*	*e* ^3^D	3	48489.66	−1268.22	1.28	K
		2	49757.88			MC
		1				
	35°	3	*48493.01*		1.14?	MC
	36°	5	*48503.30*			MC
	37°	3?	*48570.85*			MC
	38°	4	*48597.45*		1.208	MC
	39°	1	*48604*.*34*		2.06	MC
	40°	3	*48765.88*			MC
	41°	2	*48779.15*			MC
	42°	4	*48853.69*		1.23	K
	43°	3	*48933.93*			MC
	44°	2	*49037.35*			K
	45°	1	*49047.61*			MC
	46°	3	*49141.42*		1.03?	MC
	47°	4	*49165.05*			MC
4*d*^7^(*a* ^4^P)5*d*	*e* ^5^P	3	49291.06	−881.78		K
		2	50172.84			K
		1				
	48°	3	*49303.88*?			K
	49°	1	*49408.97*			K
	50°	2	*49417.50*			K
	51°	4?	*49447.53*			K
4*d*^6^ 5*s* (*a* ^6^D)5*d*	*e* ^5^S	2	49489.56			K
	C (^7^F)	5	49592.90			K
4*d*^6^ 5*s*(*a* ^6^D)5*d:*	*f* ^7^D	5				
		4	49624.26			K
		3				
		2	51058.64	−1671.16		K
		1	52729.80			K
	52°	4	*49722.18*			K
	53°	1	*49761.61*			K
	54°	3?	*49918.28*?			K
	*v* ^3^G°	3				
		4	*49949.25*	173.16		MC
		5	*50122.41*		1.16	MC
	F	1	49953.01?			K
	55°	2	*49970.65*			MC
	56°	2	*50027.96*			MC
	57°	1	*50192.07*			MC
	58°	1	*50338.99*			MC
4*d*^6^ 5*s*(*a* ^6^D)6*s*	*f* ^5^D	4	50350.52	−1014.30 −636.53		K
		3	51364.82			K
		2	52001.35			K
		1				
		0				
	59°	3	*50351.93*			K
	60°	1	*50772.05*			K
	61°	4	*51360.21*		1.094	MC
	D	3	52455.23			K
	62°	4	*53717.63*			K
	E	4	54043.38			K
	63°	3	54291.55			
Ru II(^4^F4½)	***Limit***	……	**59410**			

**Table 2:** *Ru I* observed terms*

Configuration 1*s*^2^ 2*s*^2^ 2*p*^6^ 3*s*^2^ 3*p*^6^ 3*d*^10^ 4*s*^2^ 4*p*^6^ +	Observed terms																
	
4*d*^8^	$\{\;\begin{array}{c} c^{3}P \end{array}$	*b* ^1^D	*b* ^3^F	*b* ^1^G													
4*d*^6^ 5*s*^2^	$\{\;\begin{array}{c} d^{3}P? \end{array}$	*a* ^5^D *b* ^3^D	*c* ^3^F *f* ^3^F	*b* ^3^G	*b* ^3^H	*a* ^1^I											
	
	*ns* (*n≥* 5)				*np* (*n*≥ 5)							*nd* (*n≥* 5)					
	
4*d*^7^(*a* ^4^F) *nx*	{		*a, e* ^5^F *a, e* ^3^F				*z* ^5^D°	*z* ^5^F°	*z* ^5^G°						*e* ^5^D:	*f* ^5^F:	*e* ^5^G:
		*z*, *s*:^3^D°	*z*, *w*: ^3^F°	*z* ^3^G°				*e* ^3^P:	*e* ^3^D:		*e* ^3^G:						
4*d*^7^(*a* ^4^P) *nx*	$\{\begin{array}{c} a{\;}^{5}P\\ b{\;}^{3}P \end{array}$				*z* ^5^S°	*y* ^5^P°	*x* ^5^D°						*e* ^5^P				
*z* ^3^S°	*z* ^3^P°	*y* ^3^D°															
4*d*^6^ 5*s* (*a* ^6^D) *nx*	{	*e* ^7^D *f* ^5^D				*z* ^7^P°	*z* ^5^P°	*z* ^7^D°				*e* ^5^S		*f* ^7^D:			
	*z* ^7^F°	*y* ^5^D°	*y* ^5^F°														
4*d*^7^(*a* ^2^G) *nx*	{			*a* ^3^G *a* ^1^G					*y* ^3^F°	*y* ^3^G°	*z* ^3^H°						
			*z* ^1^F°	*z* ^1^G°	*z* ^1^H°												
4*d*^7^(*a* ^2^P) *nx*	$\{\begin{array}{c} a{\;}^{3}P\\ a{\;}^{1}P \end{array}$				*y* ^3^S°	*y* ^3^P°	*x* ^3^D°										
*z* ^4^S°	*z* ^1^P°	*y* ^1^D°															
4*d*^7^(*a* ^2^D) *nx*	{	*a* ^3^D *a* ^1^D				*x* ^3^P°	*w* ^3^D°	*x* ^3^F°									
	*y* ^1^P°:	*z* ^1^D°	*y* ^1^F°														
4*d*^7^(*a* ^2^H) *nx*	{			*a* ^3^H *a* ^1^H					*x* ^3^G°	*y* ^3^H°	*z* ^3^I°						
				*y* ^1^G°	*y* ^1^H°	*z* ^1^I°											
4*d*^6^ 5*s* (*a* ^4^D) *nx*								*w* ^5^D°	*x* ^5^F°								
4*d*^7^(*a* ^2^F) *nx*	{		*d* ^3^F: *d* ^1^F:						*x* ^1^F*°:*	*x* ^1^G°:							
4*d*^6^ 5*s*(*b* ^4^P) *nx*						*y* ^5^S°	*s* ^5^P°										
4*d*^6^ 5*s* (*b* ^2^H) *nx*											*x* ^1^H°:						

* For predicted terms in the spectra of the Ru I isoelectronic sequence, see Vol. iii, Introduction.

**Table 3:** 

Intensity	Wave-length	Wave number	Term combination

	*A*	*cm*^−1^	
1	2057.07	48597.28	*a* ^5^F_5_— $38_{4}^{{}^{\circ}}$
2	2059.86	48531.47	*a* ^5^F_4_— $52_{4}^{{}^{\circ}}$
2	2061.06	48503.21	*a* ^5^F_5_— $36_{5}^{{}^{\circ}}$
4	2071.43	48260.43	*a* ^5^F_3_— $59_{3}^{{}^{\circ}}$
3	2076.43	48144.23	*a* ^5^F_5_— $31_{4}^{{}^{\circ}}$
3	2077.77	48113.19	*a* ^5^F_4_— $48_{3}^{{}^{\circ}}$
3	2077.93	48109.49	*a* ^5^F_5_— $30_{4}^{{}^{\circ}}$
1	2080.12	48058.84	*a* ^5^F_2_— $60_{1}^{{}^{\circ}}$
15	2083.78	47974.44	*a* ^5^F_4_— $47_{4}^{{}^{\circ}}$
2	2085.43	47936.48	*a* ^5^F_3_— $56_{2}^{{}^{\circ}}$
6	2090.22	47826.65	*a* ^5^F_3_— $54_{3}^{{}^{\circ}}$
8	2097.24	47666.58	*a* ^5^F_4_— $60_{1}^{{}^{\circ}}$
15	2098.46	47638.87	*a* ^5^F_2_— $59_{3}^{{}^{\circ}}$
15	2098.82	47630.70	*a* ^5^F_3_— $52_{4}^{{}^{\circ}}$
2	2105.53	47478.92	*a* ^5^F_2_— $57_{1}^{{}^{\circ}}$
1	2109.91	47380.38	*a* ^5^F_4_— $37_{3}^{{}^{\circ}}$
2	2112.33	47326.10	*a* ^5^F_3_— $50_{2}^{{}^{\circ}}$
1	2115.21	47261.67	*a* ^5^F_5_— $19_{4}^{{}^{\circ}}$
10	2117.418	47212.39	*a* ^5^F_3_— $48_{3}^{{}^{\circ}}$
2	2117.745	47205.10	*a* ^5^F_2_— $54_{3}^{{}^{\circ}}$
2	2123.67	47073.42	*a* ^5^F_3_— $47_{4}^{{}^{\circ}}$
10	2124.74	47049.72	*a* ^5^F_3_— $46_{3}^{{}^{\circ}}$
5	2129.10	46953.37	*a* ^5^F_4_— $31_{4}^{{}^{\circ}}$
2	2130.67	46918.78	*a* ^5^F_4_— $30_{4}^{{}^{\circ}}$
4	2133.12	46864.90	*a* ^5^F_1_— $55_{2}^{{}^{\circ}}$
2	2135.51	46812.46	*a* ^5^F_4_— $29_{3}^{{}^{\circ}}$
15	2140.88	46695.05	*a* ^5^F_5_— $14_{4}^{{}^{\circ}}$
4	2142.66	46656.26	*a* ^5^F_1_— $53_{1}^{{}^{\circ}}$
7	2144.00	46627.10	*a* ^5^F_4_— $27_{4}^{{}^{\circ}}$
5	2145.328	46598.24	*a* ^5^F_4_— $25_{3}^{{}^{\circ}}$
6	2149.59	46505.86	*a* ^5^F_3_— $38_{4}^{{}^{\circ}}$
5	2150.09	46495.05	*a* ^5^F_5_*—x* ${\;}^{1}H_{5}^{{}^{\circ}}$
15	2150.824	46479.19	*a* ^5^F_3_— $37_{3}^{{}^{\circ}}$
15	2152.411	46444.92	*a* ^5^F_4_— $23_{3}^{{}^{\circ}}$
5	2157.54	46334.52	*a* ^5^F_2_— $45_{1}^{{}^{\circ}}$
10	2160.394	46273.32	*a* ^5^F_5_— $12_{4}^{{}^{\circ}}$
15	2162.202	46234.63	*a* ^5^D_4_— $62_{4}^{{}^{\circ}}$
2	2162.855	46220.67	*a* ^5^F_2_— $43_{3}^{{}^{\circ}}$
10	2166.231	46148.65	*a* ^5^F_4_—*s* ${\;}^{3}D_{3}^{{}^{\circ}}$
15	2169.775	46073.27	*a* ^5^F_3_— $32_{2}^{{}^{\circ}}$
7	2170.060	46067.23	*a* ^5^F_5_— $10_{4}^{{}^{\circ}}$
7	2170.126	46065.83	*a* ^5^F_2_— $41_{2}^{{}^{\circ}}$
5	2172.396	46017.70	*a* ^5^F_3_— $30_{4}^{{}^{\circ}}$
10	2174.811	45966.60	*a* ^5^F_4_— $17_{4}^{{}^{\circ}}$
12	2175.978	45941.95	*a* ^5^F_1_— $45_{1}^{{}^{\circ}}$
10	2176.452	45931.95	*a* ^5^F_1_— $44_{2}^{{}^{\circ}}$
12	2177.43	45911.32	*a* ^5^F_3_— $29_{3}^{{}^{\circ}}$
6	2178.389	45891.11	*a* ^5^F_2_— $39_{1}^{{}^{\circ}}$
5	2180.06	45855.94	*a* ^5^F_4_— $16_{3}^{{}^{\circ}}$
20	2183.687	45779.78	*a* ^5^F_2_— $35_{3}^{{}^{\circ}}$
10	2183.829	45776.80	*a* ^5^F_3_— $28_{3}^{{}^{\circ}}$
10	2184.835	45755.73	$\{\;\begin{array}{l} a{\;}^{5}F_{5}—w{\;}^{3}F_{4}^{{}^{\circ}}\\ a{\;}^{5}F_{4}—w{\;}^{3}F_{3}^{{}^{\circ}} \end{array}$
2	2187.632	45697.23	*a* ^5^F_3_— $25_{3}^{{}^{\circ}}$
20	2188.754	45673.81	*a* ^5^F_1_— $41_{2}^{{}^{\circ}}$
15	2194.428	45555.73	*a* ^5^F_4_*—x* ${\;}^{5}P_{3}^{{}^{\circ}}$
12	2195.733	45528.66	*a* ^5^F_5_*—x* ${\;}^{1}G_{4}^{{}^{\circ}}$
10	2196.120	45520.63	*a* ^5^P_3_— $63_{3}^{{}^{\circ}}$
20	2196.901	45504.45	*a* ^5^F_4_— $14_{4}^{{}^{\circ}}$
6	2199.459	45451.54	*a* ^5^F_2_— $32_{2}^{{}^{\circ}}$
15	2200.280	45434.58	*a* ^5^F_3_— $22_{3}^{{}^{\circ}}$
8	2203.67	45364.69	*a* ^5^F_5_—*y* ${\;}^{1}G_{4}^{{}^{\circ}}$
1	2206.61	45304.26	*a* ^5^F_4_—*x* ${\;}^{1}H_{5}^{{}^{\circ}}$
40	2209.08	45253.60	*a* ^5^F_3_— $20_{2}^{{}^{\circ}}$
1	2209.356	45247.95	*a* ^5^F_3_—*s* ${\;}^{3}D_{3}^{{}^{\circ}}$
4	2210.655	45221.37	*a* ^5^F_1_— $33_{2}^{{}^{\circ}}$
3	2211.214	45209.94	*a* ^5^F_4_— $13_{4}^{{}^{\circ}}$
4	2213.823	45156.66	*a* ^5^F_3_—*w* ${\;}^{3}F_{2}^{{}^{\circ}}$
3	2217.46	45082.60	*a* ^5^F_4_— $12_{4}^{{}^{\circ}}$
2	2223.749	44955.12	*a* ^5^F_3_— $16_{3}^{{}^{\circ}}$
4	2224.162	44946.77	*a* ^5^P_3_— $62_{4}^{{}^{\circ}}$
25	2227.640	44876.60	*a* ^5^F_4_— $10_{4}^{{}^{\circ}}$
3	2228.702	44855.22	*a* ^5^F_3_—*w* ${\;}^{3}F_{3}^{{}^{\circ}}$
1	2229.76	44833.94	*a* ^5^F_2_—*s* ${\;}^{3}D_{2}^{{}^{\circ}}$
4	2230.708	44814.89	*a* ^3^F_4_— $61_{4}^{{}^{\circ}}$
20	2230.804	44812.96	*a* ^5^F_2_— $22_{3}^{{}^{\circ}}$
30	2232.08	44787.35	*a* ^5^F_2_—*x* ${\;}^{5}P_{1}^{{}^{\circ}}$
25	2235.836	44712.11	*a* ^5^F_3_—*x* ${\;}^{5}P_{2}^{{}^{\circ}}$
4	2236.261	44703.61	*a* ^5^F_1_— $26_{1}^{{}^{\circ}}$
50	2238.35	44661.90	*a* ^5^F_5_—*y* ${\;}^{3}H_{4}^{{}^{\circ}}$
2	2238.700	44654.92	*a* ^5^F_3_—*x* ${\;}^{5}P_{3}^{{}^{\circ}}$
6	2239.856	44631.87	*a* ^5^F_2_— $20_{2}^{{}^{\circ}}$
50	2241.075	44607.60	*a* ^5^F_5_—*x* ${\;}^{5}F_{4}^{{}^{\circ}}$
2	2241.278	44603.56	*a* ^5^F_3_— $14_{4}^{{}^{\circ}}$
60	2243.23	44564.75	*a* ^5^F_4_—*w* ${\;}^{3}F_{4}^{{}^{\circ}}$
15	2249.44	44441.73	*a* ^5^F_1_—*s* ${\;}^{3}D_{2}^{{}^{\circ}}$
6	2251.820	44394.77	*a* ^5^F_1_—*x* ${\;}^{5}P_{1}^{{}^{\circ}}$
50	2253.65	44358.72	*a* ^5^F_4_— $7_{3}^{{}^{\circ}}$
30	2254.71	44337.87	*a* ^5^F_4_—*x* ${\;}^{1}G_{4}^{{}^{\circ}}$
4	2254.953	44333.09	*a* ^6^F_2_— $16_{3}^{{}^{\circ}}$
80	2255.53	44321.75	*a* ^5^F_5_—*x* ${\;}^{5}F_{5}^{{}^{\circ}}$
40	2256.187	44308.85	*a* ^5^F_3_— $13_{4}^{{}^{\circ}}$
1	2257.40	44285.04	*a* ^5^F_4_— $6_{3}^{{}^{\circ}}$
100	2259.529	44243.31	*a* ^5^F_5_—*w* ${\;}^{5}D_{4}^{{}^{\circ}}$
3	2263.079	44173.92	*a* ^5^F_4_—*y* ${\;}^{1}G_{4}^{{}^{\circ}}$
20	2264.696	44142.38	*a* ^5^F_1_—*w* ${\;}^{3}F_{2}^{{}^{\circ}}$
1	2266.41	44109.00	*a* ^5^F_5_*—y* ${\;}^{3}H_{5}^{{}^{\circ}}$
2	2267.379	44090.15	*a* ^5^F_2_—*x* ${\;}^{5}P_{2}^{{}^{\circ}}$
40	2268.34	44071.47	*a* ^5^F1— $18_{0}^{{}^{\circ}}$
20	2270.322	44033.00	*a* ^5^F_2_—*x* ${\;}^{5}P_{3}^{{}^{\circ}}$
1	2271.446	44011.22	*a* ^5^F_4_—*x* ${\;}^{1}F_{3}^{{}^{\circ}}$
100	2272.091	43998.72	*a* ^5^F_5_— $2_{6}^{{}^{\circ}}$
50	2278.198	43880.79	*a* ^5^F_4_—*w* ${\;}^{5}D_{3}^{{}^{\circ}}$
60	2285.382	43742.87	*a* ^5^F_5_—*w* ${\;}^{3}G_{5}^{{}^{\circ}}$
60	2287.695	43698.65	*a* ^5^F_3_—*w* ${\;}^{5}D_{2}^{{}^{\circ}}$
1	2288.478	43683.69	*a* ^5^F_1_— $15_{1}^{{}^{\circ}}$
40	2292.333	43610.24	*a* ^5^F_4_—*x* ${\;}^{5}F_{3}^{{}^{\circ}}$
30	2293.044	43596.72	*a* ^5^F_5_—*y* ${\;}^{1}H_{5}^{{}^{\circ}}$
50	2294.054	43577.52	*a* ^3^F_4_—*v* ${\;}^{3}G_{5}^{{}^{\circ}}$
40	2295.560	43548.94	*a* ^5^F_5_—*y* ${\;}^{3}H_{6}^{{}^{\circ}}$
8	2298.11	43500.62	*a* ^5^F_3_—*x* ${\;}^{5}F_{2}^{{}^{\circ}}$
60	2299.289	43478.31	*a* ^5^F_2_—*w* ${\;}^{5}D_{1}^{{}^{\circ}}$
40	2299.641	43471.66	*a* ^5^F_4_—*y* ${\;}^{3}H_{4}^{{}^{\circ}}$
50	2300.350	43458.26	*a* ^5^F_3_— $7_{3}^{{}^{\circ}}$
8	2302.226	43422.85	*a* ^5^F_1_—*y* ${\;}^{1}P_{1}^{{}^{\circ}}$
100	2302.533	43417.06	*a* ^5^F_4_—*x* ${\;}^{5}F_{4}^{{}^{\circ}}$
70	2305.517	43360.88	*a* ^5^F_1_—*w* ${\;}^{5}D_{0}^{{}^{\circ}}$
15	2310.03	43276.17	*a* ^3^F_3_— $61_{4}^{{}^{\circ}}$
80	2317.784	43131.40	*a* ^5^F_4_—*x* ${\;}^{5}F_{5}^{{}^{\circ}}$
40	2318.905	43110.56	*a* ^5^F_3_—*x* ${\;}^{1}F_{3}^{{}^{\circ}}$
40	2320.23	43085.94	*a* ^5^F_1_—*w* ${\;}^{5}D_{1}^{{}^{\circ}}$
60	2320.699	43077.23	*a* ^5^F_2_—*w* ${\;}^{5}D_{2}^{{}^{\circ}}$
80	2322.009	43052.93	*a* ^5^F_4_—*w* ${\;}^{5}D_{4}^{{}^{\circ}}$
60	2325.952	42979.95	*a* ^5^F_3_—*w* ${\;}^{5}D_{3}^{{}^{\circ}}$
15	2327.536	42950.71	*a* ^5^F_1_— $9_{1}^{{}^{\circ}}$
2	2329.30	42918.18	*a* ^5^F_4_—*y* ${\;}^{3}H_{5}^{{}^{\circ}}$
60	2331.43	42878.98	*a* ^5^F_2_*—x* ${\;}^{5}F_{2}^{{}^{\circ}}$
40	2331.770	42872.72	*a* ^5^F_1_— $8_{1}^{{}^{\circ}}$
1	2331.98	42868.86	*a* ^5^D_4_— $59_{3}^{{}^{\circ}}$
15	2333.736	42836.67	*a* ^5^F_2_— $7_{3}^{{}^{\circ}}$
40	2335.738	42799.90	*a* ^5^F_3_— $5_{2}^{{}^{\circ}}$
1	2336.53	42785.39	*a* ^5^F_4_—*x* ${\;}^{3}G_{3}^{{}^{\circ}}$
6	2337.98	42758.86	*a* ^3^F_4_— $48_{3}^{{}^{\circ}}$
100	2340.696	42709.25	*a* ^5^F_3_—*x* ${\;}^{5}F_{3}^{{}^{\circ}}$
40	2342.02	42685.11	*a* ^5^F_1_*—w* ${\;}^{5}D_{2}^{{}^{\circ}}$
80	2342.72	42672.35	*a* ^5^F_4_—*w* ${\;}^{3}G_{4}^{{}^{\circ}}$
8	2344.534	42639.34	*a* ^5^D_4_—*v* ${\;}^{3}G_{5}^{{}^{\circ}}$
100	2348.318	42570.64	*a* ^5^F_3_—*y* ${\;}^{3}H_{4}^{{}^{\circ}}$
150	2349.338	42552.16	*a* ^5^F_4_—*w* ${\;}^{3}G_{5}^{{}^{\circ}}$
200	2351.33	42516.11	*a* ^5^F_3_—*x* ${\;}^{5}F_{4}^{{}^{\circ}}$
6	2352.84	42488.83	*a* ^5^F_2_—*x* ${\;}^{1}F_{3}^{{}^{\circ}}$
15	2352.94	42487.02	*a* ^5^F_1_*—x* ${\;}^{5}F_{2}^{{}^{\circ}}$
6	2354.10	42466.08	*a* ^5^D_4_—*v* ${\;}^{3}G_{4}^{{}^{\circ}}$
90	2360.093	42358.26	*a* ^5^F_2_—*w* ${\;}^{5}D_{3}^{{}^{\circ}}$
50	2360.712	42347.16	*a* ^5^F_5_—*x* ${\;}^{3}F_{4}^{{}^{\circ}}$
2	2362.871	42308.46	*a* ^3^F_4_— $42_{4}^{{}^{\circ}}$
1	2365.14	42267.88	*a* ^3^F_3_— $59_{3}^{{}^{\circ}}$
25	2366.743	42239.26	*a* ^5^D_4_— $52_{4}^{{}^{\circ}}$
10	2367.769	42220.95	*a* ^3^F_4_— $40_{3}^{{}^{\circ}}$
80	2370.169	42178.20	*a* ^5^F_2_— $5_{2}^{{}^{\circ}}$
3	2372.13	42143.34	*a* ^5^F_3_—*x* ${\;}^{3}P_{2}^{{}^{\circ}}$
100	2375.272	42087.60	*a* ^5^F_2_—*x* ${\;}^{5}F_{3}^{{}^{\circ}}$
2	2378.75	42026.06	*a* ^3^F_4_— $37_{3}^{{}^{\circ}}$
6	2386.678	41886.47	*a* ^3^F_3_— $55_{2}^{{}^{\circ}}$
2	2387.359	41874.53	*a* ^5^P_2_— $54_{3}^{{}^{\circ}}$
100	2387.881	41865.37	*a* ^3^F_3_—*v* ${\;}^{3}G_{4}^{{}^{\circ}}$
15	2388.188	41859.99	*a* ^3^F_4_— $34_{3}^{{}^{\circ}}$
1	2390.423	41820.86	*a* ^5^D_4_— $48_{3}^{{}^{\circ}}$
90	2392.249	41771.48	*a* ^5^F_3_—*w* ${\;}^{3}G_{4}^{{}^{\circ}}$
150	2392.425	41785.86	*a* ^5^F_1_— $5_{2}^{{}^{\circ}}$
60	2392.963	41776.47	*a* ^5^D_3_— $59_{3}^{{}^{\circ}}$
80	2395.721	41728.38	*a* ^5^F_2_— $4_{3}^{{}^{\circ}}$
3	2396.32	41717.95	*a* ^5^P_2_— $53_{1}^{{}^{\circ}}$
20	2397.406	41699.05	*a* ^5^D_1_— $60_{1}^{{}^{\circ}}$
20	2398.38	41682.12	*a* ^5^D_4_— $47_{4}^{{}^{\circ}}$
80	2399.750	41658.33	*a* ^5^D_4_— $46_{3}^{{}^{\circ}}$
25	2403.173	41598.99	*a* ^3^F_4_— $31_{4}^{{}^{\circ}}$
3	2403.798	41588.18	*a* ^3^F_2_— $60_{1}^{{}^{\circ}}$
1	2404.207	41581.10	*a* ^5^P_3_— $59_{3}^{{}^{\circ}}$
50	2405.180	41564.28	*a* ^3^F_4_— $30_{4}^{{}^{\circ}}$
10	2407.66	41521.47	*a* ^5^F_2_—*x* ${\;}^{3}P_{2}^{{}^{\circ}}$
60	2408.290	41510.61	*a* ^3^G_5_— $62_{4}^{{}^{\circ}}$
10	2411.76	41450.89	*a* ^5^D_4_— $43_{3}^{{}^{\circ}}$
20	2415.005	41395.20	*a* ^5^D_3_— $55_{2}^{{}^{\circ}}$
4	2416.247	41373.92	*a* ^5^D_3_—*v* ${\;}^{3}G_{4}^{{}^{\circ}}$
4	2416.443	41370.57	*a* ^5^D_4_— $42_{4}^{{}^{\circ}}$
5	2416.754	41365.24	*a* ^5^P_2_— $49_{1}^{{}^{\circ}}$
4	2418.065	41342.82	*a* ^5^D_3_— $54_{3}^{{}^{\circ}}$
2	2418.607	41333.56	*a* ^3^F_3_— $50_{2}^{{}^{\circ}}$
30	2419.205	41323.34	*a* ^3^F_4_— $28_{3}^{{}^{\circ}}$
1	2421.580	41282.82	*a* ^5^D_4_— $40_{3}^{{}^{\circ}}$
15	2421.772	41279.54	*a* ^5^D_0_— $60_{1}^{{}^{\circ}}$
5	2422.165	41272.85	*a* ^3^F_4_— $27_{4}^{{}^{\circ}}$
50	2422.574	41265.88	*a* ^5^D_1_— $58_{1}^{{}^{\circ}}$
2	2422.767	41262.59	*a* ^5^F_2_—*x* ${\;}^{3}G_{3}^{{}^{\circ}}$
4	2423.089	41257.11	*a* ^5^P_3_— $56_{2}^{{}^{\circ}}$
30	2423.877	41243.69	*a* ^3^F_4_— $25_{3}^{{}^{\circ}}$
5	2425.299	41219.52	*a* ^3^F_3_— $48_{3}^{{}^{\circ}}$
10	2426.699	41195.74	*a* ^5^F_1_— $3_{1}^{{}^{\circ}}$
10	2428.323	41168.19	*a* ^3^F_2_— $59_{3}^{{}^{\circ}}$
80	2429.594	41146.65	*a* ^5^D_3_— $52_{4}^{{}^{\circ}}$
5	2430.62	41129.28	*a* ^5^F_1_—*x* ${\;}^{3}P_{2}^{{}^{\circ}}$
40	2431.51	41114.23	*a* ^5^D_4_— $38_{4}^{{}^{\circ}}$
90	2432.915	41090.49	*a* ^3^F_4_— $23_{3}^{{}^{\circ}}$
20	2433.064	41087.97	*a* ^5^D_4_— $37_{3}^{{}^{\circ}}$
25	2433.477	41081.00	*a* ^3^F_3_— $47_{4}^{{}^{\circ}}$
40	2434.879	41057.35	*a* ^3^F_3_— $46_{3}^{{}^{\circ}}$
2	2437.078	41020.30	*a* ^5^D_4_— $36_{5}^{{}^{\circ}}$
50	2437.790	41008.33	*a* ^3^F_2_— $57_{1}^{{}^{\circ}}$
6	2438.055	41003.87	*a* ^5^P_2_— $45_{1}^{{}^{\circ}}$
1	2439.408	40981.13	*a* ^3^F_4_— $22_{3}^{{}^{\circ}}$
20	2440.05	40970.35	*a* ^5^D_2_— $56_{2}^{{}^{\circ}}$
20	2440.977	40954.79	*a* ^5^D_1_— $56_{2}^{{}^{\circ}}$
7	2441.200	40951.05	*a* ^5^P_3_— $52_{4}^{{}^{\circ}}$
50	2442.934	40921.98	*a* ^5^D_4_— $34_{3}^{{}^{\circ}}$
1	2443.46	40913.17	*a* ^5^D_2_— $55_{2}^{{}^{\circ}}$
1	2443.86	40906.48	*a* ^5^F_3_—*y* ${\;}^{1}F_{3}^{{}^{\circ}}$
1	2444.189	40900.97	*a* ^3^G_4_— $62_{4}^{{}^{\circ}}$
60	2444.38	40897.78	*a* ^5^D_1_— $55_{2}^{{}^{\circ}}$
30	2444.828	40890.28	*a* ^5^P_2_— $43_{3}^{{}^{\circ}}$
50	2445.43	40880.22	*a* ^3^F_4_— $21_{5}^{{}^{\circ}}$
2	2446.600	40860.67	*a* ^5^D_2_— $54_{3}^{{}^{\circ}}$
2	2447.25	40849.82	*a* ^3^F_3_— $43_{3}^{{}^{\circ}}$
40	2447.439	40846.67	*a* ^5^D_0_— $58_{1}^{{}^{\circ}}$
10	2447.566	40844.54	*a* ^3^F_2_— $56_{2}^{{}^{\circ}}$
2	2448.512	40828.76	*b* ^3^F_4_—*v* ${\;}^{3}G_{4}^{{}^{\circ}}$
30	2450.359	40797.99	*a* ^5^F_1_—*y* ${\;}^{1}D_{2}^{{}^{\circ}}$
100	2450.560	40794.64	*a* ^3^F_4_—*s* ${\;}^{3}D_{3}^{{}^{\circ}}$
2	2452.06	40769.69	*a* ^3^F_3_— $42_{4}^{{}^{\circ}}$
8	2454.555	40728.25	*a* ^5^D_3_— $48_{3}^{{}^{\circ}}$
150	2454.926	40722.10	*a* ^5^P_2_— $40_{3}^{{}^{\circ}}$
30	2456.279	40699.67	*a* ^5^D_0_— $57_{1}^{{}^{\circ}}$
10	2456.946	40688.62	*a* ^5^D_1_— $53_{1}^{{}^{\circ}}$
100	2458.622	40660.89	*a* ^5^D_4_— $31_{4}^{{}^{\circ}}$
30	2460.73	40626.06	*a* ^5^D_4_— $30_{4}^{{}^{\circ}}$
100	2462.943	40589.56	*a* ^5^D_3_— $47_{4}^{{}^{\circ}}$
20	2464.366	40566.12	*a* ^5^D_3_— $46_{3}^{{}^{\circ}}$
100	2464.699	40560.64	*a* ^5^P_2_— $39_{1}^{{}^{\circ}}$
40	24.67.576	40513.35	*a* ^3^F_3_— $38_{4}^{{}^{\circ}}$
3	2468.29	40501.63	*a* ^3^F_4_— $16_{3}^{{}^{\circ}}$
2	2469.20	40486.71	*a* ^3^F_3_— $37_{3}^{{}^{\circ}}$
30	2470.71	40461.96	*a* ^5^D_3_— $44_{2}^{{}^{\circ}}$
50	2471.48	40449.36	*a*^5^P_2_— $35_{3}^{{}^{\circ}}$
40	2472.09	40439.38	*a* ^5^F_5_— $1_{4}^{{}^{\circ}}$
80	2474.029	40407.69	*a* ^5^P_1_— $56_{2}^{{}^{\circ}}$
15	2474.396	40401.69	*a* ^3^F_4_—*w* ${\;}^{3}F_{3}^{{}^{\circ}}$
30	2474.846	40394.35	*a* ^5^F_2_—*y* ${\;}^{3}S_{1}^{{}^{\circ}}$
200H	2475.395	40385.39	*a* ^5^D_4_— $28_{3}^{{}^{\circ}}$
50	2476.32	40370.31	*a* ^5^P_3_— $46_{3}^{{}^{\circ}}$
100	2476.869	40361.36	*a* ^5^P_2_— $34_{3}^{{}^{\circ}}$
1	2477.53	40350.59	*a* ^5^P_1_*—* $55_{2}^{{}^{\circ}}$
1	2477.910	40344.40	*a* ^5^D_1_— $50_{2}^{{}^{\circ}}$
20	2479.353	40320.93	*a* ^3^F_3_— $34_{3}^{{}^{\circ}}$
15	2480.298	40305.57	*a* ^5^D_4_— $25_{3}^{{}^{\circ}}$
2	2481.56	40285.07	*a* ^5^F_2_—*y* ${\;}^{1}P_{3}^{{}^{\circ}}$
3	2481.68	40283.12	*a* ^5^P_2_— $33_{2}^{{}^{\circ}}$
2	2481.981	40278.24	*a* ^5^D_3_— $42_{4}^{{}^{\circ}}$
10	2482.077	40276.68	*a* ^5^F_5_—*y* ${\;}^{3}G_{4}^{{}^{\circ}}$
10	2482.538	40269.20	*a* ^5^D_0_— $53_{1}^{{}^{\circ}}$
3	2486.58	40203.75	*a* ^5^D_3_— $41_{2}^{{}^{\circ}}$
10	2487.390	40190.65	*a* ^5^D_3_— $40_{3}^{{}^{\circ}}$
30	2489.77	40152.24	*a* ^5^D_4_— $23_{3}^{{}^{\circ}}$
70	2489.92	40149.82	*a* ^3^F_4_— $14_{4}^{{}^{\circ}}$
20	2490.441	40141.42	*a* ^5^P_1_— $53_{1}^{{}^{\circ}}$
60	2491.76	40120.17	*a* ^3^F_2_— $48_{3}^{{}^{\circ}}$
80	2494.022	40083.79	*a* ^5^D_2_— $46_{3}^{{}^{\circ}}$
50	2496.56	40043.04	*a* ^5^D_4_— $22_{3}^{{}^{\circ}}$
40	2497.680	40025.09	*a* ^3^F_3_— $30_{4}^{{}^{\circ}}$
30	2497.866	40022.11	*a* ^5^D_3_— $38_{4}^{{}^{\circ}}$
2	2499.550	39995.15	*a* ^5^P_3_— $40_{3}^{{}^{\circ}}$
8	2500.518	39979.66	*a* ^5^D_2_— $44_{2}^{{}^{\circ}}$
30	2500.835	39974.60	*a* ^5^D_1_— $45_{1}^{{}^{\circ}}$
70	2501.48	39964.29	*a* ^5^D_1_— $44_{2}^{{}^{\circ}}$
60	2501.885	39957.82	*a* ^3^F_2_— $46_{3}^{{}^{\circ}}$
25	2502.37	39950.08	*a* ^3^F_4_—*x* ${\;}^{1}H_{5}^{{}^{\circ}}$
15	2502.87	39942.09	*a* ^5^D_4_— $21_{5}^{{}^{\circ}}$
20	2504.40	39917.70	*a* ^5^D_3_— $35_{3}^{{}^{\circ}}$
20	2504.51	39915.94	*a* ^5^F_3_*—x* ${\;}^{3}D_{2}^{{}^{\circ}}$
100	2508.270	39856.11	*a* ^5^D_4_—*s* ${\;}^{3}D_{3}^{{}^{\circ}}$
30	2510.13	39826.58	*a* ^5^P_3_— $38_{4}^{{}^{\circ}}$
1	2510.504	39820.65	*a* ^5^F_2_—*w* ${\;}^{3}D_{2}^{{}^{\circ}}$
50	2510.965	39813.34	*b* ^3^F_4_— $43_{3}^{{}^{\circ}}$
20	2511.98	39797.25	*a* ^5^P_1_— $50_{2}^{{}^{\circ}}$
1	2512.47	39789.49	*a* ^5^F_3_—*v* ${\;}^{3}D_{3}^{{}^{\circ}}$
100	2512.81	39784.11	*a* ^3^F_3_— $28_{3}^{{}^{\circ}}$
10	2513.17	39778.41	*a* ^5^D_4_— $19_{4}^{{}^{\circ}}$
3	2513.98	39765.59	*a* ^5^P_2_— $26_{1}^{{}^{\circ}}$
30	2514.45	39758.16	*a* ^5^F_4_—*z* ${\;}^{1}F_{3}^{{}^{\circ}}$
10	2514.88	39751.36	*a* ^5^D_3_— $33_{2}^{{}^{\circ}}$
80	2515.27	39745.20	*a* ^5^P_2_— $25_{3}^{{}^{\circ}}$
20	2515.98	39733.98	*a* ^3^F_3_— $27_{4}^{{}^{\circ}}$
1	2516.34	39728.30	$\{\;\begin{array}{l} a{\;}^{3}P_{2}—59_{3}^{{}^{\circ}}\\ a{\;}^{3}F_{4}—12_{4}^{{}^{\circ}} \end{array}$
10	2516.73	39722.14	*a* ^5^P_3_— $35_{3}^{{}^{\circ}}$
80	2517.62	39708.10	*a* ^5^D_2_— $40_{3}^{{}^{\circ}}$
80	2521.61	39645.28	*b* ^3^F_4_— $40_{3}^{{}^{\circ}}$
10	2522.318	39634.15	*a* ^5^P_3_— $34_{3}^{{}^{\circ}}$
30	2525.17	39589.39	*a* ^5^D_3_— $32_{2}^{{}^{\circ}}$
50	2525.63	39582.18	*a* ^3^F_2_— $40_{3}^{{}^{\circ}}$
3	2525.92	39577.64	*a* ^5^F*_4_—x* ${\;}^{3}D_{3}^{{}^{\circ}}$
200	2526.82	39563.54	*a* ^5^D_4_— $16_{3}^{{}^{\circ}}$
3	2527.347	39555.29	*a* ^5^D_0_— $45_{1}^{{}^{\circ}}$
10	2527.60	39551.33	*a* ^3^F_3_— $23_{3}^{{}^{\circ}}$
50	2528.71	39533.97	*a* ^5^D_3_— $30_{4}^{{}^{\circ}}$
75	2528.874	39531.40	*a* ^5^D_1_— $39_{1}^{{}^{\circ}}$
7	2529.456	39522.31	*a* ^3^F_4_— $10_{4}^{{}^{\circ}}$
75	2530.64	39503.82	*a* ^5^P_2_*—s* ${\;}^{3}D_{2}^{{}^{\circ}}$
8	2532.01	39482.45	*a* ^5^P_2_— $22_{3}^{{}^{\circ}}$
1	2532.36	39476.99	*b* ^3^F_4_— $38_{4}^{{}^{\circ}}$
100	2533.23	39463.44	*a* ^5^D_4_—*w* ${\;}^{3}F_{3}^{{}^{\circ}}$
1	2533.65	39456.89	*a* ^5^P_2_—*x* ${\;}^{5}P_{1}^{{}^{\circ}}$
7	2534.605	39442.03	*a* ^3^F_3_— $22_{3}^{{}^{\circ}}$
10	2535.03	39435.41	*a* ^5^D_2_— $35_{3}^{{}^{\circ}}$
20	2535.966	39420.86	*a* ^3^F_2_— $39_{1}^{{}^{\circ}}$
20	2536.20	39417.22	*a* ^5^P_1_— $44_{2}^{{}^{\circ}}$
5	2537.697	39393.97	*a* ^5^P_3_— $32_{2}^{{}^{\circ}}$
10	2537.88	39391.13	*a* ^5^F_3_—*w* ${\;}^{3}D_{3}^{{}^{\circ}}$
6	2538.42	39382.75	*b* ^3^F_4_— $36_{5}^{{}^{\circ}}$
25	2539.09	39372.36	*b* ^5^F_4_— $35_{3}^{{}^{\circ}}$
3	2540.68	39347.73	*a* ^5^D_2_— $34_{3}^{{}^{\circ}}$
70	2541.28	39338.44	*a* ^5^P_3_— $30_{4}^{{}^{\circ}}$
10	2543.16	39309.36	*a* ^3^F_2_— $35_{3}^{{}^{\circ}}$
50	2543.67	39301.47	*a* ^5^P_2_— $20_{2}^{{}^{\circ}}$
300	2544.22	39292.98	*a* ^5^D_3_— $28_{3}^{{}^{\circ}}$
1	2545.48	39273.53	*a* ^5^F_5_—*z* ${\;}^{3}H_{4}^{{}^{\circ}}$
30	2545.76	39269.21	*a* ^5^D_2_— $33_{2}^{{}^{\circ}}$
15	2546.14	39263.35	*a* ^5^D_4_—*x* ${\;}^{5}P_{3}^{{}^{\circ}}$
2	2546.285	39261.12	*a* ^3^F_3_— $20_{2}^{{}^{\circ}}$
150	2546.668	39255.21	*a* ^3^F_3_—*s* ${\;}^{3}D_{3}^{{}^{\circ}}$
2	2547.080	39248.86	*a* ^5^F_4_— $1_{4}^{{}^{\circ}}$
20	2547.48	39242.70	*a* ^5^D_3_— $27_{4}^{{}^{\circ}}$
3	2548.16	39232.23	*a* ^5^P_3_— $29_{3}^{{}^{\circ}}$
4	2548.856	39221.52	*a* ^3^F_2_— $34_{3}^{{}^{\circ}}$
300	2549.470	39212.07	*a* ^5^D_4_— $14_{4}^{{}^{\circ}}$
300	2549.56	39210.69	*a* ^3^F_4_—*w* ${\;}^{3}F_{4}^{{}^{\circ}}$
30	2549.965	39204.46	*a* ^5^P_2_—*w* ${\;}^{3}F_{3}^{{}^{\circ}}$
60	2551.72	39177.50	*a* ^3^F_3_— $19_{4}^{{}^{\circ}}$
25	2552.295	39168.67	*a* ^5^F_3_—*x* ${\;}^{3}F_{3}^{{}^{\circ}}$
1	2552.94	39158.78	*a* ^5^P_1_— $41_{2}^{{}^{\circ}}$
1	2553.288	39153.44	*a* ^3^G_5_— $61_{4}^{{}^{\circ}}$
20	2553.971	39142.97	*a* ^3^F_2_— $33_{2}^{{}^{\circ}}$
40	2556.004	39111.84	*a* ^5^D_0_— $39_{1}^{{}^{\circ}}$
50	2556.316	39107.07	*a* ^5^D_2_— $32_{2}^{{}^{\circ}}$
4	2556.948	39097.40	*a* ^5^P_3_— $28_{3}^{{}^{\circ}}$
10	2557.701	39085.89	*a* ^5^F_4_—*y* ${\;}^{3}G_{4}^{{}^{\circ}}$
100	2558.540	39073.07	*a* ^3^F_3_— $17_{4}^{{}^{\circ}}$
5	2559.406	39059.85	*a* ^5^D_3_— $23_{3}^{{}^{\circ}}$
125	2560.265	39046.75	*a* ^5^P_3_— $27_{4}^{{}^{\circ}}$
10	2561.803	39023.31	*b* ^3^F_4_— $31_{4}^{{}^{\circ}}$
1	2562.168	39017.75	*a* ^5^P_3_— $25_{3}^{{}^{\circ}}$
3	2562.55	39011.93	*a* ^5^D_4_—*x* ${\;}^{1}H_{5}^{{}^{\circ}}$
50	2563.157	39002.70	*a* ^5^P_2_— $16_{3}^{{}^{\circ}}$
10	2564.39	38983.95	*a* ^5^P_1_— $39_{1}^{{}^{\circ}}$
10	2564.41	38983.64	*a* ^3^F_4_—*x* ${\;}^{1}G_{4}^{{}^{\circ}}$
20	2564.587	38980.95	*a* ^3^F_2_— $32_{2}^{{}^{\circ}}$
20	2565.188	38971.82	*a* ^5^D_3_—*s* ${\;}^{3}D_{2}^{{}^{\circ}}$
10	2565.804	38962.46	*a* ^2^F_3_— $16_{3}^{{}^{\circ}}$
30	2566.590	38950.53	*a* ^5^D_3_— $22_{3}^{{}^{\circ}}$
40	2567.893	38930.77	*a* ^3^F_4_— $6_{3}^{{}^{\circ}}$
75	2568.772	38917.45	*a* ^5^D_4_— $13_{4}^{{}^{\circ}}$
50	2569.729	38902.95	*a* ^5^P_2_—*w* ${\;}^{3}F_{3}^{{}^{\circ}}$
30	2570.093	38897.44	*a* ^5^F_5_—*z* ${\;}^{3}H_{6}^{{}^{\circ}}$
100	2572.282	38864.35	*a* ^5^P_3_— $23_{3}^{{}^{\circ}}$
100	2572.412	38862.38	*a* ^3^F_3_—*w* ${\;}^{3}F_{3}^{{}^{\circ}}$
30	2575.242	38819.68	*a* ^3^F_4_—*y* ${\;}^{1}G_{4}^{{}^{\circ}}$
20	2576.954	38793.89	*a* ^3^P_2_— $50_{2}^{{}^{\circ}}$
2	2577.518	38785.40	*a* ^3^P_2_— $49_{1}^{{}^{\circ}}$
100	2578.571	38769.56	$\{\;\begin{array}{l} a{\;}^{5}F_{2}—w{\;}^{3}D_{3}^{{}^{\circ}}\\ a{\;}^{5}D_{3}—20_{2}^{{}^{\circ}} \end{array}$
20	2578.948	38763.90	*a* ^5^D_3_—*s* ${\;}^{3}D_{3}^{{}^{\circ}}$
40	2579.022	38762.78	*b* ^3^F_3_— $50_{2}^{{}^{\circ}}$
70	2579.222	38759.78	*a* ^5^P_2_—*x* ${\;}^{5}P_{2}^{{}^{\circ}}$
100	2579.536	38755.06	*a* ^5^P_3_— $22_{3}^{{}^{\circ}}$
10	2579.777	38751.44	*a* ^5^D_2_— $26_{1}^{{}^{\circ}}$
10	2580.026	38747.70	*b* ^3^F_4_— $28_{3}^{{}^{\circ}}$
100	2580.803	38736.04	*a* ^5^D_1_— $26_{1}^{{}^{\circ}}$
125	2581.140	38730.98	*a* ^5^D_2_— $25_{3}^{{}^{\circ}}$
50	2581.911	38719.41	*a* ^3^F_3_—*x* ${\;}^{5}P_{2}^{{}^{\circ}}$
3	2582.785	38706.31	*a* ^5^P_1_— $33_{2}^{{}^{\circ}}$
70	2583.033	38702.60	*a* ^5^P_2_—*x* ${\;}^{5}P_{3}^{{}^{\circ}}$
100	2584.136	38686.08	*a* ^5^D_3_— $19_{4}^{{}^{\circ}}$
25	2585.340	38668.06	*b* ^3^F_4_— $25_{3}^{{}^{\circ}}$
30	2585.739	38662.10	*a* ^3^F_3_—*x* ${\;}^{5}P_{3}^{{}^{\circ}}$
10	2586.087	38656.89	*a* ^3^F_4_—*x* ${\;}^{1}F_{3}^{{}^{\circ}}$
5	2588.193	38625.44	*a* ^3^F_2_— $26_{1}^{{}^{\circ}}$
150	2589.569	38604.92	*a* ^3^F_2_— $25_{3}^{{}^{\circ}}$
10	2590.963	38584.15	*a* ^5^D_4_— $10_{4}^{{}^{\circ}}$
100	2591.116	38581.87	*a* ^5^D_3_— $17_{4}^{{}^{\circ}}$
40	2591.637	38574.11	*a* ^5^P_3_— $20_{2}^{{}^{\circ}}$
100	2592.022	38568.38	*a* ^5^P_3_**—***s* ${\;}^{3}D_{3}^{{}^{\circ}}$
10	2593.619	38544.64	*a* ^5^P_1_— $32_{2}^{{}^{\circ}}$
50	2593.700	38543.44	*a* ^3^G_4_— $61_{4}^{{}^{\circ}}$
100	2594.855	38526.28	*a* ^3^F_4_—*w* ${\;}^{5}D_{3}^{{}^{\circ}}$
5	2595.419	38517.91	*a* ^3^P_2_— $46_{3}^{{}^{\circ}}$
20	2595.640	38514.63	*b* ^3^F_4_— $23_{3}^{{}^{\circ}}$
10	2595.930	38510.33	*b* ^3^F_3_— $47_{4}^{{}^{\circ}}$
75	2597.322	38489.69	*a* ^5^D_2_—*s* ${\;}^{3}D_{2}^{{}^{\circ}}$
25	2597.517	38486.80	*b* ^3^F_3_— $46_{3}^{{}^{\circ}}$
1	2597.656	38484.74	*a* ^5^P_2_—*y* ${\;}^{1}P_{1}^{{}^{\circ}}$
25	2598.361	38474.30	*a* ^5^D_1_—*s* ${\;}^{3}D_{2}^{{}^{\circ}}$
20	2598.574	38471.15	$\{\;\begin{array}{l} a{\;}^{5}D_{3}—16_{3}^{{}^{\circ}}\\ b{\;}^{3}F_{2}—54_{3}^{{}^{\circ}} \end{array}$
1	2598.75	38468.54	*a* ^5^D_2_— $22_{3}^{{}^{\circ}}$
5	2600.719	38439.42	*a* ^3^P_0_— $57_{1}^{{}^{\circ}}$
2	2601.752	38424.16	*a* ^3^P_2_— $45_{1}^{{}^{\circ}}$
2	2602.977	38406.07	*a* ^3^P_1_— $57_{1}^{{}^{\circ}}$
30	2604.315	38386.34	*a* ^5^P_3_— $17_{4}^{{}^{\circ}}$
1	2604.560	38382.73	*b* ^3^F_3_— $44_{2}^{{}^{\circ}}$
100	2605.347	38371.14	*a* ^5^D_3_—*w* ${\;}^{3}F_{3}^{{}^{\circ}}$
100	2605.853	38363.69	*a* ^3^F_2_—*s* ${\;}^{3}D_{2}^{{}^{\circ}}$
2	2606.935	38347.77	*a* ^5^F_3_— $1_{4}^{{}^{\circ}}$
50	2607.348	38341.69	*a* ^5^F_3_*—y* ${\;}^{3}F_{2}^{{}^{\circ}}$
125	2609.062	38316.51	$\{\;\begin{array}{l} a{\;}^{3}F_{3}—13_{4}^{{}^{\circ}}\\ a{\;}^{5}D_{0}—26_{1}^{{}^{\circ}} \end{array}$
75	2609.476	38310.43	*a* ^3^P_2_— $43_{3}^{{}^{\circ}}$
20	2609.873	38304.60	*b* ^3^F_4_— $21_{5}^{{}^{\circ}}$
80	2611.045	38287.41	*a* ^5^D_2_— $20_{2}^{{}^{\circ}}$
8	2611.592	38279.39	*b* ^3^F_3_— $43_{3}^{{}^{\circ}}$
150	2612.06	38272.53	*a* ^5^D_4_—*w* ${\;}^{3}F_{4}^{{}^{\circ}}$
20	2612.895	38260.30	*a* ^5^F_4_—*y* ${\;}^{3}G_{5}^{{}^{\circ}}$
5	2613.198	38255.87	*a* ^3^F_4_—*x* ${\;}^{5}F_{3}^{{}^{\circ}}$
100	2614.055	38243.33	*a* ^5^F_5_—*y* ${\;}^{3}F_{4}^{{}^{\circ}}$
75	2614.585	38235.57	*a* ^5^F_2_—*z* ${\;}^{1}F_{3}^{{}^{\circ}}$
75	2615.093	38228.15	*a* ^5^D_3_—*x* ${\;}^{5}P_{2}^{{}^{\circ}}$
20	2617.677	38190.41	*a* ^5^D_2_*—w* ${\;}^{3}F_{2}^{{}^{\circ}}$
70	2617.790	38188.76	*a* ^5^P_1_— $26_{1}^{{}^{\circ}}$
20	2618.737	38174.96	*a* ^5^D_1_**—*w*** ${\;}^{3}F_{2}^{{}^{\circ}}$
70	2619.014	38170.92	*a* ^5^D_3_—*x* ${\;}^{5}P_{3}^{{}^{\circ}}$
50	2619.666	38161.42	*a* ^3^F_2_— $20_{2}^{{}^{\circ}}$
5	2620.062	38155.65	$\{\;\begin{array}{l} a{\;}^{3}F_{2}—s{\;}^{3}D_{3}^{{}^{\circ}}\\ a{\;}^{3}P_{2}—41_{2}^{{}^{\circ}} \end{array}$
80	2620.607	38147.72	*a* ^5^P_2_*—w* ${\;}^{5}D_{1}^{{}^{\circ}}$
15	2620.870	38143.89	*a* ^5^F_3_—*y* ${\;}^{3}G_{3}^{{}^{\circ}}$
10	2620.974	38142.37	*a* ^3^P_2_— $40_{3}^{{}^{\circ}}$
15	2621.071	38140.96	*b* ^3^F_4_— $19_{4}^{{}^{\circ}}$
1	2622.191	38124.67	*b* ^3^F_3_— $41_{2}^{{}^{\circ}}$
1	2623.611	38104.04	*a* ^5^D_1_— $18_{0}^{{}^{\circ}}$
75	2623.824	38100.95	*a* ^3^D_3_— $63_{3}^{{}^{\circ}}$
5	2625.081	38082.70	*a* ^5^F_4_—*z* ${\;}^{3}H_{4}^{{}^{\circ}}$
30	2626.205	38066.40	*a* ^5^D_4_— $7_{3}^{{}^{\circ}}$
20	2626.356	38064.22	*a* ^3^F_2_*—w* ${\;}^{3}F_{2}^{{}^{\circ}}$
50	2626.478	38062.45	*a* ^3^F_4_—*x* ${\;}^{5}F_{4}^{{}^{\circ}}$
100	2627.650	38045.47	*a* ^5^D_4_—*x* ${\;}^{1}G_{4}^{{}^{\circ}}$
25	2628.262	38036.62	*b* ^3^F_4_— $17_{4}^{{}^{\circ}}$
25	2628.536	38032.65	*a* ^5^P_3_—*x* ${\;}^{5}P_{2}^{{}^{\circ}}$
40	2629.925	38012.56	*a* ^5^P_2_— $9_{1}^{{}^{\circ}}$
160	2630.231	38008.14	*a* ^5^D_0_—*x* ${\;}^{5}P_{1}^{{}^{\circ}}$
100	2631.304	37992.65	*a* ^5^D_4_— $6_{3}^{{}^{\circ}}$
100	2631.569	37988.82	*a* ^5^D_2_— $16_{3}^{{}^{\circ}}$
75	2632.127	37980.77	*a* ^3^P_2_— $39_{1}^{{}^{\circ}}$
75	2632.496	37975.44	*a* ^5^P_3_—*x* ${\;}^{5}P_{3}^{{}^{\circ}}$
80	2633.446	37961.74	*b* ^3^F_2_— $49_{1}^{{}^{\circ}}$
20	2635.861	37926.97	*a* ^5^P_1_—*s* ${\;}^{3}D_{2}^{{}^{\circ}}$
75	2636.663	37915.43	*a* ^3^G_5_—*v* ${\;}^{3}G_{5}^{{}^{\circ}}$
3	2636.952	37911.27	*a* ^5^F_4_*—x* ${\;}^{3}D_{1}^{{}^{\circ}}$
1	2637.961	37896.77	*a* ^3^F_4_— $4_{3}^{{}^{\circ}}$
75	2638.515	37888.82	*a* ^5^D_2_—*w* ${\;}^{3}F_{3}^{{}^{\circ}}$
75	2639.121	37880.12	*a* ^5^P_1_*—x* ${\;}^{5}P_{1}^{{}^{\circ}}$
10	2639.866	37869.43	*a* ^3^P_2_— $35_{3}^{{}^{\circ}}$
60	2640.324	37862.86	*a* ^3^F_2_— $16_{3}^{{}^{\circ}}$
10	2641.461	37846.56	*a* ^5^F_4_—*z* ${\;}^{1}G_{4}^{{}^{\circ}}$
20	2641.970	37839.27	*a* ^3^F_3_*—t* ${\;}^{3}D_{2}^{{}^{\circ}}$
200	2642.946	37825.30	*a* ^5^D_3_— $13_{4}^{{}^{\circ}}$
75	2646.002	37781.62	*a* ^3^P_2_— $34_{3}^{{}^{\circ}}$
75	2647.314	37762.89	*a* ^3^F_2_—*w* ${\;}^{3}F_{3}^{{}^{\circ}}$
50	2648.451	37746.68	*a* ^5^P_2_—*w* ${\;}^{5}D_{2}^{{}^{\circ}}$
40	2648.782	37741.96	*a* ^3^G_5_—*v* ${\;}^{3}G_{4}^{{}^{\circ}}$
25	2649.506	37731.65	*a* ^5^D_2_— $15_{1}^{{}^{\circ}}$
20	2649.575	37730.67	*a* ^5^D_4_*—x* ${\;}^{5}P_{2}^{{}^{\circ}}$
10	2649.992	37724.73	*a* ^5^P_1_— $20_{2}^{{}^{\circ}}$
30	2650.395	37719.00	*a* ^5^D_4_—*x* ${\;}^{1}F_{3}^{{}^{\circ}}$
10	2650.584	37716.31	*a* ^5^D_4_— $15_{1}^{{}^{\circ}}$
40	2651.289	37706.28	*a* ^3^F_3_—*w* ${\;}^{5}D_{2}^{{}^{\circ}}$
20	2651.508	37703.16	*a* ^3^P_2_— $33_{2}^{{}^{\circ}}$
30	2651.839	37698.46	*a* ^3^F_4_—*w* ${\;}^{5}D_{4}^{{}^{\circ}}$
25	2651.874	37697.96	*a* ^5^D_3_— $12_{4}^{{}^{\circ}}$
5	2652.139	37694.19	*b* ^3^F_2_— $46_{3}^{{}^{\circ}}$
25	2653.693	37672.12	*b* ^3^F_3_— $33_{2}^{{}^{\circ}}$
5	2654.470	37661.10	*a* ^3^G_3_— $61_{4}^{{}^{\circ}}$
20	2654.804	37656.36	*a* ^3^P_0_— $49_{1}^{{}^{\circ}}$
30	2655.221	37650.44	*a* ^5^F_3_—*y* ${\;}^{3}P_{2}^{{}^{\circ}}$
75	2656.563	37631.42	*a* ^3^P_1_— $50_{2}^{{}^{\circ}}$
75	2656.698	37629.51	*a* ^5^P_3_— $13_{4}^{{}^{\circ}}$
7	2656.83	37627.64	*a* ^5^P_1_—*w* ${\;}^{3}F_{2}^{{}^{\circ}}$
75	2657.163	37622.93	*a* ^3^P_4_— $49_{1}^{{}^{\circ}}$
3	2657.366	37620.05	*a* ^3^F_2_—*x* ${\;}^{5}P_{2}^{{}^{\circ}}$
80	2658.391	37605.55	*a* ^3^F_2_— $15_{1}^{{}^{\circ}}$
10	2658.768	37600.22	*b* ^3^F_2_— $45_{1}^{{}^{\circ}}$
100	2659.617	37588.22	*a* ^5^D_4_—*w* ${\;}^{5}D_{3}^{{}^{\circ}}$
15	2660.601	37574.32	*b* ^3^F_4_— $14_{4}^{{}^{\circ}}$
50	2661.861	37556.53	*a* ^5^P_1_— $18_{0}^{{}^{\circ}}$
100	2664.761	37515.66	*a* ^5^F_4_—*y* ${\;}^{5}P_{3}^{{}^{\circ}}$
1	2665.154	37510.13	*b* ^3^F_3_— $32_{2}^{{}^{\circ}}$
3	2665.466	37505.74	*a* ^5^P_2_— $7_{3}^{{}^{\circ}}$
25	2665.719	37502.18	*a* ^5^P_3_— $12_{4}^{{}^{\circ}}$
20	2666.829	37486.57	*b* ^3^F_2_— $43_{3}^{{}^{\circ}}$
10	2667.484	37477.36	*a* ^3^H_5_— $62_{4}^{{}^{\circ}}$
50	2667.969	37470.56	*a* ^5^D_2_—*y* ${\;}^{1}P_{1}^{{}^{\circ}}$
40	2668.342	37465.32	*a* ^3^F_3_— $7_{3}^{{}^{\circ}}$
2	2670.708	37432.13	*a* ^5^P_2_— $6_{3}^{{}^{\circ}}$
80	2673.477	37393.36	*a* ^5^D_1_*—w* ${\;}^{5}D_{0}^{{}^{\circ}}$
80	2673.605	37391.57	*a* ^3^F_3_— $6_{3}^{{}^{\circ}}$
3	2674.471	37379.46	*a* ^3^P_2_— $29_{3}^{{}^{\circ}}$
3	2674.827	37374.49	*b* ^3^F_4_*—x* ${\;}^{1}H_{5}^{{}^{\circ}}$
1	2676.71	37348.20	*b* ^3^F_3_— $29_{3}^{{}^{\circ}}$
20	2676.969	37344.59	*a* ^3^F_2_—*y* ${\;}^{1}P_{1}^{{}^{\circ}}$
5	2677.881	37331.87	*b* ^3^F_2_— $41_{2}^{{}^{\circ}}$
10	2678.175	37327.77	*a* ^5^F_4_—*y* ${\;}^{3}F_{2}^{{}^{\circ}}$
50	2679.763	37305.65	*a* ^3^G_4_—*v* ${\;}^{3}G_{5}^{{}^{\circ}}$
1	2680.382	37297.04	*a* ^5^D_0_— $15_{1}^{{}^{\circ}}$
2	2680.533	37294.93	*a* ^3^P_0_— $45_{1}^{{}^{\circ}}$
60	2683.676	37251.26	*a* ^3^P_1_— $44_{2}^{{}^{\circ}}$
60	2684.089	37245.53	*a* ^1^D_2_— $63_{3}^{{}^{\circ}}$
10	2684.452	37240.49	*a* ^3^G_5_— $51_{4}^{{}^{\circ}}$
175	2686.291	37215.00	*a* ^5^D_3_—*w* ${\;}^{5}D_{2}^{{}^{\circ}}$
40	2687.138	37203.27	*a* ^5^F_2_—*y* ${\;}^{3}P_{1}^{{}^{\circ}}$
1	2687.755	37194.73	*a* ^3^H_4_— $63_{3}^{{}^{\circ}}$
50	2688.583	37183.28	*a* ^5^P_1_—*x* ${\;}^{5}P_{2}^{{}^{\circ}}$
70	2688.888	37179.06	*a* ^5^D_4_—*y* ${\;}^{3}H_{4}^{{}^{\circ}}$
70	2689.894	37165.15	*a* ^3^P_2_— $25_{3}^{{}^{\circ}}$
40	2690.382	37158.41	*a* ^5^P_2_*—x* ${\;}^{1}F_{3}^{{}^{\circ}}$
2	2690.725	37153.67	*a* ^5^P_2_*—y* ${\;}^{5}S_{2}^{{}^{\circ}}$
25	2690.810	37152.50	$\{\;\begin{array}{l} b{\;}^{3}F_{4}—12_{4}^{{}^{\circ}}\\ a{\;}^{5}P_{3}—t{\;}^{3}D_{2}^{{}^{\circ}} \end{array}$
50	2692.251	37132.62	*a* ^3^G_4_—*v* ${\;}^{3}G_{4}^{{}^{\circ}}$
1	2692.716	37126.21	*b* ^3^P_2_— $60_{1}^{{}^{\circ}}$
80	2692.842	37124.47	*a* ^5^D_4_*—x* ${\;}^{5}F_{4}^{{}^{\circ}}$
150	2693.30	37118.16	*a* ^5^D_1_—*w* ${\;}^{5}D_{1}^{{}^{\circ}}$
30	2693.653	37113.29	*a* ^3^F_3_—*y* ${\;}^{5}S_{2}^{{}^{\circ}}$
50	2697.510	37060.23	*a* ^5^F_2_*—y* ${\;}^{5}P_{1}^{{}^{\circ}}$
15	2698.050	37052.81	*a* ^5^F_4_—*y* ${\;}^{3}F_{4}^{{}^{\circ}}$
10	2699.712	37030.00	*a* ^5^D_4_— $11_{0}^{{}^{\circ}}$
20	2699.793	37028.89	*a* ^5^F_2_—*y* ${\;}^{3}P_{2}^{{}^{\circ}}$
40	2699.882	37027.67	*a* ^5^P_2_—*w* ${\;}^{5}D_{3}^{{}^{\circ}}$
30	2700.477	37019.51	*a* ^5^P_3_—*w* ${\;}^{5}D_{2}^{{}^{\circ}}$
25	2700.671	37016.85	*a* ^5^D_3_—*x* ${\;}^{5}F_{2}^{{}^{\circ}}$
70	2701.337	37007.73	*a* ^3^F_2_—*w* ${\;}^{5}D_{1}^{{}^{\circ}}$
5	2701.997	36998.69	*a* ^5^D_2_— $9_{1}^{{}^{\circ}}$
4	2702.401	36993.16	*a* ^3^P_1_— $41_{2}^{{}^{\circ}}$
80	2702.833	36987.25	*a* ^3^F_3_—*w* ${\;}^{5}D_{3}^{{}^{\circ}}$
4	2703.120	36983.32	*a* ^5^D_1_— $9_{1}^{{}^{\circ}}$
4	2703.311	36980.71	*b* ^3^F_3_— $23_{3}^{{}^{\circ}}$
50	2703.796	36974.07	*a* ^5^D_3_— $7_{3}^{{}^{\circ}}$
5	2704.971	36958.01	$\{\;\begin{array}{l} a{\;}^{3}G_{5}—47_{4}^{{}^{\circ}}\\ b{\;}^{3}F_{2}—34_{3}^{{}^{\circ}} \end{array}$
10	2705.323	36953.20	*a* ^5^D_3_—*x* ${\;}^{1}G_{4}^{{}^{\circ}}$
70	2707.477	36923.81	*a* ^3^P_2_—*s* ${\;}^{3}D_{2}^{{}^{\circ}}$
100	2707.969	36917.10	*a* ^6^F_3_*—y* ${\;}^{5}P_{2}^{{}^{\circ}}$
25	2708.635	36908.02	*a* ^5^P_1_—*y* ${\;}^{1}P_{1}^{{}^{\circ}}$
25	2708.841	36905.21	$\{\;\begin{array}{l} a{\;}^{5}D_{1}—8_{1}^{{}^{\circ}}\\ a{\;}^{3}G_{4}—52_{4}^{{}^{\circ}} \end{array}$
5	2709.045	36902.44	*a* ^3^P_2_— $22_{3}^{{}^{\circ}}$
125	2709.198	36900.35	*a* ^5^D_3_— $6_{3}^{{}^{\circ}}$
5	2709.755	36892.77	*b* ^3^F_3_—*s* ${\;}^{3}D_{2}^{{}^{\circ}}$
10	2710.732	36879.47	$\{\;\begin{array}{l} b{\;}^{3}F_{2}—33_{2}^{{}^{\circ}}\\ a{\;}^{5}P_{1}—t{\;}^{3}D_{1}^{{}^{\circ}} \end{array}$
2	2711.313.	36871.57	*b* ^3^F_3_— $22_{3}^{{}^{\circ}}$
10	2712.868	36850.44	*a* ^5^D_1_*—t* ${\;}^{3}D_{2}^{{}^{\circ}}$
75	2713.192	36846.04	*a* ^5^P_1_*—w* ${\;}^{5}D_{0}^{{}^{\circ}}$
50	2713.728	36838.76	*a* ^5^D_4_—*x* ${\;}^{5}F_{5}^{{}^{\circ}}$
1	2714.998	36821.53	*a* ^5^P_3_*—x* ${\;}^{5}F_{2}^{{}^{\circ}}$
40	2715.233	36818.34	*a* ^3^P_1_— $39_{1}^{{}^{\circ}}$
75	2715.773	36811.02	*a* ^5^F_1_*—y* ${\;}^{3}P_{1}^{{}^{\circ}}$
30	2717.001	36794.39	*a* ^3^F_2_— $8_{1}^{{}^{\circ}}$
100	2717.401	36788.97	*a* ^5^F_1_—*y* ${\;}^{3}P_{0}^{{}^{\circ}}$
1*h*	2718.168	36778.59	*a* ^5^P_3_— $7_{3}^{{}^{\circ}}$
250	2719.51	36760.44	*a* ^5^D_4_*—w* ${\;}^{5}D_{4}^{{}^{\circ}}$
70	2721.562	36732.73	*a* ^5^D_2_—*w* ${\;}^{5}D_{2}^{{}^{\circ}}$
15	2722.386	36721.61	*a* ^3^P_2_— $20_{2}^{{}^{\circ}}$
50	2722.693	36717.47	$\{\;\begin{array}{l} a{\;}^{5}D_{1}—w{\;}^{5}D_{2}^{{}^{\circ}}\\ b{\;}^{3}F_{2}—32_{2}^{{}^{\circ}} \end{array}$
10	2722.82	36715.75	*a* ^3^P_2_—*s* ${\;}^{3}D_{3}^{{}^{\circ}}$
100	2724.066	36698.96	*a* ^5^D_0_*—w* ${\;}^{5}D_{1}^{{}^{\circ}}$
1	2725.13	36684.63	*b* ^3^F_3_—*s* ${\;}^{3}D_{3}^{{}^{\circ}}$
2	2726.360	36668.08	*a* ^5^F_1_*—y* ${\;}^{5}P_{1}^{{}^{\circ}}$
50	2726.969	36659.90	*a* ^1^G_4_— $61_{4}^{{}^{\circ}}$
1*h*	2727.505	36652.69	*a* ^3^G_3_— $59_{3}^{{}^{\circ}}$
2	2727.953	36646.67	*a* ^3^G_5_— $42_{4}^{{}^{\circ}}$
80	2728.836	36634.81	*b* ^3^F_4_—*w* ${\;}^{3}F_{4}^{{}^{\circ}}$
2	2729.129	36630.88	*a* ^3^G_4_— $51_{4}^{{}^{\circ}}$
60	2729.455	36626.51	*a* ^5^D_3_—*x* ${\;}^{1}F_{3}^{{}^{\circ}}$
1	2729.790	36622.01	*a* ^5^D_3_—*y* ${\;}^{5}S_{2}^{{}^{\circ}}$
80	2730.325	36614.84	*a* ^5^F_3_*—y* ${\;}^{5}P_{3}^{{}^{\circ}}$
100	2730.932	36606.70	*a* ^3^F_2_—*w* ${\;}^{5}D_{2}^{{}^{\circ}}$
30	2731.903	36593.69	*a* ^5^P_3_—*y* ${\;}^{1}G_{4}^{{}^{\circ}}$
10	2733.086	36577.85	*a* ^3^F_3_—*y* ${\;}^{3}H_{4}^{{}^{\circ}}$
125	2733.578	36571.27	*a* ^5^P_1_—*w* ${\;}^{5}D_{1}^{{}^{\circ}}$
1	2734.15	36563.62	*a* ^5^D_0_— $9_{1}^{{}^{\circ}}$
250	2735.727	36542.54	*a* ^5^F_5_—*x* ${\;}^{5}D_{4}^{{}^{\circ}}$
10	2736.315	36534.69	*a* ^5^D_2_—*x* ${\;}^{5}F_{2}^{{}^{\circ}}$
1	2737.145	26523.61	*a* ^3^F_3_—*x* ${\;}^{5}F_{4}^{{}^{\circ}}$
20	2737.463	36519.37	*a* ^5^D_1_—*x* ${\;}^{5}F_{2}^{{}^{\circ}}$
150	2739.217	36495.98	*a* ^5^D_3_—*w* ${\;}^{5}D_{3}^{{}^{\circ}}$
5	2739.454	36492.83	*a* ^5^D_4_—*x* ${\;}^{3}G_{3}^{{}^{\circ}}$
1*h*	2739.986	36485.74	*a* ^5^D_0_— $8_{1}^{{}^{\circ}}$
50	2740.217	36482.67	*a* ^5^P_1_— $11_{0}^{{}^{\circ}}$
80	2743.934	36433.25	*a* ^3^F_4_*—z* ${\;}^{3}I_{5}^{{}^{\circ}}$
125	2744.448	36426.43	*a* ^5^P_3_—*y* ${\;}^{5}S_{2}^{{}^{\circ}}$
3*h*	2744.705	36423.01	*a* ^3^P_2_— $16_{3}^{{}^{\circ}}$
75	2745.074	36418.12	*a* ^5^D_2_— $6_{3}^{{}^{\circ}}$
15	2745.827	36408.13	*b* ^3^F_4_—*x* ${\;}^{1}G_{4}^{{}^{\circ}}$
75	2746.885	36394.11	*a* ^3^F_4_—*x* ${\;}^{3}G_{4}^{{}^{\circ}}$
1	2747.163	36390.43	*a* ^3^G_5_— $38_{4}^{{}^{\circ}}$
40	2747.963	36379.83	*a* ^5^D_4_—*w* ${\;}^{3}G_{4}^{{}^{\circ}}$
40	2748.045	36378.75	*a* ^3^P_4_— $32_{2}^{{}^{\circ}}$
10	2749.014	36365.93	*a* ^3^F_2_— $7_{3}^{{}^{\circ}}$
2	2749.314	36361.96	*b* ^3^F_2_— $26_{1}^{{}^{\circ}}$
5*h*	2749.64	36357.65	$\{\;\begin{array}{l} a{\;}^{3}F_{3}—4_{3}^{{}^{\circ}}\\ a{\;}^{5}P_{1}—8_{1}^{{}^{\circ}} \end{array}$
3	2749.824	36355.21	*b* ^3^F_4_— $6_{3}^{{}^{\circ}}$
50	2750.345	36348.33	*a* ^3^G_4_— $47_{4}^{{}^{\circ}}$
2	2750.864	36341.47	*b* ^3^F_2_— $25_{3}^{{}^{\circ}}$
60	2752.262	36323.01	*a* ^3^P_2_—*w* ${\;}^{3}F_{3}^{{}^{\circ}}$
1	2753.761	36303.24	*a* ^5^P_1_—*t* ${\;}^{3}D_{2}^{{}^{\circ}}$
10	2753.968	36300.51	*a* ^5^P_3_—*w* ${\;}^{5}D_{3}^{{}^{\circ}}$
10	2754.358	36295.37	*a* ^5^F_2_—*y* ${\;}^{5}P_{2}^{{}^{\circ}}$
60	2754.603	36292.14	*a* ^3^F_2_— $6_{3}^{{}^{\circ}}$
1	2756.181	36271.37	*a* ^3^G_3_— $55_{2}^{{}^{\circ}}$
30	2757.064	36259.75	*a* ^5^D_4_—*w* ${\;}^{3}G_{5}^{{}^{\circ}}$
80	2757.798	36250.10	*a* ^3^G_3_—*v* ${\;}^{3}G_{4}^{{}^{\circ}}$
2	2758.238	36244.32	*b* ^3^F_4_—*y* ${\;}^{1}G_{4}^{{}^{\circ}}$
100	2759.682	36225.35	*a* ^5^D_3_—*x* ${\;}^{5}F_{3}^{{}^{\circ}}$
30	2760.155	36219.15	*a* ^3^G_3_— $54_{3}^{{}^{\circ}}$
100	2762.304	36190.97	*a* ^5^P_2_—*x* ${\;}^{3}P_{2}^{{}^{\circ}}$
60	2763.133	36180.12	*a* ^3^P_2_—*x* ${\;}^{5}P_{2}^{{}^{\circ}}$
200	2763.413	36176.45	*a* ^5^F_4_—*x* ${\;}^{5}D_{3}^{{}^{\circ}}$
50	2763.900	36170.07	*a* ^5^P_1_—*w* ${\;}^{5}D_{2}^{{}^{\circ}}$
10	2764.229	36165.77	*a* ^3^P_2_— $15_{1}^{{}^{\circ}}$
70	2764.714	36159.43	*a* ^3^F_3_—*w* ${\;}^{5}D_{4}^{{}^{\circ}}$
5	2765.521	36148.87	*b* ^3^F_3_—*x* ${\;}^{5}P_{2}^{{}^{\circ}}$
1	2765.853	36144.54	*a* ^5^D_2_—*x* ${\;}^{1}F_{3}^{{}^{\circ}}$
50	2766.223	36139.70	*a* ^5^D_2_—*y* ${\;}^{5}S_{2}^{{}^{\circ}}$
1	2767.389	36124.48	*a* ^5^D_1_—*y* ${\;}^{5}S_{2}^{{}^{\circ}}$
50*w*	2767.516	36122.82	*a* ^3^P_2_—*x* ${\;}^{5}P_{3}^{{}^{\circ}}$
1	2767.689	36120.56	*a* ^5^P_3_— $55_{2}^{{}^{\circ}}$
1	2767.938	36117.31	*a* ^3^G_4_— $43_{3}^{{}^{\circ}}$
1*h*	2769.893	36091.82	*b* ^3^F_3_—*x* ${\;}^{5}P_{3}^{{}^{\circ}}$
50	2770.296	36086.57	*a* ^5^D_3_—*y* ${\;}^{3}H_{4}^{{}^{\circ}}$
100	2770.698	36081.33	*b* ^3^F_4_—*x* ${\;}^{1}F_{3}^{{}^{\circ}}$
100	2772.608	36056.48	*a* ^3^P_0_— $26_{1}^{{}^{\circ}}$
1*h*	2774.101	36037.08	*a* ^3^G_4_— $42_{4}^{{}^{\circ}}$
125	2774.480	36032.15	*a* ^5^D_3_—*x* ${\;}^{5}F_{4}^{{}^{\circ}}$
70	2775.175	36023.13	$\{\;\begin{array}{c} a{\;}^{3}G_{3}—52_{4}^{{}^{\circ}}\\ a{\;}^{3}P_{1}—26_{1}^{{}^{\circ}} \end{array}$
2*h*	2775.540	36018.39	*a* ^3^F_2_—*x* ${\;}^{1}F_{3}^{{}^{\circ}}$
80	2775.902	36013.70	*a* ^5^D_2_—*w* ${\;}^{3}D_{3}^{{}^{\circ}}$
		36013.71	*a* ^3^F_2_—*y* ${\;}^{5}S_{2}^{{}^{\circ}}$
5	2777.480	35993.24	*a* ^5^F_2_—*y* ${\;}^{5}P_{3}^{{}^{\circ}}$
70	2780.759	35950.80	*b* ^3^F_4_—*w* ${\;}^{5}D_{3}^{{}^{\circ}}$
5	2781.831	35936.94	*a* ^3^G_5_— $31_{4}^{{}^{\circ}}$
80	2782.205	35932.11	*a* ^5^P_2_—*x* ${\;}^{3}G_{3}^{{}^{\circ}}$
3	2784.320	35904.82	*a* ^3^P_2_—*y* ${\;}^{1}P_{1}^{{}^{\circ}}$
30	2784.516	35902.29	*a* ^3^G_5_— $30_{4}^{{}^{\circ}}$
2	2784.864	35897.81	*b* ^3^F_2_— $20_{2}^{{}^{\circ}}$
75	2785.334	35891.75	*a* ^3^F_3_—*x* ${\;}^{3}G_{3}^{{}^{\circ}}$
80	2785.649	35887.69	*a* ^3^F_2_—*w* ${\;}^{5}D_{3}^{{}^{\circ}}$
5	2786.710	35874.03	*a* ^5^F_2_—*z* ${\;}^{3}S_{1}^{{}^{\circ}}$
100	2787.823	35859.71	*a* ^5^P_2_—*y* ${\;}^{1}D_{3}^{{}^{\circ}}$
2	2789.615	35836.67	*a* ^5^P_3_—*x* ${\;}^{5}F_{4}^{{}^{\circ}}$
1	2789.833	35833.87	*a* ^5^D_2_— $5_{2}^{{}^{\circ}}$
6	2791.036	35818.43	*a* ^3^D_4_— $5_{2}^{{}^{\circ}}$
75	2792.641	35797.84	*a* ^3^P_2_—*u* ${\;}^{3}D_{1}^{{}^{\circ}}$
20	2794.678	35771.75	*b* ^3^P_2_— $50_{2}^{{}^{\circ}}$
30	2795.508	35761.13	*a*_3_P_1_—*s* ${\;}^{3}D_{2}^{{}^{\circ}}$
1	2796.049	35754.21	*a* ^3^G_4_— $37_{3}^{{}^{\circ}}$
20	2796.543	35747.90	*a* ^3^P_0_—*x* ${\;}^{5}P_{1}^{{}^{\circ}}$
40	2796.697	35745.93	*b* ^3^F^3^— $13_{4}^{{}^{\circ}}$
1	2796.904	35743.28	*a* ^5^D_2_—*x* ${\;}^{5}F_{3}^{{}^{\circ}}$
1	2798.872	35718.15	*a* ^3^D_2_— $60_{1}^{{}^{\circ}}$
10	2801.856	35680.11	*b* ^3^F_4_—*x* ${\;}^{5}F_{3}^{{}^{\circ}}$
125	2802.805	35668.03	*a* ^5^D_3_—*w* ${\;}^{5}D_{4}^{{}^{\circ}}$
40	2803.496	35659.24	*a* ^5^D_3_—*x* ${\;}^{3}P_{2}^{{}^{\circ}}$
50	2808.221	35599.25	*b* ^3^F_2_— $16_{3}^{{}^{\circ}}$
150	2810.029	35576.34	*a* ^5^F_3_—*x* ${\;}^{5}D_{2}^{{}^{\circ}}$
250	2810.551	35569.74	*a* ^5^F_4_—*y* ${\;}^{3}D_{3}^{{}^{\circ}}$
20	2810.695	35567.92	*a* ^3^P_2_—*w* ${\;}^{5}D_{1}^{{}^{\circ}}$
25	2814.862	35515.26	*a* ^5^D_4_—*y* ${\;}^{1}F_{3}^{{}^{\circ}}$
1	2816.116	35499.45	*b* ^3^F_2_—*w* ${\;}^{3}F_{3}^{{}^{\circ}}$
100	2817.092	35487.15	*a* ^5^F_2_—*x* ${\;}^{5}F_{1}^{{}^{\circ}}$
200	2818.359	35471.20	*a* ^5^F_5_—*y* ${\;}^{5}F_{4}^{{}^{\circ}}$
40	2818.809	35465.53	*a* ^5^P_2_—*u* ${\;}^{3}D_{2}^{{}^{\circ}}$
75	2818.950	35463.76	*a* ^5^P_3_—*x* ${\;}^{3}P_{2}^{{}^{\circ}}$
5	2819.562	35456.06	*a* ^5^D_4_—*x* ${\;}^{3}G_{4}^{{}^{\circ}}$
1	2820.667	35442.17	*a* ^3^G_3_— $46_{3}^{{}^{\circ}}$
30	2821.171	35435.84	*b* ^3^P_4_— $50_{2}^{{}^{\circ}}$
3	2821.414	35432.79	*a* ^3^P_2_— $9_{1}^{{}^{\circ}}$
100	2822.034	35425.01	*a* ^3^F_3_—*u* ${\;}^{3}D_{2}^{{}^{\circ}}$
1	2822.256	35422.22	*a* ^1^G_4_—*v* ${\;}^{3}G_{5}^{{}^{\circ}}$
2	2823.878	35401.88	*b* ^3^P_2_— $45_{1}^{{}^{\circ}}$
10	2824.756	35390.87	*a* ^3^P_1_— $18_{0}^{{}^{\circ}}$
5	2827.518	35356.30	*b* ^3^F_2_—*x* ${\;}^{5}P_{2}^{{}^{\circ}}$
100	2827.857	35352.07	*a* ^5^F_4_—*x* ${\;}^{5}D_{4}^{{}^{\circ}}$
200	2829.149	35335.92	*a* ^3^F_4_—*v* ${\;}^{3}D_{3}^{{}^{\circ}}$
1	2832.044	35299.80	*a* ^3^P_2_—*t* ${\;}^{3}D_{2}^{{}^{\circ}}$
40	2832.624	35292.57	*a* ^3^G_4_— $30_{4}^{{}^{\circ}}$
20	2833.05	35287.27	*a* ^5^D_3_—*w* ${\;}^{3}G_{4}^{{}^{\circ}}$
80	2833.999	35275.45	*a* ^5^F_3_—*x* ${\;}^{5}D_{3}^{{}^{\circ}}$
2	2834.347	35271.12	*a* ^5^P_1_— $5_{2}^{{}^{\circ}}$
2	2835.415	35257.84	*a* ^3^F_2_— $4_{3}^{{}^{\circ}}$
50	2836.143	35248.79	*a* ^5^G_4_—*v* ${\;}^{3}G_{4}^{{}^{\circ}}$
70	2836.569	35243.49	*a* ^5^D_2_— $3_{1}^{{}^{\circ}}$
20	2837.269	35234.80	*a* ^3^G_3_— $43_{3}^{{}^{\circ}}$
3	2837.814	35228.03	*a* ^5^D_1_— $3_{1}^{{}^{\circ}}$
25	2838.615	35218.09	$\{\;\begin{array}{c} a{\;}^{3}G_{5}—21_{5}^{{}^{\circ}}\\ a{\;}^{1}G_{4}—54_{3}^{{}^{\circ}} \end{array}$
1	2839.677	35204.92	*a* ^5^P_3_—*x* ${\;}^{3}G_{3}^{{}^{\circ}}$
80	2840.537	35194.26	*a* ^3^F_4_—*x* ${\;}^{3}G_{5}^{{}^{\circ}}$
2	2841.173	35186.39	*a* ^3^G_4_— $29_{3}^{{}^{\circ}}$
2*h*	2841.928	35177.04	*a* ^5^D_2_—*x* ${\;}^{3}P_{2}^{{}^{\circ}}$
20	2842.527	35169.63	*a* ^3^D_3_— $61_{4}^{{}^{\circ}}$
20	2842.749	35166.88	*a* ^3^P_2_—*w* ${\;}^{5}D_{2}^{{}^{\circ}}$
70	2843.170	35161.67	*a* ^5^D_1_—*x* ${\;}^{3}P_{2}^{{}^{\circ}}$
5	2843.740	35154.62	*a* ^3^G_3_— $42_{4}^{{}^{\circ}}$
25	2845.537	35132.43	*a* ^5^P_3_—*y* ${\;}^{1}D_{2}^{{}^{\circ}}$
60	2846.318	35122.79	*b* ^3^F_4_—*w* ${\;}^{5}D_{4}^{{}^{\circ}}$
20	2846.537	35120.08	$\{\;\begin{array}{c} a{\;}^{3}H_{5}—61_{4}^{{}^{\circ}}\\ b{\;}^{3}P_{2}—40_{3}^{{}^{\circ}} \end{array}$
30	2846.750	35117.46	*a* ^3^F_2_— $3_{1}^{{}^{\circ}}$
80	2848.586	35094.82	*a* ^5^F_1_—*x* ${\;}^{5}D_{1}^{{}^{\circ}}$
3	2848.812	35092.04	*a* ^5^P_3_—*w* ${\;}^{3}G_{4}^{{}^{\circ}}$
1	2849.775	35080.18	*a* ^3^G_3_— $41_{2}^{{}^{\circ}}$
2	2850.864	35066.78	*a* ^3^G_3_— $40_{3}^{{}^{\circ}}$
15	2851.104	35063.83	*a* ^5^P_2_—*y* ${\;}^{3}S_{1}^{{}^{\circ}}$
10	2851.866	35054.46	*a* ^3^G_5_— $19_{4}^{{}^{\circ}}$
200	2854.075	35027.33	*a* ^5^F_3_—*z* ${\;}^{3}P_{2}^{{}^{\circ}}$
1	2854.528	35021.77	*a* ^1^G_4_— $52_{4}^{{}^{\circ}}$
3	2854.870	35017.58	*a* ^3^P_1_—*x* ${\;}^{5}F_{2}^{{}^{\circ}}$
30	2856.044	35003.18	*a* ^3^P_1_— $15_{1}^{{}^{\circ}}$
1	2857.240	34988.53	*b* ^3^F_4_— *y* ${\;}^{3}H_{5}^{{}^{\circ}}$
2	2858.591	34972.00	*a* ^3^G_4_— $25_{3}^{{}^{\circ}}$
1	2858.849	34968.84	*a* ^3^P_2_—*x* ${\;}^{5}F_{2}^{{}^{\circ}}$
100	2860.014	34954.60	$\{\;\begin{array}{c} a{\;}^{5}F_{2}—x{\;}^{5}D_{2}^{{}^{\circ}}\\ a{\;}^{5}P_{2}—y{\;}^{1}F_{3}^{{}^{\circ}} \end{array}$
20	2860.369	34950.26	*a* ^8^G_5_— $17_{4}^{{}^{\circ}}$
125	2861.408	34937.57	$\{\;\begin{array}{c} a{\;}^{3}F_{4}—w{\;}^{3}D_{3}^{{}^{\circ}}\\ b{\;}^{3}F_{3}—x{\;}^{5}F_{2}^{{}^{\circ}} \end{array}$
40	2861.718	34933.78	*a* ^5^D_3_—*u* ${\;}^{3}D_{2}^{{}^{\circ}}$
1	2862.358	34925.97	*a* ^3^P_2_— $7_{3}^{{}^{\circ}}$
30	2863.003	34918.11	*a* ^5^D_2_—*x* ${\;}^{3}G_{3}^{{}^{\circ}}$
20	2863.324	34914.19	*a* ^3^F_3_—*y* ${\;}^{1}F_{3}^{{}^{\circ}}$
10	2863.975	34906.26	*a* ^5^F_2_—*y* ${\;}^{3}D_{1}^{{}^{\circ}}$
1	2864.618	34898.42	*a* ^3^G_3_— $38_{4}^{{}^{\circ}}$
150	2866.653	34873.65	*a* ^5^F_3_—-*y* ${\;}^{3}D_{2}^{{}^{\circ}}$
25	2867.465	34863.77	*a* ^5^D_4_—*x* ${\;}^{3}F_{4}^{{}^{\circ}}$
25	2868.183	34855.05	*a* ^3^F_3_—*x* ^3^G_4_
30	2868.310	34853.50	*a* ^5^P_2_—*v* ^3^D_2_
8	2868.412	34852.26	*a* ^3^P_2_— $6_{3}^{{}^{\circ}}$
15	2868.544	34850.66	*a* ^5^P_2_—*w* ${\;}^{3}D_{1}^{{}^{\circ}}$
2	2868.945	34845.79	*a* ^5^D_2_—*y* ${\;}^{1}D_{2}^{{}^{\circ}}$
40	2870.213	34830.40	*a* ^5^D_1_—*y* ${\;}^{1}D_{2}^{{}^{\circ}}$
40	2871.186	34818.59	*a* ^3^G_4_— $23_{3}^{{}^{\circ}}$
100	2871.642	34813.07	*a* ^3^F_3_—*v* ${\;}^{3}D_{2}^{{}^{\circ}}$
50	2873.370	34792.13	*a* ^3^F_2_—*x* ${\;}^{3}G_{3}^{{}^{\circ}}$
25	2874.050	34783.90	*a* ^5^D_2_—*u* ${\;}^{3}D_{1}^{{}^{\circ}}$
1000*R*	2874.984	34772.60	*a* ^6^F_5_—*y* ${\;}^{5}F_{5}^{{}^{\circ}}$
10	2875.316	34768.59	*a* ^5^D_1_—*u* ${\;}^{3}D_{1}^{{}^{\circ}}$
40	2877.092	34747.12	*a* ^3^P_0_—*t* ${\;}^{3}D_{1}^{{}^{\circ}}$
1	2877.339	34744.14	*b* ^3^F_2_—*w* ${\;}^{5}D_{1}^{{}^{\circ}}$
1	2877.489	34742.33	*b* ^3^F_4_—*w* ${\;}^{3}G_{4}^{{}^{\circ}}$
50	2877.826	34738.26	*a* ^5^P_3_—*u* ${\;}^{3}D_{2}^{{}^{\circ}}$
25	2879.358	34719.78	*a* ^3^F_2_—*y* ${\;}^{1}D_{2}^{{}^{\circ}}$
100	2879.756	34714.98	*a* ^3^F_4_—*x* ${\;}^{3}F_{3}^{{}^{\circ}}$
10	2880.227	34709.30	*a* ^3^G_4_— $22_{3}^{{}^{\circ}}$
2	2880.506	34705.94	*a* ^3^G_3_— $34_{3}^{{}^{\circ}}$
60	2881.273	34696.70	*a* ^5^F_4_—*x* ${\;}^{5}D_{0}^{{}^{\circ}}$
15	2882.577	34681.01	$\{\;\begin{array}{c} b{\;}^{3}P_{2}—33_{2}^{{}^{\circ}}\\ a{\;}^{5}P_{1}—3_{1}^{{}^{\circ}} \end{array}$
5	2882.622	34680.47	*a* ^3^P_1_—*w* ${\;}^{5}D_{0}^{{}^{\circ}}$
75	2883.594	34668.78	*a* ^5^F_3_—*y* ${\;}^{3}D_{3}^{{}^{\circ}}$
50	2884.500	34657.89	*a* ^3^F_2_—*u* ${\;}^{3}D_{1}^{{}^{\circ}}$
80	2884.843	34653.77	*a* ^5^F_2_—*x* ${\;}^{5}D_{3}^{{}^{\circ}}$
150	2886.528	34633.54	*a* ^5^F_2_—*z* ${\;}^{3}P_{1}^{{}^{\circ}}$
70	2887.993	34615.97	*a* ^5^F_4_—*y* ${\;}^{5}F_{3}^{{}^{\circ}}$
50	2888.624	34608.41	*a* ^3^G_4_— $21_{5}^{{}^{\circ}}$
1*h*	2889.047	34603.34	*a* ^1^G_4_— $48_{3}^{{}^{\circ}}$
10	2890.879	34581.42	*b* ^3^P_0_— $49_{1}^{{}^{\circ}}$
50	2891.130	34578.41	*a* ^3^P_2_—*x* ${\;}^{1}F_{3}^{{}^{\circ}}$
60	2891.645	34572.26	*a* ^3^H_6_—*v* ${\;}^{3}G_{5}^{{}^{\circ}}$
10	2892.470	34562.39	*a* ^5^F_1_—*x* ${\;}^{5}D_{2}^{{}^{\circ}}$
20	2893.731	34547.34	*b* ^3^F_3_—*x* ${\;}^{1}F_{3}^{{}^{\circ}}$
15	2895.802	34522.63	*a* ^3^G_4_—*s* ${\;}^{3}D_{3}^{{}^{\circ}}$
100	2896.523	34514.04	*a* ^5^F_1_—*y* ${\;}^{3}D_{1}^{{}^{\circ}}$
50	2898.533	34490.10	*a* ^5^P_2_—*w* ${\;}^{3}D_{2}^{{}^{\circ}}$
3	2898.715	34487.94	*a* ^3^G_5_— $14_{4}^{{}^{\circ}}$
50	2899.716	34476.04	$\{\;\begin{array}{c} b{\;}^{3}F_{2}—t{\;}^{3}D_{2}^{{}^{\circ}}\\ b{\;}^{3}F_{4}—y{\;}^{1}H_{5}^{{}^{\circ}} \end{array}$
1	2900.669	34464.71	*a* ^1^G_4_— $47_{4}^{{}^{\circ}}$
25	2901.784	34451.47	*a* ^5^D_2_—*u* ${\;}^{3}D_{2}^{{}^{\circ}}$
50	2901.937	34449.65	*a* ^3^F_3_—*w* ${\;}^{3}D_{2}^{{}^{\circ}}$
50	2902.087	34447.87	*a* ^3^P_2_—*w* ${\;}^{5}D_{3}^{{}^{\circ}}$
25	2902.854	34438.77	*a* ^3^P_0_—*w* ${\;}^{5}D_{1}^{{}^{\circ}}$
40	2903.074	34436.16	*a* ^5^D_1_—*u* ${\;}^{3}D_{2}^{{}^{\circ}}$
5	2904.191	34422.91	*a* ^5^D_3_—*y* ${\;}^{1}F_{3}^{{}^{\circ}}$
10	2904.704	34416.83	*b* ^3^F_3_—*w* ${\;}^{5}D_{3}^{{}^{\circ}}$
75	2905.651	34405.61	*a* ^5^F_2_—*z* ${\;}^{3}P_{2}^{{}^{\circ}}$
50	2905.822	34403.59	*a* ^3^F_4_—*z* ${\;}^{1}F_{3}^{{}^{\circ}}$
60	2906.315	34397.76	*a* ^3^D_4_—*v* ${\;}^{3}D_{3}^{{}^{\circ}}$
2	2908.481	34372.14	*a* ^5^P_2_—*z* ${\;}^{1}P_{1}^{{}^{\circ}}$
150	2908.883	34367.39	*a* ^5^F_1_—*z* ${\;}^{3}P_{0}^{{}^{\circ}}$
30	2909.212	34363.50	$\{\;\begin{array}{c} a{\;}^{5}D_{3}—x{\;}^{3}G_{4}^{{}^{\circ}}\\ a{\;}^{3}D_{2}—50_{2}^{{}^{\circ}} \end{array}$
5	2909.940	34354.91	*a* ^3^D_2_— $49_{1}^{{}^{\circ}}$
20	2910.425	34349.18	*a* ^5^D_0_—*u* ${\;}^{3}D_{1}^{{}^{\circ}}$
10	2910.772	34345.09	*b* ^3^P_4_— $33_{2}^{{}^{\circ}}$
5	2910.936	34343.15	*b* ^3^F_2_—*w* ${\;}^{5}D_{2}^{{}^{\circ}}$
2	2911.148	34340.65	*a* ^3^G_4_— $17_{4}^{{}^{\circ}}$
50	2912.433	34325.50	*a* ^3^F_2_—*u* ${\;}^{3}D_{2}^{{}^{\circ}}$
10	2912.745	34321.83	*a* ^5^D_3_—*v* ${\;}^{3}D_{2}^{{}^{\circ}}$
60	2913.163	34316.90	*a* ^3^P_1_— $11_{0}^{{}^{\circ}}$
50	2914.294	34303.58	*a* ^3^P_0_— $9_{1}^{{}^{\circ}}$
25	2915.614	34288.05	*a* ^3^G_5_—*x* ${\;}^{1}H_{5}^{{}^{\circ}}$
150	2916.251	34280.56	*a* ^5^F_4_—*y* ${\;}^{5}F_{4}^{{}^{\circ}}$
40	2917.132	34270.21	*a* ^3^P_1_— $9_{1}^{{}^{\circ}}$
50	2917.764	34262.79	*a* ^3^F_3_—*x* ${\;}^{3}F_{4}^{{}^{\circ}}$
75	2919.604	34241.20	*a* ^5^F_4_—*z* ${\;}^{3}P_{1}^{{}^{\circ}}$
20	2920.254	34233.58	*a* ^1^G_4_— $43_{3}^{{}^{\circ}}$
1*H*	2920.765	34227.59	*a* ^5^P_3_—*y* ${\;}^{1}F_{3}^{{}^{\circ}}$
30	2920.949	34225.43	*a* ^3^P_0_— $8_{1}^{{}^{\circ}}$
3	2921.140	34223.19	*a* ^3^F_4_—*x* ${\;}^{3}D_{3}^{{}^{\circ}}$
3	2921.406	34220.08	*b* ^3^P_0_— $45_{1}^{{}^{\circ}}$
1	2923.677	34193.50	*a* ^3^G_5_— $13_{4}^{{}^{\circ}}$
5	2923.804	34192.01	*a* ^3^P_1_— $8_{1}^{{}^{\circ}}$
20	2925.067	34177.25	*a* ^3^P_2_—*x* ${\;}^{5}F_{3}^{{}^{\circ}}$
2	2925.748	34169.30	*a* ^3^G_3_— $28_{3}^{{}^{\circ}}$
5	2925.841	34168.21	*a* ^5^P_3_—*x* ${\;}^{3}G_{4}^{{}^{\circ}}$
60	2927.119	34153.29	*a* ^1^G_4_— $42_{4}^{{}^{\circ}}$
50	2928.487	34137.34	*a ^3^*P_1_—*t* ${\;}^{3}D_{2}^{{}^{\circ}}$
1	2929.122	34129.94	*a* ^3^G_4_—*w* ${\;}^{3}F_{3}^{{}^{\circ}}$
40	2929.434	34126.30	*a* ^5^P_3_— *v* ${\;}^{3}D_{2}^{{}^{\circ}}$
5*H*	2932.593	34089.54	*a* ^3^G_3_— $25_{3}^{{}^{\circ}}$
75	2934.173	34071.19	*a* ^3^F_4_—*z* ${\;}^{1}H_{5}^{{}^{\circ}}$
2	2934.658	34065.56	*a* ^1^G_4_— $40_{3}^{{}^{\circ}}$
60	2936.005	34049.93	*a* ^5^D_2_—*y* ${\;}^{3}S_{1}^{{}^{\circ}}$
30	2936.247	34047.12	*a* ^5^F_2_—*y* ${\;}^{3}D_{3}^{{}^{\circ}}$
30	2937.336	34034.50	*a* ^5^D_1_—*y* ${\;}^{3}S_{1}^{{}^{\circ}}$
1	2937.850	34028.54	*b* ^3^F_2_— $6_{3}^{{}^{\circ}}$
75	2939.135	34013.67	*a* ^5^F_1_—*z* ${\;}^{3}P_{2}^{{}^{\circ}}$
25	2939.676	34007.41	*b* ^3^F_3_— *y* ${\;}^{3}H_{4}^{{}^{\circ}}$
80	2939.938	34004.38	*a* ^3^P_1_—*w* ${\;}^{5}D_{2}^{{}^{\circ}}$
100	2940.352	33999.59	*a* ^5^D_4_—*w* ${\;}^{3}D_{3}^{{}^{\circ}}$
1	2941.212	33989.65	*b* ^3^P_2_— $23_{3}^{{}^{\circ}}$
10	2941.762	33983.30	*a* ^3^D_2_— $44_{2}^{{}^{\circ}}$
60	2943.470	33963.58	*a* ^5^P_2_—*x* ${\;}^{3}D_{2}^{{}^{\circ}}$
100	2943.919	33958.40	*a* ^5^D_3_—*w* ${\;}^{3}D_{2}^{{}^{\circ}}$
10	2944.380	33953.08	*b* ^3^F_3_—*x* ${\;}^{5}F_{4}^{{}^{\circ}}$
100	2946.981	33923.12	*a* ^3^F_3_—*x* ${\;}^{3}D_{2}^{{}^{\circ}}$
2	2948.847	33901.65	*b* ^3^P_2_—*s* ${\;}^{3}D_{2}^{{}^{\circ}}$
150	2949.492	33894.24	*a* ^3^F_4_— $1_{4}^{{}^{\circ}}$
2	2949.947	33889.01	*a* ^5^P_1_—*u* ${\;}^{3}D_{2}^{{}^{\circ}}$
50	2950.532	33882.29	*a* ^3^H_5_—*x* ${\;}^{3}G_{5}^{{}^{\circ}}$
60	2951.401	33872.31	*a* ^5^F_3_—*y* ${\;}^{5}F_{2}^{{}^{\circ}}$
80	2952.489	33859.84	*a* ^5^F_1_—*y* ${\;}^{3}D_{2}^{{}^{\circ}}$
1	2952.931	33854.77	*b* ^3^P_2_—*x* ${\;}^{5}P_{1}^{{}^{\circ}}$
125	2954.484	33836.97	*a* ^5^D_2_—*w* ${\;}^{3}D_{1}^{{}^{\circ}}$
50	2955.348	33827.08	*a* ^3^G_3_— $22_{3}^{{}^{\circ}}$
20	2955.593	33824.28	*a* ^5^D_1_—*v* ${\;}^{3}D_{2}^{{}^{\circ}}$
20	2955.833	33821.53	*a* ^5^D_1_—*w* ${\;}^{3}D_{1}^{{}^{\circ}}$
5	2956.133	33818.10	*a* ^3^P_2_— $4_{3}^{{}^{\circ}}$
4	2957.166	33806.28	*a* ^3^P_1_—*x* ${\;}^{5}F_{2}^{{}^{\circ}}$
100	2957.996	33796.80	*a* ^3^F_3_—*v* ${\;}^{3}D_{3}^{{}^{\circ}}$
2	2958.858	33786.95	*b* ^3^F_3_— $4_{3}^{{}^{\circ}}$
100	2959.729	33777.01	$\{\;\begin{array}{c} a{\;}^{5}D_{4}—x{\;}^{3}F_{3}^{{}^{\circ}}\\ b{\;}^{3}P_{0}—39_{1}^{{}^{\circ}} \end{array}$
40	2960.965	33762.91	*a* ^5^P_3_—*w* ${\;}^{3}D_{2}^{{}^{\circ}}$
1	2961.334	33758.70	*a* ^3^D_3_—*v* ${\;}^{3}G_{4}^{{}^{\circ}}$
75	2961.685	33754.70	*b* ^3^F_2_—*x* ${\;}^{1}F_{3}^{{}^{\circ}}$
100	2963.714	33731.59	*a* F_4_^3^—*y* ${\;}^{3}G_{4}^{{}^{\circ}}$
15	2964.295	33724.98	*a* ^3^D_2_— $41_{2}^{{}^{\circ}}$
150	2965.168	33715.06	*a* ^5^F_3_—*y* ${\;}^{5}F_{3}^{{}^{\circ}}$
15	2965.694	33709.08	*a* ^3^H_5_—*v* ${\;}^{3}G_{4}^{{}^{\circ}}$
20	2966.549	33699.36	*b* ^3^P_2_— $20_{2}^{{}^{\circ}}$
1	2967.064	33693.51	*b* ^3^P_2_—*s* ${\;}^{3}D_{3}^{{}^{\circ}}$
50	2967.342	33690.36	*a* ^3^F_4_—*y* ${\;}^{3}G_{3}^{{}^{\circ}}$
30	2968.398	33678.37	*a* ^3^G_4_—*x* ${\;}^{1}H_{5}^{{}^{\circ}}$
30	2968.468	33677.58	*a* ^3^P_2_— $3_{1}^{{}^{\circ}}$
100	2968.952	33672.09	*a* ^3^F_3_—*z* ${\;}^{1}D_{2}^{{}^{\circ}}$
2	2969.854	33661.86	*a* ^1^H_5_— $62_{4}^{{}^{\circ}}$
1	2971.261	33645.92	*a* ^3^G_3_— $20_{2}^{{}^{\circ}}$
25	2971.757	33640.30	*a* ^3^G_3_—*s* ${\;}^{3}D_{3}^{{}^{\circ}}$
1	2972.98	33626.47	*a* ^3^D_1_— $58_{1}^{{}^{\circ}}$
100	2973.976	33615.21	*a* ^5^D_0_—*y* ${\;}^{3}s_{1}^{{}^{\circ}}$
30	2974.327	33611.24	*a* ^3^F_2_—*x* ${\;}^{3}P_{2}^{{}^{\circ}}$
25	2975.122	33602.26	*b* ^3^P_2_—*w* ${\;}^{3}F_{2}^{{}^{\circ}}$
100	2976.923	33581.93	*a* ^5^F_4_—*y* ${\;}^{5}F_{5}^{{}^{\circ}}$
30	2978.361	33565.72	*b* ^3^P_1_—*s* ${\;}^{3}D_{2}^{{}^{\circ}}$
60	2979.713	33550.49	*a* ^3^D_2_— $39_{1}^{{}^{\circ}}$
75	2981.934	33525.50	*a* ^5^F_2_—*y* ${\;}^{5}F_{1}^{{}^{\circ}}$
10	2982.522	33518.89	*b* ^3^P_1_—*x* ${\;}^{5}P_{1}^{{}^{\circ}}$
20	2985.812	33481.96	*a* ^3^H_5_— $52_{4}^{{}^{\circ}}$
25	2986.328	33476.18	*a* ^5^D_2_—*w* ${\;}^{3}D_{2}^{{}^{\circ}}$
2	2987.269	33465.63	*a* ^5^D_4_—*z* ${\;}^{1}F_{3}^{{}^{\circ}}$
30	2987.694	33460.87	*a* ^5^D_1_—*w* ${\;}^{3}D_{2}^{{}^{\circ}}$
6	2987.928	33458.25	*a* ^3^G_3_— $17_{4}^{{}^{\circ}}$
10	2988.089	33456.45	*a* ^3^G_4_— $12_{4}^{{}^{\circ}}$
300	2988.945	33446.86	*a* ^5^F_5_—*y* ${\;}^{5}D_{4}^{{}^{\circ}}$
50	2989.652	33438.95	$\{\;\begin{array}{c} a{\;}^{5}P_{2}—w{\;}^{3}D_{3}^{{}^{\circ}}\\ a{\;}^{3}D_{2}—35_{3}^{{}^{\circ}} \end{array}$
40	2990.289	33431.83	*a* ^5^D_3_—*x* ${\;}^{3}D_{2}^{{}^{\circ}}$
40	2992.120	33411.37	*a* ^3^P_1_—*y* ${\;}^{5}s_{2}^{{}^{\circ}}$
30	2992.948	33402.13	*a* ^5^D_0_—*w* ${\;}^{3}D_{1}^{{}^{\circ}}$
100	2993.273	33398.51	*a* ^3^F_3_—*w* ${\;}^{3}D_{3}^{{}^{\circ}}$
150	2994.967	33379.62	*a* ^5^F_3_—*y* ${\;}^{5}F_{4}^{{}^{\circ}}$
75	2996.891	33358.19	*a* ^5^D_2_—*z* ${\;}^{1}P_{1}^{{}^{\circ}}$
50	2997.421	33352.29	*a* ^3^P_2_—*x* ${\;}^{3}G_{3}^{{}^{\circ}}$
50	2997.613	33350.15	*a* ^3^F_2_—*w* ${\;}^{3}D_{2}^{{}^{\circ}}$
60	3000.217	33321.21	*b* ^3^F_3_—*x* ${\;}^{3}G_{3}^{{}^{\circ}}$
60	3001.634	33305.48	*a* ^5^D_3_—*v* ${\;}^{3}D_{3}^{{}^{\circ}}$
5	3002.050	33300.86	*b* ^3^P_2_—*w* ${\;}^{3}F_{3}^{{}^{\circ}}$
20	3003.473	33285.09	*a* ^5^D_4_—*x* ${\;}^{3}D_{3}^{{}^{\circ}}$
3	3003.939	33279.92	*a* ^3^P_2_—*y* ${\;}^{1}D_{2}^{{}^{\circ}}$
15	3004.211	33276.91	*a* ^5^P_1_—*v* ${\;}^{3}D_{2}^{{}^{\circ}}$
50	3004.598	33272.62	*a* ^3^D_2_— $33_{2}^{{}^{\circ}}$
300	3006.588	33250.60	$\{\;\begin{array}{c} a{\;}^{5}F_{2}—y{\;}^{5}F_{2}^{{}^{\circ}}\\ a{\;}^{3}G_{4}—10_{4}^{{}^{\circ}} \end{array}$
2*h*	3007.88	33236.32	*a* ^5^P_3_—*x* ${\;}^{3}D_{2}^{{}^{\circ}}$
75	3008.262	33232.10	*a* ^3^F_2_—*z* ${\;}^{1}P_{1}^{{}^{\circ}}$
75	3008.797	33226.19	*b* ^3^F_4_—*x* ${\;}^{3}F_{4}^{{}^{\circ}}$
1*h*	3009.537	33218.02	*a* ^3^P_2_—*u* ${\;}^{3}D_{1}^{{}^{\circ}}$
20	3009.687	33216.37	*a* ^5^P_2_—*x* ${\;}^{3}F_{3}^{{}^{\circ}}$
40	3010.501	33207.39	*a* ^3^H_5_— $51_{4}^{{}^{\circ}}$
3	3011.607	33195.19	*b* ^3^P_1_— $18_{0}^{{}^{\circ}}$
75	3012.915	33180.78	*a* ^5^D_3_—*z* ${\;}^{1}D_{2}^{{}^{\circ}}$
75	3013.354	33175.95	*a* ^3^F_3_—*x* ${\;}^{3}F_{3}^{{}^{\circ}}$
1	3014.070	33168.07	*a* ^1^G_4_— $28_{3}^{{}^{\circ}}$
1	3015.008	33157.75	$\{\;\begin{array}{c} a{\;}^{3}G_{5}—y{\;}^{1}G_{4}^{{}^{\circ}}\\ b{\;}^{3}P_{2}—x{\;}^{5}P_{2}^{{}^{\circ}} \end{array}$
2	3016.684	33139.33	*a* ^5^P_2_—*x* ${\;}^{3}F_{2}^{{}^{\circ}}$
200	3017.235	33133.28	$\{\;\begin{array}{c} a{\;}^{5}F_{1}—y{\;}^{5}F_{1}^{{}^{\circ}}\\ a{\;}^{5}D_{4}—z{\;}^{1}H_{5}^{{}^{\circ}} \end{array}$
5	3019.291	33110.71	*a* ^3^D_2_— $32_{2}^{{}^{\circ}}$
15	3019.367	33109.88	*a* ^5^P_3_—*v* ${\;}^{3}D_{3}^{{}^{\circ}}$
1*w*	3020.218	33100.55	*b* ^3^P_2_—*x* ${\;}^{5}P_{3}^{{}^{\circ}}$
150	3020.871	33093.40	*a* ^5^F_2_—*y* ${\;}^{5}F_{3}^{{}^{\circ}}$
20	3025.085	33047.30	*a* ^3^G_3_—*x* ${\;}^{5}P_{3}^{{}^{\circ}}$
60	3027.076	33025.56	*a* ^3^H_4_—*v* ${\;}^{3}G_{5}^{{}^{\circ}}$
5	3029.373	33000.52	*a* ^5^P_1_—*z* ${\;}^{1}S_{0}^{{}^{\circ}}$
1	3029.948	32994.26	*b* ^3^F_2_— $4_{3}^{{}^{\circ}}$
60	3030.776	32985.25	*a* ^5^P_3_—*z* ${\;}^{1}D_{2}^{{}^{\circ}}$
10	3031.107	32981.65	*b* ^3^P_0_— $26_{1}^{{}^{\circ}}$
50	3031.899	32973.03	*a* ^5^P_2_—*x* ${\;}^{3}D_{1}^{{}^{\circ}}$
100	3033.449	32956.18	*a* ^5^D_4_— $1_{4}^{{}^{\circ}}$
50	3034.058	32949.57	*a* ^5^D_2_—*x* ${\;}^{3}D_{2}^{{}^{\circ}}$
50	3035.471	32934.23	*a* ^5^D_1_—*x* ${\;}^{3}D_{2}^{{}^{\circ}}$
40	3036.463	32923.47	*a* ^5^D_0_—*z* ${\;}^{1}P_{1}^{{}^{\circ}}$
5	3037.376	32913.58	*a* ^5^P_1_—*w* ${\;}^{3}D_{2}^{{}^{\circ}}$
40	3037.961	32907.24	*a* ^5^D_3_—*w* ${\;}^{3}D_{3}^{{}^{\circ}}$
50	3038.169	32904.99	*a* ^5^P_2_—*z* ${\;}^{1}F_{3}^{{}^{\circ}}$
10	3039.674	32888.70	*a* ^3^F_4_—*y* ${\;}^{3}F_{3}^{{}^{\circ}}$
25	3039.952	32885.69	*a* ^3^P_2_—*u* ${\;}^{3}D_{2}^{{}^{\circ}}$
100	3040.319	32881.72	*a* ^5^F_4_— *z* ${\;}^{5}P_{3}^{{}^{\circ}}$
40	3041.906	32864.56	*a* ^3^F_3_—*z* ${\;}^{1}F_{3}^{{}^{\circ}}$
100	3042.480	32858.36	*a* ^5^F_4_—*y* ${\;}^{5}F_{2}^{{}^{\circ}}$
60	3042.829	32854.59	*b* ^3^F_3_—*u* ${\;}^{3}D_{2}^{{}^{\circ}}$
20	3043.045	32852.26	*a* ^3^H_4_—*v* ${\;}^{3}G_{4}^{{}^{\circ}}$
1*h*	3043.553	32846.78	*a* ^3^D_3_— $44_{2}^{{}^{\circ}}$
125	3045.715	32823.47	$\{\;\begin{array}{c} a{\;}^{3}F_{2}—x{\;}^{3}D_{2}^{{}^{\circ}}\\ a{\;}^{5}D_{2}—v{\;}^{3}D_{3}^{{}^{\circ}} \end{array}$
2	3047.187	32807.61	*b* ^3^P_1_— $15_{1}^{{}^{\circ}}$
1	3048.30	32795.63	*a* ^5^P_1_—*z* ${\;}^{1}P_{1}^{{}^{\circ}}$
75	3048.496	32793.52	*a* ^5^D_4_—*y* ${\;}^{3}G_{4}^{{}^{\circ}}$
150	3048.793	32790.33	*a* ^5^F_3_—*z* ${\;}^{5}P_{2}^{{}^{\circ}}$
2	3049.066	32787.39	*b* ^3^F_2_—*x* ${\;}^{3}P_{2}^{{}^{\circ}}$
50	3051.593	32760.24	*b* ^3^F_4_—*y* ${\;}^{3}D_{3}^{{}^{\circ}}$
1	3052.075	32755.07	*a* ^3^D_2_— $26_{1}^{{}^{\circ}}$
15	3052.334	32752.29	*a* ^5^D_4_—*y* ${\;}^{3}G_{3}^{{}^{\circ}}$
3	3054.563	32728.39	*a* ^3^F_4_—*z* ${\;}^{3}H_{4}^{{}^{\circ}}$
150	3054.934	32724.42	*a* ^5^P_2_—*x* ${\;}^{3}D_{3}^{{}^{\circ}}$
30	3056.108	32711.84	$\{\;\begin{array}{c} a{\;}^{5}P_{3}—w{\;}^{3}D_{3}^{{}^{\circ}}\\ a{\;}^{3}G_{4}—x{\;}^{1}G_{4}^{{}^{\circ}} \end{array}$
8	3056.754	32704.93	*a* ^3^D_4_— $50_{2}^{{}^{\circ}}$
50	3057.353	32698.52	*a* ^5^D_2_—*z* ${\;}^{1}D_{2}^{{}^{\circ}}$
75	3058.650	32684.66	*a* ^5^D_3_—*x* ${\;}^{3}F_{3}^{{}^{\circ}}$
75	3058.795	32683.11	*a* ^5^D_1_—*z* ${\;}^{1}D_{2}^{{}^{\circ}}$
1	3059.733	32673.09	*b* ^3^P_0_—*x* ${\;}^{5}P_{1}^{{}^{\circ}}$
25	3062.043	32648.44	*c* ^3^F_4_— $63_{3}^{{}^{\circ}}$
1	3062.914	32639.16	*a* ^1^G_4_—*s* ${\;}^{3}D_{3}^{{}^{\circ}}$
20*w*	3064.22	32625.25	*a* ^3^H_4_— $52_{4}^{{}^{\circ}}$
250	3064.834	32618.71	*b* ^3^F_4_—*x* ${\;}^{3}G_{5}^{{}^{\circ}}$
1	3065.882	32607.57	*a* ^5^D_3_—*x* ${\;}^{3}F_{2}^{{}^{\circ}}$
5	3067.665	32588.61	*a* ^3^D_3_— $41_{2}^{{}^{\circ}}$
50	3069.181	32572.52	*a* ^3^F_2_—*z* ${\;}^{1}D_{2}^{{}^{\circ}}$
30	3071.484	32548.09	*a* ^3^G_4_—*y* ${\;}^{1}G_{4}^{{}^{\circ}}$
80	3073.337	32528.47	*b* ^3^F_2_—*x* ${\;}^{3}G_{3}^{{}^{\circ}}$
60	3076.773	32492.15	*a* ^3^F_4_—*z* ${\;}^{1}G_{4}^{{}^{\circ}}$
20	3077.057	32489.15	*a* ^5^P_3_—*x* ${\;}^{3}F_{3}^{{}^{\circ}}$
30	3077.542	32484.03	*a* ^3^P_2_—*y* ${\;}^{3}S_{1}^{{}^{\circ}}$
1	3078.679	32472.03	*a* ^3^D_2_— $22_{3}^{{}^{\circ}}$
50	3080.188	32456.12	*b* ^3^F_2_—*y* ${\;}^{1}D_{2}^{{}^{\circ}}$
75	3080.898	32448.64	*a* ^3^P_1_—*x* ${\;}^{3}P_{2}^{{}^{\circ}}$
60	3083.145	32425.00	*a* ^5^D_2_—*w* ${\;}^{3}D_{3}^{{}^{\circ}}$
30	3084.521	32410.53	*b* ^3^P_2_— $9_{1}^{{}^{\circ}}$
4	3084.877	32406.79	*a* ^3^D_3_— $38_{4}^{{}^{\circ}}$
5	3085.473	32400.53	*a* ^3^G_5_—*x* ${\;}^{5}F_{4}^{{}^{\circ}}$
75	3086.076	32394.20	*b* ^3^F_2_—*u* ${\;}^{3}D_{1}^{{}^{\circ}}$
20	3086.520	32389.54	*a* ^5^P_2_—*y* ${\;}^{3}F_{2}^{{}^{\circ}}$
10	3086.771	32386.91	*a* ^5^P_1_—*x* ${\;}^{3}D_{2}^{{}^{\circ}}$
30	3086.929	32385.25	*a* ^3^G_4_—*x* ${\;}^{1}F_{3}^{{}^{\circ}}$
1*h*	3087.924	32374.82	*a* ^3^P_2_—*y* ${\;}^{1}F_{3}^{{}^{\circ}}$
25	3088.078	32373.20	*a* ^5^D_3_—2 ${\;}^{1}F_{3}^{{}^{\circ}}$
1	3088.245	32371.45	*a* ^1^D_2_— $50_{2}^{{}^{\circ}}$
80	3089.147	32362.00	*b* ^3^F_4_—*w* ${\;}^{3}D_{3}^{{}^{\circ}}$
60	3089.802	32355.14	*a* ^3^F_3_— $1_{4}^{{}^{\circ}}$
50	3090.225	32350.71	*a* ^3^H_4_— $51_{4}^{{}^{\circ}}$
40	3090.896	32343.69	*b* ^3^F_3_—*y* ${\;}^{1}F_{3}^{{}^{\circ}}$
70	3091.867	32333.53	*a* ^5^F_2_—*z* ${\;}^{5}P_{1}^{{}^{\circ}}$
100	3096.565	32284.48	*b* ^3^F_3_—*x* ${\;}^{3}G_{4}^{{}^{\circ}}$
40	3097.225	32277.60	*b* ^3^P_2_—*t* ${\;}^{3}D_{2}^{{}^{\circ}}$
75	3097.606	32273.63	*a* ^3^P_2_—*v* ${\;}^{3}D_{2}^{{}^{\circ}}$
3	3097.865	32270.93	*a* ^3^P_2_—*w* ${\;}^{3}D_{1}^{{}^{\circ}}$
250	3099.284	32256.16	*a* ^5^F_4_—*y* ${\;}^{5}D_{4}^{{}^{\circ}}$
10	3100.582	32242.65	*b* ^3^F_3_—*v* ${\;}^{3}D_{2}^{{}^{\circ}}$
100	3100.836	32240.01	*a* ^5^F_4_—*y* ${\;}^{5}D_{3}^{{}^{\circ}}$
10	3102.364	32224.13	*a* ^3^G_3_—*t* ${\;}^{3}D_{2}^{{}^{\circ}}$
2	3103.748	32209.76	*b* ^3^P_1_—*w* ${\;}^{5}D_{1}^{{}^{\circ}}$
50	3104.457	32202.41	*a* ^5^D_2_—*x* ${\;}^{3}F_{3}^{{}^{\circ}}$
50	3105.278	32193.90	*a* ^3^D_2_—*w* ${\;}^{3}F_{2}^{{}^{\circ}}$
50	3105.407	32192.56	$\{\;\begin{array}{c} a{\;}^{5}D_{3}—x{\;}^{3}D_{3}^{{}^{\circ}}\\ a{\;}^{3}F_{3}—y{\;}^{3}G_{4}^{{}^{\circ}} \end{array}$
50	3106.837	32177.74	*a* ^5^P_3_—*z* ${\;}^{1}F_{3}^{{}^{\circ}}$
150	3107.720	32168.60	*a* ^5^F_2_—*z* ${\;}^{5}P_{2}^{{}^{\circ}}$
30	3108.423	32161.32	*a* ^3^F_4_—*y* ${\;}^{5}P_{3}^{{}^{\circ}}$
35	3109.394	32151.28	*a* ^3^F_3_— *y* ${\;}^{3}G_{3}^{{}^{\circ}}$
75	3110.536	32139.48	*b* ^3^F_4_—*x* ${\;}^{3}F_{3}^{{}^{\circ}}$
50	3111.910	32125.29	*a* ^5^D_2_—*x* ${\;}^{3}F_{2}^{{}^{\circ}}$
30	3112.299	32121.27	*b* ^3^P_1_— $11_{0}^{{}^{\circ}}$
70	3112.676	32117.38	*a* ^3^P_1_—*y* ${\;}^{1}D_{2}^{{}^{\circ}}$
80	3113.393	32109.99	*a* ^5^D_1_—*x* ${\;}^{3}F_{2}^{{}^{\circ}}$
8	3115.436	32088.93	*a* ^3^P_0_—*u* ${\;}^{3}D_{1}^{{}^{\circ}}$
50	3116.657	32076.36	*a* ^3^F_2_—*x* ${\;}^{3}F_{3}^{{}^{\circ}}$
25	3116.834	32074.54	$\{\;\begin{array}{c} b{\;}^{3}P_{1}—9_{1}^{{}^{\circ}}\\ c{\;}^{3}F_{4}—62_{4}^{{}^{\circ}} \end{array}$
3	3117.454	32068.16	*a* ^3^H_4_— $47_{4}^{{}^{\circ}}$
4	3117.631	32066.34	*a* ^3^D_1_— $41_{2}^{{}^{\circ}}$
80	3118.065	32061.88	*b* ^3^F*_2_*—*u* ${\;}^{3}D_{2}^{{}^{\circ}}$
80	3118.687	32055.48	*a* ^3^P_1_—*u* ${\;}^{3}D_{1}^{{}^{\circ}}$
30	3120.542	32036.42	*a* ^3^G_5_—*w* ${\;}^{5}D_{4}^{{}^{\circ}}$
100	3124.164	31999.29	*a* ^3^F_2_—*x* ${\;}^{3}F_{2}^{{}^{\circ}}$
25	3124.362	31997.26	*a* ^5^P_3_—*x* ${\;}^{3}D_{3}^{{}^{\circ}}$
50	3124.601	31994.81	*a* ^1^G_4_— $14_{4}^{{}^{\circ}}$
15	3125.651	31984.06	*a* ^3^G_4_—*x* ${\;}^{5}F_{3}^{{}^{\circ}}$
100	3125.958	31980.92	*a* ^5^F_3_—*z* ${\;}^{5}P_{3}^{{}^{\circ}}$
015	3126.613	31974.22	*a* ^3^D_3_— $32_{2}^{{}^{\circ}}$
2	3127.258	31967.63	*a* ^5^D_4_—*y* ${\;}^{3}G_{5}^{{}^{\circ}}$
2	3128.085	31959.18	*a* ^3^D_2_—*x* ${\;}^{3}D_{1}^{{}^{\circ}}$
1	3128.920	31950.65	*a* ^5^D_4_—*y* ${\;}^{3}F_{3}^{{}^{\circ}}$
2	3129.135	31948.45	*c* ^3^F_2_— $63_{3}^{{}^{\circ}}$
30	3129.601	31943.70	*a* ^5^D_1_—*x* ${\;}^{3}D_{1}^{{}^{\circ}}$
75	3129.835	31941.31	*a* ^5^F_1_—*z* ${\;}^{5}P_{1}^{{}^{\circ}}$
100	3132.874	31910.33	*a* ^3^P_2_—*w* ${\;}^{3}D_{2}^{{}^{\circ}}$
4	3133.511	31903.84	$\{\;\begin{array}{c} a{\;}^{3}H_{5}—31_{4}^{{}^{\circ}}\\ b{\;}^{3}P_{2}—7_{3}^{{}^{\circ}} \end{array}$
20	3133.687	31902.05	*a* ^3^G_5_—*y* ${\;}^{3}H_{5}^{{}^{\circ}}$
1	3134.631	31892.44	*a* ^3^D_2_—*w* ${\;}^{3}F_{3}^{{}^{\circ}}$
2	3134.777	31890.95	*a* ^5^D_2_—*z* ${\;}^{1}F_{3}^{{}^{\circ}}$
2	3135.066	31888.01	*a* ^1^D_2_— $43_{3}^{{}^{\circ}}$
25	3135.933	31879.20	*b* ^3^F_3_—*w* ${\;}^{3}D_{2}^{{}^{\circ}}$
5	3136.349	31874.97	*a* ^3^H_6_— $21_{5}^{{}^{\circ}}$
100	3136.554	31872.89	*a* ^3^P_2_—*y* ${\;}^{3}P_{1}^{{}^{\circ}}$
30	3136.911	31869.26	*a* ^3^H_5_— $30_{4}^{{}^{\circ}}$
10	3138.761	31850.48	*a* ^3^G_3_— $7_{3}^{{}^{\circ}}$
50	3139.273	31845.28	*a* ^3^G_4_—*y* ${\;}^{3}H_{4}^{{}^{\circ}}$
10	3140.086	31837.04	*a* ^3^H_4_— $43_{3}^{{}^{\circ}}$
75	3140.485	31832.99	*a* ^3^F_2_—*x* ${\;}^{3}D_{1}^{{}^{\circ}}$
125	3140.979	31827.99	*b* ^3^F_4_—*z* ${\;}^{1}F_{3}^{{}^{\circ}}$
5	3142.522	31812.36	*a* ^3^D_3_— $29_{3}^{{}^{\circ}}$
1	3142.88	31808.74	*b* ^3^P_1_—*w* ${\;}^{5}D_{2}^{{}^{\circ}}$
150	3144.265	31794.72	*a* ^1^G_4_—*x* ${\;}^{1}H_{5}^{{}^{\circ}}$
1	3144.502	31792.33	*a* ^3^P_2_—*z* ${\;}^{1}P_{1}^{{}^{\circ}}$
1	3144.579	31791.55	*a* ^3^G_5_— $2_{6}^{{}^{\circ}}$
1	3144.640	31790.93	*a* ^3^G_4_—*x* ${\;}^{5}F_{4}^{{}^{\circ}}$
15	3144.715	31790 18	*a* ^3^D_4_— *z* ${\;}^{3}H_{4}^{{}^{\circ}}$
50	3146.075	31776.44	*a* ^5^F_1_—*z* ${\;}^{5}P_{2}^{{}^{\circ}}$
100	3147.214	31764.94	*a* ^3^F_2_—*z* ${\;}^{1}F_{3}^{{}^{\circ}}$
10	3148.021	31756.79	*a* ^3^H_4_— $42_{4}^{{}^{\circ}}$
75	3148.493	31752.03	*a* ^3^F_4_—*z* ${\;}^{3}H_{5}^{{}^{\circ}}$
4	3150.172	31735.11	*a* ^3^D_2_— $15_{1}^{{}^{\circ}}$
100	3150.699	31729.80	*a* ^5^P_2_—*y* ${\;}^{5}P_{1}^{{}^{\circ}}$
80	3151.363	31723.12	*a* ^3^P_1_—*u* ${\;}^{3}D_{2}^{{}^{\circ}}$
2	3151.686	31719.86	*a* ^1^D_2_— $40_{3}^{{}^{\circ}}$
1	3152.611	31710.56	*a* ^5^D_2_—*x* ${\;}^{3}D_{3}^{{}^{\circ}}$
1*h*	3153.583	31700.78	*b* ^3^P_0_—*y* ${\;}^{1}P_{1}^{{}^{\circ}}$
150	3153.831	31698.29	*a* ^5^P_2_—*y* ${\;}^{3}P_{2}^{{}^{\circ}}$
25	3154.428	31692.29	*b* ^3^F_3_—*x* ${\;}^{3}F_{4}^{{}^{\circ}}$
3	3155.895	31677.56	*a* ^3^D_3_— $28_{3}^{{}^{\circ}}$
25	3156.821	31668.27	*a* ^5^P_3_— $1_{4}^{{}^{\circ}}$
20	3157.090	31665.57	*a* ^3^G_3_—*y* ${\;}^{1}G_{4}^{{}^{\circ}}$
25	3157.414	31662.32	*a* ^5^P_3_—*y* ${\;}^{3}F_{2}^{{}^{\circ}}$
20	3157.626	31660.20	$\{\;\begin{array}{c} b{\;}^{3}F_{2}—y{\;}^{3}S_{1}^{{}^{\circ}}\\ a{\;}^{5}D_{3}—y{\;}^{3}G_{3}^{{}^{\circ}} \end{array}$
15	3157.850	31657.95	*a* ^3^F_3_—*y* ${\;}^{3}P_{2}^{{}^{\circ}}$
1	3158.057	31655.88	*a* ^3^G_5_—*w* ${\;}^{3}G_{4}^{{}^{\circ}}$
125	3158.899	31647.44	*b* ^3^F_4_—*x* ${\;}^{3}D_{3}^{{}^{\circ}}$
200	3159.935	31637.06	*a* ^5^F_3_—*y* ${\;}^{5}D_{2}^{{}^{\circ}}$
1	3160.924	31627.16	*a* ^3^D_3_— $27_{4}^{{}^{\circ}}$
3	3162.223	31614.17	*a* ^3^D_1_— $33_{32}^{{}^{\circ}}$
1*h*	3162.589	31610.52	*b* ^3^P_1_—*x* ${\;}^{5}F_{2}^{{}^{\circ}}$
25	3163.831	31598.11	*a* ^3^D_3_— $25_{3}^{{}^{\circ}}$
25	3165.195	31584.49	*a* ^3^F_2_—*x* ${\;}^{3}D_{3}^{{}^{\circ}}$
1	3165.887	31577.59	*a* ^3^H_5_— $27_{4}^{{}^{\circ}}$
2	3166.339	31573.08	*a* ^1^G_4_— $12_{4}^{{}^{\circ}}$
5	3167.381	31562.69	*a* ^5^P_1_—*x* ${\;}^{3}F_{2}^{{}^{\circ}}$
8	3167.820	31558.32	*a* ^1^D_2_— $39_{1}^{{}^{\circ}}$
40	3168.243	31554.10	*a* ^5^D_4_—*z* ${\;}^{1}G_{4}^{{}^{\circ}}$
30	3168.489	31551.65	*b* ^3^P_2_— *y* ${\;}^{5}S_{2}^{{}^{\circ}}$
60	3168.560	31550.95	*b* ^3^F_2_—*y* ${\;}^{1}F_{3}^{{}^{\circ}}$
80	3170.088	31535.74	*a* ^3^G_5_—*w* ${\;}^{3}G_{5}^{{}^{\circ}}$
50	3171.239	31524.30	*a* ^5^D_0_—*x* ${\;}^{3}D_{1}^{{}^{\circ}}$
25	3173.110	31505.71	*a* ^5^P_3_—*z* ${\;}^{3}G_{4}^{{}^{\circ}}$
25	3173.394	31502.89	*a* ^3^G_3_—*x* ${\;}^{1}F_{3}^{{}^{\circ}}$
60	3174.128	31495.60	*b* ^3^F_4_—*z* ${\;}^{1}H_{5}^{{}^{\circ}}$
50	3176.286	31474.21	*a* ^3^D_2_—*y* ${\;}^{1}P_{1}^{{}^{\circ}}$
25	3178.736	31449.95	*b* ^3^F_2_—*v* ${\;}^{3}D_{2}^{{}^{\circ}}$
2	3179.025	31447.09	*a* ^1^D_2_— $35_{3}^{{}^{\circ}}$
30	3179.263	31444.73	*a* ^3^D_3_— $23_{3}^{{}^{\circ}}$
5	3181.189	31425.70	*b* ^3^P_2_—*w* ${\;}^{5}D_{3}^{{}^{\circ}}$
1	3183.137	31406.47	*a* ^3^H_4_— $36_{5}^{{}^{\circ}}$
2	3183.513	31402.76	*a* ^3^H_5_— $24_{5}^{{}^{\circ}}$
1	3184.177	31396.21	$\{\;\begin{array}{c} a{\;}^{5}P_{1}—x{\;}^{3}D_{1}^{{}^{\circ}}\\ a{\;}^{3}H_{4}—35_{3}^{{}^{\circ}} \end{array}$
50	3184.847	31389.60	*a* ^3^G_5_—*y* ${\;}^{1}H_{5}^{{}^{\circ}}$
10	3185.447	31383.69	*a* ^3^P_2_—*x* ${\;}^{3}D_{2}^{{}^{\circ}}$
200	3186.042	31377.83	*a* ^5^F_2_— *y* ${\;}^{5}D_{1}^{{}^{\circ}}$
1	3186.584	31372.49	*a* ^3^G_3_—*w* ${\;}^{5}D_{3}^{{}^{\circ}}$
1	3187.822	31360.31	*a* ^5^D_1_—*y* ${\;}^{3}F_{2}^{{}^{\circ}}$
40	3187.939	31359.16	$\{\;\begin{array}{c} a{\;}^{5}F_{2}—z{\;}^{5}P_{3}^{{}^{\circ}}\\ a{\;}^{1}D_{2}—34_{3}^{{}^{\circ}} \end{array}$
200	3188.340	31355.22	*a* ^5^F_3_—*y* ${\;}^{5}D_{4}^{{}^{\circ}}$
25	3188.609	31352.57	*b* ^3^F_3_—*x* ${\;}^{3}D_{2}^{{}^{\circ}}$
30	3188.916	31349.56	*a* ^3^F_3_—*y* ${\;}^{3}F_{3}^{{}^{\circ}}$
30	3189.297	31345.81	*a* ^5^F_5_—*z* ${\;}^{5}G_{4}^{{}^{\circ}}$
20	3189.726	31341.60	*a* ^3^G_5_—*y* ${\;}^{3}H_{6}^{{}^{\circ}}$
200	3189.979	31339.11	*a* ^5^F_3_—*y* ${\;}^{5}D_{3}^{{}^{\circ}}$
20	3191.778	31321.45	*a* ^3^P_1_—*y* ${\;}^{3}S_{1}^{{}^{\circ}}$
100	3192.068	31318.60	*b* ^3^F_4_— $1_{4}^{{}^{\circ}}$
40	3193.509	31304.47	*a* ^1^H_5_— $61_{4}^{{}^{\circ}}$
100	3194.746	31292.35	*a* ^3^G_4_—*y* ${\;}^{3}H_{5}^{{}^{\circ}}$
15	3195.317	31286.75	*b* ^3^H_4_— $63_{3}^{{}^{\circ}}$
1	3195.909	31280.96	*a* ^1^D_2_— $33_{2}^{{}^{\circ}}$
200	3196.606	31274.14	*a* ^5^F_1_—*y* ${\;}^{5}D_{0}^{{}^{\circ}}$
40	3198.329	31257.29	*a* ^3^P_2_—*v* ${\;}^{3}D_{3}^{{}^{\circ}}$
15	3199.119	21249.58	*a* ^3^F_2_—*y* ${\;}^{3}F_{2}^{{}^{\circ}}$
1	3199.528	31245.58	*b* ^3^P_2_— $5_{2}^{{}^{\circ}}$
70	3201.512	31226.22	*b* ^3^F_3_—*v* ${\;}^{3}D_{3}^{{}^{\circ}}$
3	3201.812	31223.29	*a* ^5^D_4_—*y* ${\;}^{5}P_{3}^{{}^{\circ}}$
40	3202.598	31215.63	*b* ^3^P_1_—*y* ${\;}^{5}S_{2}^{{}^{\circ}}$
75	3204.071	31201.28	*a* ^5^F_4_—*z* ${\;}^{3}F_{3}^{{}^{\circ}}$
50	3205.318	31189.14	*a* ^3^F_3_—*z* ${\;}^{3}H_{4}^{{}^{\circ}}$
2	3205.736	31185.07	*a* ^3^H_5_— $21_{5}^{{}^{\circ}}$
2	3206.491	31177.73	*a* ^5^D_2_—*y* ${\;}^{3}G_{3}^{{}^{\circ}}$
6	3207.635	31166.61	*a* ^5^D_3_—*y* ${\;}^{3}P_{2}^{{}^{\circ}}$
25	3208.419	31159.00	*a* ^3^G_4_—*x* ${\;}^{3}G_{3}^{{}^{\circ}}$
6	3208.732	31155.96	*b* ^3^F_4_—*y* ${\;}^{3}G_{4}^{{}^{\circ}}$
8	3208.813	31155.17	*b* ^3^P_2_—*x* ${\;}^{5}F_{3}^{{}^{\circ}}$
1	3209.303	31150.41	*b* ^3^P_0_— $8_{1}^{{}^{\circ}}$
3	3209.489	31148.61	*a* ^3^D_3_—*s* ${\;}^{3}D_{3}^{{}^{\circ}}$
30	3210.169	31142.01	*a* ^3^P_0_—*w* ${\;}^{3}D_{1}^{{}^{\circ}}$
3	3211.144	31132.56	*a* ^3^P_2_—*z* ${\;}^{1}D_{2}^{{}^{\circ}}$
100	3212.983	31114.74	*b* ^3^F_4_—*y* ${\;}^{3}G_{3}^{{}^{\circ}}$
50	3213.355	31111.13	*a* ^3^P_1_—*v* ${\;}^{3}D_{2}^{{}^{\circ}}$
50	3214.335	31101.65	$\{\;\begin{array}{c} b{\;}^{3}F_{3}—z{\;}^{1}D_{2}^{{}^{\circ}}\\ a{\;}^{3}G_{3}—x{\;}^{5}F_{3}^{{}^{\circ}} \end{array}$
50	3215.910	31086.42	*b* ^3^F_2_—*w* ${\;}^{3}D_{2}^{{}^{\circ}}$
100	3216.528	31080.45	*a* ^5^F_3_—*z* ${\;}^{3}F_{2}^{{}^{\circ}}$
5	3217.523	31070.84	*a* ^3^D_3_— $19_{4}^{{}^{\circ}}$
1	3218.920	31057.35	*a* ^3^D_3_—*w* ${\;}^{3}F_{2}^{{}^{\circ}}$
8	3219.148	31055.15	*a* ^1^G_4_—*w* ${\;}^{3}F_{4}^{{}^{\circ}}$
50	3220.081	31046.15	*a* ^3^G_4_—*w* ${\;}^{3}G_{4}^{{}^{\circ}}$
200	3223.279	31015.35	*a* ^5^F_2_—*y* ${\;}^{5}D_{2}^{{}^{\circ}}$
25	3224.658	31002.09	*a* ^3^D_2_— $9_{1}^{{}^{\circ}}$
100	3226.380	30985.54	*a* ^5^F_1_—*y* ${\;}^{5}D_{1}^{{}^{\circ}}$
75	3227.889	30971.06	*a* ^5^P_3_—*y* ${\;}^{3}P_{2}^{{}^{\circ}}$
40	3228.158	30968.48	*b* ^3^F_2_—*z* ${\;}^{1}P_{1}^{{}^{\circ}}$
150	3228.531	30964.90	*a* ^5^P_2_—*y* ${\;}^{5}P_{2}^{{}^{\circ}}$
2	3228.724	30963.05	*a* ^3^G_3_—*y* ${\;}^{3}H_{4}^{{}^{\circ}}$
50	3229.768	30953.04	*a* ^3^F_3_—*z* ${\;}^{1}G_{4}^{{}^{\circ}}$
30	3230.621	30944.87	*a* ^3^H_6_—*x* ${\;}^{1}H_{5}^{{}^{\circ}}$
80	3232.753	30924.46	*a* ^3^F_3_—*y* ${\;}^{5}P_{2}^{{}^{\circ}}$
5	3233.519	30917.14	*a* ^3^H_5_— $17_{4}^{{}^{\circ}}$
10	3234.428	30908.45	*a* ^3^G_3_—*x* ${\;}^{5}F_{4}^{{}^{\circ}}$
100	3238.537	30869.23	*a* ^3^D_2_—*t* ${\;}^{3}D_{2}^{{}^{\circ}}$
30	3238.780	30866.92	*a* ^5^F_2_—*z* ${\;}^{3}D_{1}^{{}^{\circ}}$
30	3239.596	30859.14	$\{\;\begin{array}{c} a{\;}^{3}P_{2}—w{\;}^{3}D_{3}^{{}^{\circ}}\\ a{\;}^{5}D_{2}—y{\;}^{3}P_{1}^{{}^{\circ}} \end{array}$
10	3239.686	30858.28	*a* ^5^D_3_—*y* ${\;}^{3}F_{3}^{{}^{\circ}}$
1	3239.928	30855.98	*a* ^3^D_3_— $16_{3}^{{}^{\circ}}$
100	3241.236	30843.53	*a* ^5^D_1_—*y* ${\;}^{3}P_{1}^{{}^{\circ}}$
1*h*	3241.786	30838.29	*b* ^1^D_1_— $63_{3}^{{}^{\circ}}$
40	3242.159	30834.75	*a* ^3^P_1_—*z* ${\;}^{1}S_{0}^{{}^{\circ}}$
25	3242.850	30828.18	$\{\;\begin{array}{c} a{\;}^{1}G_{4}—x{\;}^{1}G_{4}^{{}^{\circ}}\\ b{\;}^{3}F_{3}—w{\;}^{3}D_{3}^{{}^{\circ}} \end{array}$
100	3243.506	30821.94	*a* ^3^F_4_—*x* ${\;}^{5}D_{3}^{{}^{\circ}}$
10	3244.334	30814.08	*a* ^5^D_4_—*z* ${\;}^{3}H_{5}^{{}^{\circ}}$
15	3244.456	30812.92	*a* ^5^P_1_—*y* ${\;}^{3}F_{2}^{{}^{\circ}}$
2	3246.257	30795.82	*b* ^3^P_2_— $4_{3}^{{}^{\circ}}$
15	3248.841	30771.33	$\{\;\begin{array}{c} a{\;}^{3}G_{5}—z{\;}^{3}I_{5}^{{}^{\circ}}\\ a{\;}^{3}H_{4}—28_{3}^{{}^{\circ}} \end{array}$
50	3250.002	30760.34	*a* ^5^D_4_—*y* ${\;}^{3}F_{4}^{{}^{\circ}}$
5	3250.458	30756.02	*a* ^3^D_3_—*w* ${\;}^{3}F_{3}^{{}^{\circ}}$
50	3251.327	30747.80	*a* ^3^P_1_—*w* ${\;}^{3}D_{2}^{{}^{\circ}}$
10	3251.893	30742.45	*a* ^3^G_3_— $4_{3}^{{}^{\circ}}$
10	3252.540	30736.34	*a* ^3^D_2_—*w* ${\;}^{5}D_{2}^{{}^{\circ}}$
10	3252.898	30732.96	*a* ^3^F_2_—*y* ${\;}^{3}P_{1}^{{}^{\circ}}$
15	3252.993	30732.06	*a* ^3^G_5_—*x* ${\;}^{3}G_{4}^{{}^{\circ}}$
80	3254.539	30717.46	*a* ^5^F_2_—*y* ${\;}^{5}D_{3}^{{}^{\circ}}$
80	3254.707	30715.87	*a* ^5^D_2_—*y* ${\;}^{5}P_{1}^{{}^{\circ}}$
75	3256.334	30700.53	*a* ^5^D_1_—*y* ${\;}^{5}P_{1}^{{}^{\circ}}$
5	3256.611	30697.92	*a* ^5^D_3_—*z* ${\;}^{3}H_{4}^{{}^{\circ}}$
75	3258.042	30684.43	*a* ^5^D_2_—*y* ${\;}^{3}P_{2}^{{}^{\circ}}$
100	3259.677	30669.04	*a* ^5^D_1_—*y* ${\;}^{3}P_{2}^{{}^{\circ}}$
10	3260.167	30664.43	*a* ^1^G_4_—*y* ${\;}^{1}G_{4}^{{}^{\circ}}$
400	3260.367	30662.55	*a* ^5^F_4_—*z* ${\;}^{3}G_{3}^{{}^{\circ}}$
60	3261.128	30655.40	*b* ^3^P_2_— $3_{1}^{{}^{\circ}}$
5	3263.131	30636.58	*a* ^3^P_2_—*x* ${\;}^{3}F_{3}^{{}^{\circ}}$
100	3263.856	30629.78	*a* ^3^P_1_—*z* ${\;}^{1}P_{1}^{{}^{\circ}}$
100	3264.561	30623.16	*a* ^5^F_1_—*y* ${\;}^{5}D_{2}^{{}^{\circ}}$
100	3264.663	30622.21	*a* ^3^F_3_—*y* ${\;}^{5}P_{3}^{{}^{\circ}}$
2	3265.649	30612.96	*a* ^3^D_3_—*x* ${\;}^{5}P_{2}^{{}^{\circ}}$
125	3266.453	30605.42	*b* ^3^F_3_—*x* ${\;}^{3}F_{3}^{{}^{\circ}}$
200	3268.214	30588.93	*b* ^3^P_2_—*x* ${\;}^{3}P_{2}^{{}^{\circ}}$
2	3271.490	30558.31	*a* ^3^F_2_—*y* ${\;}^{3}P_{2}^{{}^{\circ}}$
2	3272.806	30546.02	*a* ^3^H_4_— $24_{5}^{{}^{\circ}}$
200	3273.082	30543.44	*a* ^5^P_2_—*z* ${\;}^{3}S_{1}^{{}^{\circ}}$
3	3273.612	30538.50	$\left\{ \begin{array}{l} \;a{\;}^{3}H_{4}—23_{3}^{{}^{\circ}}\\ \;a{\;}^{3}D_{2}—x{\;}^{5}F_{2}^{{}^{\circ}} \end{array}\right.$
200	3274.691	30528.43	*b* ^3^F_3_—*x* ${\;}^{3}F_{2}^{{}^{\circ}}$
125	3277.564	30501.67	*a* ^1^G_4_—*x* ${\;}^{1}F_{3}^{{}^{\circ}}$
100	3280.462	30474.73	*a* ^5^F_1_—*z* ${\;}^{3}D_{1}^{{}^{\circ}}$
8	3281.593	30464.23	*a*^3^D_1_— $18_{0}^{{}^{\circ}}$
75	3281.861	30461.74	*a* ^5^D_3_—*z* ${\;}^{1}G_{4}^{{}^{\circ}}$
10	3282.605	30454.84	*a* ^3^H_5_— $14_{4}^{{}^{\circ}}$
150	3284.935	30433.24	*a* ^5^D_3_—*y* ${\;}^{5}P_{2}^{{}^{\circ}}$
25	3285.377	30429.14	*a* ^3^H_4_— $22_{3}^{{}^{\circ}}$
25	3285.915	30424.16	*a* ^5^D_0_—*y* ${\;}^{3}P_{1}^{{}^{\circ}}$
100*h*	3289.244	30393.37	*a* ^3^P_2_—*x* ${\;}^{3}D_{1}^{{}^{\circ}}$
75	3291.119	30376.05	*a* ^5^D_2_—*y* ${\;}^{3}F_{3}^{{}^{\circ}}$
60	3291.657	30371.09	*a* ^1^G_4_—*w* ${\;}^{5}D_{3}^{{}^{\circ}}$
500	3294.109	30348.48	*a* ^5^F_5_—*z* ${\;}^{3}F_{4}^{{}^{\circ}}$
100	3296.116	30330.00	$\left\{ \begin{array}{l} \;b{\;}^{3}F_{4}—y{\;}^{3}G_{5}^{{}^{\circ}}\\ \;b{\;}^{3}P_{2}—x{\;}^{3}G_{3}^{{}^{\circ}} \end{array}\right.$
80	3296.645	30325.14	*a* ^3^P_2_—*z* ${\;}^{1}F_{3}^{{}^{\circ}}$
80	3297.259	30319.49	*b* ^3^P_1_— $3_{1}^{{}^{\circ}}$
100	3297.960	30313.05	*b* ^3^F_4_—*y* ${\;}^{3}F_{3}^{{}^{\circ}}$
30	3298.408	30308.93	*b* ^3^F_2_—*z* ${\;}^{1}D_{2}^{{}^{\circ}}$
100	3299.336	30300.40	*a* ^5^F_3_—*z* ${\;}^{3}F_{3}^{{}^{\circ}}$
8	3299.788	30296.26	*a* ^5^P_1_—*y* ${\;}^{3}P_{1}^{{}^{\circ}}$
500	3301.593	30279.69	*a* ^5^F5—*z* ${\;}^{5}G_{5}^{{}^{\circ}}$
75	3301.911	30276.78	*a* ^3^G_3_—*x* ${\;}^{3}G_{3}^{{}^{\circ}}$
5	3302.182	30274.29	*a* ^5^P_1_—*y* ${\;}^{3}P_{0}^{{}^{\circ}}$
100	3303.998	30257.65	*b* ^3^P_2_—*y* ${\;}^{1}D_{2}^{{}^{\circ}}$
1	3304.288	30255.00	*a* ^3^H_5_—*x* ${\;}^{1}H_{5}^{{}^{\circ}}$
40	3304.502	30253.04	*b* ^3^P_1_—*x* ${\;}^{3}P_{2}^{{}^{\circ}}$
40	3304.637	30251.80	*a* ^5^F_3_—*z* ${\;}^{7}P_{2}^{{}^{\circ}}$
50	3304.789	30250.41	*a* ^3^F_2_—*y* ${\;}^{3}F_{3}^{{}^{\circ}}$
20	3304.820	30250.13	*a* ^5^F_5_—*z* ${\;}^{7}P_{4}^{{}^{\circ}}$
1	3305.653	30242.50	*a* ^3^H_4_—*s* ${\;}^{3}D_{3}^{{}^{\circ}}$
300	3306.179	30237.69	*a* ^5^P_3_—*y* ${\;}^{5}P_{2}^{{}^{\circ}}$
75	3307.985	30221.18	*a* ^3^P_1_—*x* ${\;}^{3}D_{2}^{{}^{\circ}}$
50	3308.624	30215.35	*a* ^3^F_4_—*y* ${\;}^{3}D_{3}^{{}^{\circ}}$
10	3309.834	30204.30	*a* ^3^G_3_—*y* ${\;}^{1}D_{2}^{{}^{\circ}}$
8	3310.082	30202.04	*a* ^1^D_2_—*w* ${\;}^{3}F_{2}^{{}^{\circ}}$
75	3310.958	30194.05	*a* ^5^F_4_—*z* ${\;}^{7}P_{3}^{{}^{\circ}}$
2	3312.313	30181.70	*a* ^3^G_4_—*y* ${\;}^{1}F_{3}^{{}^{\circ}}$
2	3314.512	30161.67	*a* ^3^G_4_—*z* ${\;}^{3}I_{5}^{{}^{\circ}}$
20	3314.770	30159.33	*a* ^3^F_3_—*y* ${\;}^{3}F_{4}^{{}^{\circ}}$
40	3315.052	30156.76	*a* ^5^P_2_—*x* ${\;}^{5}D_{1}^{{}^{\circ}}$
80	3315.255	30154.91	*a* ^5^F_4_—*z* ${\;}^{5}G_{4}^{{}^{\circ}}$
20	3315.438	30153.25	*a* ^5^P_1_—*y* ${\;}^{5}P_{1}^{{}^{\circ}}$
2	3315.994	30148.19	*a* ^3^D_2_—*x* ${\;}^{1}F_{3}^{{}^{\circ}}$
150	3316.380	30144.68	*a* ^3^P_2_—*x* ${\;}^{3}D_{3}^{{}^{\circ}}$
20	3316.908	30139.89	*a* ^3^G_5_—*x* ${\;}^{3}F_{4}^{{}^{\circ}}$
100	3317.883	30131.03	*a* ^5^D_3_—*y* ${\;}^{5}P_{3}^{{}^{\circ}}$
40	3318.821	30122.51	*a* ^3^G_4_—*x* ${\;}^{3}G_{4}^{{}^{\circ}}$
40	3318.900	30121.80	*a* ^5^P_1_—*y* ${\;}^{3}P_{2}^{{}^{\circ}}$
60	3319.527	30116.11	*a* ^5^F_3_—*z* ${\;}^{3}D_{2}^{{}^{\circ}}$
25	3319.801	30113.62	*b* ^3^F_3_—*x* ${\;}^{3}D_{3}^{{}^{\circ}}$
60	3321.248	30100.50	*a* ^1^G_4_—*x* ${\;}^{5}F_{3}^{{}^{\circ}}$
5*h*	3321.644	30096.92	*a* ^1^P_1_— $58_{1}^{{}^{\circ}}$
1	3323.213	30082.71	*a* ^3^D_3_— $12_{4}^{{}^{\circ}}$
100	3324.999	30066.55	$\left\{ \begin{array}{l} \;a{\;}^{5}F_{1}—z{\;}^{3}F_{2}^{{}^{\circ}}\\ \;a{\;}^{1}H_{5}—v{\;}^{3}G_{5}^{{}^{\circ}} \end{array}\right.$
25	3328.456	30035.32	*b* ^3^F_2_—*w* ${\;}^{3}D_{3}^{{}^{\circ}}$
125	3332.641	29997.61	*a* ^3^F_4_—*x* ${\;}^{5}D_{4}^{{}^{\circ}}$
150	3335.689	29970.20	*a* ^3^P_1_—*z* ${\;}^{1}D_{2}^{{}^{\circ}}$
60	3336.631	29961.73	*a* ^1^G_4_—*y* ${\;}^{3}H_{4}^{{}^{\circ}}$
60	3337.829	29950.98	*a* ^5^D_2_—*y* ${\;}^{5}P_{2}^{{}^{\circ}}$
500	3339.555	29935.50	$\begin{array}{l} \left\{ \begin{array}{l} \;a{\;}^{5}P_{3}—y{\;}^{5}P_{3}^{{}^{\circ}}\\ \;a{\;}^{5}D_{1}—y{\;}^{5}P_{2}^{{}^{\circ}} \end{array}\right. \end{array}$
60	3341.090	29921.75	*b* ^3^P_1_—*y* ${\;}^{1}D_{2}^{{}^{\circ}}$
150	3341.665	29916.60	*b* ^3^F_4_—*z* ${\;}^{1}G_{4}^{{}^{\circ}}$
6	3342.709	29907.26	*a* ^1^G_4_—*x* ${\;}^{5}F_{4}^{{}^{\circ}}$
1	3343.431	29900.80	*a* ^1^D_2_—*w* ${\;}^{3}F_{3}^{{}^{\circ}}$
1	3344.266	29893.33	*a* ^1^H_5_—*v* ${\;}^{3}G_{4}^{{}^{\circ}}$
75	3344.535	29890.93	*a* ^5^F_5_—*z* ${\;}^{3}G_{4}^{{}^{\circ}}$
75	3345.316	29883.95	*a* ^5^D_4_—*x* ${\;}^{5}D_{3}^{{}^{\circ}}$
100	3347.617	29863.41	*b* ^3^P_2_—*u* ${\;}^{3}D_{2}^{{}^{\circ}}$
50	3348.017	29859.84	*b* ^3^P_1_—*u* ${\;}^{3}D_{1}^{{}^{\circ}}$
75	3348.704	29853.72	*a* ^5^F_4_—*z* ${\;}^{3}D_{3}^{{}^{\circ}}$
30	3350.550	29837.27	*a* ^3^D_2_— $5_{2}^{{}^{\circ}}$
2	3351.695	29827.08	*a* ^3^H_5_— $10_{4}^{{}^{\circ}}$
100	3351.930	29824.99	*a* ^3^F_2_—*y* ${\;}^{5}P_{2}^{{}^{\circ}}$
10	3352.970	29815.74	*a* ^3^D_1_—*y* ${\;}^{1}P_{1}^{{}^{\circ}}$
60	3353.310	29812.71	*b* ^3^F_2_—*x* ${\;}^{3}F_{3}^{{}^{\circ}}$
100	3353.648	29809.71	*a* ^3^P_2_—*y* ${\;}^{3}F_{2}^{{}^{\circ}}$
50	3356.201	29787.03	*a* ^3^D_1_—*t* ${\;}^{3}D_{1}^{{}^{\circ}}$
25	3356.476	29784.59	*b* ^3^F_3_— $1_{4}^{{}^{\circ}}$
3	3357.15	29778.61	*b* ^3^F_3_—*y* ${\;}^{3}F_{2}^{{}^{\circ}}$
200	3359.095	29761.37	*a* ^5^F_3_—*z* ${\;}^{3}G_{3}^{{}^{\circ}}$
1	3359.507	29757.72	*a* ^1^D_2_—*x* ${\;}^{5}P_{2}^{{}^{\circ}}$
50	3361.145	29743.22	*a* ^1^D_2_— $15_{1}^{{}^{\circ}}$
100	3362.007	29735.60	*b* ^3^F_2_—*x* ${\;}^{3}F_{2}^{{}^{\circ}}$
50	3362.337	29732.68	*a* ^3^D_3_—*t* ${\;}^{3}D_{2}^{{}^{\circ}}$
60	3364.100	29717.10	*c* ^3^F_4_— $61_{4}^{{}^{\circ}}$
250	3368.455	29678.67	*a* ^5^F_2_—*z* ${\;}^{3}F_{3}^{{}^{\circ}}$
50	3369.674	29667.94	*a* ^5^D_3_—*y* ${\;}^{3}F_{4}^{{}^{\circ}}$
125	3371.863	29648.68	*a* ^5^D_2_—*y* ${\;}^{5}P_{3}^{{}^{\circ}}$
75	3373.986	29630.03	*a* ^5^F_2_—*z* ${\;}^{7}P_{2}^{{}^{\circ}}$
125	3374.656	29624.14	*a* ^5^P_2_—*x* ${\;}^{5}D_{2}^{{}^{\circ}}$
50	3374.901	29621.99	*b* ^3^F_3_—*y* ${\;}^{3}G_{4}^{{}^{\circ}}$
15	3376.058	29611.84	*a* ^3^P_2_—*y* ${\;}^{3}G_{3}^{{}^{\circ}}$
1	3377.633	29598.03	*a* ^3^H_4_— $14_{4}^{{}^{\circ}}$
125	3378.034	29594.52	*a* ^5^F_5_—*z* ${\;}^{7}F_{4}^{{}^{\circ}}$
25	3379.033	29585.77	*b* ^3^F_4_—*y* ${\;}^{5}P_{3}^{{}^{\circ}}$
40	3379.267	29583.72	*a* ^3^F_3_—*x* ${\;}^{5}D_{2}^{{}^{\circ}}$
100	3379.608	29580.74	*b* ^3^F_3_—*y* ${\;}^{3}G_{3}^{{}^{\circ}}$
100	3380.173	29575.79	*a* ^5^P_2_—*y* ${\;}^{3}D_{1}^{{}^{\circ}}$
50	3380.916	29569.29	*b* ^3^F_2_—*x* ${\;}^{3}D_{1}^{{}^{\circ}}$
125	3385.161	29532.21	*a* ^3^G_5_—*x* ${\;}^{3}G_{5}^{{}^{\circ}}$
2	3385.403	29530.10	*a* ^3^G_4_—*x* ${\;}^{3}F_{4}^{{}^{\circ}}$
30	3385.480	29529.43	*a* ^5^D_2_—*z* ${\;}^{3}S_{1}^{{}^{\circ}}$
50	3385.704	29527.48	*b* ^3^P_1_—*u* ${\;}^{3}D_{2}^{{}^{\circ}}$
50	3386.254	29522.68	*a* ^3^F_2_—*y* ${\;}^{5}P_{3}^{{}^{\circ}}$
50	3387.232	29514.16	*a* ^5^D_1_—*z* ${\;}^{3}S_{1}^{{}^{\circ}}$
200	3388.710	29501.29	*b* ^3^F_2_—*z* ${\;}^{1}F_{3}^{{}^{\circ}}$
150	3389.503	29494.38	*a* ^5^F_2_—*z* ${\;}^{3}D_{2}^{{}^{\circ}}$
16	3390.899	29482.24	*a* ^1^D_2_—*y* ${\;}^{1}P_{1}^{{}^{\circ}}$
60	3391.895	29473.58	*b* ^3^P_0_— $3_{1}^{{}^{\circ}}$
2	3392.027	29472.44	*a* ^5^P_3_—*y* ${\;}^{3}F_{4}^{{}^{\circ}}$
300	3392.533	29468.04	*a* ^5^F_5_—*z* ${\;}^{7}F_{5}^{{}^{\circ}}$
2	3393.259	29461.74	*b* ^3^P_2_—*y* ${\;}^{3}S_{1}^{{}^{\circ}}$
50	3399.383	29408.66	*a* ^1^G_4_—*y* ${\;}^{3}H_{5}^{{}^{\circ}}$
4	3399.983	29403.47	*a* ^3^F_2_—*z* ${\;}^{3}S_{1}^{{}^{\circ}}$
4	3400.594	29398.19	*a* ^3^H_4_—*x* ${\;}^{1}H_{5}^{{}^{\circ}}$
20	3400.750	29396.84	*a* ^3^P_1_—*x* ${\;}^{3}F_{2}^{{}^{\circ}}$
20	3401.508	29390.29	*a* ^3^D_1_— $11_{0}^{{}^{\circ}}$
500	3401.740	29388.29	*a* ^5^P_1_—*y* ${\;}^{5}P_{2}^{{}^{\circ}}$
6	3403.779	29370.69	*a* ^3^G_5_—*z* ${\;}^{3}I_{6}^{{}^{\circ}}$
3	3405.147	29358.89	*a* ^3^D_3_— $7_{3}^{{}^{\circ}}$
50	3405.885	29352.52	*b* ^3^P_2_—*y* ${\;}^{1}F_{3}^{{}^{\circ}}$
60	3406.598	29346.38	*a* ^5^F_4_—*z* ${\;}^{5}G_{3}^{{}^{\circ}}$
2	3406.914	29343.66	*a* ^3^D_1_— $9_{1}^{{}^{\circ}}$
1*h*	3407.581	29337.91	*a* ^3^D_3_—*x* ${\;}^{1}G_{4}^{{}^{\circ}}$
400	3409.279	29323.30	*a* ^5^P_2_—*x* ${\;}^{5}D_{3}^{{}^{\circ}}$
20	3409.573	29320.77	*b* ^3^F_2_—*x* ${\;}^{3}D_{3}^{{}^{\circ}}$
300	3411.637	29303.04	*a* ^5^P_2_—*z* ${\;}^{3}P_{1}^{{}^{\circ}}$
10	3412.076	29299.27	*a* ^3^G_3_—*y* ${\;}^{1}F_{3}^{{}^{\circ}}$
100	3412.799	29293.06	$\left\{ \begin{array}{l} \;a{\;}^{5}F_{3}—z{\;}^{7}P_{3}^{{}^{\circ}}\\ \;a{\;}^{3}P_{2}—y{\;}^{3}P_{1}^{{}^{\circ}} \end{array}\right.$
4	3413.719	29285.16	*a* ^3^D_3_— $6_{3}^{{}^{\circ}}$
4	3413.983	29282.90	*a* ^3^F_3_—*x* ${\;}^{5}D_{3}^{{}^{\circ}}$
100	3414.641	29277.26	*a* ^5^D_4_—*y* ${\;}^{3}D_{3}^{{}^{\circ}}$
6	3414.843	29275.53	*a* ^1^G_4_—*x* ${\;}^{3}G_{3}^{{}^{\circ}}$
80	3416.188	29264.00	*a* ^3^P_0_—*x* ${\;}^{3}D_{1}^{{}^{\circ}}$
1000*R*	3417.332	29254.20	*a* ^5^F_3_—*z* ${\;}^{5}G_{4}^{{}^{\circ}}$
20	3417.648	29251.50	*b* ^3^P_2_—*v* ${\;}^{3}D_{2}^{{}^{\circ}}$
50	3417.975	29248.70	*b* ^3^P_2_—*w* ${\;}^{3}D_{1}^{{}^{\circ}}$
2	3418.154	29247.17	*a* ^3^D_2_— $3_{1}^{{}^{\circ}}$
50	3419.248	29237.81	*a* ^5^F_1_—*z* ${\;}^{7}P_{2}^{{}^{\circ}}$
300	3420.090	29230.61	*a* ^3^P_1_—*x* ${\;}^{3}D_{1}^{{}^{\circ}}$
8	3422.414	29210.77	*a* ^3^D_1_—*t* ${\;}^{3}D_{2}^{{}^{\circ}}$
30	3425.952	29180.60	*a* ^3^D_2_—*x* ${\;}^{3}P_{2}^{{}^{\circ}}$
10	3426.434	29176.50	$\left\{ \begin{array}{l} \;b{\;}^{3}F_{4}—z{\;}^{3}H_{5}^{{}^{\circ}}\\ \;a{\;}^{3}H_{4}—12_{4}^{{}^{\circ}} \end{array}\right.$
1500*R*	3428.319	29160.45	*a* ^5^F_5_—*z* ${\;}^{7}F_{6}^{{}^{\circ}}$
80	3428.628	29157.83	*a* ^5^F_4_—*z* ${\;}^{3}F_{4}^{{}^{\circ}}$
200	3429.547	29150.01	*a* ^3^P_2_—*y* ${\;}^{5}P_{1}^{{}^{\circ}}$
2	3430.387	29142.88	*a* ^5^D_2_—*x* ${\;}^{5}D_{1}^{{}^{\circ}}$
500	3430.764	29139.67	*a* ^5^F_2_—*z* ${\;}^{3}G_{3}^{{}^{\circ}}$
150	3432.204	29127.45	*a* ^5^D_1_—*x* ${\;}^{5}D_{1}^{{}^{\circ}}$
400	3432.755	29122.77	*b* ^3^F_4_—*y* ${\;}^{3}F_{4}^{{}^{\circ}}$
600	3433.255	29118.53	*a* ^3^P_2_—*y* ${\;}^{3}P_{2}^{{}^{\circ}}$
200	3435.183	29102.19	*a* ^5^F_1_—*z* ${\;}^{3}D_{2}^{{}^{\circ}}$
20	3436.330	29092.47	*a* ^5^D_3_—*x* ${\;}^{5}D_{2}^{{}^{\circ}}$
3000*R*	3436.737	29089.03	*a* ^5^F_4_—*z* ${\;}^{5}G_{5}^{{}^{\circ}}$
400	3438.369	29075.22	*a* ^5^P_2_—*z* ${\;}^{3}P_{2}^{{}^{\circ}}$
500	3440.207	29059.69	$\left\{ \begin{array}{l} \;a{\;}^{5}D_{4}—x{\;}^{5}D_{4}^{{}^{\circ}}\\ \;a{\;}^{5}F_{4}—z{\;}^{7}P_{4}^{{}^{\circ}} \end{array}\right.$
40	3443.153	29034.83	*a* ^3^F_3_—*z* ${\;}^{3}P_{2}^{{}^{\circ}}$
20	3445.296	29016.77	*a* ^3^F_2_—*x* ${\;}^{5}D_{1}^{{}^{\circ}}$
6	3445.939	29011.35	*a* ^3^D_3_—*x* ${\;}^{1}F_{3}^{{}^{\circ}}$
50	3446.070	29010.25	*a* ^1^D_2_— $9_{1}^{{}^{\circ}}$
50	3446.490	29006.71	*a* ^3^D_3_—*y* ${\;}^{5}S_{2}^{{}^{\circ}}$
500	3448.959	28985.95	*b* ^3^F_2_—*y* ${\;}^{3}F_{2}^{{}^{\circ}}$
1	3450.821	28970.31	*a* ^3^H_4_— $10_{4}^{{}^{\circ}}$
1	3451.224	28966.93	*a* ^5^P_1_—*z* ${\;}^{3}S_{1}^{{}^{\circ}}$
400	3452.906	28952.82	*a* ^5^F_3_—*z* ${\;}^{3}D_{3}^{{}^{\circ}}$
30	3455.385	28932.05	*a* ^1^D_2_— $8_{1}^{{}^{\circ}}$
200	3456.621	28921.70	*a* ^5^P_2_—*y* ${\;}^{3}D_{2}^{{}^{\circ}}$
2	3457.688	28912.78	*b* ^3^P_1_—*w* ${\;}^{3}D_{1}^{{}^{\circ}}$
40	3459.585	28896.93	*a* ^5^P_3_—*x* ${\;}^{5}D_{2}^{{}^{\circ}}$
2	3460.647	28888.06	*b* ^3^P_2_—*w* ${\;}^{3}D_{2}^{{}^{\circ}}$
60	3461.930	28877.35	*a* ^1^D_2_—*t* ${\;}^{3}D_{2}^{{}^{\circ}}$
300	3463.143	28867.24	*a* ^5^F_3_—*z* ${\;}^{5}G_{2}^{{}^{\circ}}$
4	3465.292	28849.33	*a* ^3^D_2_—*y* ${\;}^{1}D_{2}^{{}^{\circ}}$
75	3467.051	28834.70	*a* ^3^G_3_—*w* ${\;}^{3}D_{2}^{{}^{\circ}}$
1	3467.876	28827.84	*c* ^3^P_2_— $53_{2}^{{}^{\circ}}$
2	3470.001	28810.18	*a* ^3^P_2_—*y* ${\;}^{3}F_{3}^{{}^{\circ}}$
150	3472.244	28791.58	*a* ^5^D_3_—*x* ${\;}^{5}D_{3}^{{}^{\circ}}$
20	3472.669	28788.05	*b* ^3^F_2_—*y* ${\;}^{3}G_{3}^{{}^{\circ}}$
10	3472.739	28787.47	*a* ^3^D_2_—*u* ${\;}^{3}D_{1}^{{}^{\circ}}$
700	3473.752	28779.08	*b* ^3^F_3_—*y* ${\;}^{3}F_{3}^{{}^{\circ}}$
100	3474.845	28770.03	*b* ^3^P_2_—*z* ${\;}^{1}P_{1}^{{}^{\circ}}$
2	3479.794	28729.11	*a* ^5^D_1_—*x* ${\;}^{5}D_{0}^{{}^{\circ}}$
600	3481.308	28716.61	*a* ^5^P_2_—*y* ${\;}^{3}D_{3}^{{}^{\circ}}$
80	3482.347	28708.05	*a* ^5^D_0_—*x* ${\;}^{5}D_{1}^{{}^{\circ}}$
200	3483.174	28701.23	*a* ^5^F_4_—*z* ${\;}^{7}F_{3}^{{}^{\circ}}$
200	3483.294	28700.24	*a* ^5^F_4_—*z* ${\;}^{3}G_{4}^{{}^{\circ}}$
50	3486.211	28676.23	*a* ^3^F_3_—*y* ${\;}^{3}D_{3}^{{}^{\circ}}$
50	3486.799	28671.39	*a* ^5^F_2_—*z* ${\;}^{7}P_{3}^{{}^{\circ}}$
4	3487.461	28665.95	*a* ^3^G_4_—*w* ${\;}^{3}D_{3}^{{}^{\circ}}$
15	3489.750	28647.15	*a* ^3^P_1_—*y* ${\;}^{3}F_{2}^{{}^{\circ}}$
20	3490.729	28639.12	*b* ^3^P_1_—*z* ${\;}^{1}S_{0}^{{}^{\circ}}$
50	3493.227	28618.64	*b* ^3^F_3_—*z* ${\;}^{3}H_{4}^{{}^{\circ}}$
150	3494.254	28610.22	*a* ^5^D_2_—*x* ${\;}^{5}D_{2}^{{}^{\circ}}$
150	3495.985	28596.06	*a* ^5^P_3_—*x* ${\;}^{5}D_{3}^{{}^{\circ}}$
100	3496.130	28594.88	*a* ^5^D_1_—*x* ${\;}^{5}D_{2}^{{}^{\circ}}$
100	3497.935	28580.12	*a* ^5^P_1_—*x* ${\;}^{5}D_{1}^{{}^{\circ}}$
6000*R*	3498.944	28571.88	*a* ^5^F_5_—*z* ${\;}^{5}G_{6}^{{}^{\circ}}$
2	3500.528	28558.95	*a* ^3^H_6_—*y* ${\;}^{3}H_{5}^{{}^{\circ}}$
60	3501.365	28552.12	*b* ^3^P_1_—*w* ${\;}^{3}D_{2}^{{}^{\circ}}$
2	3502.039	28546.63	*a* ^5^D_1_—*y* ${\;}^{3}D_{1}^{{}^{\circ}}$
80	3502.423	28543.51	*a* ^5^D_3_—*z* ${\;}^{3}P_{2}^{{}^{\circ}}$
**3***h*	3507.327	28503.59	*a* ^1^D_2_— $7_{3}^{{}^{\circ}}$
2	3508.371	28495.11	*a* ^5^F_5_—*z* ${\;}^{3}G_{5}^{{}^{\circ}}$
100	3509.716	28484.19	*a* ^3^F_2_—*x* ${\;}^{5}D_{2}^{{}^{\circ}}$
4	3510.314	28479.34	$\left\{ \begin{array}{l} \;c{\;}^{3}F_{4}—v{\;}^{3}G_{5}^{{}^{\circ}}\\ \;c{\;}^{3}P_{1}—60_{1}^{{}^{\circ}} \end{array}\right.$
20	3511.131	28472.71	*a* ^5^F_2_—*z* ${\;}^{5}S_{2}^{{}^{\circ}}$
10	3511.561	28469.22	*b* ^3^F_2_—*y* ${\;}^{3}P_{1}^{{}^{\circ}}$
40	3512.885	28458.49	*a* ^3^F_3_—*x* ${\;}^{5}D_{4}^{{}^{\circ}}$
2	3513.299	28455.14	*a* ^3^D_2_—*u* ${\;}^{3}D_{2}^{{}^{\circ}}$
5	3514.12	28448.49	*a* ^3^H_6_— $2_{6}^{{}^{\circ}}$
1000	3514.491	28445.49	*a* ^3^F_3_—*z* ${\;}^{5}G_{3}^{{}^{\circ}}$
60	3514.757	28443.33	*a* ^3^G_4_—*x* ${\;}^{3}F_{3}^{{}^{\circ}}$
40	3515.682	28435.85	*a* ^3^F_2_—*y* ${\;}^{3}D_{1}^{{}^{\circ}}$
40	3515.897	28434.11	*b* ^3^P_1_—*z* ${\;}^{1}P_{1}^{{}^{\circ}}$
6	3516.194	28431.71	*a* ^3^H_4_—*x* ${\;}^{1}G_{4}^{{}^{\circ}}$
20	3517.417	28421.82	*a* ^3^H_5_—*y* ${\;}^{3}H_{4}^{{}^{\circ}}$
2	3518.009	28417.04	*a* ^3^D_3_—*x* ${\;}^{5}F_{4}^{{}^{\circ}}$
60	3518.991	28409.11	*a* ^3^G_5_—*z* ${\;}^{1}H_{5}^{{}^{\circ}}$
500	3519.641	28403.87	*a* ^5^F_4_—*z* ${\;}^{7}F_{4}^{{}^{\circ}}$
300	3520.134	28399.89	*a* ^3^D_1_—*z* ${\;}^{3}P_{0}^{{}^{\circ}}$
40	3521.972	28385.07	*a* ^3^P_2_—*y* ${\;}^{5}P_{2}^{{}^{\circ}}$
40	3522.284	28382.56	*b* ^3^F_3_—*z* ${\;}^{1}G_{4}^{{}^{\circ}}$
4	3524.161	28367.44	*a* ^3^H_5_—*x* ${\;}^{5}F_{4}^{{}^{\circ}}$
50	3524.905	28361.45	*b* ^3^P_2_—*x* ${\;}^{3}D_{2}^{{}^{\circ}}$
10	3525.657	28355.40	*b* ^3^H_4_— $61_{4}^{{}^{\circ}}$
10	3525.829	28354.02	*b* ^3^F_3_—*y* ${\;}^{5}P_{2}^{{}^{\circ}}$
80	3526.578	28348.00	*a* ^5^P_3_—*z* ${\;}^{3}P_{2}^{{}^{\circ}}$
250	3528.679	28331.12	*a* ^5^F_2_—*z* ${\;}^{3}D_{3}^{{}^{\circ}}$
50	3529.292	28326.20	*b* ^3^F_2_—*y* ${\;}^{5}P_{1}^{{}^{\circ}}$
2	3529.621	28323.56	*a* ^5^D_4_—*y* ${\;}^{5}F_{3}^{{}^{\circ}}$
200	3531.389	28309.38	*a* ^5^D_2_—*x* ${\;}^{5}D_{3}^{{}^{\circ}}$
10	3531.808	28306.02	*c* ^3^F_4_—*v* ${\;}^{3}G_{4}^{{}^{\circ}}$
300	3532.810	28297.99	*a* ^1^G_4_—*y* ${\;}^{1}F_{3}^{{}^{\circ}}$
25	3533.917	28289.13	*a* ^5^D_2_—*z* ${\;}^{3}P_{1}^{{}^{\circ}}$
50	3535.059	28279.99	*b* ^3^P_0_—*y* ${\;}^{3}S_{1}^{{}^{\circ}}$
60	3535.32	28277.90	*a* ^1^G_4_—*z* ${\;}^{3}I_{5}^{{}^{\circ}}$
200	3535.382	28277.41	*a* ^5^F_4_—*z* ${\;}^{7}F_{5}^{{}^{\circ}}$
200	3535.836	28273.77	*a* ^5^D_1_—*z* ${\;}^{3}P_{1}^{{}^{\circ}}$
60	3536.572	28267.89	*a* ^3^H_4_—*y* ${\;}^{1}G_{4}^{{}^{\circ}}$
400	3537.941	28256.95	*a* ^5^F_3_—*z* ${\;}^{3}F_{4}^{{}^{\circ}}$
200	3539.254	28246.47	*b* ^3^F_4_—*x* ${\;}^{5}D_{3}^{{}^{\circ}}$
400	3539.368	28245.56	*a* ^5^F_2_—*z* ${\;}^{5}G_{2}^{{}^{\circ}}$
50	3540.210	28238.84	*a* ^1^G_4_—*x* ${\;}^{3}G_{4}^{{}^{\circ}}$
2	3540.673	28235.15	*b* ^3^P_2_—*v* ${\;}^{3}D_{3}^{{}^{\circ}}$
50	3541.040	28232.22	*a* ^3^G_5_— $1_{4}^{{}^{\circ}}$
200	3541.622	28227.58	*a* ^3^F_4_—*y* ${\;}^{5}F_{5}^{{}^{\circ}}$
2	3545.713	28195.02	*a* ^5^P_2_—*y* ${\;}^{5}F_{1}^{{}^{\circ}}$
10	3545.797	28194.35	*a* ^5^P_3_—*y* ${\;}^{3}D_{2}^{{}^{\circ}}$
4	3546.014	28192.62	*a* ^3^H_6_—*w* ${\;}^{3}G_{5}^{{}^{\circ}}$
100	3546.982	28184.93	*a* ^5^D_3_—*y* ${\;}^{3}D_{3}^{{}^{\circ}}$
50	3547.184	28183.33	*a* ^3^F_2_—*x* ${\;}^{5}D_{3}^{{}^{\circ}}$
50	3547.380	28181.77	$\left\{ \begin{array}{l} \;a{\;}^{5}P_{1}—x{\;}^{5}D_{0}^{{}^{\circ}}\\ \;a{\;}^{3}G_{3}—v{\;}^{3}D_{3}^{{}^{\circ}} \end{array}\right.$
10	3547.760	28178.75	*a* ^3^D_1_— $5_{2}^{{}^{\circ}}$
6	3549.632	28163.89	*a* ^3^P_0_—*y* ${\;}^{3}P_{1}^{{}^{\circ}}$
30	3549.737	28163.06	*a* ^3^F_2_—*z* ${\;}^{3}P_{1}^{{}^{\circ}}$
200	3550.271	28158.82	*a* ^5^F_3_—*z* ${\;}^{7}P_{4}^{{}^{\circ}}$
50	3550.635	28155.93	*a* ^1^D_2_—*x* ${\;}^{1}F_{3}^{{}^{\circ}}$
10	3551.220	28151.29	*a* ^1^D_2_—*y* ${\;}^{5}S_{2}^{{}^{\circ}}$
20	3553.672	28131.87	*a* ^3^G_4_—*z* ${\;}^{1}F_{3}^{{}^{\circ}}$
200	3553.850	28130.46	*a* ^3^P_1_—*y* ${\;}^{3}P_{1}^{{}^{\circ}}$
10	3554.274	28127.11	*a* ^5^D_0_—*y* ${\;}^{3}D_{1}^{{}^{\circ}}$
2	3555.827	28114.82	*b* ^3^G_5_— $62_{4}^{{}^{\circ}}$
6	3556.393	28110.35	*b* ^3^P_2_—*z* ${\;}^{1}D_{2}^{{}^{\circ}}$
125	3556.634	28108.44	*a* ^3^P_1_—*y* ${\;}^{3}P_{0}^{{}^{\circ}}$
125	3557.062	28105.06	*a* ^3^H_4_—*x* ${\;}^{1}F_{3}^{{}^{\circ}}$
4	3559.643	28084.68	*a* ^1^P_1_— $33_{2}^{{}^{\circ}}$
50	3559.883	28082.79	*a* ^3^P_2_—*y* ${\;}^{5}P_{3}^{{}^{\circ}}$
20	3560.029	28081.64	*a* ^3^H_5_—*x* ${\;}^{5}F_{5}^{{}^{\circ}}$
30	3560.175	28080.49	*a* ^5^F_1_—*z* ${\;}^{5}S_{2}^{{}^{\circ}}$
40*H*	3561.570	28069.49	*a* ^3^G_5_—*y* ${\;}^{3}G_{4}^{{}^{\circ}}$
100*H*	3561.900	28066.89	*b* ^3^P_0_—*w* ${\;}^{3}D_{1}^{{}^{\circ}}$
60	3562.618	28061.23	*a* ^5^D_2_—*z* ${\;}^{3}P_{2}^{{}^{\circ}}$
20	3563.148	28057.06	*a* ^3^G_3_—*z* ${\;}^{1}D_{2}^{{}^{\circ}}$
20	3563.611	28053.41	*a* ^3^D_2_—*y* ${\;}^{3}S_{1}^{{}^{\circ}}$
10	3563.827	28051.71	*b* ^3^F_3_— *y* ${\;}^{5}P_{3}^{{}^{\circ}}$
100	3564.364	28047.49	*a* ^5^P_1_—*x* ${\;}^{5}D_{2}^{{}^{\circ}}$
30	3564.504	28046.38	$\left\{ \begin{array}{l} \;a{\;}^{3}H_{6}—y{\;}^{1}H_{5}^{{}^{\circ}}\\ \;c{\;}^{3}P_{1}—58_{1}^{{}^{\circ}} \end{array}\right.$
150	3564.570	28045.87	*a* ^5^D_1_—*z* ${\;}^{3}P_{2}^{{}^{\circ}}$
60	3564.809	28043.99	*a* ^3^D_3_—*x* ${\;}^{3}P_{2}^{{}^{\circ}}$
100	3567.157	28025.53	*b* ^3^P_1_—*x* ${\;}^{3}D_{2}^{{}^{\circ}}$
10	3567.751	28020.86	*a* ^3^P_0_—*y* ${\;}^{5}P_{1}^{{}^{\circ}}$
50	3568.527	28014.77	*a* ^5^F_5_—*z* ${\;}^{5}F_{4}^{{}^{\circ}}$
1000	3570.606	27998.46	*a* ^3^H_6_—*y* ${\;}^{3}H_{6}^{{}^{\circ}}$
60	3571.767	27989.36	*a* ^5^P_3_—*y* ${\;}^{3}D_{3}^{{}^{\circ}}$
150	3572.022	27987.36	*a* ^3^P_1_—*y* ${\;}^{5}P_{1}^{{}^{\circ}}$
10	3573.666	27974.48	*a* ^3^H_4_—*w* ${\;}^{5}D_{3}^{{}^{\circ}}$
200	3574.601	27967.17	*a* ^5^D_3_—*x* ${\;}^{5}D_{4}^{{}^{\circ}}$
40	3575.054	27963.62	*a* ^3^P_2_—*z* ${\;}^{3}S_{1}^{{}^{\circ}}$
70	3577.540	27944.19	*a* ^3^D_2_—*y* ${\;}^{1}F_{3}^{{}^{\circ}}$
30	3578.686	27935.24	*a* ^3^F_2_—*z* ${\;}^{3}P_{2}^{{}^{\circ}}$
100	3579.773	27926.76	*a* ^5^F_3_—*z* ${\;}^{7}F_{2}^{{}^{\circ}}$
150	3584.198	27892.28	*a* ^5^D_1_—*y* ${\;}^{3}D_{2}^{{}^{\circ}}$
30	3585.812	27879.73	*a* ^3^F_3_—*y* ${\;}^{5}F_{2}^{{}^{\circ}}$
300	3587.204	27868.91	*a* ^3^H_5_—*y* ${\;}^{3}H_{5}^{{}^{\circ}}$
2000*R*	3589.220	27853.26	*a* ^5^F_1_—*z* ${\;}^{5}G_{2}^{{}^{\circ}}$
10	3590.525	27843.13	*a* ^3^D_2_—*v* ${\;}^{3}D_{2}^{{}^{\circ}}$
60	3590.884	27840.35	*a* ^3^D_2_—*w* ${\;}^{3}D_{1}^{{}^{\circ}}$
2	3591.333	27836.87	*b* ^3^P_2_—*w* ${\;}^{3}D_{3}^{{}^{\circ}}$
3000*R*	3593.029	27823.73	*a* ^5^F_2_—*z* ${\;}^{5}G_{3}^{{}^{\circ}}$
50	3596.057	27800.30	*a* ^5^F_3_—*z* ${\;}^{7}F_{3}^{{}^{\circ}}$
2000*R*	3596.185	27799.32	*a* ^5^F_3_—*z* ${\;}^{3}G_{4}^{{}^{\circ}}$
10	3598.225	27783.55	*a* ^3^G_3_—*w* ${\;}^{3}D_{3}^{{}^{\circ}}$
20	3598.478	27781.60	*a* ^3^F_2_—*y* ${\;}^{3}D_{2}^{{}^{\circ}}$
30	3599.400	27774.48	*b* ^3^P_1_—*z* ${\;}^{1}D_{2}^{{}^{\circ}}$
1000	3599.769	27771.64	*a* ^5^P_3_—*x* ${\;}^{5}D_{4}^{{}^{\circ}}$
150	3601.487	27758.39	*a* ^3^H_5_— $2_{6}^{{}^{\circ}}$
2	3601.955	27754.78	*a* ^1^D_2_—*x* ${\;}^{5}F_{3}^{{}^{\circ}}$
200	3605.640	27726.42	*a* ^5^P_1_—*z* ${\;}^{3}P_{1}^{{}^{\circ}}$
20	3606.153	27722.47	*a* ^3^F_3_—*y* ${\;}^{5}F_{3}^{{}^{\circ}}$
6	3607.414	27712.78	*a* ^3^D_3_—*y* ${\;}^{1}D_{2}^{{}^{\circ}}$
150	3608.729	27702.69	*a* ^5^D_2_—*y* ${\;}^{3}D_{3}^{{}^{\circ}}$
60	3609.102	27699.82	*a* ^5^F_4_—*z* ${\;}^{5}F_{3}^{{}^{\circ}}$
4	3612.934	27670.44	*c* ^3^P_2_— $39_{1}^{{}^{\circ}}$
5	3616.056	27646.55	*a* ^1^G_4_—*x* ${\;}^{3}F_{4}^{{}^{\circ}}$
150	3616.954	27639.69	*b* ^3^F_4_—*y* ${\;}^{3}D_{3}^{{}^{\circ}}$
2	3618.570	27627.35	*c* ^3^F_2_— $55_{2}^{{}^{\circ}}$
200	3619.201	27622.53	$\left\{ \begin{array}{l} \;a{\;}^{3}G_{4}—1_{4}^{{}^{\circ}}\\ \;a{\;}^{3}H_{5}—w{\;}^{3}G_{4}^{{}^{\circ}} \end{array}\right.$
150	3620.288	27614.24	*b* ^3^P_2_—*x* ${\;}^{3}F_{3}^{{}^{\circ}}$
60	3623.634	27588.74	*b* ^3^F_3_—*y* ${\;}^{3}F_{4}^{{}^{\circ}}$
50	3623.694	27588.28	*b* ^3^P_0_—*z* ${\;}^{1}P_{1}^{{}^{\circ}}$
10*h*	3623.855	27587.06	*a* ^1^H_5_— $24_{5}^{{}^{\circ}}$
300	3625.197	27576.85	*a* ^3^P_2_—*x* ${\;}^{5}D_{1}^{{}^{\circ}}$
300	3626.740	27565.11	*a* ^3^H_4_—*y* ${\;}^{3}H_{4}^{{}^{\circ}}$
100	3627.285	27560.97	*a* ^3^G_3_—*x* ${\;}^{3}F_{3}^{{}^{\circ}}$
20	3631.414	27529.63	*c* ^3^F_3_—*v* ${\;}^{3}G_{4}^{{}^{\circ}}$
150	3631.710	27527.39	*a* ^3^F_4_—*z* ${\;}^{5}P_{3}^{{}^{\circ}}$
20	3632.412	27522.07	*a* ^3^D_1_—*x* ${\;}^{3}P_{2}^{{}^{\circ}}$
150	3633.916	27510.68	*a* ^3^H_4_—*x* ${\;}^{5}F_{4}^{{}^{\circ}}$
1000*R*	3634.931	27503.00	*a* ^5^F_3_—*z* ${\;}^{7}F_{4}^{{}^{\circ}}$
150	3635.512	27498.61	*a* ^5^P_1_—*z* ${\;}^{3}P_{2}^{{}^{\circ}}$
250	3637.461	27483.87	*a* ^3^G_3_—*x* ${\;}^{3}F_{2}^{{}^{\circ}}$
150	3638.011	27479.72	*a* ^3^D_2_—*w* ${\;}^{3}D_{2}^{{}^{\circ}}$
1	3639.140	27471.19	*c* ^3^P_2_— $34_{3}^{{}^{\circ}}$
150	3640.638	27459.89	*a* ^3^G_4_—*y* ${\;}^{3}G_{4}^{{}^{\circ}}$
2	3644.582	27430.17	*b* ^3^H_5_—*v* ${\;}^{3}G_{4}^{{}^{\circ}}$
4	3644.852	27428.14	*a* ^3^H_6_—*z* ${\;}^{3}I_{5}^{{}^{\circ}}$
40	3645.674	27421.96	*b* ^3^F_4_—*x* ${\;}^{5}D_{4}^{{}^{\circ}}$
150	3646.111	27418.67	$\left\{ \begin{array}{l} \;a{\;}^{3}G_{4}—y{\;}^{3}G_{3}^{{}^{\circ}}\\ \;c{\;}^{3}F_{2}—53_{1}^{{}^{\circ}} \end{array}\right.$
2	3649.196	27395.49	*a* ^1^D_2_— $4_{3}^{{}^{\circ}}$
2	3649.538	27392.92	*c* ^3^P_2_— $33_{2}^{{}^{\circ}}$
175	3650.324	27387.03	*a* ^3^F_3_—*y* ${\;}^{5}F_{4}^{{}^{\circ}}$
100	3652.311	27372.13	*a* ^5^F_2_—*z* ${\;}^{7}F_{1}^{{}^{\circ}}$
7	3652.488	27370.80	*b* ^3^P_2_—*x* ${\;}^{3}D_{1}^{{}^{\circ}}$
2	3652.667	27369.46	*a* ^1^H_5_— $21_{5}^{{}^{\circ}}$
30	3653.698	27361.74	*a* ^3^D_2_—*z* ${\;}^{1}P_{1}^{{}^{\circ}}$
150	3654.405	27356.44	*a* ^3^H_5_—*y* ${\;}^{1}H_{5}^{{}^{\circ}}$
4	3655.936	27344.99	*a* ^5^P_1_—*y* ${\;}^{3}D_{2}^{{}^{\circ}}$
6	3655.984	27344.63	*a* ^3^H_4_— $4_{3}^{{}^{\circ}}$
150	3657.169	27335.77	*a* ^5^F_3_—*z* ${\;}^{5}F_{2}^{{}^{\circ}}$
30	3659.470	27318.58	$\left\{ \begin{array}{l} \;a{\;}^{3}D_{3}—u{\;}^{3}D_{2}^{{}^{\circ}}\\ \;b{\;}^{1}D_{2}—60_{1}^{{}^{\circ}} \end{array}\right.$
60	3660.813	27308.56	*a* ^3^H_5_—*y* ${\;}^{3}H_{6}^{{}^{\circ}}$
2000*R*	3661.364	27304.45	*a* ^5^F_4_— *z* ${\;}^{3}G_{5}^{{}^{\circ}}$
50	3661.570	27302.91	*b* ^3^P_2_—*z* ${\;}^{1}F_{3}^{{}^{\circ}}$
800	3663.378	27289.44	*a* ^5^D_4_—*y* ${\;}^{5}F_{5}^{{}^{\circ}}$
2	3667.982	27255.19	*a* ^1^D_2_— $3_{1}^{{}^{\circ}}$
30	3668.742	27249.54	*a* ^3^G_3_—*z* ${\;}^{1}F_{3}^{{}^{\circ}}$
600	3669.546	27243.57	*a* ^3^G_5_—*y* ${\;}^{3}G_{5}^{{}^{\circ}}$
80	3671.212	27231.21	*a* ^5^D_3_—*y* ${\;}^{5}F_{3}^{{}^{\circ}}$
10	3672.059	27224.92	*a* ^3^H_4_—*x* ${\;}^{5}F_{5}^{{}^{\circ}}$
100	3672.378	27222.56	*a* ^3^P_1_—*y* ${\;}^{5}P_{2}^{{}^{\circ}}$
2	3674.002	27210.53	*c* ^3^F_4_— $42_{4}^{{}^{\circ}}$
2	3674.634	27205.85	*a* ^1^H_5_— $19_{4}^{{}^{\circ}}$
40	3675.253	27201.26	*b* ^3^P_1_—*x* ${\;}^{3}F_{2}^{{}^{\circ}}$
2	3676.376	27192.96	*a* ^5^P_3_—*y* ${\;}^{5}F_{2}^{{}^{\circ}}$
60	3676.663	27190.84	*a* ^3^D_1_—*y* ${\;}^{1}D_{2}^{{}^{\circ}}$
40	3676.952	27188.70	*a* ^1^D_2_—*x* ${\;}^{3}P_{2}^{{}^{\circ}}$
10	3677.976	27181.13	*a* ^5^D_2_—*y* ${\;}^{5}F_{1}^{{}^{\circ}}$
10	3678.056	27180.54	*a* ^1^G_4_—*v* ${\;}^{3}D_{3}^{{}^{\circ}}$
200	3678.314	27178.63	*a* ^5^F_2_—*z* ${\;}^{7}F_{3}^{{}^{\circ}}$
10	3680.052	27165.80	*a* ^5^D_1_—*y* ${\;}^{5}F_{1}^{{}^{\circ}}$
1	3682.644	27146.68	*a* ^3^H_4_—*w* ${\;}^{5}D_{4}^{{}^{\circ}}$
16	3683.573	27139.83	*b* ^3^F_2_—*z* ${\;}^{3}S_{1}^{{}^{\circ}}$
40	3685.050	27128.95	*a* ^3^D_1_—*u* ${\;}^{3}D_{1}^{{}^{\circ}}$
100	3685.944	27122.37	*b* ^3^P_2_—*x* ${\;}^{3}D_{3}^{{}^{\circ}}$
20	3686.588	27117.63	*b* ^3^H_4_—*v* ${\;}^{3}G_{5}^{{}^{\circ}}$
4	3688.776	27101.55	*a* ^1^H_5_— $17_{4}^{{}^{\circ}}$
50	3693.589	27066.24	*a* ^3^G_5_—*z* ${\;}^{3}H_{4}^{{}^{\circ}}$
10	3693.634	27065.91	*c* ^3^F_2_— $49_{1}^{{}^{\circ}}$
200	3696.583	27044.31	*a* ^3^P_2_—*x* ${\;}^{5}D_{2}^{{}^{\circ}}$
60	3697.857	27035.00	*b* ^3^P_1_—*x* ${\;}^{3}D_{1}^{{}^{\circ}}$
150	3700.980	27012.18	*a* ^3^H_4_—*y* ${\;}^{3}H_{5}^{{}^{\circ}}$
60	3701.312	27009.76	*a* ^5^F_1_—*z* ${\;}^{7}F_{0}^{{}^{\circ}}$
2	3701.833	27005.96	*a* ^1^P_1_—*w* ${\;}^{3}F_{2}^{{}^{\circ}}$
50	3702.228	27003.08	*a* ^5^P_2_—*z* ${\;}^{5}P_{1}^{{}^{\circ}}$
6	3702.920	26998.03	*c* ^3^F_3_— $50_{2}^{{}^{\circ}}$
60	3703.203	26995.97	*a* ^3^P_2_—*y* ${\;}^{3}D_{1}^{{}^{\circ}}$
30	3705.347	26980.35	*a* ^5^F_2_—*z* ${\;}^{5}F_{1}^{{}^{\circ}}$
3	3705.400	26979.96	*a* ^5^F_1_—*z* ${\;}^{7}F_{1}^{{}^{\circ}}$
4	3709.089	26953.13	*a* ^3^D_2_—*x* ${\;}^{3}D_{2}^{{}^{\circ}}$
20	3710.302	26944.32	*b* ^3^H_4_—*v* ${\;}^{3}G_{4}^{{}^{\circ}}$
100	3712.299	26929.82	*a* ^1^D_2_—*x* ${\;}^{3}G_{3}^{{}^{\circ}}$
50	3714.644	26912.82	*a* ^5^F_1_—*z* ${\;}^{7}F_{2}^{{}^{\circ}}$
125	3715.559	26906.20	*a* ^5^D_2_—*y* ${\;}^{5}F_{2}^{{}^{\circ}}$
150	3716.173	26901.75	*a* ^3^F_4_—*y* ${\;}^{4}D_{4}^{{}^{\circ}}$
300	3716.998	26895.78	*a* ^5^D_3_—*y* ${\;}^{5}F_{4}^{{}^{\circ}}$
40	3717.674	26890.89	*a* ^5^D_1_—*y* ${\;}^{5}F_{2}^{{}^{\circ}}$
50	3718.400	26885.64	*a* ^3^F_4_—*y* ${\;}^{5}D_{3}^{{}^{\circ}}$
300	3719.325	26878.95	*a* ^3^H_4_—*x* ${\;}^{3}G_{3}^{{}^{\circ}}$
4	3721.929	26860.15	*c* ^3^F_4_— $36_{5}^{{}^{\circ}}$
10	3722.303	26857.45	*a* ^1^D_2_—*y* ${\;}^{1}D_{2}^{{}^{\circ}}$
150	3724.967	26838.24	*a* ^5^P_2_—*z* ${\;}^{5}P_{2}^{{}^{\circ}}$
50	3725.482	26834.53	*a* ^3^P_0_—*z* ${\;}^{3}s_{1}^{{}^{\circ}}$
300	3726.096	26830.11	*a* ^3^G_5_—*z* ${\;}^{1}G_{4}^{{}^{\circ}}$
5000*R*	3726.926	26824.14	*a* ^5^F_4_—*z* ${\;}^{5}F_{4}^{{}^{\circ}}$
9000*R*	3728.026	26816.22	*a* ^5^F_5_—*z* ${\;}^{5}F_{5}^{{}^{\circ}}$
2000*R*	3730.432	26798.93	*a* ^5^F_3_—*z* ${\;}^{5}F_{3}^{{}^{\circ}}$
50	3730.592	26797.78	*a* ^3^F_3_—*z* ${\;}^{5}P_{2}^{{}^{\circ}}$
40	3730.899	26795.57	*a* ^1^D_2_—*u* ${\;}^{3}D_{1}^{{}^{\circ}}$
100	3732.020	26787.52	*b* ^3^P_2_—*y* ${\;}^{3}F_{2}^{{}^{\circ}}$
4	3732.744	26782.33	*a* ^1^G_4_—*w* ${\;}^{3}D_{3}^{{}^{\circ}}$
100	3733.041	26780.20	*a* ^3^F_2_—*y* ${\;}^{5}F_{2}^{{}^{\circ}}$
40	3735.020	26766.01	*a* ^3^H_4_—*w* ${\;}^{3}G_{4}^{{}^{\circ}}$
2	3736.823	26753.09	*b* ^3^F_2_—*x* ${\;}^{5}D_{1}^{{}^{\circ}}$
300	3737.407	26748.91	*a* ^5^D_2_—*y* ${\;}^{5}F_{3}^{{}^{\circ}}$
50	3737.758	26746.40	*a* ^5^D_0_—*y* ${\;}^{5}F_{1}^{{}^{\circ}}$
150	3738.630	26740.16	*a* ^3^G_3_— $1_{4}^{{}^{\circ}}$
200	3738.914	26738.13	*a* ^3^H_5_—*z* ${\;}^{3}I_{5}^{{}^{\circ}}$
250	3739.470	26734.16	*a* ^3^G_3_— *y* ${\;}^{3}F_{2}^{{}^{\circ}}$
10	3741.006	26723.18	*a* ^3^P_2_—*z* ${\;}^{3}P_{1}^{{}^{\circ}}$
1000*R*	3742.287	26714.03	*a* ^5^F_2_—*z* ${\;}^{5}F_{2}^{{}^{\circ}}$
400	3742.798	26710.39	*a* ^3^H_6_—*z* ${\;}^{3}I_{7}^{{}^{\circ}}$
60	3743.328	26706.60	*a* ^3^D_3_—*v* ${\;}^{3}D_{2}^{{}^{\circ}}$
40	3743.956	26702.12	*a* ^3^D_2_—*z* ${\;}^{1}D_{2}^{{}^{\circ}}$
125	3744.219	26700.25	*a* ^5^P_3_—*y* ${\;}^{5}F_{4}^{{}^{\circ}}$
125	3744.396	26698.99	*a* ^3^H_5_—*x* ${\;}^{3}G_{4}^{{}^{\circ}}$
500	3745.592	26690.46	*a* ^3^G_5_—*z* ${\;}^{3}H_{6}^{{}^{\circ}}$
50	3746.218	26686.00	*b* ^3^F_4_—*y* ${\;}^{5}F_{3}^{{}^{\circ}}$
40	3751.856	26645.90	*a* ^3^H_4_—*w* ${\;}^{3}G_{5}^{{}^{\circ}}$
60	3752.787	26639.29	*a* ^1^H_5_— $14_{4}^{{}^{\circ}}$
150	3753.546	26633.91	*a* ^3^G_4_—*y* ${\;}^{3}G_{5}^{{}^{\circ}}$
200	3755.094	26622.93	*a* ^3^F_2_—*y* ${\;}^{5}F_{3}^{{}^{\circ}}$
50	3755.728	26618.43	*a* ^5^P_1_—*y* ${\;}^{5}F_{1}^{{}^{\circ}}$
300	3755.937	26616.95	*a* ^3^G_4_—*y* ${\;}^{3}F_{3}^{{}^{\circ}}$
200	3759.838	26589.33	*a* ^5^D_4_—*z* ${\;}^{5}P_{3}^{{}^{\circ}}$
400	3760.019	26588.05	*a* ^5^F_1_—*z* ${\;}^{5}F_{1}^{{}^{\circ}}$
200	3761.511	26577.51	*a* ^3^G_3_—*y* ${\;}^{3}G_{4}^{{}^{\circ}}$
50	3764.037	26559.67	*a* ^1^G_4_—*x* ${\;}^{3}F_{3}^{{}^{\circ}}$
10	3765.808	26547.18	*a* ^1^P_1_— $15_{1}^{{}^{\circ}}$
150	3767.353	26536.30	*a* ^3^G_3_—*y* ${\;}^{3}G_{3}^{{}^{\circ}}$
2	3770.463.	26514.41	*c* ^3^F_3_— $43_{3}^{{}^{\circ}}$
2	3772.386	26500.89	*c* ^3^F_4_— $31_{4}^{{}^{\circ}}$
60	3773.175	26495.35	*a* ^3^P_2_—*z* ${\;}^{3}P_{2}^{{}^{\circ}}$
400	3777.588	26464.40	$\left\{ \begin{array}{l} \;a{\;}^{5}F_{1}—z{\;}^{5}D_{0}^{{}^{\circ}}\\ \;b{\;}^{3}F_{3}—z{\;}^{3}P_{2}^{{}^{\circ}} \end{array}\right.$
60	3777.758	26463.21	*a* ^1^D_2_—*u* ${\;}^{3}D_{2}^{{}^{\circ}}$
150	3778.711	26456.54	*a* ^3^G_4_—*z* ${\;}^{3}H_{4}^{{}^{\circ}}$
10	3779.426	26451.53	*b* ^3^P_1_—*y* ${\;}^{3}F_{2}^{{}^{\circ}}$
50	3779.969	26447.73	*a* ^3^P_0_—*x* ${\;}^{5}D_{1}^{{}^{\circ}}$
150	3781.171	26439.32	*a* ^1^H_5_—*x* ${\;}^{1}H_{5}^{{}^{\circ}}$
30	3781.660	26435.91	*c* ^3^F_2_— $41_{2}^{{}^{\circ}}$
120	3782.749	26428.30	*d* ^3^F_4_— $62_{4}^{{}^{\circ}}$
6	3784.749	26414.33	*a* ^3^P_1_—*x* ${\;}^{5}D_{1}^{{}^{\circ}}$
1000	3786.065	26405.15	*a* ^5^F_2_—*z* ${\;}^{5}D_{1}^{{}^{\circ}}$
1500*R*	3790.521	26374.11	*a* ^5^F_3_—*z* ${\;}^{5}D_{2}^{{}^{\circ}}$
2	3793.916	26350.51	*b* ^3^F_4_—*y* ${\;}^{5}F_{4}^{{}^{\circ}}$
125	3794.924	26343.51	*a* ^5^P_1_—*y* ${\;}^{5}F_{2}^{{}^{\circ}}$
100	3795.185	26341.70	*a* ^3^P_2_—*y* ${\;}^{3}D_{2}^{{}^{\circ}}$
250	3798.054	26321.80	*a* ^5^F_4_—*z* ${\;}^{5}F_{2}^{{}^{\circ}}$
3000*R*	3798.899	26315.95	*a* ^5^F_4_—*z* ${\;}^{5}D_{3}^{{}^{\circ}}$
3000*R*	3799.353	26312.80	*a* ^5^F_5_—*z* ${\;}^{5}D_{4}^{{}^{\circ}}$
150	3800.261	26306.51	*a* ^5^D_3_—*z* ${\;}^{5}P_{2}^{{}^{\circ}}$
100	3803.191	26286.25	*a* ^1^P_1_—*y* ${\;}^{1}P_{1}^{{}^{\circ}}$
100	3805.432	26270.77	*b* ^3^P_2_—*y* ${\;}^{3}P_{1}^{{}^{\circ}}$
125	3808.689	26248.30	*a* ^1^G_4_—*z* ${\;}^{1}F_{3}^{{}^{\circ}}$
300	3812.739	26220.42	$\left\{ \begin{array}{l} \;a{\;}^{3}G_{4}—z{\;}^{1}G_{4}^{{}^{\circ}}\\ \;b{\;}^{3}F_{2}—x{\;}^{5}D_{2}^{{}^{\circ}} \end{array}\right.$
10	3813.164	26217.50	*a* ^1^H_5_— $12_{4}^{{}^{\circ}}$
150	3814.848	26205.93	*a* ^3^D_2_—*x* ${\;}^{3}F_{3}^{{}^{\circ}}$
200	3817.293	26189.14	*a* ^3^H_6_—*x* ${\;}^{3}G_{5}^{{}^{\circ}}$
50	3817.948	26184.65	*a* ^3^D_1_—*v* ${\;}^{3}D_{2}^{{}^{\circ}}$
30	3818.354	26181.87	*a* ^3^D_1_—*w* ${\;}^{3}D_{1}^{{}^{\circ}}$
400	3819.039	26177.17	*a* ^5^F_2_—*z* ${\;}^{5}F_{3}^{{}^{\circ}}$
100	3819.764	26172.20	*b* ^3^F_2_—*y* ${\;}^{3}D_{1}^{{}^{\circ}}$
10*h*	3820.964	26163.98	*a* ^3^H_5_—*z* ${\;}^{1}I_{6}^{{}^{\circ}}$
10	3821.650	26159.28	*a* ^1^F_3_— $61_{4}^{{}^{\circ}}$
300	3822.091	26156.27	*a* ^3^D_3_—*x* ${\;}^{3}F_{4}^{{}^{\circ}}$
2	3823.035	26149.81	*c* ^3^F_2_— $35_{3}^{{}^{\circ}}$
200	3824.938	26136.80	$\left\{ \begin{array}{l} \;a{\;}^{3}P_{2}—y{\;}^{3}D_{3}^{{}^{\circ}}\\ \;b{\;}^{3}H_{4}—46_{3}^{{}^{\circ}} \end{array}\right.$
60	3826.103	26128.84	*a* ^3^D_2_—*x* ${\;}^{3}F_{2}^{{}^{\circ}}$
4	3826.259	26127.77	*b* ^3^P_2_—*y* ${\;}^{5}P_{1}^{{}^{\circ}}$
125	3828.719	26110.99	*a* ^5^P3—*z* ${\;}^{5}P_{2}^{{}^{\circ}}$
50	3829.336	26106.78	*a* ^3^H_5_—*x* ${\;}^{3}F_{4}^{{}^{\circ}}$
20	3829.493	26105.71	*b* ^3^F_3_—*y* ${\;}^{3}D_{3}^{{}^{\circ}}$
50	3830.875	26096.29	*b* ^3^P_2_—*y* ${\;}^{3}P_{2}^{{}^{\circ}}$
200	3831.795	26090.03	*a* ^3^G_5_—*z* ${\;}^{3}H_{5}^{{}^{\circ}}$
5	3834.229	26073.47	*c* ^3^F_3_— $35_{3}^{{}^{\circ}}$
150	3835.062	26067.80	*a* ^1^G_4_—*x* ${\;}^{3}D_{3}^{{}^{\circ}}$
20	3835.983	26061.54	*a* ^1^D_2_—*y* ${\;}^{3}S_{1}^{{}^{\circ}}$
3	3836.968	26054.85	*b* ^1^G_4_— $51_{4}^{{}^{\circ}}$
150	3838.069	26047.38	*a* ^5^P_2_—*y* ${\;}^{5}D_{1}^{{}^{\circ}}$
80	3838.725	26042.93	*a* ^3^G_3_— *y* ${\;}^{3}P_{2}^{{}^{\circ}}$
400	3839.699	26036.32	*a* ^3^G_5_— *y* ${\;}^{3}F_{4}^{{}^{\circ}}$
125	3840.823	26028.70	*a* ^5^P_2_—*z* ${\;}^{5}P_{3}^{{}^{\circ}}$
100	3840.989	26027.58	*a* ^3^H_6_—*z* ${\;}^{3}I_{6}^{{}^{\circ}}$
30	3842.671	26016.18	*a* ^3^P_1_—*x* ${\;}^{5}D_{0}^{{}^{\circ}}$
150	3843.152	26012.93	*a* ^5^F_1_ —*z* ${\;}^{5}D_{1}^{{}^{\circ}}$
300	3846.672	25989.13	*a* ^5^D_2_—*z* ${\;}^{5}P_{1}^{{}^{\circ}}$
50	3846.801	25988.26	*a* ^3^F_3_—*z* ${\;}^{5}P_{3}^{{}^{\circ}}$
2	3847.491	25983.60	*c* ^3^F_2_— $33_{2}^{{}^{\circ}}$
40	3848.944	25973.79	*a* ^5^D_1_—*z* ${\;}^{5}P_{1}^{{}^{\circ}}$
400	3850.441	25963.69	*a* ^5^D_4_—*y* ${\;}^{5}D_{4}^{{}^{\circ}}$
125	3852.132	25952.29	*a* ^1^D_2_—*y* ${\;}^{1}F_{3}^{{}^{\circ}}$
10	3852.565	25949.37	$\left\{ \begin{array}{l} \;z{\;}^{7}D_{1}^{{}^{\circ}}—f{\;}^{7}D_{1}\\ \;a{\;}^{1}P_{1}—w{\;}^{5}D_{1}^{{}^{\circ}} \end{array}\right.$
20	3852.835	25947.56	*a* ^5^D_4_—*y* ${\;}^{5}D_{3}^{{}^{\circ}}$
100	3854.720	25934.87	*b* ^3^P_1_—*y* ${\;}^{3}P_{1}^{{}^{\circ}}$
5	3855.579	25929.09	*b* ^3^H_4_— $43_{3}^{{}^{\circ}}$
300	3856.458	25923.18	*a* ^5^F_3_—*z* ${\;}^{5}F_{4}^{{}^{\circ}}$
60	3856.972	25919.72	*b* ^3^F_2_—*x* ${\;}^{5}D_{3}^{{}^{\circ}}$
1000	3857.551	25915.83	*a* ^1^G_4_—*z* ${\;}^{1}H_{5}^{{}^{\circ}}$
30	3857.998	25912.83	*b* ^3^P_1_—*y* ${\;}^{3}P_{0}^{{}^{\circ}}$
60	3858.687	25908.20	*a* ^3^D_4_—*z* ${\;}^{1}S_{0}^{{}^{\circ}}$
100	3859.696	25901.43	*a* ^3^H_4_—*y* ${\;}^{1}F_{3}^{{}^{\circ}}$
200	3860.725	25894.53	*a* ^3^D_2_—*z* ${\;}^{1}F_{3}^{{}^{\circ}}$
30	3861.453	25889.65	*a* ^3^G_4_—*y* ${\;}^{5}P_{3}^{{}^{\circ}}$
1000	3862.690	25881.36	*a* ^3^H_4_—*z* ${\;}^{3}I_{5}^{{}^{\circ}}$
40	3864.851	25866.89	*a* ^3^P_0_—*y* ${\;}^{3}D_{1}^{{}^{\circ}}$
150	3865.419	25863.09	*a* ^3^F_2_—*z* ${\;}^{5}P_{1}^{{}^{\circ}}$
6	3865.742	25860.92	*a* ^1^P_1_— $11_{0}^{{}^{\circ}}$
4	3867.186	25851.27	*a* ^1^D_2_—*v* ${\;}^{3}D_{2}^{{}^{\circ}}$
1500	3867.844	25846.87	*a* ^3^F_4_—*z* ${\;}^{3}F_{3}^{{}^{\circ}}$
100	3871.231	25824.26	*a* ^5^D_2_—*z* ${\;}^{5}P_{2}^{{}^{\circ}}$
10	3871.695	25821.16	*a* ^3^D_1_—*w* ${\;}^{3}D_{2}^{{}^{\circ}}$
6	3872.381	25816.59	*a* ^3^D_3_—*x* ${\;}^{3}D_{2}^{{}^{\circ}}$
4	3872.730	25814.26	*a* ^1^P_1_— $9_{1}^{{}^{\circ}}$
200	3873.537	25808.88	*a* ^5^D_1_—*z* ${\;}^{5}P_{2}^{{}^{\circ}}$
150	3876.102	25791.81	*b* ^3^P_1_—*y* ${\;}^{5}P_{1}^{{}^{\circ}}$
100	3876.679	25787.97	*b* ^3^P_2_—*y* ${\;}^{3}F_{3}^{{}^{\circ}}$
4	3877.575	25782.01	*c* ^3^F_4_— $21_{5}^{{}^{\circ}}$
60	3880.833	25760.36	*b* ^3^P_1_—*y* ${\;}^{3}P_{2}^{{}^{\circ}}$
125	3882.034	25752.39	*a* ^5^F_2_—*z* ${\;}^{5}D_{2}^{{}^{\circ}}$
100	3884.075	25738.86	*a* ^1^G_4_— $1_{4}^{{}^{\circ}}$
100	3884.718	25734.60	*a* ^3^G_3_—*y* ${\;}^{3}F_{3}^{{}^{\circ}}$
2	3887.308	25717.46	*b* ^3^G_4_— $61_{4}^{{}^{\circ}}$
100	3887.826	25714.03	*a* ^3^D_2_—*x* ${\;}^{3}D_{3}^{{}^{\circ}}$
125	3890.216	25698.23	*a* ^3^F_2_—*z* ${\;}^{5}P_{2}^{{}^{\circ}}$
60	3891.425	25690.25	*a* ^3^D_3_—*v* ${\;}^{3}D_{3}^{{}^{\circ}}$
500	3892.230	25684.93	*a* ^5^P_2_—*y* ${\;}^{5}D_{2}^{{}^{\circ}}$
40	3892.773	25681.35	*a* ^1^P_1_—*t* ${\;}^{3}D_{2}^{{}^{\circ}}$
50	3894.255	25671.58	*b* ^3^F_2_—*z* ${\;}^{3}P_{2}^{{}^{\circ}}$
125	3897.249	25651.86	*b* ^3^F_4_—*y* ${\;}^{5}F_{5}^{{}^{\circ}}$
125	3898.366	25644.51	*a* ^3^F_3_—*y* ${\;}^{5}D_{2}^{{}^{\circ}}$
150	3901.257	25625.50	*a* ^5^F_4_—*z* ${\;}^{5}F_{5}^{{}^{\circ}}$
15	3902.824	25615.22	*a* ^3^P_2_—*y* ${\;}^{5}F_{1}^{{}^{\circ}}$
20	3905.993	25594.43	*c* ^3^P_2_—*y* ${\;}^{1}P_{1}^{{}^{\circ}}$
125	3908.768	25576.26	*a* ^1^G_4_—*y* ${\;}^{3}G_{4}^{{}^{\circ}}$
600	3909.085	25574.19	*a* ^3^G_3_—*z* ${\;}^{3}H_{4}^{{}^{\circ}}$
40	3911.147	25560.71	*a* ^3^P_1_—*z* ${\;}^{3}P_{1}^{{}^{\circ}}$
125	3912.117	25554.37	*a* ^5^D_0_—*z* ${\;}^{5}P_{1}^{{}^{\circ}}$
2	3912.992	25548.66	*a* ^1^P_1_—*w* ${\;}^{5}D_{2}^{{}^{\circ}}$
200	3914.859	25536.47	*a* ^5^P_2_—*z* ${\;}^{3}D_{1}^{{}^{\circ}}$
4	3917.706	25517.92	*b* ^3^F_2_—*y* ${\;}^{3}D_{2}^{{}^{\circ}}$
150	3920.923	25496.98	*a* ^5^D_3_—*z* ${\;}^{5}P_{3}^{{}^{\circ}}$
10	3922.338	25487.78	*a* ^1^D_2_—*w* ${\;}^{3}D_{2}^{{}^{\circ}}$
1000	3923.486	25480.32	*a* ^3^G_4_—*z* ${\;}^{3}H_{5}^{{}^{\circ}}$
60	3924.636	25472.86	*a* ^1^H_5_—*x* ${\;}^{1}G_{4}^{{}^{\circ}}$
1000	3925.930	25464.46	*a* ^5^F_5_—*z* ${\;}^{7}D_{4}^{{}^{\circ}}$
3	3926.46	25461.02	*b* ^1^G_4_— $42_{4}^{{}^{\circ}}$
600	3931.787	25426.53	$\left\{ \begin{array}{l} \;a{\;}^{5}P_{1}—z{\;}^{5}P_{1}^{{}^{\circ}}\\ \;a{\;}^{3}G_{4}—y{\;}^{3}F_{4}^{{}^{\circ}} \end{array}\right.$
300	3933.566	25415.03	*a* ^5^F_3_—*z* ${\;}^{5}D_{3}^{{}^{\circ}}$
2	3935.363	25403.43	*c* ^3^F_4_— $16_{3}^{{}^{\circ}}$
4	3936.168	25398.23	*c* ^3^F_3_— $27_{4}^{{}^{\circ}}$
250	3937.919	25386.94	*a* ^5^P2—*y* ${\;}^{5}D_{3}^{{}^{\circ}}$
10	3939.126	25379.16	*a* ^3^D_2_—*y* ${\;}^{3}F_{2}^{{}^{\circ}}$
200	3941.672	25362.76	$\left\{ \begin{array}{l} \;b{\;}^{3}P_{2}—y{\;}^{5}P_{2}^{{}^{\circ}}\\ \;a{\;}^{3}F_{3}—y{\;}^{5}D_{4}^{{}^{\circ}} \end{array}\right.$
200	3942.078	25360.15	*a* ^5^F_1_—*z* ${\;}^{5}D_{2}^{{}^{\circ}}$
200	3944.200	25346.51	*a* ^3^F_3_—*y* ${\;}^{5}D_{3}^{{}^{\circ}}$
1000	3945.586	25337.61	*a* ^3^H_5_—*z* ${\;}^{3}I_{6}^{{}^{\circ}}$
60	3946.321	25332.89	*a* ^3^P_1_—*z* ${\;}^{3}P_{2}^{{}^{\circ}}$
60	3949.417	25313.03	*b* ^3^F_2_— *y* ${\;}^{3}D_{3}^{{}^{\circ}}$
150	3950.041	25309.03	$\left\{ \begin{array}{l} \;a{\;}^{1}H_{5}—y{\;}^{1}G_{4}^{{}^{\circ}}\\ \;b{\;}^{3}F_{3}—y{\;}^{5}F_{2}^{{}^{\circ}} \end{array}\right.$
200	3950.230	25307.82	*a* ^3^F_4_—*z* ${\;}^{3}G_{3}^{{}^{\circ}}$
150	3950.410	25306.67	*a* ^5^D_1_—*y* ${\;}^{5}D_{0}^{{}^{\circ}}$
125	3951.212	25301.53	*a* ^5^P_3_—*z* ${\;}^{5}P_{3}^{{}^{\circ}}$
20	3952.290	25294.63	*a* ^3^D_1_—*x* ${\;}^{3}D_{2}^{{}^{\circ}}$
200	3952.703	25291.99	*a* ^3^D_3_—*w* ${\;}^{3}D_{3}^{{}^{\circ}}$
10*h*	3953.849	25284.66	*b* ^3^G_3_— $61_{4}^{{}^{\circ}}$
40	3957.228	25263.07	*b* ^3^H_6_— $21_{5}^{{}^{\circ}}$
100	3957.448	25261.66	*a* ^5^P_1_—*z* ${\;}^{5}P_{2}^{{}^{\circ}}$
500	3964.908	25214.13	*a* ^5^F_5_—*z* ${\;}^{7}D_{5}^{{}^{\circ}}$
100	3969.794	25183.10	*a* ^3^P_2_—*y* ${\;}^{5}F_{3}^{{}^{\circ}}$
4	3970.404	25179.23	*a* ^3^P_1_—*y* ${\;}^{3}D_{2}^{{}^{\circ}}$
150	3974.504	25153.26	*a* ^5^D_3_*—y* ${\;}^{5}D_{2}^{{}^{\circ}}$
2	3976.729	25139.18	*b* ^3^H_4_— $31_{4}^{{}^{\circ}}$
500	3978.449	25128.32	*a* ^5^P_2_—*z* ${\;}^{3}F_{2}^{{}^{\circ}}$
500	3979.420	25122.18	*a* ^5^F_4_—*z* ${\;}^{5}D_{4}^{{}^{\circ}}$
60	3981.895	25106.57	*c* ^3^F_3_— $22_{3}^{{}^{\circ}}$
50	3982.217	25104.54	*b* ^3^H_4_— $30_{4}^{{}^{\circ}}$
20	3984.678	25089.04	*b* ^3^P_0_*—y* ${\;}^{3}P_{1}^{{}^{\circ}}$
1000	3984.862	25087.88	*a* ^3^F_3_—*z* ${\;}^{3}F_{2}^{{}^{\circ}}$
400	3987.797	25069.41	*a* ^3^D_3_*—x* ${\;}^{3}F_{3}^{{}^{\circ}}$
60	3989.194	25060.63	*b* ^3^P_2_*—y* ${\;}^{5}P_{3}^{{}^{\circ}}$
4	3991.793	25044.32	*c* ^3^P_2_— $8_{1}^{{}^{\circ}}$
100	3993.532	25033.41	*a* ^5^D_2_*—y* ${\;}^{5}D_{1}^{{}^{\circ}}$
10	3994.562	25026.96	*b* ^3^P_1_*—y* ${\;}^{5}P_{2}^{{}^{\circ}}$
200	3995.982	25018.06	*a* ^5^D_1_*—y* ${\;}^{5}D_{1}^{{}^{\circ}}$
100	3996.504	25014.80	*a* ^5^D_2_—*z* ${\;}^{5}P_{3}^{{}^{\circ}}$
4	3999.488	24996.13	*c* ^3^F_2_—*s* ${\;}^{3}D_{3}^{{}^{\circ}}$
4	4000.103	24992.29	*a* ^3^D_3_*—x* ${\;}^{3}F_{2}^{{}^{\circ}}$
4	4000.550	24989.50	*c* ^3^P_2_*—t* ${\;}^{3}D_{2}^{{}^{\circ}}$
6	4005.089	24961.18	*a* ^1^D_2_—*x* ${\;}^{3}D_{2}^{{}^{\circ}}$
200	4005.646	24957.71	*a* ^5^P_3_*—y* ${\;}^{5}D_{2}^{{}^{\circ}}$
150	4006.602	24951.75	*b* ^3^F_4_—*z* ${\;}^{5}P_{3}^{{}^{\circ}}$
100	4007.533	24945.96	*b* ^3^P_0_—*y* ${\;}^{5}P_{1}^{{}^{\circ}}$
125	4008.266	24941.39	*b* ^3^P_2_—*z* ${\;}^{3}S_{1}^{{}^{\circ}}$
20	4011.732	24919.85	*c* ^3^F_3_—*s* ${\;}^{3}D_{3}^{{}^{\circ}}$
125	4013.502	24908.86	*a* ^5^D_4_—*z* ${\;}^{3}F_{3}^{{}^{\circ}}$
40	4013.736	24907.40	*a* ^3^F_2_*—y* ${\;}^{5}D_{1}^{{}^{\circ}}$
40	4014.153	24904.82	*c* ^3^F_2_—*w* ${\;}^{3}F_{2}^{{}^{\circ}}$
20	4016.740	24888.78	*a* ^3^F_2_—*z* ${\;}^{5}P_{3}^{{}^{\circ}}$
60	4019.551	24871.37	*a* ^5^D_3_*—y* ${\;}^{5}D_{4}^{{}^{\circ}}$
150	4020.994	24862.45	*a* ^3^D_2_*—y* ${\;}^{3}P_{1}^{{}^{\circ}}$
800	4022.168	24855.19	*a* ^5^D_3_—*z* ${\;}^{5}D_{3}^{{}^{\circ}}$
30	4022.680	24852.03	*c* ^3^F_4_*—x* ${\;}^{1}H_{3}^{{}^{\circ}}$
600	4023.832	24844.91	*a* ^5^F_4_—*z* ${\;}^{7}D_{3}^{{}^{\circ}}$
50	4024.300	24842.02	*c* ^3^F_3_— $19_{4}^{{}^{\circ}}$
100	4024.689	24839.62	*a* ^3^F_4_—*z* ${\;}^{7}P_{3}^{{}^{\circ}}$
2	4025.465	24834.83	*a* ^1^D_2_— *v* ${\;}^{3}D_{3}^{{}^{\circ}}$
10	4026.492	24828.50	*c* ^3^F_3_—*w* ${\;}^{3}F_{2}^{{}^{\circ}}$
40	4028.434	24816.53	*b* ^3^F_3_*—y* ${\;}^{5}F_{4}^{{}^{\circ}}$
200	4031.003	24800.71	*a* ^3^F_4_—*z* ${\;}^{5}G_{4}^{{}^{\circ}}$
200	4032.207	24793.31	*a* ^5^F_2_ —*z* ${\;}^{5}D_{3}^{{}^{\circ}}$
50	4032.509	24791.45	*b* ^3^F_2_*—y* ${\;}^{5}F_{1}^{{}^{\circ}}$
1	4033.724	24783.98	$\{\;\begin{array}{c} a{\;}^{3}H_{4}—v{\;}^{3}D_{3}^{{}^{\circ}}\\ b{\;}^{3}H_{4}—25_{3}^{{}^{\circ}} \end{array}$
80	4037.738	24759.35	*a* ^5^P_1_*—y* ${\;}^{5}D_{0}^{{}^{\circ}}$
300	4039.215	24750.29	*a* ^1^G_4_*—y* ${\;}^{3}G_{5}^{{}^{\circ}}$
50	4040.474	24742.58	6 ^3^H_5_— $19_{4}^{{}^{\circ}}$
6	4041.259	24737.78	*c* ^3^F_3_— $17_{4}^{{}^{\circ}}$
20	4041.980	24733.36	*a* ^1^G_4_*—y* ${\;}^{3}F_{3}^{{}^{\circ}}$
2	4044.262	24719.41	*a* ^3^D_2_—*y* ${\;}^{5}P_{1}^{{}^{\circ}}$
2	4044.710	24716.67	*b* ^1^G_4_— $30_{4}^{{}^{\circ}}$
150	4045.770	24710.19	*a* ^1^D_2_—*z* ${\;}^{1}D_{2}^{{}^{\circ}}$
20	4046.883	24703.40	*c* ^3^F_2_— $16_{3}^{{}^{\circ}}$
150	4049.418	24687.93	*a* ^3^D_2_*—y* ${\;}^{3}P_{2}^{{}^{\circ}}$
1000	4051.402	24675.84	*a* ^5^P_3_*—y* ${\;}^{5}D_{4}^{{}^{\circ}}$
200	4052.194	24671.02	*a* ^5^D_2_*—y* ${\;}^{5}D_{2}^{{}^{\circ}}$
600	4054.050	24659.73	*a* ^5^P_3_—*y* ${\;}^{5}D_{3}^{{}^{\circ}}$
10	4054.713	24655.69	*a* ^5^D_1_—*y* ${\;}^{5}D_{2}^{{}^{\circ}}$
1	4057.562	24638.38	*b* ^3^H_5_— $17_{4}^{{}^{\circ}}$
25	4058.852	24630.55	*b* ^3^H_4_— $23_{3}^{{}^{\circ}}$
4	4059.427	24627.06	*c* ^3^F_3_— $16_{3}^{{}^{\circ}}$
60	4062.854	24606.29	*a* ^1^H_5_*—y* ${\;}^{3}H_{4}^{{}^{\circ}}$
200	4062.989	24605.47	*b* ^3^P_1_—*z* ${\;}^{3}S_{1}^{{}^{\circ}}$
20	4063.325	24603.44	*c* ^3^F_2_—*w* ${\;}^{3}F_{3}^{{}^{\circ}}$
200	4064.105	24598.72	*a* ^5^D_0_—*y* ${\;}^{5}D_{1}^{{}^{\circ}}$
600	4064.456	24596.59	*a* ^5^D_3_—*z* ${\;}^{3}F_{2}^{{}^{\circ}}$
6	4066.222	24585.91	*z* ${\;}^{7}D_{2}^{{}^{\circ}}$—*f* ${\;}^{7}D_{2}^{{}^{\circ}}$
200	4067.610	24577.52	*a* ^3^D_3_*—x* ${\;}^{3}D_{3}^{{}^{\circ}}$
600	4068.367	24572.95	*a* ^1^G_4_*—z* ${\;}^{3}H_{4}^{{}^{\circ}}$
150	4071.398	24554.65	*b* ^3^P_2_—*x* ${\;}^{5}D_{1}^{{}^{\circ}}$
20	4071.865	24551.84	*a* ^1^H_5_*—x* ${\;}^{5}F_{4}^{{}^{\circ}}$
125	4072.996	24545.02	*a* ^3^F_2_—*y* ${\;}^{5}D_{2}^{{}^{\circ}}$
60	4073.116	24544.30	*a* ^3^G_3_*—y* ${\;}^{3}F_{4}^{{}^{\circ}}$
3	4075.978	24527.06	*c* ^3^F_3_—*w* ${\;}^{3}F_{3}^{{}^{\circ}}$
800	4076.730	24522.54	*a* ^5^D_2_—*z* ${\;}^{3}D_{1}^{{}^{\circ}}$
100	4079.277	24507.23	*a* ^5^D_1_—*z* ${\;}^{3}D_{1}^{{}^{\circ}}$
1500	4080.599	24499.29	*a* ^3^F_4_—*z* ${\;}^{3}D_{3}^{{}^{\circ}}$
100	4082.789	24486.15	*a* ^3^P_0_*—y* ${\;}^{5}F_{1}^{{}^{\circ}}$
4	4083.868	24479.68	*b* ^3^G_4_—*v* ${\;}^{3}G_{5}^{{}^{\circ}}$
100	4085.353	24470.78	*a* ^5^P_1_—*y* ${\;}^{5}D_{1}^{{}^{\circ}}$
300	4085.432	24470.31	*a* ^3^D_1_*—x* ${\;}^{3}F_{2}^{{}^{\circ}}$
40	4088.370	24452.72	*a* ^3^P_1_*—y* ${\;}^{5}F_{1}^{{}^{\circ}}$
150	4091.062	24436.63	*a* ^1^D_2_—*w* ${\;}^{3}D_{3}^{{}^{\circ}}$
10	4093.792	24420.34	*b* ^3^H_4_— $21_{5}^{{}^{\circ}}$
250	4097.022	24401.09	*a* ^5^P_3_—*z* ${\;}^{3}F_{2}^{{}^{\circ}}$
1000	4097.787	24396.53	*a* ^3^F_2_—*z* ${\;}^{3}D_{1}^{{}^{\circ}}$
150	4100.365	24381.19	*a* ^5^F_3_—*z* ${\;}^{7}D_{2}^{{}^{\circ}}$
40	4101.226	24376.07	*a* ^3^H_5_—*z* ${\;}^{1}H_{5}^{{}^{\circ}}$
300	4101.742	24373.01	*a* ^5^D_2_*—y* ${\;}^{5}D_{3}^{{}^{\circ}}$
150	4102.281	24369.81	*a* ^5^D_4_—*z* ${\;}^{3}D_{3}^{{}^{\circ}}$
2	4104.049	24359.31	*b* ^3^F_2_—*z* ${\;}^{5}F_{3}^{{}^{\circ}}$
50	4105.909	24348.27	*a* ^5^P_2_—*z* ${\;}^{3}F_{2}^{{}^{\circ}}$
2	4106.217	24346.45	*b* ^3^G_6_—*v* ${\;}^{3}G_{4}^{{}^{\circ}}$
200	4107.837	24336.84	*a* ^1^G_4_—*z* ${\;}^{1}G_{4}^{{}^{\circ}}$
100	4108.055	24335.55	*a* ^3^G_5_*—x* ${\;}^{5}D_{4}^{{}^{\circ}}$
60	4109.640	24326.17	*b* ^3^F_4_—*y* ${\;}^{5}D_{4}^{{}^{\circ}}$
2000	4112.741	24307.83	*a* ^3^F_3_—2 ${\;}^{3}F_{3}^{{}^{\circ}}$
150	4113.380	24304.05	*a* ^3^D_1_—*x* ${\;}^{3}D_{1}^{{}^{\circ}}$
60	4114.128	24299.63	*a* ^5^P_2_—*z* ${\;}^{7}P_{2}^{{}^{\circ}}$
4	4117.76	24278.20	*z* ${\;}^{7}D_{2}^{{}^{\circ}}$*—f* ^7^D_2_
200	4118.500	24273.84	*a* ^5^F_4_—*z* ${\;}^{7}D_{4}^{{}^{\circ}}$
6	4119.458	24268.19	*c* ^3^P_2_—*x* ${\;}^{1}F_{3}^{{}^{\circ}}$
300	4120.986	24259.19	*a* ^3^F_3_—*z* ${\;}^{7}P_{2}^{{}^{\circ}}$
100	4121.126	24258.37	*a* ^3^P_2_—*z* ${\;}^{5}P_{2}^{{}^{\circ}}$
5	4122.786	24248.60	*a* ^3^D_3_— $1_{4}^{{}^{\circ}}$
300	4123.061	24246.99	*a* ^3^F_2_—*y* ${\;}^{5}D_{3}^{{}^{\circ}}$
75	4123.801	24242.63	$\{\;\begin{array}{c} a{\;}^{3}D_{3}—y{\;}^{3}F_{2}^{{}^{\circ}}\\ b{\;}^{1}G_{4}—23_{3}^{{}^{\circ}} \end{array}$
30	4126.407	24227.32	*b* ^3^F_3_—*z* ${\;}^{5}P_{2}^{{}^{\circ}}$
200	4127.439	24221.27	*a* ^5^F_3_—*z* ${\;}^{5}D_{4}^{{}^{\circ}}$
200	4127.869	24218.74	*b* ^3^P_1_—*x* ${\;}^{5}D_{1}^{{}^{\circ}}$
2	4128.664	24214.08	*a* ^1^D_2_*—x* ${\;}^{3}F_{3}^{{}^{\circ}}$
20	4131.215	24199.13	*a* ^3^H_5_— $1_{4}^{{}^{\circ}}$
1	4133.614	24185.08	*c* ^3^F_2_—*y* ${\;}^{1}P_{1}^{{}^{\circ}}$
50	4134.854	24177.83	*a* ^3^P_1_—*y* ${\;}^{5}F_{2}^{{}^{\circ}}$
200	4137.223	24163.99	*a* ^5^P_2_—*z* ${\;}^{3}D_{2}^{{}^{\circ}}$
4	4137.948	24159.76	*z* ${\;}^{7}D_{4}^{{}^{\circ}}$—*f* ${\;}^{7}D_{4}^{{}^{\circ}}$
2	4139.192	24152.49	$\{\;\begin{array}{c} b{\;}^{3}H_{4}—17_{4}^{{}^{\circ}}\\ z{\;}^{3}G_{4}^{{}^{\circ}}—E_{4}\; \end{array}$
4	4140.276	24146.17	*z* ${\;}^{7}D_{2}^{{}^{\circ}}$—*e* ^7^D_1_
2*H*	4141.856	24136.96	*a* ^1^D_2_*—x* ${\;}^{3}F_{2}^{{}^{\circ}}$
2000	4144.160	24123.54	*a* ^3^F_3_—*z* ${\;}^{3}D_{2}^{{}^{\circ}}$
1500	4145.737	24114.36	*a* ^5^D_2_—*z* ${\;}^{3}F_{2}^{{}^{\circ}}$
600	4146.770	24108.36	*a* ^5^P_1_*—y* ${\;}^{5}D_{2}^{{}^{\circ}}$
200	4148.376	24099.02	*a* ^5^D_1_*—z* ${\;}^{3}F_{2}^{{}^{\circ}}$
100	4150.303	24087.83	*a* ^5^D_0_—*z* ${\;}^{3}D_{1}^{{}^{\circ}}$
10	4150.620	24085.99	*a* ^3^D_3_—*y* ${\;}^{3}D_{4}^{{}^{\circ}}$
2	4152.922	24072.64	*b* ^1^D_2_— $22_{3}^{{}^{\circ}}$
30	4153.865	24067.18	*a* ^5^F_2_—*z* ${\;}^{7}D_{1}^{{}^{\circ}}$
25*H*	4156.254	24053.34	*a* ^1^H_5_*—y* ${\;}^{3}H_{5}^{{}^{\circ}}$
50	4157.734	24044.78	*a* ^3^D_3_—*y* ${\;}^{3}G_{3}^{{}^{\circ}}$
4	4158.260	24041.74	*b* ^3^H_4_— $16_{3}^{{}^{\circ}}$
6	4158.967	24037.66	*z* ${\;}^{5}D_{4}^{{}^{\circ}}$—*f* ^5^D_4_
80	4159.168	24036.49	*a* ^3^H_5_*—y* ${\;}^{3}G_{4}^{{}^{\circ}}$
250	4161.655	24022.13	*b* ^3^P_2_—*x* ${\;}^{5}D_{2}^{{}^{\circ}}$
10	4164.387	24006.37	*d* ^3^P_2_— $43_{3}^{{}^{\circ}}$
150	4166.879	23992.01	*a* ^3^F_4_—*z* ${\;}^{5}D_{3}^{{}^{\circ}}$
2000	4167.514	23988.36	*a* ^3^F_2_—*z* ${\;}^{3}F_{2}^{{}^{\circ}}$
6	4168.758	23981.20	*z* ${\;}^{7}D_{3}^{{}^{\circ}}$—*e* ^7^D_3_
200	4170.050	23973.77	*b* ^3^P_2_—*y* ${\;}^{3}D_{1}^{{}^{\circ}}$
4	4170.592	23970.65	*a* ^1^D_2_—*x* ${\;}^{3}D_{1}^{{}^{\circ}}$
1	4170.908	23968.84	*a* ^3^G_3_*—x* ${\;}^{5}D_{2}^{{}^{\circ}}$
4	4171.686	23964.37	*a* ^1^F_3_— $47_{4}^{{}^{\circ}}$
1	4172.464	23959.90	*a* ^5^P_1_—*z* ${\;}^{3}D_{1}^{{}^{\circ}}$
20	4173.396	23954.55	*a* ^3^D_2_—*y* ${\;}^{5}P_{2}^{{}^{\circ}}$
10	4175.299	23943.63	*a* ^3^G_4_—*y* ${\;}^{3}D_{3}^{{}^{\circ}}$
30	4175.436	23942.85	*a* ^1^H_5_—*z* $2_{6}^{{}^{\circ}}$
6*h*	4175.614	23941.83	*b* ^3^H_4_*—w* ${\;}^{3}F_{3}^{{}^{\circ}}$
200	4182.455	23902.67	*a* ^1^D_2_—*z* ${\;}^{1}F_{3}^{{}^{\circ}}$
100	4182.646	23901.58	*a* ^5^D_4_—*z* ${\;}^{7}P_{3}^{{}^{\circ}}$
60	4182.832	23900.51	*a* ^3^H_6_*—y* ${\;}^{3}G_{5}^{{}^{\circ}}$
10	4185.465	23885.48	*c* ^3^F_4_—*x* ${\;}^{1}G_{4}^{{}^{\circ}}$
4	4188.380	23868.85	*b* ^1^G_4_— $19_{4}^{{}^{\circ}}$
150	4189.463	23862.68	*a* ^5^D_4_—*z* ${\;}^{5}G_{4}^{{}^{\circ}}$
6	4191.022	23853.81	*c* ^3^F_3_— $12_{4}^{{}^{\circ}}$
1	4192.582	23844.93	*b* ^3^G_5_— $51_{4}^{{}^{\circ}}$
4	4193.723	23838.44	*z* ${\;}^{7}D_{1}^{{}^{\circ}}$—*e* ^7^D_1_
200	4196.863	23820.61	*b* ^3^P_1_—*x* ${\;}^{5}D_{0}^{{}^{\circ}}$
400	4197.572	23816.58	*a* ^5^D_3_—*z* ${\;}^{3}F_{3}^{{}^{\circ}}$
400	4198.864	23809.26	*a* ^5^P_2_—*z* ${\;}^{3}G_{3}^{{}^{\circ}}$
3000	4199.892	23803.43	*a* ^3^F_4_—*z* ${\;}^{3}F_{4}^{{}^{\circ}}$
20	4205.678	23770.68	*z* ${\;}^{7}D_{4}^{{}^{\circ}}$—*e* ^7^D_4_
800	4206.020	23768.75	*a* ^3^F_3_—*z* ${\;}^{3}G_{3}^{{}^{\circ}}$
150	4207.626	23759.68	*z* ${\;}^{7}D_{1}^{{}^{\circ}}$—*e* ^7^D_2_
1500	4212.062	23734.66	*a* ^3^F_4_—*z* ${\;}^{5}G_{5}^{{}^{\circ}}$
300	4214.445	23721.24	*b* ^3^P_2_—*x* ${\;}^{5}D_{3}^{{}^{\circ}}$
100	4214.553	23720.63	*a* ^3^D_1_*—y* ${\;}^{3}F_{2}^{{}^{\circ}}$
200	4217.263	23705.38	*a* ^3^F_4_—*z* ${\;}^{7}P_{4}^{{}^{\circ}}$
160	4220.675	23686.22	*b* ^3^P_1_*—x* ${\;}^{5}D_{2}^{{}^{\circ}}$
1	4223.347	23671.24	*a* ^3^H_4_*—x* ${\;}^{3}D_{3}^{{}^{\circ}}$
120	4225.095	23661.44	*a* ^1^P_1_—*y* ${\;}^{1}D_{2}^{{}^{\circ}}$
20	4226.668	23652.64	*a* ^1^F_3_— $42_{4}^{{}^{\circ}}$
100	4229.312	23637.85	*b* ^3^P_1_*—y* ${\;}^{3}D_{1}^{{}^{\circ}}$
250	4230.309	23632.28	*a* ^5^D_3_—*z* ${\;}^{3}D_{2}^{{}^{\circ}}$
100	4232.321	23621.05	*a* ^5^P_3_—*z* ${\;}^{3}F_{3}^{{}^{\circ}}$
60	4236.675	23596.77	*a* ^1^G_4_—*z* ${\;}^{3}H_{5}^{{}^{\circ}}$
6	4239.660	23580.16	*c* ^3^F_2_—*t* ${\;}^{3}D_{2}^{{}^{\circ}}$
4	4240.031	23578.09	*a* ^1^F_3_— $41_{2}^{{}^{\circ}}$
400	4241.058	23572.38	*a* ^5^P_3_—*z* ${\;}^{7}P_{2}^{{}^{\circ}}$
400	4243.058	23561.27	*a* ^5^D_4_—*z* ${\;}^{3}D_{3}^{{}^{\circ}}$
60	4244.834	23551.42	*a* ^3^D_3_—*y* ${\;}^{3}P_{2}^{{}^{\circ}}$
100	4246.342	23543.05	*a* ^1^G_4_*—y* ${\;}^{3}F_{4}^{{}^{\circ}}$
125	4246.736	23540.87	*a* ^1^H_5_*—y* ${\;}^{1}H_{5}^{{}^{\circ}}$
30	4248.150	23533.03	*a* ^3^D_2_—*z* ${\;}^{3}S_{1}^{{}^{\circ}}$
3	4250.615	23519.39	*a* ^3^H_4_—*z* ${\;}^{1}H_{5}^{{}^{\circ}}$
2	4255.397	23492.96	*a* ^1^H_5_—*y* ${\;}^{3}H_{6}^{{}^{\circ}}$
10	4255.716	23491.20	*b* ^3^P_1_—*z* ${\;}^{3}P_{0}^{{}^{\circ}}$
2	4255.887	23490.25	*b* ^3^H_4_—*x* ${\;}^{1}H_{5}^{{}^{\circ}}$
200	4258.989	23473.14	*b* ^3^P_2_—*z* ${\;}^{3}P_{2}^{{}^{\circ}}$
100	4260.007	23467.53	*a* ^3^P_2_*—y* ${\;}^{5}D_{1}^{{}^{\circ}}$
1	4262.472	23453.96	*z* ${\;}^{7}D_{3}^{{}^{\circ}}$—*e* ^5^S_2_
30	4263.403	23448.84	*a* ^3^P_2_—*z* ${\;}^{5}P_{3}^{{}^{\circ}}$
40	4265.618	23436.66	*a* ^5^P_3_—*z* ${\;}^{3}D_{2}^{{}^{\circ}}$
4	4265.990	23434.62	*b* ^3^F_2_—*z* ${\;}^{5}P_{2}^{{}^{\circ}}$
2	4269.053	23417.81	*b* ^3^F_3_—*z* ${\;}^{5}P_{3}^{{}^{\circ}}$
6	4272.957	23396.41	*a* ^1^F_3_— $38_{4}^{{}^{\circ}}$
1	4274.639	23387.21	*a* ^1^D_2_—*y* ${\;}^{3}F_{2}^{{}^{\circ}}$
30	4277.272	23372.81	$\{\;\begin{array}{c} b{\;}^{3}P_{0}—x{\;}^{5}D_{1}^{{}^{\circ}}\\ a{\;}^{5}F_{3}—z{\;}^{7}D_{4}^{{}^{\circ}} \end{array}$
1	4278.272	23367.35	$\{\;\begin{array}{c} a{\;}^{5}F_{1}—z{\;}^{7}D_{2}^{{}^{\circ}}\\ c{\;}^{3}P_{2}—3_{1}^{{}^{\circ}} \end{array}$
30	4278.698	23365.02	*b* ^3^P_1_*—z* ${\;}^{3}P_{1}^{{}^{\circ}}$
30*H*	4281.941	23347.33	*a* ^3^H_6_—*z* ${\;}^{3}H_{6}^{{}^{\circ}}$
80	4282.219	23345.81	*a* ^3^F_4_—*z* ${\;}^{3}G_{4}^{{}^{\circ}}$
6	4282.972	23341.71	*b* ^3^G_3_— $50_{2}^{{}^{\circ}}$
1	4283.113	23340.94	*a* ^5^P_2_—*z* ${\;}^{7}P_{3}^{{}^{\circ}}$
400	4284.330	23334.31	*a* ^5^D_2_—*z* ${\;}^{3}F_{3}^{{}^{\circ}}$
200	4287.048	23319.51	*b* ^3^P_2_*—y* ${\;}^{3}D_{2}^{{}^{\circ}}$
30	4290.551	23300.47	*a* ^3^F_3_—*z* ${\;}^{7}P_{3}^{{}^{\circ}}$
6	4292.274	23291.12	*b* ^3^G_4_— $43_{3}^{{}^{\circ}}$
150	4293.284	23285.64	*a* ^5^D_2_—*z* ${\;}^{7}P_{2}^{{}^{\circ}}$
200	4294.793	23277.46	*a* ^5^D_3_—*z* ${\;}^{3}G_{3}^{{}^{\circ}}$
150	4295.932	23271.29	*b* ^3^F_4_—*z* ${\;}^{3}F_{3}^{{}^{\circ}}$
10	4296.694	23267.16	*a* ^1^P_1_*—u* ${\;}^{3}D_{2}^{{}^{\circ}}$
1000	4297.714	23261.64	*a* ^3^F_3_—*z* ${\;}^{5}G_{4}^{{}^{\circ}}$
10	4301.154	23243.04	*a* ^3^D_3_*—y* ${\;}^{3}F_{3}^{{}^{\circ}}$
400	4307.604	23208.23	*a* ^3^F_2_—*z* ${\;}^{3}F_{3}^{{}^{\circ}}$
1	4308.404	23203.92	*a* ^3^D_1_—*y* ${\;}^{3}P_{1}^{{}^{\circ}}$
50	4309.215	23199.56	*z* ${\;}^{7}D_{3}^{{}^{\circ}}$*—e* ^7^D_4_
16	4312.494	23181.92	*a* ^3^D_1_*—y* ${\;}^{3}P_{0}^{{}^{\circ}}$
1	4312.902	23179.72	*a* ^3^H_4_—*y* ${\;}^{3}G_{4}^{{}^{\circ}}$
60	4314.308	23172.17	*z* ${\;}^{7}D_{5}^{{}^{\circ}}$*—e* ^7^D_5_
50	4316.643	23159.64	*a* ^3^F_2_—*z* ${\;}^{7}P_{2}^{{}^{\circ}}$
150	4318.441	23150.00	*a* ^5^D_2_—*z* ${\;}^{3}D_{2}^{{}^{\circ}}$
10	4319.132	23146.29	*a* ^3^D_2_*—x* ${\;}^{5}D_{1}^{{}^{\circ}}$
150	4319.871	23142.33	*a* ^5^P_2_—*z* ${\;}^{5}S_{2}^{{}^{\circ}}$
8	4320.585	23138.51	*a* ^3^H_4_*—y* ${\;}^{3}G_{3}^{{}^{\circ}}$
8	4320.826	23137.22	*b* ^3^P_1_—*z* ${\;}^{3}P_{2}^{{}^{\circ}}$
20	4321.307	23134.64	*a* ^5^D_1_—*z* ${\;}^{3}D_{2}^{{}^{\circ}}$
20	4322.971	23125.74	*a* ^1^F_3_— $33_{2}^{{}^{\circ}}$
2*h*	4323.469	23123.07	*b* ^3^G_4_— $40_{3}^{{}^{\circ}}$
150	4325.059	23114.57	*b* ^3^P_2_*—y* ${\;}^{3}D_{3}^{{}^{\circ}}$
150	4326.822	23105.15	*a* ^3^P_2_*—y* ${\;}^{5}D_{2}^{{}^{\circ}}$
100	4327.425	23101.93	*a* ^3^F_3_—*z* ${\;}^{5}S_{2}^{{}^{\circ}}$
16	4328.563	23095.86	*a* ^3^P_1_—*z* ${\;}^{5}P_{2}^{{}^{\circ}}$
100	4331.168	23081.97	*a* ^5^P_3_— *z* ${\;}^{3}G_{3}^{{}^{\circ}}$
40	4332.505	23074.85	*b* ^1^D_2_—*y* ${\;}^{1}P_{1}^{{}^{\circ}}$
10	4332.649	23074.08	*b* ^3^F_3_—*y* ${\;}^{5}D_{2}^{{}^{\circ}}$
1	4335.125	23060.90	*a* ^3^D_1_—*y* ${\;}^{5}P_{1}^{{}^{\circ}}$
80	4336.431	23053.96	*a* ^5^D_4_—*z* ${\;}^{5}G_{3}^{{}^{\circ}}$
200	4337.268	23049.51	*a* ^3^F_4_—*z* ${\;}^{7}F_{4}^{{}^{\circ}}$
20	4338.675	23042.03	*c* ^3^P_2_—*x* ${\;}^{3}G_{3}^{{}^{\circ}}$
40	4340.351	23033.14	*a* ^3^H_5_—*z* ${\;}^{3}H_{4}^{{}^{\circ}}$
30*H*	4341.050	23029.43	*a* ^3^D_1_*—y* ${\;}^{3}P_{2}^{{}^{\circ}}$
400	4342.073	23024.00	*a* ^3^F_2_—*z* ${\;}^{3}D_{2}^{{}^{\circ}}$
100	4346.484	23000.64	*a* ^5^P_2_—*z* ${\;}^{3}D_{3}^{{}^{\circ}}$
200	4349.704	22983.61	*b* ^3^P_1_—*y* ${\;}^{3}D_{2}^{{}^{\circ}}$
250	4354.130	22960.25	*a* ^3^F_3_—*z* ${\;}^{3}D_{3}^{{}^{\circ}}$
100	4354.804	22956.69	*a* ^3^P_2_—*z* ${\;}^{3}D_{1}^{{}^{\circ}}$
1000	4361.204	22923.00	*a* ^3^F_4_—*z* ${\;}^{7}F_{5}^{{}^{\circ}}$
10	4362.707	22915.11	*a* ^5^P_2_—*z* ${\;}^{5}G_{2}^{{}^{\circ}}$
10	4364.108	22907.75	*c* ^3^P_2_—*u* ${\;}^{3}D_{1}^{{}^{\circ}}$
4	4368.761	22883.36	*a* ^1^H_5_*—x* ${\;}^{3}G_{4}^{{}^{\circ}}$
40	4370.418	22874.68	*a* ^3^F_3_—*z* ${\;}^{5}G_{2}^{{}^{\circ}}$
60	4371.209	22870.54	*a* ^1^D_2_*—y* ${\;}^{3}P_{1}^{{}^{\circ}}$
2000	4372.200	22865.36	*a* ^5^D_4_—*z* ${\;}^{3}F_{4}^{{}^{\circ}}$
30	4381.276	22817.99	*a* ^3^D_3_—*y* ${\;}^{5}P_{2}^{{}^{\circ}}$
60	4383.368	22807.10	*a* ^3^P_2_—*y* ${\;}^{5}D_{3}^{{}^{\circ}}$
400	4385.393	22796.57	*a* ^5^D_4_—*z* ${\;}^{5}G_{5}^{{}^{\circ}}$
400	4385.650	22795.23	*a* ^5^D_2_—*z* ${\;}^{3}G_{3}^{{}^{\circ}}$
80	4386.272	22792.00	*b* ^3^P_0_—*y* ${\;}^{3}D_{1}^{{}^{\circ}}$
1*h*	4388.106	22782.48	*c* ^3^F_3_—*x* ${\;}^{1}F_{3}^{{}^{\circ}}$
30	4388.998	22777.85	$\{\;\begin{array}{c} c{\;}^{3}F_{3}—y{\;}^{5}S_{2}^{{}^{\circ}}\\ b{\;}^{3}G_{3}—42_{4}^{{}^{\circ}} \end{array}$
500	4390.440	22770.36	*a* ^5^D_3_—*z* ${\;}^{5}G_{4}^{{}^{\circ}}$
100	4391.031	22767.30	*a* ^5^D_4_—*z* ${\;}^{7}P_{4}^{{}^{\circ}}$
30	4394.970	22746.90	*a* ^3^H_6_—*z* ${\;}^{3}H_{5}^{{}^{\circ}}$
2	4396.700	22737.94	*b* ^1^D_2_—*w* ${\;}^{5}D_{1}^{{}^{\circ}}$
150	4397.803	22732.24	*b* ^3^F_4_—*z* ${\;}^{3}G_{3}^{{}^{\circ}}$
1*h*	4398.733	22727.44	*a* ^1^D_2_—*y* ${\;}^{5}P_{1}^{{}^{\circ}}$
60	4399.597	22722.97	*a* ^5^P_1_—*z* ${\;}^{7}P_{2}^{{}^{\circ}}$
2	4402.286	22709.09	*z* ${\;}^{7}D_{1}^{{}^{\circ}}$—*e* ^5^S_2_
40	4404.815	22696.05	*a* ^1^D_2_—*y* ${\;}^{3}P_{2}^{{}^{\circ}}$
500	4410.028	22669.23	*a* ^3^F_2_—*z* ${\;}^{3}G_{3}^{{}^{\circ}}$
1	4412.324	22657.43	*a* ^3^H_5_—*z* ${\;}^{3}H_{6}^{{}^{\circ}}$
20	4413.300	22652.42	*a* ^1^P_1_—*w* ${\;}^{3}D_{1}^{{}^{\circ}}$
4	4414.995	22643.72	*b* ^3^F_2_—*y* ${\;}^{5}D_{1}^{{}^{\circ}}$
100	4420.852	22613.72	$\{\;\begin{array}{c} a{\;}^{3}D_{2}—x{\;}^{5}D_{2}^{{}^{\circ}}\\ a{\;}^{5}P_{3}—z{\;}^{7}P_{3}^{{}^{\circ}} \end{array}$
150	4421.456	22610.64	*a* ^5^D_3_—*z* ${\;}^{5}S_{2}^{{}^{\circ}}$
4	4422.978	22602.85	*b* ^1^D_2_— $9_{1}^{{}^{\circ}}$
50	4424.796	22593.57	*a* ^3^P_1_—*y* ${\;}^{5}D_{0}^{{}^{\circ}}$
4	4426.014	22587.35	*a* ^5^P_1_—*z* ${\;}^{3}D_{2}^{{}^{\circ}}$
250	4428.441	22574.97	*a* ^5^P_3_*—z* ${\;}^{5}G_{4}^{{}^{\circ}}$
4	4430.309	22565.45	$\{\;\begin{array}{c} a{\;}^{3}D_{2}—y{\;}^{3}D_{1}^{{}^{\circ}}\\ a{\;}^{3}G_{5}—y{\;}^{5}F_{5}^{{}^{\circ}} \end{array}$
1	4433.641	22548.49	*a* ^3^P_2_—*z* ${\;}^{3}F_{2}^{{}^{\circ}}$
2*H*	4438.343	22524.61	*b* ^1^D_2_— $8_{1}^{{}^{\circ}}$
10	4439.413	22519.18	*b* ^3^P_0_—*z* ${\;}^{3}P_{1}^{{}^{\circ}}$
250	4439.745	22517.50	*b* ^3^F_3_—*z* ${\;}^{3}F_{2}^{{}^{\circ}}$
10	4440.069	22515.85	*a* ^3^D_3_—*y* ${\;}^{5}P_{3}^{{}^{\circ}}$
150	4444.494	22493.44	*a* ^5^P_2_—*z* ${\;}^{5}G_{3}^{{}^{\circ}}$
400	4449.322	22469.03	*a* ^5^D_3_—*z* ${\;}^{3}D_{3}^{{}^{\circ}}$
1	4452.520	22452.89	*a* ^3^F_3_—*z* ${\;}^{5}G_{3}^{{}^{\circ}}$
800	4460.031	22415.08	*a* ^5^P_3_—*z* ${\;}^{5}S_{2}^{{}^{\circ}}$
8	4461.472	22407.84	*a* ^5^D_4_—*z* ${\;}^{3}G_{4}^{{}^{\circ}}$
10	4465.483	22387.71	*a* ^1^D_2_—*y* ${\;}^{3}F_{3}^{{}^{\circ}}$
30	4466.352	22383.36	*a* ^5^D_3_—*z* ${\;}^{5}G_{2}^{{}^{\circ}}$
40	4467.257	22378.82	*a* ^1^P_1_—*z* ${\;}^{1}S_{0}^{{}^{\circ}}$
6	4471.018	22360.00	*b* ^3^H_4_*—y* ${\;}^{1}G_{4}^{{}^{\circ}}$
4	4472.257	22353.80	*a* ^3^H_4_—*y* ${\;}^{3}G_{5}^{{}^{\circ}}$
30	4473.324	22348.47	*a* ^1^H_5_—*z* ${\;}^{1}I_{6}^{{}^{\circ}}$
150	4473.909	22345.55	*a* ^3^F_4_—*z* ${\;}^{5}F_{3}^{{}^{\circ}}$
2	4475.640	22336.91	$\{\;\begin{array}{c} b{\;}^{1}D_{2}—w{\;}^{5}D_{2}^{{}^{\circ}}\\ a{\;}^{3}H_{4}—y{\;}^{2}F_{3}^{{}^{\circ}} \end{array}$
2	4475.880	22335.71	*z* ${\;}^{5}F_{4}^{{}^{\circ}}$—*f* ^5^D_4_
4*h*	4476.475	22332.74	*c* ^3^F_4_—*x* ${\;}^{3}G_{3}^{{}^{\circ}}$
4	4477.97	22325.28	*a* ^1^F_3_— $22_{3}^{{}^{\circ}}$
60	4479.393	22318.19	*b* ^3^P_2_—*y* ${\;}^{5}F_{2}^{{}^{\circ}}$
150	4480.434	22313.01	*a* ^3^D_2_—*x* ${\;}^{5}D_{3}^{{}^{\circ}}$
60	4482.031	22305.06	*a* ^3^P_1_—*y* ${\;}^{5}D_{1}^{{}^{\circ}}$
1	4483.824	22296.14	*a* ^3^D_1_—*y* ${\;}^{5}P_{2}^{{}^{\circ}}$
1	4484.674	22291.91	*a* ^1^P_1_—*w* ${\;}^{3}D_{2}^{{}^{\circ}}$
1	4486.801	22281.34	*b* ^3^F_2_—*y* ${\;}^{5}D_{2}^{{}^{\circ}}$
150	4488.377	22273.52	*a* ^5^P_3_—*z* ${\;}^{3}D_{3}^{{}^{\circ}}$
140	4490.220	22264.38	*a* ^3^F_3_—*z* ${\;}^{3}F_{4}^{{}^{\circ}}$
100	4491.674	22257.17	*b* ^3^P_1_—*y* ${\;}^{5}F_{1}^{{}^{\circ}}$
1	4492.920	22251.00	*b* ^3^G_3_— $33_{2}^{{}^{\circ}}$
1	4496.780	22231.90	*d* ^3^F_2_— $45_{1}^{{}^{\circ}}$
200	4498.138	22225.19	*b* ^3^F_4_—*z* ${\;}^{5}G_{4}^{{}^{\circ}}$
40	4508.043	22176.35	*a* ^3^H_4_—*z* ${\;}^{3}H_{4}^{{}^{\circ}}$
60	4508.553	22173.85	$\{\;\begin{array}{c} a{\;}^{1}P_{1}—z{\;}^{1}P_{1}^{{}^{\circ}}\\ c{\;}^{3}P_{2}—y{\;}^{3}S_{1}^{{}^{\circ}} \end{array}$
160	4510.082	22166.33	*a* ^3^F_3_—*z* ${\;}^{7}P_{4}^{{}^{\circ}}$
140	4511.183	22160.92	*b* ^3^P_2_—*y* ${\;}^{5}F_{3}^{{}^{\circ}}$
2	4514.83	22143.02	*b* ^3^H_5_—*y* ${\;}^{3}H_{4}^{{}^{\circ}}$
50	4516.272	22135.95	*b* ^1^G_4_—*x* ${\;}^{1}G_{4}^{{}^{\circ}}$
200	4516.877	22132.98	*b* ^3^F_2_—*z* ${\;}^{3}D_{1}^{{}^{\circ}}$
160	4517.794	22128.49	*a* ^5^D_2_—*z* ${\;}^{5}S_{2}^{{}^{\circ}}$
160	4520.931	22113.14	*a* ^5^D_1_—*z* ${\;}^{5}S_{2}^{{}^{\circ}}$
2*h*	4525.931	22088.71	*b* ^3^H_5_—*x* ${\;}^{5}F_{4}^{{}^{\circ}}$
4	4529.044	22073.53	*z* ${\;}^{5}D_{4}^{{}^{\circ}}$—*e* ^7^D_5_
200	4530.860	22064.68	$\{\;\begin{array}{c} c{\;}^{3}P_{2}—y{\;}^{1}F_{3}^{{}^{\circ}}\\ a{\;}^{3}D_{2}—z{\;}^{3}P_{2}^{{}^{\circ}} \end{array}$
4*h*	4531.78	22060.20	*a* ^1^G_4_—*y* ${\;}^{3}D_{3}^{{}^{\circ}}$
10	4532.443	22056.97	*a* ^3^H_5_—*z* ${\;}^{3}H_{5}^{{}^{\circ}}$
1	4533.301	22052.80	*a* ^3^D_3_—*y* ${\;}^{3}F_{4}^{{}^{\circ}}$
2	4534.480	22047.06	$\{\;\begin{array}{c} z{\;}^{5}D_{3}^{{}^{\circ}}—e{\;}^{5}G_{3}\\ a{\;}^{1}F_{3}—w{\;}^{3}F_{2}^{{}^{\circ}} \end{array}$
50	4535.584	22041.70	*a* ^5^P_2_—*z* ${\;}^{7}F_{1}^{{}^{\circ}}$
10	4542.430	22008.48	*c* ^3^P_1_— $3_{1}^{{}^{\circ}}$
150	4543.690	22002.37	*a* ^3^F_2_—*z* ${\;}^{5}S_{2}^{{}^{\circ}}$
8	4545.713	21992.58	*b* ^3^G_4_— $23_{3}^{{}^{\circ}}$
125	4546.930	21986.70	*a* ^5^D_2_—*z* ${\;}^{3}D_{3}^{{}^{\circ}}$
200	4547.278	21985.02	*a* ^5^D_4_—*z* ${\;}^{7}F_{5}^{{}^{\circ}}$
150	4547.850	21982.25	*b* ^3^P_1_—*y* ${\;}^{5}F_{2}^{{}^{\circ}}$
80	4549.423	21974.65	*a* ^5^P_2_—*z* ${\;}^{7}F_{2}^{{}^{\circ}}$
120	4549.940	21972.15	*b* ^1^G_4_—*y* ${\;}^{1}G_{4}^{{}^{\circ}}$
200	4552.118	21961.64	*a* ^5^D_3_—*z* ${\;}^{5}G_{3}^{{}^{\circ}}$
1500	4554.514	21950.09	*a* ^3^F_4_—*z* ${\;}^{3}G_{5}^{{}^{\circ}}$
10	4556.064	21942.62	*a* ^3^P_1_—*y* ${\;}^{5}D_{2}^{{}^{\circ}}$
6	4557.811	21934.21	*a* ^3^F_3_—*z* ${\;}^{7}F_{2}^{{}^{\circ}}$
200	4559.982	21923.77	*b* ^3^F_4_—*z* ${\;}^{3}D_{3}^{{}^{\circ}}$
50	4562.601	21911.18	*a* ^3^D_2_—*y* ${\;}^{3}D_{2}^{{}^{\circ}}$
100	4564.693	21901.14	*a* ^5^D_2_—*z* ${\;}^{5}G_{2}^{{}^{\circ}}$
1	4566.681	21891.61	*c* ^3^F_2_—*x* ${\;}^{3}P_{2}^{{}^{\circ}}$
2	4567.885	21885.84	*a* ^5^D_1_—*z* ${\;}^{5}G_{2}^{{}^{\circ}}$
10	4570.241	21874.55	*a* ^3^D_1_—*z* ${\;}^{3}S_{1}^{{}^{\circ}}$
4	4575.738	21848.28	*a* ^5^P_2_—*z* ${\;}^{7}F_{3}^{{}^{\circ}}$
2	4576.303	21845.58	*a* ^1^F_3_— $16_{3}^{{}^{\circ}}$
150	4580.068	21827.62	*a* ^3^P_0_—*z* ${\;}^{3}D_{1}^{{}^{\circ}}$
1000	4584.440	21806.81	*a* ^3^F_3_—*z* ${\;}^{3}G_{4}^{{}^{\circ}}$
60	4587.099	21794.16	*a* ^3^P_1_—*z* ${\;}^{3}D_{1}^{{}^{\circ}}$
15	4589.574	21782.41	*b* ^3^G_4_— $21_{5}^{{}^{\circ}}$
2*h*	4590.727	21776.94	*z* ${\;}^{7}D_{5}^{{}^{\circ}}$—*e* ^5^G_6_
200	4591.097	21775.19	*a* ^3^F_2_—*z* ${\;}^{5}G_{2}^{{}^{\circ}}$
100	4591.546	21773.06	*a* ^5^D_3_—*z* ${\;}^{3}F_{4}^{{}^{\circ}}$
6	4592.005	21770.88	*z* ${\;}^{5}G_{2}^{{}^{\circ}}$—*f* ^7^D_1_
200	4592.513	21768.47	*a* ^3^P_2_—*z* ${\;}^{3}F_{3}^{{}^{\circ}}$
10	4592.995	21766.19	a ^5^P_3_—*z* ${\;}^{5}G_{3}^{{}^{\circ}}$
10	4593.195	21765.24	*a* ^1^P_1_*—x* ${\;}^{3}D_{2}^{{}^{\circ}}$
1	4594.641	21758.39	*z* ${\;}^{7}D_{5}^{{}^{\circ}}$*—e* ^5^D_4_
150	4596.703	21748.63	*b* ^1^D_2_*—x* ${\;}^{1}F_{3}^{{}^{\circ}}$
1	4597.331	21745.66	*a* ^1^F_3_—*w* ${\;}^{3}F_{3}^{{}^{\circ}}$
1	4597.697	21743.93	*b* ^1^D_2_*—y* ${\;}^{5}S_{2}^{{}^{\circ}}$
500	4599.082	21737.38	*b* ^3^F_3_—*z* ${\;}^{3}F_{3}^{{}^{\circ}}$
150	4601.757	21724.74	*b* ^3^F_2_—*z* ${\;}^{3}F_{2}^{{}^{\circ}}$
30	4602.820	21719.73	*a* ^3^P_2_—*z* ${\;}^{7}P_{2}^{{}^{\circ}}$
150	4605.671	21706.28	*a* ^3^D_2_*—y* ${\;}^{3}D_{3}^{{}^{\circ}}$
2	4608.673	21692.14	*z* ${\;}^{7}D_{5}^{{}^{\circ}}$—*f* ^5^F_5_
10	4610.492	21683.59	*a*^1^H_5_—*x* ${\;}^{3}G_{5}^{{}^{\circ}}$
60	4612.328	21674.95	*a* ^5^D_3_—*z* ${\;}^{7}P_{4}^{{}^{\circ}}$
2	4615.425	21660.41	*a* ^1^D_2_—*y* ${\;}^{5}P_{3}^{{}^{\circ}}$
2	4616.093	21657.28	*b* ^3^H_4_*—y* ${\;}^{3}H_{4}^{{}^{\circ}}$
30	4617.662	21649.92	*a* ^5^P_2_—*z* ${\;}^{5}F_{1}^{{}^{\circ}}$
4*h*	4618.024	21648.22	*d* ^3^F_3_— $47_{4}^{{}^{\circ}}$
6	4618.799	21644.59	*d* ^3^F_4_— $43_{3}^{{}^{\circ}}$
8	4624.322	21618.74	*b* ^3^G_4_— $19_{4}^{{}^{\circ}}$
4	4624.464	21618.07	*b* ^1^D_2_—*w* ${\;}^{5}D_{3}^{{}^{\circ}}$
20	4626.035	21610.73	*c* ^3^P_1_—*y* ${\;}^{1}D_{2}^{{}^{\circ}}$
30	4628.337	21599.98	*c* ^3^P_2_—*w* ${\;}^{3}D_{2}^{{}^{\circ}}$
10	4630.442	21590.16	$\left\{ \begin{array}{l} \;b{\;}^{3}H_{5}—y{\;}^{3}H_{5}^{{}^{\circ}}\\ \;z{\;}^{5}F_{4}^{{}^{\circ}}—e{\;}^{5}G_{3} \end{array}\right.$
1	4631.74	21584.11	*a* ^3^P_2_*—z* ${\;}^{3}D_{2}^{{}^{\circ}}$
2	4633.173	21577.44	*a* ^5^P_3_—*z* ${\;}^{3}F_{4}^{{}^{\circ}}$
300	4635.682	21565.76	*a* ^5^P_1_—*z* ${\;}^{5}S_{2}^{{}^{\circ}}$
20*h*	4638.419	21553.04	*b* ^3^F_3_—*z* ${\;}^{3}D_{2}^{{}^{\circ}}$
20	4639.318	21548.86	c ^3^P_1_*—u* ${\;}^{3}D_{1}^{{}^{\circ}}$
30	4640.983	21541.13	*a* ^1^D_2_—*z* ${\;}^{3}S_{1}^{{}^{\circ}}$
200	4645.09	21522.08	*a* ^1^H_5_*—z* ${\;}^{3}I_{6}^{{}^{\circ}}$
6	4646.810	21514.12	*a* ^1^P_1_*—z* ${\;}^{1}D_{2}^{{}^{\circ}}$
600	4647.594	21510.49	*a* ^3^F_3_—*z* ${\;}^{7}F_{4}^{{}^{\circ}}$
60	4648.158	21507.88	*z* ${\;}^{7}D_{4}^{{}^{\circ}}$*—e* ^5^D_4_
4	4650.211	21498.38	*c* ^3^F_2_—*u* ${\;}^{3}D_{1}^{{}^{\circ}}$
4	4652.498	21487.81	*a* ^3^D_1_—*x* ${\;}^{5}D_{1}^{{}^{\circ}}$
2*h*	4653.322	21484.01	*c* ^3^F_3_—*y* ${\;}^{1}D_{2}^{{}^{\circ}}$
70	4653.759	21481.99	*c* ^3^P_2_—*z* ${\;}^{1}P_{1}^{{}^{\circ}}$
400	4654.311	21479.44	$\left\{ \begin{array}{l} \;a{\;}^{5}D_{2}—z{\;}^{5}G_{3}^{{}^{\circ}}\\ \;a{\;}^{5}P_{3}—z{\;}^{7}P_{4}^{{}^{\circ}} \end{array}\right.$
50	4654.780	21477.28	*a* ^3^D_3_—*x* ${\;}^{5}D_{2}^{{}^{\circ}}$
60	4656.415	21469.74	*a* ^3^F_4_—*z* ${\;}^{5}F_{4}^{{}^{\circ}}$
4	4660.640	21450.28	*b* ^3^G_3_— $22_{3}^{{}^{\circ}}$
6	4662.254	21442.85	*a* ^5^D_3_—*z* ${\;}^{7}F_{2}^{{}^{\circ}}$
20	4662.494	21441.75	*z* ${\;}^{7}D_{4}^{{}^{\circ}}$*—f* ^5^F_5_
100	4669.139	21411.23	*b* ^3^P_0_—*y* ${\;}^{5}F_{1}^{{}^{\circ}}$
250	4669.973	21407.41	*a* ^5^D_4_—*z* ${\;}^{5}F_{3}^{{}^{\circ}}$
6	4671.368	21401.02	*b* ^3^P_2_— *z* ${\;}^{5}P_{1}^{{}^{\circ}}$
300	4674.638	21386.05	*a* ^3^P_1_—*z* ${\;}^{3}F_{2}^{{}^{\circ}}$
40	4681.408	21355.12	*c* ^3^F_4_—*y* ${\;}^{1}F_{3}^{{}^{\circ}}$
400	4681.780	21353.42	*a* ^3^F_2_—*z* ${\;}^{5}G_{3}^{{}^{\circ}}$
300	4684.013	21343.24	*a* ^3^F_3_—*z* ${\;}^{5}F_{2}^{{}^{\circ}}$
2	4685.052	21338.51	*a* ^5^P_1_—*z* ${\;}^{5}G_{2}^{{}^{\circ}}$
400	4690.114	21315.48	*a* ^5^D_3_—*z* ${\;}^{3}G_{4}^{{}^{\circ}}$
2*h*	4692.912	21302.77	*z* ${\;}^{5}D_{3}^{{}^{\circ}}$—*f* ^5^F_3_
10	4695.253	21292.15	*z* ${\;}^{5}D_{2}^{{}^{\circ}}$*—e* ^3^D_2_
1	4698.700	21276.53	*z* ${\;}^{5}F_{4}^{{}^{\circ}}$—*e* ^5^P_3_
2	4700.261	21269.46	*z* ${\;}^{5}S_{2}^{{}^{\circ}}$—D_3_
1	4705.16	21247.32	*a* ^5^P_3_—*z* ${\;}^{7}F_{2}^{{}^{\circ}}$
1000	4709.482	21227.82	*b* ^3^F_4_—*z* ${\;}^{3}F_{4}^{{}^{\circ}}$
20	4710.968	21221.12	*z* ${\;}^{5}D_{3}^{{}^{\circ}}$—*e* ^3^G_4_
80	4711.988	21216.53	*c* ^3^P_1_—*u* ${\;}^{3}D_{2}^{{}^{\circ}}$
16*h*	4712.502	21214.21	*z* ${\;}^{5}D_{3}^{{}^{\circ}}$—*e* ^5^D_2_
10	4713.370	21210.31	*z* ${\;}^{5}D_{2}^{{}^{\circ}}$*—e* ^3^G_3_
1	4714.834	21203.72	*d* ^3^F_4_— $35_{3}^{{}^{\circ}}$
6	4715.615	21200.21	*a* ^3^H_4_*—z* ${\;}^{3}H_{5}^{{}^{\circ}}$
100	4716.049	21198.26	*b* ^3^F_3_—*z* ${\;}^{3}G_{3}^{{}^{\circ}}$
6	4718.840	21185.72	*b* ^3^G_3_— $19_{4}^{{}^{\circ}}$
120	4720.920	21176.39	*a* ^3^D_3_—*x* ${\;}^{5}D_{3}^{{}^{\circ}}$
1	4721.848	21172.23	*b* ^3^G_3_—*w* ${\;}^{3}F_{2}^{{}^{\circ}}$
10	4723.226	21166.05	*c* ^3^F_2_*—u* ${\;}^{3}D_{2}^{{}^{\circ}}$
40	4724.799	21159.00	*b* ^3^F_4_—*z* ${\;}^{5}G_{5}^{{}^{\circ}}$
4	4727.584	21146.54	*a* ^3^H_4_*—y* ${\;}^{3}F_{4}^{{}^{\circ}}$
300	4731.323	21129.83	*b* ^3^F_4_—*z* ${\;}^{7}P_{4}^{{}^{\circ}}$
150	4733.319	21120.92	*a* ^5^P_3_—*z* ${\;}^{7}F_{3}^{{}^{\circ}}$
300	4733.520	21120.02	*a* ^5^P_3_—*z* ${\;}^{3}G_{4}^{{}^{\circ}}$
10	4740.332	21089.67	$\left\{ \begin{array}{l} \;a{\;}^{3}D_{1}—x{\;}^{5}D_{0}^{{}^{\circ}}\\ \;c{\;}^{3}F_{3}—u{\;}^{3}D_{2}^{{}^{\circ}} \end{array}\right.$
125	4743.028	21077.68	*b* ^3^H_5_*—y* ${\;}^{1}H_{5}^{{}^{\circ}}$
2	4743.670	21074.83	*a* ^5^P_2_—*z* ${\;}^{5}D_{1}^{{}^{\circ}}$
2	4743.984	21073.44	*c* ^3^P_2_*—x* ${\;}^{3}D_{2}^{{}^{\circ}}$
4	4744.654	21070.46	2 ${\;}^{7}P_{3}^{{}^{\circ}}$—D_3_
1	4745.859	21065.11	*b* ^3^P_1_*—z* ${\;}^{5}P_{1}^{{}^{\circ}}$
2	4749.803	21047.62	*z* ${\;}^{5}D_{2}^{{}^{\circ}}$—*e* ^5^D_1_
30	4751.015	21042.25	*a* ^5^D_1_*—z* ${\;}^{7}F_{0}^{{}^{\circ}}$
4*h*	4751.454	21040.30	*z* ${\;}^{7}F_{2}^{{}^{\circ}}$*—f* ^7^D_2_
30	4752.140	21037.27	*z* ${\;}^{5}D_{3}^{{}^{\circ}}$*—f* ^5^F_4_
6	4753.844	21029.73	*b* ^3^H_5_—*y* ${\;}^{3}H_{6}^{{}^{\circ}}$
250	4756.244	21019.12	*a* ^5^D_3_*—z* ${\;}^{7}F_{4}^{{}^{\circ}}$
500	4757.844	21012.05	*a* ^5^D_4_—*z* ${\;}^{3}G_{5}^{{}^{\circ}}$
6	4761.533	20995.77	*d* ^3^P_2_*—t* ${\;}^{3}D_{2}^{{}^{\circ}}$
150	4764.406	20983.11	2 ${\;}^{5}D_{3}^{{}^{\circ}}$—*e* ^3^D_3_
4	4766.647	20973.24	2 ${\;}^{7}F_{1}^{{}^{\circ}}$—*f* ^7^D_2_
60	4767.153	20971.02	*b* ^3^H_4_*—x* ${\;}^{3}G_{3}^{{}^{\circ}}$
300	4769.306	20961.55	*a* ^3^F_4_*—z* ${\;}^{5}D_{3}^{{}^{\circ}}$
125	4773.155	20944.65	*b* ^3^F_2_—*z* ${\;}^{3}F_{3}^{{}^{\circ}}$
125	4774.004	20940.92	*a* ^1^P_1_*—x* ${\;}^{3}F_{2}^{{}^{\circ}}$
15	4781.112	20909.79	*a* ^3^D_2_*—y* ${\;}^{5}F_{2}^{{}^{\circ}}$
50	4781.770	20906.91	*a* ^3^D_1_—*y* ${\;}^{3}D_{1}^{{}^{\circ}}$
20	4783.300	20900.23	*b* ^3^P_1_—*z* ${\;}^{5}P_{2}^{{}^{\circ}}$
150	4784.266	20896.01	*b* ^3^F_2_—*z* ${\;}^{7}P_{2}^{{}^{\circ}}$
1	4785.079	20892.46	*b* ^3^G_5_—*x* ${\;}^{1}H_{5}^{{}^{\circ}}$
10	4787.357	20882.51	*z* ${\;}^{7}F_{5}^{{}^{\circ}}$—*f* ^5^D_4_
4*h*	4790.809	20867.47	*z* ${\;}^{5}F_{3}^{{}^{\circ}}$—*e* ^3^D_2_
150	4794.386	20851.90	*a* ^5^D_3_*—z* ${\;}^{5}F_{2}^{{}^{\circ}}$
150	4795.575	20846.73	*a* ^5^P_2_*—z* ${\;}^{5}F_{3}^{{}^{\circ}}$
150	4798.450	20834.24	*a* ^5^D_2_—*z* ${\;}^{7}F_{3}^{{}^{\circ}}$
60	4801.180	20822.39	*c* ^3^P_2_*—z* ${\;}^{1}D_{2}^{{}^{\circ}}$
20	4802.91	20814.89	*c* ^3^P_1_*—y* ${\;}^{3}S_{1}^{{}^{\circ}}$
200	4804.887	20806.33	*a* ^3^F_3_—*z* ${\;}^{5}F_{3}^{{}^{\circ}}$
4	4807.633	20794.45	*z* ${\;}^{5}F_{4}^{{}^{\circ}}$—*f* ^5^F_3_
20	4809.714	20785.45	*z* ${\;}^{5}F_{3}^{{}^{\circ}}$—*e* ^3^G_3_
4	4810.693	20781.22	*b* ^1^D_2_*—x* ${\;}^{3}P_{2}^{{}^{\circ}}$
6	4812.225	20774.60	$\left\{ \begin{array}{l} \;a{\;}^{3}D_{3}—y{\;}^{3}D_{2}^{{}^{\circ}}\\ \;a{\;}^{1}P_{1}—x{\;}^{3}D_{1}^{{}^{\circ}} \end{array}\right.$
6	4812.849	20771.91	*z* ${\;}^{5}D_{4}^{{}^{\circ}}$—*e* ^3^G_5_
20	4813.231	20770.26	*b* ^3^F_4_—*z* ${\;}^{3}G_{4}^{{}^{\circ}}$
200	4815.531	20760.34	$\left\{ \begin{array}{l} \;b{\;}^{3}F_{2}—z{\;}^{3}D_{2}^{{}^{\circ}}\\ \;a{\;}^{3}D_{1}—z{\;}^{3}P_{0}^{{}^{\circ}} \end{array}\right.$
60	4817.353	20752.49	*a* ^3^D_2_*—y* ${\;}^{5}F_{3}^{{}^{\circ}}$
2	4820.251	20740.01	*z* ${\;}^{3}G_{5}^{{}^{\circ}}$—*e* ^7^D_4_
80	4822.584	20729.98	*b* ^3^F_3_—*z* ${\;}^{7}P_{3}^{{}^{\circ}}$
2	4824.356	20722.36	*a* ^1^F_3_—*t* ${\;}^{3}D_{2}^{{}^{\circ}}$
4	4825.751	20716.38	*b* ^1^G_4_—*y* ${\;}^{3}H_{5}^{{}^{\circ}}$
30*h*	4826.207	20714.42	*z* ${\;}^{5}F_{3}^{{}^{\circ}}$*—e* ^5^G_4_
30*h*	4826.567	20712.87	*z* ${\;}^{5}F_{4}^{{}^{\circ}}$—*e* ^3^G_4_
4*h*	4827.648	20708.24	*a* ^3^F_2_—*z* ${\;}^{7}F_{3}^{{}^{\circ}}$
40	4828.683	20703.80	*c* ^3^F_4_*—x* ${\;}^{3}F_{4}^{{}^{\circ}}$
2	4831.633	20691.16	*b* ^3^F_3_—*z* ${\;}^{5}G_{4}^{{}^{\circ}}$
1	4836.412	20670.71	*b* ^3^G_3_—*x* ${\;}^{5}P_{3}^{{}^{\circ}}$
40	4838.161	20663.24	*z* ${\;}^{5}F_{3}^{{}^{\circ}}$*—e* ^5^G_3_
150	4839.014	20659.60	*z* ${\;}^{5}D_{4}^{{}^{\circ}}$—*e* ^5^D_4_
125	4839.760	20656.41	*a* ^5^P_3_*—z* ${\;}^{5}F_{2}^{{}^{\circ}}$
10	4841.756	20647.90	*z* ${\;}^{7}F_{3}^{{}^{\circ}}$—*e* ^7^D_2_
10	4843.767	20639.32	*z* ${\;}^{5}D_{1}^{{}^{\circ}}$—*e* ^3^D_2_
200	4844.560	20635.94	*a* ^5^D_2_—*z* ${\;}^{5}F_{1}^{{}^{\circ}}$
6	4845.899	20630.24	*a* ^3^G_4_—*y* ${\;}^{5}D_{4}^{{}^{\circ}}$
10	4847.871	20621.85	*a* ^1^D_2_*—x* ${\;}^{5}D_{2}^{{}^{\circ}}$
15	4848.166	20620.60	*a* ^5^D_1_*—z* ${\;}^{5}F_{1}^{{}^{\circ}}$
6	4851.935	20604.58	*c* ^3^P_1_—*v* ${\;}^{3}D_{2}^{{}^{\circ}}$
1	4852.60	20601.75	*c* ^3^P_1_*—w* ${\;}^{3}D_{1}^{{}^{\circ}}$
2	4852.898	20600.49	*z* ${\;}^{7}F_{2}^{{}^{\circ}}$*—e* ^7^D_1_
150	4854.559	20593.44	*z* ${\;}^{5}D_{4}^{{}^{\circ}}$—*f* ^5^F_5_
2	4857.990	20578.90	*c* ^3^F_3_—*y* ${\;}^{1}F_{3}^{{}^{\circ}}$
2	4859.245	20573.58	*a* ^1^D_2_*—y* ${\;}^{3}D_{1}^{{}^{\circ}}$
8	4860.157	20569.72	*a* ^3^D_3_—*y* ${\;}^{3}D_{3}^{{}^{\circ}}$
150	4861.865	20562.50	*a* ^3^P_2_—*z* ${\;}^{5}S_{2}^{{}^{\circ}}$
60	4863.105	20557.25	*a* ^3^P_1_*—z* ${\;}^{7}P_{2}^{{}^{\circ}}$
75	4865.090	20548.87	*c* ^3^P_2_*—w* ${\;}^{3}D_{3}^{{}^{\circ}}$
1000	4869.163	20531.68	*a* ^5^D_4_—*z* ${\;}^{5}F_{4}^{{}^{\circ}}$
100	4869.782	20529.07	*z* ${\;}^{5}F_{4}^{{}^{\circ}}$—*f* ^5^F_4_
1*h*	4871.368	20522.38	*b* ^1^D_2_—*x* ${\;}^{3}G_{3}^{{}^{\circ}}$
4	4871.588	20521.46	*z* ${\;}^{7}F_{2}^{{}^{\circ}}$—*e* ^7^D_2_
150	4874.329	20509.92	*a* ^3^F_2_—*z* ${\;}^{5}F_{1}^{{}^{\circ}}$
160	4875.020	20507.01	*z* ${\;}^{5}F_{4}^{{}^{\circ}}$—*e* ^5^G_5_
50	4877.414	20496.94	*a* ^5^D_1_*—z* ${\;}^{5}D_{0}^{{}^{\circ}}$
30	4877.885	20494.96	*a* ^5^P_1_*—z* ${\;}^{7}F_{0}^{{}^{\circ}}$
50	4882.654	20474.95	*z* ${\;}^{5}F_{4}^{{}^{\circ}}$—*e* ^3^D_3_
40	4885.016	20465.05	*a* ^5^P_1_—*z* ${\;}^{7}F_{1}^{{}^{\circ}}$
6*h*	4886.347	20459.47	*b* ^3^H_5_*—z* ${\;}^{3}I_{5}^{{}^{\circ}}$
1	4887.538	20454.49	*z* ${\;}^{7}F_{1}^{{}^{\circ}}$*—e* ^7^D_2_
30	4888.605	20450.02	*b* ^7^D_2_—*y* ${\;}^{1}D_{2}^{{}^{\circ}}$
10*h*	4892.824	20432.39	*z* ${\;}^{7}F_{6}^{{}^{\circ}}$—C_5_
4	4894.208	20426.61	*b* ^3^P_2_—*z* ${\;}^{5}P_{3}^{{}^{\circ}}$
200	4895.340	20421.89	*a* ^5^P_2_—*z* ${\;}^{5}D_{2}^{{}^{\circ}}$
400	4895.609	20420.77	*a* ^3^P_2_—*z* ${\;}^{3}D_{3}^{{}^{\circ}}$
200	4899.255	20405.57	*b* ^3^F_2_—z ${\;}^{3}G_{3}^{{}^{\circ}}$
100	4901.071	20398.01	$\left\{ \begin{array}{l} \;a{\;}^{5}P_{1}—z{\;}^{7}F_{2}^{{}^{\circ}}\\ \;b^{3}\;P_{1}—y{\;}^{5}D_{0}^{{}^{\circ}} \end{array}\right.$
80	4901.855	20394.75	*z* ${\;}^{5}D_{1}^{{}^{\circ}}$*—e* ^5^D_2_
600	4903.066	20389.71	*b* ^3^F_3_—z ${\;}^{3}D_{3}^{{}^{\circ}}$
60*h*	4904.593	20383.36	*a* ^1^H_5_— $1_{4}^{{}^{\circ}}$
150	4905.030	20381.55	*a* ^3^F_3_—*z* ${\;}^{5}D_{2}^{{}^{\circ}}$
200	4907.895	20369.65	*a* ^5^D_2_—*z* ${\;}^{5}F_{2}^{{}^{\circ}}$
100	4911.598	20354.29	*a* ^5^D_1_*—z* ${\;}^{5}F_{2}^{{}^{\circ}}$
60	4913.278	20347.33	*b* ^3^F_4_—*z* ${\;}^{7}F_{5}^{{}^{\circ}}$
80	4914.195	20343.53	*z* ${\;}^{5}D_{2}^{{}^{\circ}}$—*f* ^5^F_3_
20	4917.341	20330.52	*z* ${\;}^{5}F_{2}^{{}^{\circ}}$*—e* ^3^D_2_
6	4918.369	20326.27	*c* ^3^P_2_*—x* ${\;}^{3}F_{3}^{{}^{\circ}}$
4	4919.646	20320.99	*a* ^1^D_2_—*x* ${\;}^{5}D_{3}^{{}^{\circ}}$
500	4921.081	20315.07	*a* ^5^D_3_—*z* ${\;}^{5}F_{3}^{{}^{\circ}}$
2	4923.725	20304.16	*b* ^3^F_3_—*z* ${\;}^{5}G_{2}^{{}^{\circ}}$
2	4924.518	20300.89	*a* ^1^D_2_—*z* ${\;}^{3}P_{1}^{{}^{\circ}}$
4	4930.934	20274.48	*d* ^3^P_2_—*x* ${\;}^{1}F_{3}^{{}^{\circ}}$
1	4931.73	20271.20	*a* ^3^F_4_—*z* ${\;}^{5}F_{5}^{{}^{\circ}}$
1	4932, 37	20268.57	*z* ${\;}^{5}F_{5}^{{}^{\circ}}$*—e* ^3^G_5_
10	4934.613	20259.36	*z* ${\;}^{5}F_{1}^{{}^{\circ}}$*—F*_1_
150	4935.640	20255.14	*z* ${\;}^{5}D_{2}^{{}^{\circ}}$*—e* ^5^D_2_
50	4936.244	20252.67	*a* ^3^D_1_*—y* ${\;}^{3}D_{2}^{{}^{\circ}}$
125	4937.219	20248.67	2 ${\;}^{5}F_{2}^{{}^{\circ}}$*—e* ^3^G_3_
200	4938.444	20243.64	*a* ^3^F_2_*—z* ${\;}^{5}F_{2}^{{}^{\circ}}$
1	4940.131	20236.73	*d* ^3^F_4_— $22_{3}^{{}^{\circ}}$
20	4941.575	20230.82	*d* ^3^F_2_— $16_{3}^{{}^{\circ}}$
60	4944.006	20220.87	*a* ^1^H_5_*—y* ${\;}^{3}G_{4}^{{}^{\circ}}$
20	4944.404	20219.24	*b* ^3^P_0_— *z* ${\;}^{5}P_{1}^{{}^{\circ}}$
2	4951.26	20191.24	*a* ^1^P_1_*—y* ${\;}^{3}F_{2}^{{}^{\circ}}$
200	4955.272	20174.90	*z* ${\;}^{5}F_{5}^{{}^{\circ}}$—*e* ^5^G_6_
150	4959.872	20156.19	$\left\{ \begin{array}{l} \;z{\;}^{5}F_{5}^{{}^{\circ}}—e{\;}^{5}D_{4}\\ \;z{\;}^{7}F_{5}^{{}^{\circ}}—f{\;}^{7}D_{4} \end{array}\right.$
1	4962.872	20144.00	*d* ^3^P_2_*—w* ${\;}^{5}D_{3}^{{}^{\circ}}$
15	4964.885	20135.83	*d* ^3^F_4_— $21_{5}^{{}^{\circ}}$
10	4966.109	20130.87	*d* ^3^F_2_—*w* ${\;}^{3}F_{3}^{{}^{\circ}}$
10	4967.368	20125.77	*z* ${\;}^{3}G_{4}^{{}^{\circ}}$*—e* ^7^D_3_
30	4967.618	20124.76	$\left\{ \begin{array}{l} \;z{\;}^{7}F_{3}^{{}^{\circ}}—e{\;}^{7}D_{3}\\ \;z{\;}^{7}F_{5}^{{}^{\circ}}—C_{5} \end{array}\right.$
50	4968.006	20123.19	*c* ^3^P_1_*—z* ${\;}^{1}P_{1}^{{}^{\circ}}$
300	4968.900	20119.57	*a* ^5^P_3_*—z* ${\;}^{5}F_{3}^{{}^{\circ}}$
6	4970.181	20114.38	*c* ^3^F_3_*—w* ${\;}^{3}D_{2}^{{}^{\circ}}$
10	4970.798	20111.88	2 ${\;}^{7}P_{2}^{{}^{\circ}}$—D_3_
10	4971.414	20109.39	*b* ^3^P_1_*—y* ${\;}^{5}D_{1}^{{}^{\circ}}$
150	4974.106	20098.51	*b* ^3^H_6_—*z* ${\;}^{3}I_{7}^{{}^{\circ}}$
250	4976.200	20090.05	*z* ${\;}^{5}F_{5}^{{}^{\circ}}$—*f* ^5^F_5_
10*h*	4977.208	20085.98	*z* ${\;}^{5}F_{2}^{{}^{\circ}}$—*e* ^5^D_1_
50	4977.982	20082.86	$\left\{ \begin{array}{l} \;b{\;}^{3}P_{2}—y{\;}^{5}D_{2}^{{}^{\circ}}\\ \;c{\;}^{3}P_{2}—x{\;}^{3}D_{1}^{{}^{\circ}} \end{array}\right.$
300	4980.359	20073.27	*a* ^5^P_1_*—z* ${\;}^{5}F_{1}^{{}^{\circ}}$
6*h*	4983.103	20062.22	*z* ${\;}^{5}F_{2}^{{}^{\circ}}$*—e* ^5^S_2_
150	4983.443	20060.85	*a* ^5^D_2_—*z* ${\;}^{5}D_{1}^{{}^{\circ}}$
30	4984.695	20055.81	*b* ^1^D_2_—*u* ${\;}^{3}D_{2}^{{}^{\circ}}$
160	4987.262	20045.49	*a* ^5^D_1_—*z* ${\;}^{5}D_{1}^{{}^{\circ}}$
2*h*	4991.196	20029.69	$\left\{ \begin{array}{l} \;z{\;}^{7}F_{4}^{{}^{\circ}}—f{\;}^{7}D_{4}\\ \;a{\;}^{3}G_{3}—y{\;}^{5}D_{2}^{{}^{\circ}} \end{array}\right.$
200	4992.741	20023.49	*a* ^5^D_4_—*z* ${\;}^{5}D_{3}^{{}^{\circ}}$
10	4994.898	20014.85	c ^3^P_2_—*z* ${\;}^{1}F_{3}^{{}^{\circ}}$
1	4996.24	20009.47	*d* ^3^F_3_— $22_{3}^{{}^{\circ}}$
10*h*	4998.049	20002.23	*z* ${\;}^{3}F_{4}^{{}^{\circ}}$—*f* ^5^D_4_
1*h*	4999.00	19998.42	$\left\{ \begin{array}{l} \;z{\;}^{7}F_{2}^{{}^{\circ}}—e{\;}^{7}D_{3}\\ \;z{\;}^{7}F_{4}^{{}^{\circ}}—\;C_{5} \end{array}\right.$
6*h*	4999.538	19996.27	*a* ^1^F_3_—*y* ${\;}^{5}S_{2}^{{}^{\circ}}$
125	5003.523	19980.34	*z* ${\;}^{5}D_{3}^{{}^{\circ}}$—B_4_
100	5005.243	19973.48	*b* ^3^H_4_*—z* ${\;}^{3}I_{5}^{{}^{\circ}}$
125	5010.600	19952.13	*z* ${\;}^{5}D_{3}^{{}^{\circ}}$—*e* ^3^P_2_
250	5011.234	19949.60	*a* ^5^P_1_—*z* ${\;}^{5}D_{0}^{{}^{\circ}}$
1	5014.320	19937.32	*b* ^3^F_2_—*z* ${\;}^{7}P_{3}^{{}^{\circ}}$
200	5014.957	19934.79	*a* ^3^F_2_—*z* ${\;}^{5}D_{1}^{{}^{\circ}}$
2	5015.997	19930.66	*a* ^3^F_3_-—*z* ${\;}^{5}F_{4}^{{}^{\circ}}$
8	5016.816	19927.40	*c* ^3^F_3_—*x* ${\;}^{3}F_{4}^{{}^{\circ}}$
150	5018.961	19918.89	*z* ${\;}^{5}F_{3}^{{}^{\circ}}$—*f* ^5^F_3_
80	5020.315	19913.52	*a* ^3^P_2_—*z* ${\;}^{5}G_{3}^{{}^{\circ}}$
300	5026.197	19890.21	*a* ^5^D_3_—*z* ${\;}^{5}D_{2}^{{}^{\circ}}$
200	5028.162	19882.44	*b* ^3^F_3_—*z* ${\;}^{5}G_{3}^{{}^{\circ}}$
4	5031.895	19867.69	*z* ${\;}^{7}F_{1}^{{}^{\circ}}$—F_1_
1	5032.34	19865.93	*z* ${\;}^{7}F_{3}^{{}^{\circ}}$*—e* ^3^D_2_
2	5032.900	19863.72	*z* ${\;}^{5}F_{2}^{{}^{\circ}}$—*e* ^5^P_3_
6	5036.986	19847.61	*b* ^3^G_3_*—t* ${\;}^{3}D_{2}^{{}^{\circ}}$
10	5039.037	19839.53	*c* ^3^F_4_*—w* ${\;}^{3}D_{3}^{{}^{\circ}}$
150	5039.627	19837.21	*z* ${\;}^{5}F_{3}^{{}^{\circ}}$*—e* ^3^G_4_
125	5040.357	19834.33	*c* ^3^P_2_—*x* ${\;}^{3}D_{3}^{{}^{\circ}}$
160	5040.738	19832.83	*a* ^5^D_2_—*z* ${\;}^{5}F_{3}^{{}^{\circ}}$
20	5041.350	19830.43	*z* ${\;}^{5}F_{3}^{{}^{\circ}}$—*e* ^5^D_2_
20	5041.978	19827.96	$\left\{ \begin{array}{l} \;b{\;}^{3}H_{5}—x{\;}^{3}F_{4}^{{}^{\circ}}\\ \;a{\;}^{3}D_{2}—z{\;}^{5}P_{2}^{{}^{\circ}} \end{array}\right.$
100	5045.432	19814.38	*z* ${\;}^{5}G_{6}^{{}^{\circ}}$—*e* ^7^D_5_
200	5047.308	19807.02	*a* ^5^P_1_*—z* ${\;}^{5}F_{2}^{{}^{\circ}}$
100	5052.955	19784.88	$\left\{ \begin{array}{l} \;b{\;}^{3}P_{2}—y{\;}^{5}D_{3}^{{}^{\circ}}\\ \;z{\;}^{3}G_{4}—e{\;}^{3}G_{3} \end{array}\right.$
1000	5057.340	19767.73	*a* ^3^F_4_—*z* ${\;}^{5}D_{4}^{{}^{\circ}}$
10	5057.68	1 9766.40	*z* ${\;}^{7}P_{4}^{{}^{\circ}}$*—e* ^7^D_3_
125	5062.658	19746.96	*b* ^3^P_1_*—y* ${\;}^{5}D_{2}^{{}^{\circ}}$
15	5063.196	19744.87	*d* ^3^F_3_— $19_{4}^{{}^{\circ}}$
10	5066.668	19731.34	*d* ^3^F_3_*—w* ${\;}^{3}F_{2}^{{}^{\circ}}$
60	5071.013	19714.43	$\left\{ \begin{array}{l} \;c{\;}^{3}P_{1}—x{\;}^{3}D_{2}^{{}^{\circ}}\\ \;a{\;}^{1}D_{2}—y{\;}^{3}D_{3}^{{}^{\circ}} \end{array}\right.$
250	5072.993	19706.74	*a* ^3^F_2_—*z* ${\;}^{5}F_{3}^{{}^{\circ}}$
4*h*	5075.641	19696.45	2 ${\;}^{7}F_{4}^{{}^{\circ}}$*—e* ^5^P_3_
100	5076.087	19694.72	*a* ^5^P_3_—*z* ${\;}^{5}D_{2}^{{}^{\circ}}$
300	5076.327	19693.79	*b* ^3^F_3_—*z* ${\;}^{3}F_{4}^{{}^{\circ}}$
125	5079.463	19681.63	*z* ${\;}^{5}D_{3}^{{}^{\circ}}$—*e* ^5^D_3_
2*h*	5081.466	19673.88	*z* ${\;}^{7}P_{3}^{{}^{\circ}}$—*f* ^7^D_2_
1	5082.91	19668.29	*z* ${\;}^{3}F_{4}^{{}^{\circ}}$*—e* ^7^D_3_
2*h*	5083.992	19664.10	*c* ^3^F_2_*—x* ${\;}^{3}D_{2}^{{}^{\circ}}$
1*h*	5084.58	19661.83	*z* ${\;}^{7}F_{3}^{{}^{\circ}}$*—e* ^5^G_3_
150*H*	5086.776	19653.34	*z* ${\;}^{5}F_{3}^{{}^{\circ}}$—*f* ^5^F_4_
100	5090.086	19640.56	*z* ${\;}^{7}F_{4}^{{}^{\circ}}$—*e* ^7^D_4_
600	5093.850	19626.05	*a* ^5^D_0_—*z* ${\;}^{5}D_{1}^{{}^{\circ}}$
20	5096.220	19616.92	*c* ^3^F_4_—*x* ${\;}^{3}F_{3}^{{}^{\circ}}$
10*h*	5100.850	19599.11	2 ${\;}^{5}F_{3}^{{}^{\circ}}$*—e* ^3^D_3_
200	5101.395	19597.02	*b* ^3^F_2_—*z* ${\;}^{3}D_{3}^{{}^{\circ}}$
125	5101.724	19595.76	*b* ^3^F_3_—*z* ${\;}^{7}P_{4}^{{}^{\circ}}$
100	51 06.570	19577.16	*b* ^3^H_6_*—x* ${\;}^{3}G_{5}^{{}^{\circ}}$
200	5107.071	19575.24	*a* ^3^G_4_—*z* ${\;}^{3}F_{3}^{{}^{\circ}}$
2*h*	5111.249	19559.24	*b* ^3^G_4_—*x* ${\;}^{1}F_{3}^{{}^{\circ}}$
40	5114.590	19546.46	*b* ^1^G_4_—*x* ${\;}^{3}G_{4}^{{}^{\circ}}$
60	5114.996	19544.91	*b* ^1^D_2_*—y* ${\;}^{1}F_{3}^{{}^{\circ}}$
30	5119.866	19526.32	*b* ^3^P_2_—*z* ${\;}^{3}F_{2}^{{}^{\circ}}$
125	5123.761	19511.48	*b* ^3^F_2_—*z* ${\;}^{5}G_{2}^{{}^{\circ}}$
6	5126.775	19500.01	*a* ^1^P_1_—*y* ${\;}^{3}P_{2}^{{}^{\circ}}$
150	5127.265	19498.14	*a* ^5^P_1_—*z* ${\;}^{5}D_{1}^{{}^{\circ}}$
30	5129.279	19490.49	a ^3^F_4_—*z* ${\;}^{7}D_{3}^{{}^{\circ}}$
20	5133.895	19472.96	*a* ^3^G_3_—*z* ${\;}^{3}F_{2}^{{}^{\circ}}$
150	5134.111	19472.14	*z* ${\;}^{5}F_{4}^{{}^{\circ}}$—B_4_
1000	5136.558	19462.87	*a* ^5^P^2^—*z* ${\;}^{5}D_{3}^{{}^{\circ}}$
40	5142.325	19441.04	*b* ^1^D_2_—*w* ${\;}^{3}D_{1}^{{}^{\circ}}$
400	5142.772	19439.35	*a* ^5^D_3_—*z* ${\;}^{5}F_{4}^{{}^{\circ}}$
400	5147.246	19422.45	*a* ^3^F_3_—*z* ${\;}^{5}D_{3}^{{}^{\circ}}$
10	5149.753	19413.00	*c* ^3^F_2_—*z* ${\;}^{1}D_{2}^{{}^{\circ}}$
300	5151.070	19408.04	*a* ^5^D_2_—*z* ${\;}^{5}D_{2}^{{}^{\circ}}$
125	5153.214	19399.96	*a* ^3^P_1_—*z* ${\;}^{5}S_{2}^{{}^{\circ}}$
60	5154.604	19394.73	$\left\{ \begin{array}{l} \;a{\;}^{3}P_{2}—z{\;}^{7}F_{2}^{{}^{\circ}}\\ \;a{\;}^{1}H_{5}—y{\;}^{3}G_{5}^{{}^{\circ}} \end{array}\right.$
400	5155.140	19392.71	*a* ^5^D_1_*—z* ${\;}^{5}D_{2}^{{}^{\circ}}$
150	5160.008	19374.42	*b* ^3^F_4_—*z* ${\;}^{3}G_{5}^{{}^{\circ}}$
50	5168.062	19344.23	*z* ${\;}^{3}G_{4}^{{}^{\circ}}$*—e* ^7^D_4_
20	5168.636	19342.08	*b* ^3^H_4_—*x* ${\;}^{3}F_{4}^{{}^{\circ}}$
20	5170.078	19336.68	*c* ^3^F_3_*—z* ${\;}^{1}D_{2}^{{}^{\circ}}$
1000	5171.026	19333.14	*a* ^5^D_4_*—z* ${\;}^{5}F_{5}^{{}^{\circ}}$
50	5177.993	19307.13	*d* ^3^P_2_*—x* ${\;}^{3}P_{2}^{{}^{\circ}}$
30	5184.753	19281.95	*a* ^3^F*_2_—z* ${\;}^{5}D_{2}^{{}^{\circ}}$
6	5188.410	19268.36	*a* ^3^P_2_—*z* ${\;}^{7}F_{3}^{{}^{\circ}}$
20	5189.699	19263.58	*b* ^3^P_0_*—y* ${\;}^{5}D_{1}^{{}^{\circ}}$
4	5193.026	19251.23	*a* ^3^D_1_—*y* ${\;}^{5}F_{2}^{{}^{\circ}}$
400	5195.017	19243.86	*a* ^5^P_3_—*z* ${\;}^{5}F_{4}^{{}^{\circ}}$
50	5197.058	19236.30	*b* ^3^F_3_—*z* ${\;}^{3}G_{4}^{{}^{\circ}}$
150	5199.873	19225.88	z ${\;}^{7}F_{6}^{{}^{\circ}}$—*e* ^7^D_5_
100*h*	5202.127	19217.55	*a* ^1^H_5_—*z* ${\;}^{3}H_{4}^{{}^{\circ}}$
4	5202.897	19214.71	*z* ${\;}^{7}F_{4}^{{}^{\circ}}$—*f* ^5^F_3_
2*h*	5205.330	19205.73	*z* ${\;}^{3}G_{3}^{{}^{\circ}}$—*f* ^7^D_2_
100	5209.502	19190.35	*b* ^3^P_1_*—z* ${\;}^{3}F_{2}^{{}^{\circ}}$
100	5214.079	19173.51	*z* ${\;}^{5}F_{4}^{{}^{\circ}}$—*e* ^5^D_3_
4	5219.072	19155.16	*z* ${\;}^{7}P_{3}^{{}^{\circ}}$*—e* ^7^D_2_
300	5223.550	19138.74	*a* ^3^G_5_—*z* ${\;}^{5}G_{4}^{{}^{\circ}}$
50	5225.086	19133.11	*z* ${\;}^{7}F_{4}^{{}^{\circ}}$—*e* ^3^G_4_
2*h*	5226.362	19128.44	*z* ${\;}^{3}D_{3}^{{}^{\circ}}$—*e* ^5^P_2_
40	5227.278	19125.09	*c* ^3^F_4_*—x* ${\;}^{3}D_{3}^{{}^{\circ}}$
10	5236.951	19089.77	*b* ^3^F_2_—*z* ${\;}^{5}G_{3}^{{}^{\circ}}$
200	5242.386	19069.98	$\left\{ \begin{array}{l} \;a{\;}^{3}P_{2}—z{\;}^{5}F_{1}^{{}^{\circ}}\\ \;z{\;}^{5}F_{4}^{{}^{\circ}}—e{\;}^{3}G_{5} \end{array}\right.$
150	5242.951	19067.92	*z* ${\;}^{3}G_{3}^{{}^{\circ}}$—*e* ^5^G_4_
2*h*	5244.234	19063.26	*c* ^3^F_3_*—w* ${\;}^{3}D_{3}^{{}^{\circ}}$
50	5245.453	19058.83	*b* ^3^H_5_—*z* ${\;}^{3}I_{6}^{{}^{\circ}}$
2*h*	5246.840	19053.79	*z* ${\;}^{7}F_{5}^{{}^{\circ}}$—*e* ^5^G_5_
10	5250.458	19040.66	*z* ${\;}^{7}P_{4}^{{}^{\circ}}$—*e* ^5^P_3_
300	5251.664	19036.29	*a* ^3^G_4_—*z* ${\;}^{3}G_{3}^{{}^{\circ}}$
200	5257.081	19016.67	*z* ${\;}^{5}G_{3}^{{}^{\circ}}$*—e* ^5^G_3_
100	5263.960	18991.82	*z* ${\;}^{3}G_{5}^{{}^{\circ}}$—B_4_
30	5265.922	18984.74	*z* ${\;}^{7}P_{4}^{{}^{\circ}}$—*e* ^7^D_4_
100	5266.469	18982.77	*c* ^3^P_2_—*y* ${\;}^{3}P_{1}^{{}^{\circ}}$
100	5266.815	18981.52	*a* ^1^H_5_—*z* ${\;}^{1}G_{4}^{{}^{\circ}}$
2	5269.139	18973.15	*c* ^3^F_4_*—z* ${\;}^{1}H_{5}^{{}^{\circ}}$
4	5271.174	18965.83	*z* ${\;}^{7}P_{3}^{{}^{\circ}}$—*f* ^5^D_4_
1	5271.45	18964.84	*b* ^3^G_4_—*x* ${\;}^{5}F_{4}^{{}^{\circ}}$
1	5273.46	18957.61	z ${\;}^{5}F_{4}^{{}^{\circ}}$*—e* ^5^D_4_
10	5274.042	18955.51	*z* ${\;}^{5}G_{5}^{{}^{\circ}}$*—e* ^7^D_4_
60	5275.756	18949.36	*z* ${\;}^{7}F_{4}^{{}^{\circ}}$—*f* ^5^F_4_
60	5278.375	18939.95	*b* ^3^F3—*z* ${\;}^{7}F_{4}^{{}^{\circ}}$
20*h*	5280.074	18933.86	*c* ^3^P_0_*—y* ${\;}^{3}S_{1}^{{}^{\circ}}$
150	5280.812	18931.21	a ^5^D_3_—*z* ${\;}^{5}D_{3}^{{}^{\circ}}$
300	5284, 089	18919.47	*a* ^3^F_4_—*z* ${\;}^{7}D_{4}^{{}^{\circ}}$
50	5284.418	18918.30	*z* ${\;}^{7}F_{5}^{{}^{\circ}}$*—e* ^7^D_5_
150	5291.169	18894 16	*b* ^3^F_4_—*z* ${\;}^{5}F_{4}^{{}^{\circ}}$
2	5291.886	18891.60	*z* ${\;}^{5}F_{4}^{{}^{\circ}}$—*f* ^5^F_5_
2	5293.226	18886.82	*z* ${\;}^{3}F_{4}^{{}^{\circ}}$—*e* ^7^D_4_
200	5304.852	18845.43	*a* ^5^P_1_—*z* ${\;}^{5}D_{2}^{{}^{\circ}}$
50	5305.850	18841.88	*a* ^1^H_5_—*z* ${\;}^{3}H_{6}^{{}^{\circ}}$
50	5306.453	18839.74	$\left\{ \begin{array}{l} \;c{\;}^{3}P_{2}—y{\;}^{5}P_{1}^{{}^{\circ}}\\ \;c{\;}^{3}F_{2}—x{\;}^{3}F_{2}^{{}^{\circ}} \end{array}\right.$
60	5307.284	18836.79	*z* ${\;}^{3}G_{4}^{{}^{\circ}}$—*e* ^3^G_4_
1000	5309.259	18829.78	*a* ^5^D_4_—*z* ${\;}^{5}D_{4}^{{}^{\circ}}$
200	5315.339	18808.24	*c* ^3^P_2_—*y* ${\;}^{3}P_{2}^{{}^{\circ}}$
4	5316.589	18803.82	*a* ^3^P_2_—*z* ${\;}^{5}F_{2}^{{}^{\circ}}$
1	5317.92	18799.12	2 ${\;}^{5}G_{2}^{{}^{\circ}}$—*e* ^3^D_2_
6	5318.749	18796.18	*c* ^3^F_4_— $1_{4}^{{}^{\circ}}$
1	5325.37	18772.82	*b* ^3^F_3_—*z* ${\;}^{5}F_{2}^{{}^{\circ}}$
10*H*	5327.065	18766.84	*a* ^1^P_1_—*y* ${\;}^{5}P_{2}^{{}^{\circ}}$
50	5528.023	18763.47	c ^3^F_3_—*x* ${\;}^{3}F_{2}^{{}^{\circ}}$
2	5328.822	18760.65	*a* ^1^D_2_—*y* ${\;}^{5}F_{3}^{{}^{\circ}}$
150	5332.932	18746.20	*b* ^3^P_2_—*z* ${\;}^{3}F_{3}^{{}^{\circ}}$
250	5335.924	18735.68	*a* ^5^P_3_*—z* ${\;}^{5}D_{3}^{{}^{\circ}}$
4	5340.175	18720.77	*c* ^3^P_0_*—w* ${\;}^{3}D_{1}^{{}^{\circ}}$
4	5341.712	18715.39	*z* ${\;}^{7}P_{2}^{{}^{\circ}}$—*f* ^7^D_2_
4	5342.230	18713.57	*z* ${\;}^{3}D_{3}^{{}^{\circ}}$—*e* ^3^D_2_
2	5343.308	18709.80	*a* ^3^H_4_*—y* ${\;}^{5}F_{3}^{{}^{\circ}}$
50	5346.791	18697.61	*b* ^3^P_2_—*z* ${\;}^{7}P_{2}^{{}^{\circ}}$
80	5348.132	18692.92	*a* ^3^G_3_*—z* ${\;}^{3}F_{3}^{{}^{\circ}}$
1	5348.58	18691.35	*a*^3^D_3_—*z* ${\;}^{5}P_{2}^{{}^{\circ}}$
6	5353.693	18673.50	*c* ^3^F_2_—*x* ${\;}^{3}D_{1}^{{}^{\circ}}$
20	5354.952	18669.11	*a* ^3^F_4_*—z* ${\;}^{7}D_{5}^{{}^{\circ}}$
100	5359.644	18652.77	*z* ${\;}^{3}G_{4}^{{}^{\circ}}$—*f* ^5^F_4_
50	5362.094	18644.25	*a* ^3^G_3_*—z* ${\;}^{7}P_{2}^{{}^{\circ}}$
125	5365.604	18632.05	*z* ${\;}^{7}P_{3}^{{}^{\circ}}$—*e* ^7^D_3_
20	5373.281	18605.43	*c* ^3^F_2_—*z* ${\;}^{1}F_{3}^{{}^{\circ}}$
60	5377.068	18592.33	*c* ^3^F_4_—*y* ${\;}^{3}G_{3}^{{}^{\circ}}$
300	5377.840	18589.66	*z* ${\;}^{3}G_{5}^{{}^{\circ}}$—*e* ^3^G_5_
3*h*	5384.12	18567.97	*a* ^3^G_4_—*z* ${\;}^{7}P_{3}^{{}^{\circ}}$
200	5385.898	18561.84	*b* ^3^P_2_— *z* ${\;}^{3}D_{2}^{{}^{\circ}}$
50	5388.629	18552.44	*a* ^5^D_4_—*z* ${\;}^{7}D_{3}^{{}^{\circ}}$
2*h*	5391.59	18542.25	*d* ^3^P_1_— $11_{0}^{{}^{\circ}}$
40	5395.385	18529.21	$\left\{ \begin{array}{l} \;a{\;}^{3}G_{4}—z{\;}^{5}G_{4}^{{}^{\circ}}\\ \;c{\;}^{3}F_{3}—z{\;}^{1}F_{3}^{{}^{\circ}} \end{array}\right.$
400	5401.402	18508.57	*a* ^3^G_3_—*z* ${\;}^{3}D_{2}^{{}^{\circ}}$
4	5405.249	18495.39	*z* ${\;}^{3}G_{4}^{{}^{\circ}}$—*e* ^7^D_5_
4	5405.384	18494.93	*a* ^3^P_2_—*z* ${\;}^{5}D_{1}^{{}^{\circ}}$
20	5410.377	18477.86	*b* ^3^H_4_*—w* ${\;}^{3}D_{3}^{{}^{\circ}}$
1	5413.75	18466.35	*d* ^3^F_4_*—w* ${\;}^{3}F_{4}^{{}^{\circ}}$
1	5415.38	18460.79	*z* ${\;}^{3}F_{4}^{{}^{\circ}}$—*f* ^5^F_3_
20	5417.344	18454.10	*b* ^3^H_6_—*z* ${\;}^{1}H_{5}^{{}^{\circ}}$
250	5418.864	18448.92	*a* ^5^D_2_—*z* ${\;}^{5}D_{3}^{{}^{\circ}}$
5*H*	5419.13	18448.02	*z* ${\;}^{5}G_{5}^{{}^{\circ}}$*—e* ^3^G_4_
1	5420.15	18444.55	*b* ^3^F_2_—*z* ${\;}^{7}F_{3}^{{}^{\circ}}$
4	5424.722	18429.00	*a* ^5^P_2_—*z* ${\;}^{7}D_{2}^{{}^{\circ}}$
400	5427.610	18419.20	*z* ${\;}^{5}G_{6}^{{}^{\circ}}$—*e* ^5^G_6_
1*h*	5429.96	18411.22	$\left\{ \begin{array}{l} \;z{\;}^{3}D_{2}^{{}^{\circ}}—e{\;}^{7}D_{1}\\ \;z{\;}^{3}G_{5}^{{}^{\circ}}—f{\;}^{5}F_{5} \end{array}\right.$
1	5436.65	18388.57	*a* ^3^F_3_—*z* ${\;}^{7}D_{2}^{{}^{\circ}}$
100	5439.416	18379.22	*z* ${\;}^{3}F_{4}^{{}^{\circ}}$—*e* ^3^G_4_
60	5440.206	18376.55	*a* ^3^D_2_*—y* ${\;}^{5}D_{3}^{{}^{\circ}}$
4	5444.621	18361.65	*b* ^3^P_1_—*z* ${\;}^{7}P_{2}^{{}^{\circ}}$
6	5448.471	18348.67	*c* ^3^F_3_*—x* ${\;}^{3}D_{3}^{{}^{\circ}}$
2	5449.51	18345.18	*a* ^1^P_1_—*z* ${\;}^{3}S_{1}^{{}^{\circ}}$
100	5452.728	18334.35	$\left\{ \begin{array}{l} \;z{\;}^{5}G_{6}^{{}^{\circ}}—f{\;}^{5}F_{5}\\ \;a{\;}^{3}D_{1}—z{\;}^{5}P_{1}^{{}^{\circ}} \end{array}\right.$
6	5453.388	18332.13	*z* ${\;}^{3}D_{2}^{{}^{\circ}}$—*e* ^7^D_2_
4	5453.974	18330.16	*z* ${\;}^{5}G_{4}^{{}^{\circ}}$—*e* ^3^G_3_
4	5454.26	18329.20	*a* ^3^P_1_—*z* ${\;}^{7}F_{0}^{{}^{\circ}}$
250	5454.809	18327.35	*z* ${\;}^{5}S_{2}^{{}^{\circ}}$—*e* ^5^D_1_
200	5456.124	18322.94	*a* ^3^F_2_—*z* ${\;}^{5}D_{3}^{{}^{\circ}}$
20	5461.902	18303.55	*z* ${\;}^{5}S_{2}^{{}^{\circ}}$—*e* ^5^S_2_
15	5463.161	18299.34	*a* ^3^P_1_—*z* ${\;}^{7}F_{1}^{{}^{\circ}}$
20	5464.913	18293.47	*z* ${\;}^{7}P_{4}^{{}^{\circ}}$—*f* ^5^F_4_
4	5470.266	18275.57	*z* ${\;}^{7}P_{2}^{{}^{\circ}}$—*e* ^7^D_1_
40*h*	5471.526	18271.36	*z* ${\;}^{7}P_{4}^{{}^{\circ}}$—e ^5^G_5_
80	5472.852	18266.93	*a* ^3^P_2_—*z* ${\;}^{5}F_{3}^{{}^{\circ}}$
20*h*	5473.674	18264.19	*z* ${\;}^{5}G_{5}^{{}^{\circ}}$—*f* ^5^F_4_
150	5475.187	18259.14	*z* ${\;}^{5}G_{4}^{{}^{\circ}}$*—e* ^5^G_4_
60	5476.360	18255.23	*b* ^3^H_4_—*x* ${\;}^{3}F_{3}^{{}^{\circ}}$
200	5480.305	18242.09	$\left\{ \begin{array}{l} \;z{\;}^{5}G_{5}^{{}^{\circ}}—e{\;}^{5}G_{5}\\ \;c{\;}^{3}P_{0}—z{\;}^{1}P_{1}^{{}^{\circ}} \end{array}\right.$
4	5481.130	18239.34	$\left\{ \begin{array}{l} \;z{\;}^{7}P_{4}^{{}^{\circ}}—e{\;}^{3}D_{3}\\ \;d{\;}^{3}F_{4}—x{\;}^{1}G_{4}^{{}^{\circ}} \end{array}\right.$
10	5482.180	18235.85	*b* ^3^F_3_—*z* ${\;}^{5}F_{3}^{{}^{\circ}}$
10*h*	5483.251	18232.29	*a* ^3^P_1_—*z* ${\;}^{7}F_{2}^{{}^{\circ}}$
200	5484.325	18228.72	*a* ^3^F_3_—*z* ${\;}^{5}D_{4}^{{}^{\circ}}$
100	5484.646	18227.65	*a* ^3^G_4_—*z* ${\;}^{3}D_{3}^{{}^{\circ}}$
2*h*	5485.11	18226.11	*b* ^3^P_1_—*z* ${\;}^{3}D_{2}^{{}^{\circ}}$
30	5490.578	18207.96	*z* ${\;}^{5}G_{4}^{{}^{\circ}}$—*e* ^5^G_3_
2	5490.824	18207.15	*b* ^3^P_2_—*z* ${\;}^{3}G_{3}^{{}^{\circ}}$
100	5494.36	18195.43	*z* ${\;}^{3}F_{4}^{{}^{\circ}}$—*f* ^5^F_4_
160	5496.696	18187.69	*a* ^1^H_5_—*y* ${\;}^{3}F_{4}^{{}^{\circ}}$
4	5498.999	18180.08	*d* ^3^P_2_—*y* ${\;}^{3}S_{1}^{{}^{\circ}}$
150	5501.024	18173.39	*z* ${\;}^{3}F_{4}^{{}^{\circ}}$—*e* ^5^G_5_
1	5502.20	18169.50	*a* ^3^D_1_*—z* ${\;}^{5}P_{2}^{{}^{\circ}}$
50	5506.940	18153.86	a ^3^G_3_—*z* ${\;}^{3}G_{3}^{{}^{\circ}}$
500	5510.723	18141.40	*a* ^3^G_5_—*z* ${\;}^{3}F_{4}^{{}^{\circ}}$
80	5512.378	18135.95	*z* ${\;}^{7}P_{4}^{{}^{\circ}}$—*e* ^7^D_5_
60	5517.846	18117.98	*a* ^3^D_2_—*z* ${\;}^{3}F_{2}^{{}^{\circ}}$
60	5521.780	18105.07	*z* ${\;}^{5}S_{2}^{{}^{\circ}}$*—e* ^5^P_3_
4	5524.148	18097.31	*b* ^3^H_5_*—z* ${\;}^{1}H_{5}^{{}^{\circ}}$
6	5526.346	18090.11	$\left\{ \begin{array}{l} \;c{\;}^{3}F_{2}—y{\;}^{3}F_{2}^{{}^{\circ}}\\ \;b{\;}^{1}G_{4}—w{\;}^{3}D_{3}^{{}^{\circ}} \end{array}\right.$
60	5531.001	18074.89	*c* ^3^P_2_—*y* ${\;}^{5}P_{2}^{{}^{\circ}}$
150	5540.659	18043.38	*a* ^3^G_5_—*z* ${\;}^{7}P_{4}^{{}^{\circ}}$
4	5542.35	18037.88	*z* ${\;}^{3}F_{4}^{{}^{\circ}}$*—e* ^7^D_5_
30	5549.749	18013.83	*c* ^3^F_3_*—y* ${\;}^{3}F_{2}^{{}^{\circ}}$
1	5553.78	18000.76	*a* ^1^D_2_—*z* ${\;}^{5}P_{1}^{{}^{\circ}}$
100	5556.524	17991.87	*a* ^5^P_2_—*z* ${\;}^{7}D_{3}^{{}^{\circ}}$
400	5559.744	17981.45	*a* ^5^D_4_—*z* ${\;}^{7}D_{4}^{{}^{\circ}}$
150	5569.036	17951.44	*a* ^3^F_3_—*z* ${\;}^{7}D_{3}^{{}^{\circ}}$
15	5570.71	17946.05	*b* ^3^G_5_*—y* ${\;}^{3}H_{6}^{{}^{\circ}}$
2	5572.30	17940.93	*a* ^3^P_0_—*z* ${\;}^{5}F_{1}^{{}^{\circ}}$
10	5578.688	17920.39	*b* ^3^H_5_— $1_{4}^{{}^{\circ}}$
60	5579.435	17917.99	*y* ${\;}^{5}D_{4}^{{}^{\circ}}$—*f* ^5^D_3_
4*h*	5583.054	17906.37	*z* ${\;}^{7}P_{3}^{{}^{\circ}}$—*e* ^5^P_3_
4	5587.436	17892.33	$\left\{ \begin{array}{l} \;c{\;}^{3}F_{2}—y{\;}^{3}G_{3}^{{}^{\circ}}\\ \;z{\;}^{7}F_{4}^{{}^{\circ}}—B_{4} \end{array}\right.$
20	5595.208	17867.48	*b* ^1^G_4_—*x* ${\;}^{3}F_{3}^{{}^{\circ}}$
50	5600.530	17850.50	*z* ${\;}^{7}P_{3}^{{}^{\circ}}$—*e* ^7^D_4_
125	5603.144	17842.17	*a* ^3^P_2_—*z* ${\;}^{5}D_{2}^{{}^{\circ}}$
150	5606.748	17830.70	*z* ${\;}^{7}F_{6}^{{}^{\circ}}$*—e* ^5^G_6_
4*h*	5607.744	17827.53	*b* ^3^G_3_—*y* ${\;}^{1}D_{2}^{{}^{\circ}}$
100	5609.147	17823.08	*z* ${\;}^{3}G_{3}^{{}^{\circ}}$—*e* ^3^G_3_
6	5611.398	17815.93	*c* ^3^F_3_—*y* ${\;}^{3}G_{3}^{{}^{\circ}}$
4	5619.374	17790.64	*c* ^3^F_4_—*y* ${\;}^{3}F_{3}^{{}^{\circ}}$
2*h*	5625.07	17772.62	*c* ^3^P_2_—*y* ${\;}^{5}P_{3}^{{}^{\circ}}$
60*h*	5627.506	17764.93	*z* ${\;}^{3}D_{3}^{{}^{\circ}}$—*f* ^5^F_3_
60	5629.798	17757.70	*b* ^3^H_5_—*y* ${\;}^{3}G_{4}^{{}^{\circ}}$
30*h*	5631.584	17752.07	*z* ${\;}^{3}G_{3}^{{}^{\circ}}$—*e* ^5^G_4_
20	5633.560	17745.84	*z* ${\;}^{7}F_{6}^{{}^{\circ}}$*—f* ^5^F_5_
1000	5636.233	17737.42	*a* ^5^D_3_—*z* ${\;}^{5}D_{4}^{{}^{\circ}}$
6*h*	5638.716	17729.61	*b* ^1^D_2_—*x* ${\;}^{3}F_{2}^{{}^{\circ}}$
100	5641.671	17720.33	*a* ^3^G_4_—*z* ${\;}^{5}G_{3}^{{}^{\circ}}$
1	5645.74	17707.56	*a* ^5^D_1_—*z* ${\;}^{7}D_{1}^{{}^{\circ}}$
60	5647.577	17701.80	*a* ^5^P_3_—*z* ${\;}^{7}D_{2}^{{}^{\circ}}$
80 *h*	5647.888	17700.82	*z* ${\;}^{3}G_{3}^{{}^{\circ}}$—*e* ^5^G_3_
125	5649.569	17695.56	*b* ^3^F_4_—*z* ${\;}^{5}F_{5}^{{}^{\circ}}$
10	5650.818	17691.64	*a* ^1^G**_4_**—*z* ${\;}^{3}F_{3}^{{}^{\circ}}$
1	5652.80	17685.44	$\left\{ \;\begin{array}{l} a{\;}^{3}G_{3}—z{\;}^{7}P_{3}^{{}^{\circ}}\\ d{\;}^{3}F_{3}—x{\;}^{1}F_{3}^{{}^{\circ}} \end{array}\right.$
80	5653.314	17683.83	*a* ^3^G_5_—*z* ${\;}^{3}G_{4}^{{}^{\circ}}$
10	5656.640	17673.44	*z* ${\;}^{7}P_{2}^{{}^{\circ}}$—*e* ^7^D_3_
6	5663.071	17653.37	*c* ^3^P_2_—*z* ${\;}^{3}S_{1}^{{}^{\circ}}$
100	5665.206	17646.71	*a* ^3^G_3_—*z* ${\;}^{5}G_{4}^{{}^{\circ}}$
6	5670.512	17630.20	*c* ^3^F_4_—*z* ${\;}^{3}H_{4}^{{}^{\circ}}$
2	5671.93	17625.79	*d* ^3^F_2_— $4_{3}^{{}^{\circ}}$
10*h*	5674.844	17616.74	*z* ${\;}^{7}F_{5}^{{}^{\circ}}$—*e* ^3^G_5_
30	5676.556	17611.43	*b* ^3^H_4_—*z* ${\;}^{1}H_{5}^{{}^{\circ}}$
100	5679.623	17601.92	*c* ^3^P_1_—*y* ${\;}^{3}P_{0}^{{}^{\circ}}$
4	5681.524	17596.03	*z* ${\;}^{3}G_{4}^{{}^{\circ}}$—B_4_
1	5682.26	17593.75	*z* ${\;}^{7}F_{4}^{{}^{\circ}}$—*e* ^5^D_3_
50	5688.830	17573.43	*c* ^3^F_2_—*y* ${\;}^{3}P_{1}^{{}^{\circ}}$
6	5692.114	17563.29	*b* ^1^D_2_—*x* ${\;}^{3}D_{1}^{{}^{\circ}}$
100	5693.032	17560.46	*a* ^1^P_1_—*x* ${\;}^{5}D_{0}^{{}^{\circ}}$
50	5694.462	17556.05	*b* ^1^G_4_—*z* ${\;}^{1}F_{3}^{{}^{\circ}}$
80	5696.35	17550.23	*z* ${\;}^{3}D_{2}^{{}^{\circ}}$—*e* ^3^D_2_
600	5699.049	17541.92	*a* ^5^P_3_—*z* ${\;}^{5}D_{4}^{{}^{\circ}}$
100	5699.58	17540.29	*b* ^3^P_2_—*z* ${\;}^{5}S_{2}^{{}^{\circ}}$
150	5702.368	17531.71	*a* ^3^G_4_—*z* ${\;}^{3}F_{4}^{{}^{\circ}}$
2	5705.15	17523.16	*z* ${\;}^{7}F_{5}^{{}^{\circ}}$—*e* ^5^G_6_
1*h*	5711.27	17504.39	*z* ${\;}^{7}F_{5}^{{}^{\circ}}$—*e* ^5^D_4_
150	5712.86	17499.51	*z* ${\;}^{3}D_{3}^{{}^{\circ}}$—*f* ^5^F_4_
40	5714.233	17495.31	*b* ^1^D_2_—*z* ${\;}^{1}F_{3}^{{}^{\circ}}$
4*h*	5715.89	17490.24	*z* ${\;}^{7}F_{4}^{{}^{\circ}}$—*e* ^3^G_5_
2	5718.96	17480.85	*c* ^3^P_1_—*y* ${\;}^{5}P_{1}^{{}^{\circ}}$
40	5723.058	17468.33	*z* ${\;}^{3}D_{2}^{{}^{\circ}}$—*e* ^3^G_3_
125	5724.818	17462.96	*a* ^3^G_4_—*z* ${\;}^{5}G_{5}^{{}^{\circ}}$
100	5725.740	17460.15	*a* ^5^D_3_—*z* ${\;}^{7}D_{3}^{{}^{\circ}}$
40*h*	5730.589	17445.37	*z* ${\;}^{3}D_{3}^{{}^{\circ}}$—*e* ^3^D_3_
1	5731.295	17443.23	*b* ^3^F_2_—*z* ${\;}^{5}F_{3}^{{}^{\circ}}$
4*h*	5732.90	17438.34	$\left\{ \;\begin{array}{l} z{\;}^{7}F_{5}^{{}^{\circ}}—f{\;}^{5}F_{5}\\ z{\;}^{3}G_{3}^{{}^{\circ}}—e{\;}^{5}P_{3} \end{array}\right.$
10	5734.445	17433.64	*a* ^3^G_4_—*z* ${\;}^{7}P_{4}^{{}^{\circ}}$
1*H*	5735.54	17430.32	*c* ^3^F_2_— *y* ${\;}^{5}P_{1}^{{}^{\circ}}$
4	5736.980	17425.94	*a* ^1^P_1_—*x* ${\;}^{5}D_{2}^{{}^{\circ}}$
1	5737.43	17424.57	*z* ${\;}^{7}P_{3}^{{}^{\circ}}$—*f* ^5^F_3_
40	5740.555	17415.09	*a* ^5^D_2_—*z* ${\;}^{7}D_{2}^{{}^{\circ}}$
100	5745.65	17399.65	*a* ^5^D_1_—*z* ${\;}^{7}D_{2}^{{}^{\circ}}$
150	5746.002	17398.58	*b* ^3^P_2_—*z* ${\;}^{3}D_{3}^{{}^{\circ}}$
150	5747.481	17394.10	*c* ^3^F_4_—*z* ${\;}^{1}G_{4}^{{}^{\circ}}$
125	5752.022	17380.37	*a* ^3^F_3_—*z* ${\;}^{7}D_{4}^{{}^{\circ}}$
20	5753.621	17375.54	$\left\{ \;\begin{array}{l} b{\;}^{1}G_{4}—x{\;}^{3}D_{3}^{{}^{\circ}}\\ b{\;}^{3}G_{5}—z{\;}^{3}I_{5}^{{}^{\circ}} \end{array}\right.$
16*h*	5756.188	17367.79	*z* ${\;}^{3}F_{2}^{{}^{\circ}}$—*e* ^7^D_2_
100	5756.832	17365.85	$\left\{ \;\begin{array}{l} a{\;}^{3}P_{0}—z{\;}^{5}D_{1}^{{}^{\circ}}\\ z{\;}^{3}F_{3}^{{}^{\circ}}—e{\;}^{3}D_{2} \end{array}\right.$
60	5758.722	17360.15	$\left\{ \;\begin{array}{l} b{\;}^{3}F_{3}—z{\;}^{5}F_{4}^{{}^{\circ}}\\ b{\;}^{3}D_{3}—s{\;}^{5}D_{3}^{{}^{\circ}} \end{array}\right.$
1	5760.23	17355.61	*b* ^3^G_4_—*y* ${\;}^{1}F_{3}^{{}^{\circ}}$
30	5763.659	17345.28	*a* ^3^G_3_—*z* ${\;}^{3}D_{3}^{{}^{\circ}}$
4	5766.119	17337.88	*a* ^3^D_2_—*z* ${\;}^{3}F_{3}^{{}^{\circ}}$
2	5766.589	17336.47	*b* ^3^G_5_—*x* ${\;}^{3}G_{4}^{{}^{\circ}}$
6	5766.905	17335.52	*b* ^3^G_4_—*z* ${\;}^{3}I_{5}^{{}^{\circ}}$
100	5767.923	17332.46	*a* ^3^P_1_—*z* ${\;}^{5}D_{1}^{{}^{\circ}}$
30	5771.215	17322.57	*c* ^3^F_3_—*y* ${\;}^{3}P_{2}^{{}^{\circ}}$
100	5774.384	17313.06	*b* ^3^P_2_—*z* ${\;}^{5}G_{2}^{{}^{\circ}}$
4*h*	5774.847	17311.68	*z* ${\;}^{7}F_{4}^{{}^{\circ}}$—*f* ^5^F_5_
6*h*	5776.866	17305.63	*z* ${\;}^{3}D_{2}^{{}^{\circ}}$—*e* ^5^D_1_
6	5779.961	17296.36	$\left\{ \;\begin{array}{l} b{\;}^{3}G_{4}—x{\;}^{3}G_{4}^{{}^{\circ}}\\ z{\;}^{7}F_{3}^{{}^{\circ}}—e{\;}^{5}G_{3} \end{array}\right.$
60	5782.365	17289.17	*a* ^3^F_2_—*z* ${\;}^{7}D_{2}^{{}^{\circ}}$
80	5782.572	17288.55	*b* ^3^H_6_—*y* ${\;}^{5}G_{5}^{{}^{\circ}}$
20*h*	5784.091	17284.01	*z* ${\;}^{3}F_{3}^{{}^{\circ}}$—*e* ^3^G_3_
1	5784.78	17281.95	*z* ${\;}^{3}D_{2}^{{}^{\circ}}$—*e* ^5^S_2_
50	5790.588	17264.62	*a* ^5^P_3_—*z* ${\;}^{7}D_{3}^{{}^{\circ}}$
50	5792.226	17259.73	*a* ^3^G_3_—*z* ${\;}^{5}G_{2}^{{}^{\circ}}$
6	5798.845	17240.03	*a* ^3^D_3_—*y* ${\;}^{5}D_{3}^{{}^{\circ}}$
10*h*	5800.011	17236.57	*z* ${\;}^{7}P_{4}^{{}^{\circ}}$—B_4_
10	5802.016	17230.61	*b* ^3^H_4_—*y* ${\;}^{3}G_{3}^{{}^{\circ}}$
150	5804.382	17223.59	*b* ^1^G_4_—*z* ${\;}^{1}H_{5}^{{}^{\circ}}$
60*h*	5807.960	17212.98	*z* ${\;}^{3}F_{3}^{{}^{\circ}}$—*e* ^5^G_4_
2	5810.87	17204.36	*b* ^3^P_1_—*z* ${\;}^{5}S_{2}^{{}^{\circ}}$
2	5813.014	17198.01	*z* ${\;}^{5}G_{4}^{{}^{\circ}}$—*f* ^5^F_4_
400	5814.988	17192.17	*b* ^3^F_4_—*z* ${\;}^{5}D_{4}^{{}^{\circ}}$
1	5825.29	17161.77	*z* ${\;}^{3}F_{3}^{{}^{\circ}}$—*e* ^5^G_3_
30	5825.846	17160.13	*a* ^5^P_1_—*z* ${\;}^{7}D_{1}^{{}^{\circ}}$
100	5828.075	17153.57	*a* ^3^D_2_—z ${\;}^{3}D_{2}^{{}^{\circ}}$
50	5828.408	17152.59	$\left\{ \;\begin{array}{l} a^{1}G_{4}—z{\;}^{3}G_{3}^{{}^{\circ}}\\ d{\;}^{3}F_{4}—4_{3}^{{}^{\circ}} \end{array}\right.$
10	5830.844	17145.42	*d* ^3^F_3_—*y* ${\;}^{3}H_{4}^{{}^{\circ}}$
100	5833.197	17138.51	*z* ${\;}^{3}F_{4}^{{}^{\circ}}$—B_4_
30	5864.624	17046.67	*b* ^1^G_4_— $1_{4}^{{}^{\circ}}$
6	5874.378	17018.36	*b* ^3^F_2_—*z* ${\;}^{5}D_{2}^{{}^{\circ}}$
4	5875.797	17014.25	*c* ^3^F_3_—*y* ${\;}^{3}F_{3}^{{}^{\circ}}$
2*h*	5886.611	16983.00	*z* ${\;}^{7}D_{3}^{{}^{\circ}}$—*e* ^5^F_4_
40	5887.161	16981.41	*a* ^3^D_3_—*z* ${\;}^{3}F_{2}^{{}^{\circ}}$
10	5887.692	16979.88	*b* ^1^D_2_—*y* ${\;}^{3}F_{2}^{{}^{\circ}}$
10	5888.383	16977.89	*a* ^5^D_2_—*z* ${\;}^{7}D_{3}^{{}^{\circ}}$
15*H*	5888.74	16976.86	*b* ^3^P_1_—*z* ${\;}^{5}G_{2}^{{}^{\circ}}$
4	5891.95	16967.61	*b* ^3^D_3_—*w* ${\;}^{3}F_{3}^{{}^{\circ}}$
8	5894.721	16959.63	*z* ${\;}^{3}D_{1}^{{}^{\circ}}$—*e* ^7^D_2_
1	5896.488	16954.55	*z* ${\;}^{5}P_{1}^{{}^{\circ}}$—*f* ^5^D_2_
15	5896.894	16953.38	$\left\{ \;\begin{array}{l} a{\;}^{3}G_{5}—z{\;}^{7}F_{6}^{{}^{\circ}}\\ d{\;}^{3}P_{2}—v{\;}^{3}D_{3}^{{}^{\circ}} \end{array}\right.$
2	5902.284	16937.90	*z* ${\;}^{7}P_{4}^{{}^{\circ}}$—*e* ^5^D_3_
80	5904.426	16931.76	*b* ^3^H_5_—*y* ${\;}^{3}G_{5}^{{}^{\circ}}$
60	5914.226	16903.70	*y* ${\;}^{5}D_{4}^{{}^{\circ}}$—*f* ^5^D_4_
200	5919.340	16889.10	*a* ^5^D_3_—*z* ${\;}^{7}D_{4}^{{}^{\circ}}$
500	5921.450	16883.08	*a* ^3^P_2_—z ${\;}^{5}D_{3}^{{}^{\circ}}$
6*h*	5924.362	16874.78	*z* ${\;}^{3}G_{3}^{{}^{\circ}}$—*e* ^3^G_4_
150	5926.862	16867.66	*a* ^3^D_1_—*z* ${\;}^{3}D_{1}^{{}^{\circ}}$
6	5928.362	16863.39	*b* ^3^G_3_—*x* ${\;}^{3}G_{4}^{{}^{\circ}}$
2	5929.630	16859.79	*z* ${\;}^{7}D_{3}^{{}^{\circ}}$—*f* ^3^F_4_
100	5932.384	16851.96	$\left\{ \;\begin{array}{l} b{\;}^{3}F_{3}—z{\;}^{5}D_{3}^{{}^{\circ}}\\ a{\;}^{3}F_{2}—z{\;}^{7}D_{3}^{{}^{\circ}} \end{array}\right.$
150	5936.644	16839.87	*z* ${\;}^{3}F_{4}^{{}^{\circ}}$—*e* ^5^D_3_
40	5938.580	16834.38	*z* ${\;}^{7}P_{4}^{{}^{\circ}}$—*e* ^3^G_5_
20	5943.728	16819.80	*d* ^3^F_4_—*y* ${\;}^{3}H_{5}^{{}^{\circ}}$
50	5944.386	16817.94	*a* ^1^I_6_—*x* ${\;}^{1}H_{5}^{{}^{\circ}}$
125	5951.154	16798.81	*a* ^3^D_2_—*z* ${\;}^{3}G_{3}^{{}^{\circ}}$
30	5970.544	16744.26	*b* ^3^G_5_—*x* ${\;}^{3}F_{4}^{{}^{\circ}}$
400	5973.370	16736.33	*z* ${\;}^{3}F_{4}^{{}^{\circ}}$—*e* ^3^G_5_
100	5973.672	16735.49	*b* ^3^H_6_—*z* ${\;}^{3}H_{6}^{{}^{\circ}}$
150	5974.162	16734.11	*c* ^3^P_2_—*x* ${\;}^{5}D_{2}^{{}^{\circ}}$
1*h*	5978.022	16723.31	*a* ^1^P_1_—*y* ${D^{3}}_{2}^{{}^{\circ}}$
10	5980.643	16715.98	*c* ^3^P_1_—*y* ${\;}^{5}P_{2}^{{}^{\circ}}$
8	5982.243	16711.51	*z* ${\;}^{5}G_{5}^{{}^{\circ}}$—*e* ^5^G_6_
2*h*	5984.966	16703.91	*b* ^3^D_2_— $9_{1}^{{}^{\circ}}$
200	5988.676	16693.56	*a* ^5^P_3_—*z* ${\;}^{7}D_{4}^{{}^{\circ}}$
2*h*	5989.594	16691.00	*z* ${\;}^{3}G_{3}^{{}^{\circ}}$—*ƒ* ^5^F_4_
3	5991.194	16686.54	*d* ^3^F_4_—*x* ${\;}^{3}G_{3}^{{}^{\circ}}$
2	5991.485	16685.73	*c* ^3^P_2_—*y* ${\;}^{3}D_{1}^{{}^{\circ}}$
20	5992.580	16682.68	*a* ^1^D_2_—*y* ${\;}^{5}D_{2}^{{}^{\circ}}$
150	5993.658	16679.68	*a* ^3^P_1_—*z* ${\;}^{5}D_{2}^{{}^{\circ}}$
4	6002.889	16654.03	*c* ^3^F_4_—*z* ${\;}^{3}H_{5}^{{}^{\circ}}$
30	6003.872	16651.31	$\{\;\begin{array}{c} a{\;}^{3}G_{4}—z{\;}^{7}F_{5}^{{}^{\circ}}\\ a{\;}^{5}G_{3}^{{}^{\circ}}—e{\;}^{5}G_{3} \end{array}$
2	6006.972	16642.71	*z* ${\;}^{7}D_{2}^{{}^{\circ}}$—*e* ^3^F_3_
6	6009.108	16636.80	*z* ${\;}^{3}G_{3}^{{}^{\circ}}$—*e* ^3^D_3_
6	6012.776	16626.65	*z* ${\;}^{5}G_{5}^{{}^{\circ}}$—*f* ^5^F_5_
30*h*	6021.840	16601.62	*z* ${\;}^{3}D_{2}^{{}^{\circ}}$—*ƒ* ^5^F_3_
100	6022.302	16600.35	*c* ^3^F_4_—*y* ${\;}^{3}F_{4}^{{}^{\circ}}$
4	6026.344	16589.22	*c* ^3^F_3_—*y* ${\;}^{5}P_{2}^{{}^{\circ}}$
40 *h*	6027.497	16586.04	*y* ${\;}^{5}D_{3}^{{}^{\circ}}$—*e* ^7^D_3_
20	6033.341	16569.98	*y* ${\;}^{5}D_{4}^{{}^{\circ}}$—*e* ^7^D_3_
4*h*	6037.735	16557.92	*z* ${\;}^{3}F_{4}^{{}^{\circ}}$—*f* ^5^F_5_
4	6038.734	16555.18	*a* ^3^P_2_—*w* ${\;}^{3}D_{3}^{{}^{\circ}}$
100	6046.376	16534.25	*a* ^1^D_2_—*z* ${\;}^{3}D_{1}^{{}^{\circ}}$
20	6054.071	16513.24	*z* ${\;}^{3}D_{2}^{{}^{\circ}}$—*e* ^5^D_2_
4*h*	6057.453	16504.02	*z* ${\;}^{3}F_{2}^{{}^{\circ}}$—*e* ^3^G_3_
20	6065.189	16482.97	*z* ${\;}^{5}P_{2}^{{}^{\circ}}$—*f* ^5^D_3_
10	6073.90	16459.33	$\{\;\begin{array}{c} a{\;}^{3}D_{1}—z{\;}^{3}F_{2}^{{}^{\circ}}\\ d{\;}^{3}F_{3}—x{\;}^{3}G_{3}^{{}^{\circ}} \end{array}$
1*h*	6077.79	16448.79	*y* ${\;}^{5}D_{1}^{{}^{\circ}}$—*e* ^7^D_2_
2	6078.831	16445.98	*b* ^3^H_4_—*y* ${\;}^{3}G_{5}^{{}^{\circ}}$
200	6080.08	16442.60	*z* ${\;}^{3}D_{3}^{{}^{\circ}}$—B_4_
60	6083.522	16433.30	*c* ^3^P_2_—*x* ${\;}^{5}D_{3}^{{}^{\circ}}$
50	6085.121	16428.98	*b* ^3^H_4_—*y* ${\;}^{3}F_{3}^{{}^{\circ}}$
10	6089.426	16417.37	*z* ${\;}^{3}F_{3}^{{}^{\circ}}$—*ƒ* ^5^F_3_
300*h*	6090.522	16414.41	*z* ${\;}^{3}D_{3}^{{}^{\circ}}$—*e* ^3^P_2_
15	6101.560	16384.72	*a* ^1^D_2_—*y* ${\;}^{5}D_{3}^{{}^{\circ}}$
100	6108.97	16364.84	*a* ^3^G_5_—*z* ${\;}^{5}G_{6}^{{}^{\circ}}$
300	6116.770	16343.97	$\{\;\begin{array}{c} a{\;}^{1}G_{4}—z{\;}^{3}D_{3}^{{}^{\circ}}\\ b{\;}^{3}F_{4}—z{\;}^{7}D_{4}^{{}^{\circ}} \end{array}$
60	6119.844	16335.76	*z* ${\;}^{3}F_{3}^{{}^{\circ}}$—*e* ^3^G_4_
6	6121.032	16332.59	*d* ^3^P_2_—*x* ${\;}^{3}F_{3}^{{}^{\circ}}$
50	6122, 376	16329.01	*z* ${\;}^{3}F_{3}^{{}^{\circ}}$—*e* ^5^D_2_
1	6137.77	16288.05	$\{\;\begin{array}{c} a{\;}^{3}G_{5}—z{\;}^{3}G_{5}^{{}^{\circ}}\\ y{\;}^{5}D_{2}^{{}^{\circ}}—e{\;}^{7}D_{3} \end{array}$
60	6141.527	16278.09	*z* ${\;}^{5}P_{3}^{{}^{\circ}}$—*ƒ* ^5^D_4_
10	6150.073	16255.47	*d* ^3^P_2_—*x* ${\;}^{3}F_{2}^{{}^{\circ}}$
6	6170.237	16202.35	*z* ${\;}^{5}F_{5}^{{}^{\circ}}$—*e* ^5^F_4_
200	6170.605	16201.38	*a* ^3^D_3_—*z* ${\;}^{3}F_{3}^{{}^{\circ}}$
10	6173.127	16194.76	*y* ${\;}^{5}F_{3}^{{}^{\circ}}$—*ƒ* ^5^D_2_
50	6174.210	16191.92	*a* ^3^G_3_—*z* ${\;}^{3}G_{4}^{{}^{\circ}}$
100	6176.774	16185.20	*c* ^3^P_2_—*z* ${\;}^{3}P_{2}^{{}^{\circ}}$
10	6177.736	16182.68	*d* ^3^F_2_—*y* ${\;}^{1}F_{3}^{{}^{\circ}}$
4	6179.973	16176.82	*z* ${\;}^{5}P_{2}^{{}^{\circ}}$—*ƒ* ^7^D_2_
10	6185.835	16161.49	*z* ${\;}^{5}F_{4}^{{}^{\circ}}$—*e* ^5^F_3_
10	6189.488	16151.96	*z* ${\;}^{3}F_{3}^{{}^{\circ}}$—*ƒ* ^5^F_4_
300	6192.552	16143.96	*z* ${\;}^{3}D_{3}^{{}^{\circ}}$—*e* ^5^D_3_
30	6195.33	16136.73	*b* ^3^G_5_—*x* ${\;}^{3}G_{5}^{{}^{\circ}}$
20	6195.97	16135.06	*b* ^3^H_6_—*z* ${\;}^{3}H_{5}^{{}^{\circ}}$
10	6197.15	16131.99	*a* ^3^D_2_—*z* ${\;}^{5}S_{2}^{{}^{\circ}}$
400	6199.42	16126.08	*a* ^1^D_2_—*z* ${\;}^{3}F_{2}^{{}^{\circ}}$
30*h*	6210.31	16097.80	*z* ${\;}^{3}F_{3}^{{}^{\circ}}$—*e* ^3^D_3_
50	6210.75	16096.66	*b* ^3^G_4_—*x* ${\;}^{3}G_{5}^{{}^{\circ}}$
4	6213.63	16089.20	*d* ^3^P_2_—*x* ${\;}^{3}D_{1}^{{}^{\circ}}$
2*H*	6217.35	16079.57	*z* ${\;}^{5}F_{3}^{{}^{\circ}}$—*e* ^3^F_2_
4	6217.52	16079.13	*z* ${\;}^{5}F_{5}^{{}^{\circ}}$—*ƒ* ^3^F_4_
10	6219.58	16073.81	*a* ^3^G_4_—*z* ${\;}^{5}F_{3}^{{}^{\circ}}$
300	6225.22	16059.25	*b* ^3^F_2_—*z* ${\;}^{5}D_{3}^{{}^{\circ}}$
10	6225.67	16058.09	*b* ^1^G_4_—*y* ${\;}^{3}G_{5}^{{}^{\circ}}$
20	6231.81	16042.27	*z* ${\;}^{7}D_{5}^{{}^{\circ}}$—*e* ^5^F_5_
6*h*	6233.68	16037.45	*y* ${\;}^{5}F_{2}^{{}^{\circ}}$—*f* ^5^D_2_
100	6235.63	16032.44	*b* ^3^H_4_—*z* ${\;}^{1}G_{4}^{{}^{\circ}}$
10Λ	6235.97	16031.56	*c* ^3^P_2_—*y* ${\;}^{3}D_{2}^{{}^{\circ}}$
10	6241.62	16017.05	*a* ^3^D_3_—*z* ${\;}^{3}D_{2}^{{}^{\circ}}$
150	6252.067	15990.29	*a* ^3^D_2_—*z* ${\;}^{3}D_{3}^{{}^{\circ}}$
6	6255.293	15982.04	*a* ^l^F_3_—*x* ${\;}^{3}F_{2}^{{}^{\circ}}$
6	6258.00	15975.13	*b* ^3^G_5_—*z* ${\;}^{3}I_{6}^{{}^{\circ}}$
4	6270.07	15944.38	$\{\;\begin{array}{c} b{\;}^{3}D_{3}—t{\;}^{3}D_{2}^{{}^{\circ}}\\ z{\;}^{5}P_{3}^{{}^{\circ}}—e{\;}^{7}D_{3} \end{array}$
20	6274.503	15933.11	*z* ${\;}^{3}D_{1}^{{}^{\circ}}$—*e* ^5^D_1_
4	6275.12	15931.54	*b* ^3^G_3_—*x* ${\;}^{3}D_{2}^{{}^{\circ}}$
100	6284.49	15907.79	*c* ^3^P_1_—*x* ${\;}^{5}D_{1}^{{}^{\circ}}$
6	6285.68	15904.78	*a* ^3^D_2_—*z* ${\;}^{5}G_{2}^{{}^{\circ}}$
4	6289.34	15895.52	*a* ^3^G_3_—*z* ${\;}^{7}F_{4}^{{}^{\circ}}$
50	6290.100	15893.60	*y* ${\;}^{5}F_{4}^{{}^{\circ}}$—*f* ^5^D_3_
300	6295.220	15880.68	*b* ^1^G_4_—*z* ${\;}^{3}H_{4}^{{}^{\circ}}$
30	6303.242	15860.47	*y* ${\;}^{5}D_{3}^{{}^{\circ}}$—*e* ^5^P_3_
40	6304.49	15857.33	*c* ^3^F_2_—*x* ${\;}^{5}D_{1}^{{}^{\circ}}$
10	6306.79	15851.54	*z* ${\;}^{5}G_{1}^{{}^{\circ}}$—*e* ^3^F_2_
2	6307.60	15849.51	*b* ^3^D_2_—*x* ${\;}^{1}F_{3}^{{}^{\circ}}$
60	6309.66	15844.33	*y* ${\;}^{5}D_{4}^{{}^{\circ}}$—*e* ^5^P_3_
4	6310.37	15842.55	*z* ${\;}^{5}G_{4}^{{}^{\circ}}$—*e* ^5^D_3_
50	6311.10	15840.72	*d* ^3^P_2_—*x* ${\;}^{3}D_{3}^{{}^{\circ}}$
4	6311.39	15839.99	*b* ^3^G_4_—*w* ${\;}^{3}D_{3}^{{}^{\circ}}$
60	6316.73	15826.60	*c* ^3^P_2_—*y* ${\;}^{3}D_{3}^{{}^{\circ}}$
50	6324.24	15807.81	*a* ^3^G_5_—*z* ${\;}^{5}F_{4}^{{}^{\circ}}$
150	6330.62	15791.88	*z* ${\;}^{7}D_{4}^{{}^{\circ}}$—*e* ^5^F_5_
3	6331.52	15789.63	*z* ${\;}^{7}D_{3}^{{}^{\circ}}$—*e* ^3^F_4_
30	6336.10	15778.22	*b* ^3^H_5_—*z* ${\;}^{3}H_{5}^{{}^{\circ}}$
20	6343.07	15760.88	*y* ${\;}^{5}D_{2}^{{}^{\circ}}$—*e* ^5^S_2_
20	6351.90	15738.97	*z* ${\;}^{5}G_{4}^{{}^{\circ}}$—*e* ^3^G_5_
15	6357.75	15724.49	*b* ^3^H_5_—*y* ${\;}^{3}F_{4}^{{}^{\circ}}$
150	6357.95	15723.99	*c* ^3^F_4_—*x* ${\;}^{5}D_{3}^{{}^{\circ}}$
100	6363.41	15710.50	*z* ${\;}^{5}D_{2}^{{}^{\circ}}$—*e* ^5^F_3_
100	6376.45	15678.37	*a* ^3^G_4_—*z* ${\;}^{3}G_{5}^{{}^{\circ}}$
60	6382.98	15662.33	*a* ^3^D_3_—*z* ${\;}^{3}G_{3}^{{}^{\circ}}$
50	6384.66	15658.21	*b* ^3^F_3_—*z* ${\;}^{5}D_{4}^{{}^{\circ}}$
15	6388.06	15649.88	*d* ^3^F_4_—*x* ${\;}^{3}G_{4}^{{}^{\circ}}$
1	6388.79	15648.09	*a* ^1^G_4_—*z* ${\;}^{3}F_{4}^{{}^{\circ}}$
80	6390.21	15644.61	*b* ^1^G_4_—*z* ${\;}^{1}G_{4}^{{}^{\circ}}$
2	6395.89	15630.72	*a* ^3^D_1_—*z* ${\;}^{7}P_{2}^{{}^{\circ}}$
5	6401.40	15617.27	*b* ^3^G_4_—*x* ${\;}^{3}F_{3}^{{}^{\circ}}$
2	6404.86	15608.83	*z* ${\;}^{5}D_{3}^{{}^{\circ}}$—*e* ^3^F_3_
10	6406.07	15605.88	*z* ${\;}^{3}G_{3}^{{}^{\circ}}$—*e* ^3^P_2_
2	6413.33	15588.22	*b* ^3^P_1_—*z* ${\;}^{5}D_{0}^{{}^{\circ}}$
100	6417.56	15577.94	*y* ${\;}^{5}F_{5}^{{}^{\circ}}$—*f* ^5^D_4_
10	6429.55	15548.89	*z* ${\;}^{3}F_{2}^{{}^{\circ}}$—*e* ^5^D_2_
6	6432.10	15542, 73	*z* ${\;}^{5}F_{2}^{{}^{\circ}}$—*e* ^3^F_2_
200	6444.84	15512.00	*z* ${\;}^{5}D_{3}^{{}^{\circ}}$—*e* ^5^F_4_
4	6445.85	15509.57	*c* ^3^P_1_—*x* ${\;}^{5}D_{0}^{{}^{\circ}}$
4	6451.88	15495.08	*a* ^3^D_1_—*z* ${\;}^{3}D_{2}^{{}^{\circ}}$
8	6456.91	15483.01	*a* ^3^D_2_—*z* ${\;}^{5}G_{3}^{{}^{\circ}}$
10	6457.43	15481.76	*d* ^3^F_3_—*y* ${\;}^{1}F_{3}^{{}^{\circ}}$
4	6461.19	15472.75	*b* ^3^P_2_—*z* ${\;}^{5}D_{1}^{{}^{\circ}}$
8	6472.51	15445.69	*b* ^3^P_1_—*z* ${\;}^{5}F_{2}^{{}^{\circ}}$
4	6482.22	15422.55	*d* ^3^F_3_—*x* ${\;}^{3}G_{4}^{{}^{\circ}}$
12	6484.49	15417.15	*z* ${\;}^{5}P_{3}^{{}^{\circ}}$—*e* ^5^S_2_
3	6492.36	15398.47	*y* ${\;}^{5}D_{1}^{{}^{\circ}}$—*e* ^5^S_2_
100	6496.43	15388.82	*z* ${\;}^{5}D_{3}^{{}^{\circ}}$—*f* ^3^F_4_
2	6499.78	15380.89	*b* ^3^F_3_—*z* ${\;}^{7}D_{3}^{{}^{\circ}}$
30	6519.06	15335.40	*z* ${\;}^{3}G_{3}^{{}^{\circ}}$—*e* ^5^D_3_
2	6531.60	15305.96	*b* ^3^G_4_—*z* ${\;}^{1}F_{3}^{{}^{\circ}}$
70	6540.24	15285.74	*z* ${\;}^{5}F_{3}^{{}^{\circ}}$—*e* ^5^F_3_
15	6544.23	15276.42	$\{\;\begin{array}{c} c{\;}^{3}F_{2}—y{\;}^{3}D_{1}^{{}^{\circ}}\\ z{\;}^{5}F_{1}^{{}^{\circ}}—e{\;}^{3}F_{2} \end{array}$
2	6556.24	15248.43	*c* ^3^F_3_—*x* ${\;}^{5}D_{2}^{{}^{\circ}}$
2	6557.83	15244.74	*b* ^3^P_2_—*z* ${\;}^{5}F_{3}^{{}^{\circ}}$
6	6559.46	15240.95	*y* ${\;}^{3}D_{3}^{{}^{\circ}}$—*f* ^5^D_2_
60	6560.45	15238.65	*b* ^3^H_4_—*y* ${\;}^{3}F_{4}^{{}^{\circ}}$
15	6569.09	15218.61	*z* ${\;}^{5}P_{3}^{{}^{\circ}}$—*e* ^5^P_3_
2	6577.96	15198.08	*a* ^3^G_4_—*z* ${\;}^{5}F_{4}^{{}^{\circ}}$
30	6593.75	15161.69	*a* ^1^D_2_—*z* ${\;}^{3}D_{2}^{{}^{\circ}}$
10	6596.58	15155.19	*a* ^3^D_3_—*z* ${\;}^{5}G_{4}^{{}^{\circ}}$
10	6613.11	15117.30	*c* ^3^F_4_—*y* ${\;}^{3}D_{3}^{{}^{\circ}}$
50	6618.22	15105.63	*a* ^3^H_5_—*z* ${\;}^{5}G_{4}^{{}^{\circ}}$
2	6620.40	15100.66	*z* ${\;}^{5}F_{4}^{{}^{\circ}}$—*e* ^3^F_3_
3	6633.40	15071.06	*z* ${\;}^{5}P_{2}^{{}^{\circ}}$—E_1_
10	6635.27	15066.82	*z* ${\;}^{3}F_{3}^{{}^{\circ}}$—*e* ^3^P_2_
6	6640.88	15054.09	*c* ^3^P_1_—*z* ${\;}^{3}P_{1}^{{}^{\circ}}$
7	6649.54	15034.48	*a* ^1^F_3_—*y* ${\;}^{3}G_{3}^{{}^{\circ}}$
7	6651.47	15030.12	*c* ^3^P_2_—*y* ${\;}^{5}F_{2}^{{}^{\circ}}$
2	6658.83	15013.51	*b* ^3^G_5_—*z* ${\;}^{1}H_{5}^{{}^{\circ}}$
200	6663.16	15003.75	*z* ${\;}^{5}F_{4}^{{}^{\circ}}$—*e* ^5^F_4_
10	6664.10	15001.64	*z* ${\;}^{5}F_{3}^{{}^{\circ}}$—*e* ^5^F_2_
3	6669.72	14989.00	*d* ^3^P_2_—*y* ${\;}^{3}P_{1}^{{}^{\circ}}$
1	6676.62	14973.50	*b* ^3^G_4_—*z* ${\;}^{1}H_{5}^{{}^{\circ}}$
2	6688.22	14947.53	*c* ^3^F_3_—*x* ${\;}^{5}D_{3}^{{}^{\circ}}$
400	6690.01	14943.54	*z* ${\;}^{5}D_{4}^{{}^{\circ}}$—*e* ^5^F_5_
20	6702.08	14916.62	*z* ${\;}^{5}F_{2}^{{}^{\circ}}$—*e* ^5^F_1_
2	6706.78	14906.17	*z* ${\;}^{5}P_{1}^{{}^{\circ}}$—F_1_
30	6707.56	14904.44	*b* ^1^G_4_—*z* ${\;}^{3}H_{5}^{{}^{\circ}}$
2	6717.57	14882.23	*b* ^3^D_2_—*x* ${\;}^{3}P_{2}^{{}^{\circ}}$
80	6718.31	14880.59	*z* ${\;}^{5}F_{4}^{{}^{\circ}}$—*ƒ* ^3^F_4_
2	6718.91	14879.26	*y* ${\;}^{5}F_{4}^{{}^{\circ}}$—*f* ^5^D_4_
7	6721.81	14872.84	*c* ^3^P_2_—*y* ${\;}^{5}F_{3}^{{}^{\circ}}$
1	6724.87	14866.07	*b* ^3^P_0_—*z* ${\;}^{5}F_{1}^{{}^{\circ}}$
80	6730.46	14853.72	*a* ^3^D_3_—*z* ${\;}^{3}D_{3}^{{}^{\circ}}$
20	6731.79	14850.79	*b* ^1^G_4_—*y* ${\;}^{3}F_{4}^{{}^{\circ}}$
3	6733.95	14846.03	*d* ^3^P_2_—*y* ${\;}^{5}P_{1}^{{}^{\circ}}$
2	6738.12	14836.84	*b* ^3^G_5_— $1_{4}^{{}^{\circ}}$
10	6742.92	14826.28	*c* ^3^P_1_—*z* ${\;}^{3}P_{2}^{{}^{\circ}}$
2	6750.39	14809.87	*b* ^3^F_3_—*z* ${\;}^{7}D_{4}^{{}^{\circ}}$
10	6751.73	14806.93	*a* ^1^D_2_—*z* ${\;}^{3}G_{3}^{{}^{\circ}}$
100	6756.54	14796.39	$\{\;\begin{array}{c} z{\;}^{3}F_{3}^{{}^{\circ}}—e{\;}^{5}D_{3}\\ b{\;}^{3}G_{4}—1_{4}^{{}^{\circ}} \end{array}$
50	6766.77	14774.02	*z* ${\;}^{5}D_{0}^{{}^{\circ}}$—*e* ^5^F_1_
150	6766.98	14773.56	*z* ${\;}^{5}D_{1}^{{}^{\circ}}$—*e* ^5^F_2_
2	6770.15	14766.65	*a* ^3^G_3_—*z* ${\;}^{5}D_{2}^{{}^{\circ}}$
100	6775.01	14756.05	*a* ^3^H_4_—*z* ${\;}^{3}G_{3}^{{}^{\circ}}$
1*h*	6778.30	14748.89	*z* ${\;}^{5}F_{2}^{{}^{\circ}}$—*e* ^5^F_3_
2	6779.15	14747.04	*b* ^1^D_2_—*x* ${\;}^{5}D_{1}^{{}^{\circ}}$
60	6787.20	14729.55	*a* ^3^H_6_—*z* ${\;}^{5}G_{5}^{{}^{\circ}}$
1	6792.94	14717.10	*a* ^1^H_5_—*y* ${\;}^{5}F_{5}^{{}^{\circ}}$
8	6805.53	14689.88	*a* ^3^G_4_—*z* ${\;}^{5}D_{3}^{{}^{\circ}}$
1	6807.00	14686.71	*a* ^3^P_1_—*z* ${\;}^{7}D_{2}^{{}^{\circ}}$
8	6812.90	14673.99	*b* ^3^G_5_—*y* ${\;}^{3}G_{4}^{{}^{\circ}}$
40	6813.52	14672.65	*c* ^3^P_1_—*y* ${\;}^{3}D_{2}^{{}^{\circ}}$
3	6821.73	14654.99	*y* ${\;}^{5}F_{2}^{{}^{\circ}}$—*e* ^7^D_1_
100	6823.88	14650.38	*z* ${\;}^{5}F_{1}^{{}^{\circ}}$—*e* ^5^F_1_
150	6824.17	14649.75	*z* ${\;}^{5}D_{2}^{{}^{\circ}}$—*e* ^3^F_3_
20	6831.44	14634.17	*x* ${\;}^{5}D_{3}^{{}^{\circ}}$—*ƒ* ^5^D_2_
30	6831.54	14633.95	*b* ^3^G_4_—*y* ${\;}^{3}G_{4}^{{}^{\circ}}$
1	6832.71	14631.45	*z* ${\;}^{5}P_{2}^{{}^{\circ}}$—*e* ^5^D_1_
1	6834.11	14628.45	*b* ^3^D_3_—*x* ${\;}^{5}F_{4}^{{}^{\circ}}$
3	6837.04	14622.18	*c* ^3^F_2_—*y* ${\;}^{3}D_{2}^{{}^{\circ}}$
2	6843.13	14609.17	*a* ^3^G_5_—*z* ${\;}^{5}F_{5}^{{}^{\circ}}$
1	6843.83	14607.67	*z* ${\;}^{5}P_{2}^{{}^{\circ}}$—*e* ^5^S_2_
5	6850.84	14592.72	$\{\;\begin{array}{l} b{\;}^{3}G_{4}—y{\;}^{3}G_{3}^{{}^{\circ}}\\ y{\;}^{5}G_{5}^{{}^{\circ}}—E_{4} \end{array}$
1	6852.93	14588.27	*b* ^3^F_2_—*z* ${\;}^{7}D_{3}^{{}^{\circ}}$
1*h*	6858.76	14575.87	*y* ${\;}^{5}F_{2}^{{}^{\circ}}$—*e* ^7^D_2_
20	6872.92	14545.84	*c* ^3^F_3_—*y* ${\;}^{3}D_{2}^{{}^{\circ}}$
1	6875.15	14541.13	*a* ^1^F_3_—*y* ${\;}^{3}P_{2}^{{}^{\circ}}$
3	6883.51	14523.47	*z* ${\;}^{3}G_{5}^{{}^{\circ}}$—*e* ^5^F_4_
1	6891.70	14506.21	*d* ^3^P_2_—*y* ${\;}^{3}F_{3}^{{}^{\circ}}$
6	6902.25	14484.03	*b* ^3^P_1_—*z* ${\;}^{5}D_{2}^{{}^{\circ}}$
1	6908.25	14471.45	*z* ${\;}^{5}P_{3}^{{}^{\circ}}$—*ƒ* ^5^F_4_
200	6911.46	14464.73	*z* ${\;}^{5}F_{2}^{{}^{\circ}}$—*e* ^5^F_2_
1	6912.47	14462.62	*y* ${\;}^{5}F_{5}^{{}^{\circ}}$—*e* ^7^D_4_
20	6918.50	14450.01	*d* ^3^F_4_—*x* ${\;}^{3}G_{5}^{{}^{\circ}}$
2	6920.13	14446.61	*d* ^3^P_1_—*x* ${\;}^{3}D_{2}^{{}^{\circ}}$
4	6921.97	14442.77	*z* ${\;}^{5}P_{1}^{{}^{\circ}}$—*e* ^5^S_2_
800	6923.21	14440.18	*z* ${\;}^{5}F_{5}^{{}^{\circ}}$—*e* ^5^F_5_
2	6926.67	14432.97	*z* ${\;}^{5}G_{3}^{{}^{\circ}}$—*e* ^3^F_2_
1	6934.20	14417.30	$\{\;\begin{array}{c} c{\;}^{3}F_{2}—y{\;}^{3}D_{3}^{{}^{\circ}}\\ z{\;}^{5}P_{3}^{{}^{\circ}}—e{\;}^{5}D_{3} \end{array}$
3	6938.13	14409.13	*z* ${\;}^{5}P_{2}^{{}^{\circ}}$—*e* ^5^P_3_
1*h*	6952.10	14380.18	*y* ${\;}^{5}F_{1}^{{}^{\circ}}$—*e* ^7^D_1_
3	6958.34	14367.28	*d* ^3^F_2_—*x* ${\;}^{3}F_{2}^{{}^{\circ}}$
3	6958.85	14366.23	*y* ${\;}^{5}F_{3}^{{}^{\circ}}$—*e* ^5^P_2_
1	6959.78	14364.31	*d* ^3^F_3_—*v* ${\;}^{3}D_{3}^{{}^{\circ}}$
20	6960.76	14362.29	*b* ^3^H_4_—*x* ${\;}^{5}D_{3}^{{}^{\circ}}$
1	6963.06	14357.54	*b* ^3^G_3_—*y* ${\;}^{3}F_{2}^{{}^{\circ}}$
2	6968.43	14346.48	*a* ^3^D_3_—*z* ${\;}^{5}G_{3}^{{}^{\circ}}$
1	6971.12	14340.94	*c* ^3^F_3_—*y* ${\;}^{3}D_{3}^{{}^{\circ}}$
300	6981.98	14318.64	*z* ${\;}^{5}D_{3}^{{}^{\circ}}$—*e* ^3^F_4_
3	6995.49	14290.98	*b* ^3^P_0_—*z* ${\;}^{5}D_{1}^{{}^{\circ}}$
1*h*	6997.56	14286.76	*z* ${\;}^{3}F_{2}^{{}^{\circ}}$—*e* ^3^P_2_
4	6998.27	14285.31	*z* ${\;}^{3}G_{4}^{{}^{\circ}}$—*e* ^5^F_3_
1	7012.87	14255.57	*b* ^3^D_3_—*x* ${\;}^{3}P_{2}^{{}^{\circ}}$
3	7016.13	14248.94	*a* ^3^H_4_—*z* ${\;}^{5}G_{4}^{{}^{\circ}}$
4	7017.46	14246.24	*a* ^3^D_1_—*z* ${\;}^{5}C_{2}^{{}^{\circ}}$
400	7027.95	14224.98	*z* ${\;}^{5}F_{3}^{{}^{\circ}}$—*e* ^3^F_3_
3	7033.12	14214.52	*b* ^1^D_2_—*x* ${\;}^{5}D_{2}^{{}^{\circ}}$
10*h*	7034.47	14211.79	*y* ${\;}^{3}F_{4}^{{}^{\circ}}$—D_3_
2	7035.87	14208.97	*y* ${\;}^{5}F_{2}^{{}^{\circ}}$—*e* ^5^P_2_
2	7039.85	14200.93	*b* ^3^G_3_—*y* ${\;}^{3}G_{4}^{{}^{\circ}}$
1	7042.49	14195.61	*d* ^3^P_1_—*z* ${\;}^{1}D_{2}^{{}^{\circ}}$
9	7057.12	14166.18	*b* ^1^D_2_—*y* ${\;}^{3}D_{1}^{{}^{\circ}}$
10	7058.42	14163.57	*c* ^3^F_4_—*y* ${\;}^{5}F_{3}^{{}^{\circ}}$
60	7060.36	14159.68	*b* ^3^G_3_—*y* ${\;}^{3}G_{3}^{{}^{\circ}}$
10*h*	7061.12	14158.16	*z* ${\;}^{7}F_{2}^{{}^{\circ}}$—*e* ^5^F_3_
100	7061.25	14157.90	*a* ^3^D_3_—*z* ${\;}^{3}F_{4}^{{}^{\circ}}$
1	7061.80	14156.79	*b* ^3^D_2_—*u* ${\;}^{3}D_{2}^{{}^{\circ}}$
8	7070.15	14140.07	*a* ^1^D_2_—*z* ${\;}^{5}S_{2}^{{}^{\circ}}$
1	7073.70	14132.98	*d* ^3^F_2_—*z* ${\;}^{1}F_{3}^{{}^{\circ}}$
1	7076.16	14128.06	*z* ${\;}^{5}F_{3}^{{}^{\circ}}$—*e* ^5^F_4_
8	7083.69	14113.05	*c* ^3^P_2_—*z* ${\;}^{5}P_{1}^{{}^{\circ}}$
200	7086.04	14108.37	*a* ^3^H_5_—*z* ${\;}^{3}F_{4}^{{}^{\circ}}$
200	7087.32	14105.82	*a* ^3^G_5_—*z* ${\;}^{5}D_{4}^{{}^{\circ}}$
2	7104.15	14072.40	*a* ^1^F_3_—*z* ${\;}^{3}H_{4}^{{}^{\circ}}$
20*h*	7110.57	14059.69	*a* ^3^D_3_—*z* ${\;}^{7}P_{4}^{{}^{\circ}}$
1*h*	7120.43	14040.23	*y* ${\;}^{5}D_{4}^{{}^{\circ}}$—B_4_
2	7120.74	14039.61	*a* ^3^H_5_—*z* ${\;}^{5}G_{5}^{{}^{\circ}}$
15	7135.17	14011.22	*z* ${\;}^{5}G_{2}^{{}^{\circ}}$—*e* ^3^F_2_
20	7135.64	14010.30	*a* ^3^H_5_—*z* ${\;}^{7}P_{4}^{{}^{\circ}}$
6	7140.81	14000.15	*z* ${\;}^{7}F_{3}^{{}^{\circ}}$—*e* ^5^F_2_
70	7141.71	13998.39	*a* ^1^D_2_—*z* ${\;}^{3}D_{3}^{{}^{\circ}}$
3	7146.41	13989.18	*y* ${\;}^{5}F_{2}^{{}^{\circ}}$—F_1_
2*h*	7165.83	13951.27	*y* ${\;}^{5}F_{3}^{{}^{\circ}}$—*e* ^3^D_2_
15	7167.76	13947.52	*a* ^3^H_4_—*z* ${\;}^{3}D_{3}^{{}^{\circ}}$
5	7168.46	13946.15	*c* ^3^P_1_—*y* ${\;}^{5}F_{1}^{{}^{\circ}}$
2	7171.75	13939.76	*z* ${\;}^{3}P_{2}^{{}^{\circ}}$—*ƒ* ^7^D_2_
2*h*	7178.94	13925.80	*z* ${\;}^{7}P_{4}^{{}^{\circ}}$—*e* ^5^F_3_
15	7182.20	13919.47	*a* ^1^I_6_—*y* ${\;}^{1}H_{5}^{{}^{\circ}}$
30	7183.01	13917.91	*a* ^3^H_6_—*z* ${\;}^{7}F_{5}^{{}^{\circ}}$
20	7194.50	13895.68	*c* ^3^F_2_—*y* ${\;}^{5}F_{1}^{{}^{\circ}}$
2	7205.87	13873.75	*z* ${\;}^{7}F_{2}^{{}^{\circ}}$—*e* ^5^F_2_
10	7212.57	13860.86	*b* ^3^P_2_—*z* ${\;}^{5}D_{3}^{{}^{\circ}}$
150	7219.26	13848.02	*b* ^3^G_5_—*y* ${\;}^{3}G_{5}^{{}^{\circ}}$
5	7225.32	13836.40	*a* ^3^D_2_—*z* ${\;}^{5}F_{3}^{{}^{\circ}}$
4	7229.81	13827.81	*z* ${\;}^{3}F_{4}^{{}^{\circ}}$—*e* ^5^F_3_
4	7233.96	13819.88	*y* ${\;}^{5}F_{4}^{{}^{\circ}}$—*e* ^5^P_3_
400	7238.90	13810.45	*z* ${\;}^{5}F_{4}^{{}^{\circ}}$—*e* ^3^F_4_
20	7240.22	13807.93	$\{\;\begin{array}{l} b{\;}^{3}G_{4}—y{\;}^{3}G_{5}^{{}^{\circ}}\\ y{\;}^{3}G_{3}^{{}^{\circ}}—E_{4} \end{array}$
3	7240.41	13807.57	*a* ^3^G_3_—*z* ${\;}^{5}D_{3}^{{}^{\circ}}$
1	7247.12	13794.78	*a* ^1^G_4_—*z* ${\;}^{3}G_{5}^{{}^{\circ}}$
1*h*	7247.54	13793.98	*y* ${\;}^{5}F_{2}^{{}^{\circ}}$—*e* ^3^D_2_
10	7249.10	13791.02	*b* ^3^G_4_—*y* ${\;}^{3}F_{3}^{{}^{\circ}}$
2	7255.16	13779.50	*y* ${\;}^{3}D_{3}^{{}^{\circ}}$—*e* ^7^D_2_
1	7255.47	13778.91	*d* ^3^P_2_—*y* ${\;}^{5}P_{3}^{{}^{\circ}}$
10	7267.76	13755.61	*b* ^3^H_4_—*y* ${\;}^{3}D_{3}^{{}^{\circ}}$
1	7268.02	13755.11	*b* ^3^D_2_—*y* ${\;}^{3}S_{1}^{{}^{\circ}}$
4*h*	7272.24	13747.13	*y* ${\;}^{5}F_{3}^{{}^{\circ}}$—*e* ^5^G_3_
3*h*	7289.76	13714.10	*y* ${\;}^{5}F_{1}^{{}^{\circ}}$—F_1_
2*h*	7290.90	13711.95	*z* ${\;}^{3}P_{1}^{{}^{\circ}}$—*ƒ* ^7^D_2_
20	7297.10	13700.30	*a* ^3^D_3_—*z* ${\;}^{3}G_{4}^{{}^{\circ}}$
2	7306.36	13682.94	*y* ${\;}^{5}F_{3}^{{}^{\circ}}$—*e* ^5^S_2_
30	7312.63	13671.20	*c* ^3^P_1_—*y* ${\;}^{5}F_{2}^{{}^{\circ}}$
20	7315.66	13665.54	*b* ^1^D_2_—*z* ${\;}^{3}P_{2}^{{}^{\circ}}$
4	7318.81	13659.66	*d* ^3^P_2_—*z* ${\;}^{3}S_{1}^{{}^{\circ}}$
7	7322.06	13653.60	*y* ${\;}^{3}D_{2}^{{}^{\circ}}$—*e* ^7^D_1_
150	7323.57	13650.78	*a* ^3^H_5_—*z* ${\;}^{3}G_{4}^{{}^{\circ}}$
3	7326.21	13645.86	*b* ^3^D_2_—*y* ${\;}^{1}F_{3}^{{}^{\circ}}$
10	7329.82	13639.14	*z* ${\;}^{5}G_{3}^{{}^{\circ}}$—*e* ^5^F_3_
1	7334.43	13630.57	*b* ^3^G_4_—*z* ${\;}^{3}H_{4}^{{}^{\circ}}$
10	7338.89	13622.29	*d* ^3^P_1_—*x* ${\;}^{3}F_{2}^{{}^{\circ}}$
1	7339.73	13620.73	*c* ^3^F_2_—*y* ${\;}^{5}F_{2}^{{}^{\circ}}$
9	7341.44	13617.55	*d* ^3^F_2_—*y* ${\;}^{3}F_{2}^{{}^{\circ}}$
1	7343.49	13613.75	*y* ${\;}^{5}F_{5}^{{}^{\circ}}$—*e* ^7^D_5_
40	7348.72	13604.06	$1_{4}^{{}^{\circ}}$—E_4_
2*h*	7356.27	13590.10	*y* ${\;}^{3}D_{3}^{{}^{\circ}}$—*ƒ* ^5^D_4_
100	7377.77	13550.50	*z* ${\;}^{7}F_{5}^{{}^{\circ}}$—*e* ^5^F_4_
40	7381.09	13544.40	*c* ^3^F_3_—*y* ${\;}^{5}F_{2}^{{}^{\circ}}$
2	7391.33	13525.64	$\{\;\begin{array}{c} y{\;}^{3}F_{2}^{{}^{\circ}}—e{\;}^{5}S_{2}\\ y{\;}^{5}D_{4}^{{}^{\circ}}—e{\;}^{5}D_{4} \end{array}$
800	7393.93	13520.88	*z* ${\;}^{7}F_{4}^{{}^{\circ}}$—*e* ^3^F_3_
20	7407.51	13496.10	*a* ^3^G_4_—*z* ${\;}^{5}D_{4}^{{}^{\circ}}$
40	7410.27	13491.07	*a* ^1^D_2_—*z* ${\;}^{5}G_{3}^{{}^{\circ}}$
10	7419.59	13474.12	*x* ${\;}^{5}D_{4}^{{}^{\circ}}$—*e* ^7^D_3_
1	7425.44	13463.51	*c* ^3^F_2_—*y* ${\;}^{5}F_{3}^{{}^{\circ}}$
1*h*	7427.66	13459.48	*y* ${\;}^{5}D_{4}^{{}^{\circ}}$—*ƒ* ^5^F_5_
2	7429.55	13456.06	*d* ^3^P_1_—*x* ${\;}^{3}D_{1}^{{}^{\circ}}$
20	7438.32	13440.20	*a* ^3^H_4_—*z* ${\;}^{5}G_{3}^{{}^{\circ}}$
20	7445.44	13427.34	*z* ${\;}^{7}F_{5}^{{}^{\circ}}$—*ƒ* ^3^F_4_
10	7449.66	13419.74	*d* ^3^F_2_—*y* ${\;}^{3}G_{3}^{{}^{\circ}}$
30*h*	7450.62	13418.01	*z* ${\;}^{1}G_{4}^{{}^{\circ}}$—D_3_
40	7453.62	13412.61	*y* ${\;}^{3}D_{3}^{{}^{\circ}}$—*e* ^5^P_2_
2	7454.15	13411.65	*a* ^3^D_2_—*z* ${\;}^{5}D_{2}^{{}^{\circ}}$
9	7458.43	13403.96	*a* ^3^D_3_—*z* ${\;}^{7}F_{4}^{{}^{\circ}}$
30	7463.70	13394.49	*b* ^3^G_4_—*z* ${\;}^{1}G_{4}^{{}^{\circ}}$
5	7465.77	13390.78	*x* ${\;}^{5}D_{2}^{{}^{\circ}}$—*ƒ* ^7^D_2_
1	7467.78	13387.17	*c* ^3^F_3_—*y* ${\;}^{5}F_{3}^{{}^{\circ}}$
700	7468.91	13385.15	*z* ${\;}^{5}G_{2}^{{}^{\circ}}$—*e* ^5^F_1_
800	7485.75	13355.04	*z* ${\;}^{5}G_{3}^{{}^{\circ}}$—*e* ^5^F_2_
30	7486.07	13354.47	*a* ^3^H_5_—*z* ${\;}^{7}F_{4}^{{}^{\circ}}$
4	7495.18	13338.23	$\{\;\begin{array}{c} a{\;}^{1}P_{1}—z{\;}^{3}D_{1}^{{}^{\circ}}\\ y{\;}^{5}F_{4}^{{}^{\circ}}—f{\;}^{5}F_{3} \end{array}$
800	7499.74	13330.13	*z* ${\;}^{3}G_{5}^{{}^{\circ}}$—*e* ^3^F_4_
2	7516.05	13301.20	*a* ^1^I_6_—*z* ${\;}^{3}I_{5}^{{}^{\circ}}$
30	7519.59	13294.94	*b* ^3^G5—*z* ${\;}^{3}H_{6}^{{}^{\circ}}$
150	7532.07	13272.91	*d* ^3^P_2_—*x* ${\;}^{5}D_{1}^{{}^{\circ}}$
7	7540.85	13257.46	*a* ^3^G_5_—*z* ${\;}^{7}D_{4}^{{}^{\circ}}$
6	7544.19	13251.59	$\{\;\begin{array}{c} a{\;}^{3}H_{4}—z{\;}^{3}F_{4}^{{}^{\circ}}\\ d{\;}^{3}F_{3}—x{\;}^{3}D_{3}^{{}^{\circ}} \end{array}$
2	7549.87	13241.62	*z* ${\;}^{5}F_{4}^{{}^{\circ}}$—*e* ^5^F_5_
700	7559.62	13224.54	*z* ${\;}^{3}G_{4}^{{}^{\circ}}$—*e* ^3^F_3_
2	7563.68	13217.44	*z* ${\;}^{5}G_{2}^{{}^{\circ}}$—*e* ^5^F_3_
5	7569.34	13207.56	*y* ${\;}^{3}D_{2}^{{}^{\circ}}$—*e* ^5^P_2_
10	7575.08	13197.55	*b* ^3^G_3_—*z* ${\;}^{3}H_{4}^{{}^{\circ}}$
3	7598.23	13157.34	*c* ^3^P_2_—*y* ${\;}^{5}D_{1}^{{}^{\circ}}$
1	7600.39	13153.60	*a* ^3^H_4_—*z* ${\;}^{7}P_{4}^{{}^{\circ}}$
4	7609.01	13138.70	*c* ^3^P_2_—*z* ${\;}^{5}P_{3}^{{}^{\circ}}$
100	7612.97	13131.86	*z* ${\;}^{3}D_{3}^{{}^{\circ}}$—*e* ^5^F_3_
500	7621.52	13117.13	*z* ${\;}^{3}G_{3}^{{}^{\circ}}$—*e* ^3^F_2_
3*h*	7633.11	13097.21	*z* ${\;}^{7}F_{2}^{{}^{\circ}}$—*e* ^3^F_3_
5	7647.41	13072.72	*y* ${\;}^{5}F_{4}^{{}^{\circ}}$—*ƒ* ^5^F_4_
50	7658.43	13053.91	*z* ${\;}^{3}P_{2}^{{}^{\circ}}$—*e* ^5^P_2_
5	7660.35	13050.64	*y* ${\;}^{5}F_{4}^{{}^{\circ}}$—*e* ^5^G_5_
10	7665.14	13042.49	*a* ^1^F_3_—*y* ${\;}^{3}F_{4}^{{}^{\circ}}$
20*h*	7677.45	13021.58	*y* ${\;}^{3}F_{3}^{{}^{\circ}}$—D_3_
20*h*	7687.50	13004.55	*z* ${\;}^{3}G_{4}^{{}^{\circ}}$—*ƒ* ^3^F_4_
1	7688.62	13002.66	*y* ${\;}^{5}F_{3}^{{}^{\circ}}$—*ƒ* ^5^F_3_
3	7690.54	12999.41	*y* ${\;}^{3}D_{1}^{{}^{\circ}}$—*e* ^7^D_1_
2*h*	7691.61	12997.60	*y* ${\;}^{3}D_{3}^{{}^{\circ}}$—*e* ^3^D_2_
3	7694.52	12992.69	*y* ${\;}^{5}P_{2}^{{}^{\circ}}$—*f* ^5^D_2_
4*h*	7697.42	12987.79	*y* ${\;}^{3}P_{2}^{{}^{\circ}}$—*ƒ* ^7^D_1_
1	7697.67	12987.37	*d* ^3^F_4_—*y* ${\;}^{3}G_{4}^{{}^{\circ}}$
9	7701.42	12981.05	*a* ^3^D_1_—*z* ${\;}^{5}F_{1}^{{}^{\circ}}$
10	7719.26	12951.05	*x* ${\;}^{5}D_{2}^{{}^{\circ}}$—*e* ${\;}^{7}D_{1}^{{}^{\circ}}$
500	7722.89	12944.96	*a* ^3^H_6_—*z* ${\;}^{3}G_{5}^{{}^{\circ}}$
3	7728.99	12934.74	*z* ${\;}^{5}F_{3}^{{}^{\circ}}$—*e* ^3^F_4_
20	7729.87	12933.27	*z* ${\;}^{5}G_{2}^{{}^{\circ}}$—*e* ^5^F_2_
2	7733.99	12926.38	*d* ^3^F_2_—*y* ${\;}^{3}P_{2}^{{}^{\circ}}$
1	7736.19	12922.70	*d* ^3^F_3_— $1_{4}^{{}^{\circ}}$
2*h*	7737.17	12921.07	*y* ${\;}^{5}F_{3}^{{}^{\circ}}$—*e* ^3^G_4_
4	7739.79	12916.69	*d* ^3^F_3_—*y* ${\;}^{3}F_{2}^{{}^{\circ}}$
7	7770.86	12865.05	*z* ${\;}^{7}P_{4}^{{}^{\circ}}$—*e* ^3^F_3_
2	7774.98	12858.23	*x* ${\;}^{5}D_{1}^{{}^{\circ}}$—*ƒ* ^7^D_2_
6	7781.34	12847.72	*z* ${\;}^{3}D_{3}^{{}^{\circ}}$—*e* ^5^F_2_
3*h*	7788.90	12835.25	*y* ${\;}^{3}P_{0}^{{}^{\circ}}$—*ƒ* ^7^D_1_
2	7789.58	12834.13	*z* ${\;}^{3}P_{2}^{{}^{\circ}}$—F_1_
300	7791.81	12830.46	*z* ${\;}^{5}G_{4}^{{}^{\circ}}$—*e* ^5^F_3_
3	7800.16	12816.72	*x* ${\;}^{5}D_{0}^{{}^{\circ}}$—*e* ^7^D_1_
1*h*	7802.32	12813.17	*y* ${\;}^{3}P_{1}^{{}^{\circ}}$—*ƒ* ^7^D_1_
2	7806.52	12806.28	*a* ^1^G_4_—*z* ${\;}^{5}D_{3}^{{}^{\circ}}$
100	7806.78	12805.85	*x* ${\;}^{5}D_{3}^{{}^{\circ}}$—*e* ^5^P_2_
5	7809.19	12801.90	*b* ^3^H_4_—*y* ${\;}^{5}F_{3}^{{}^{\circ}}$
40	7813.43	12794.96	$\{\;\begin{array}{c} c{\;}^{3}P_{2}—y{\;}^{5}D_{2}^{{}^{\circ}}\\ a{\;}^{3}H_{4}—z{\;}^{7}F_{3}^{{}^{\circ}} \end{array}$
5	7813.96	12794.09	*a* ^3^H_4_—*z* ${\;}^{3}G_{4}^{{}^{\circ}}$
5*h*	7815.38	12791.76	*z* ${\;}^{7}P_{3}^{{}^{\circ}}$—*e* ^5^F_3_
70	7829.81	12768.19	*z* ${\;}^{7}P_{4}^{{}^{\circ}}$—*e* ^5^F_4_
10	7830.51	12767.05	*z* ${\;}^{3}F_{4}^{{}^{\circ}}$—*e* ^3^F_3_
100	7833.37	12762.39	*z* ${\;}^{3}D_{2}^{{}^{\circ}}$—*e* ^3^F_2_
1	7834.02	12761.33	*z* ${\;}^{3}G_{5}^{{}^{\circ}}$—*e* ^5^F_5_
40	7834.78	12760.09	*d* ^3^F_3_—*y* ${\;}^{3}G_{4}^{{}^{\circ}}$
3	7838.42	12754.17	*c* ^3^P_1_—*z* ${\;}^{5}P_{1}^{{}^{\circ}}$
100	7841.92	12748.47	*x* ${\;}^{5}D_{4}^{{}^{\circ}}$—*e* ^5^P_3_
300	7847.81	12738.90	*z* ${\;}^{5}G_{5}^{{}^{\circ}}$—*e* ^5^F_4_
20	7853.76	12729.25	*y* ${\;}^{3}D_{3}^{{}^{\circ}}$—*e* ^5^S_2_
1	7862.70	12714.78	*a* ^3^D_1_—*z* ${\;}^{5}F_{2}^{{}^{\circ}}$
10	7871.92	12699.89	*a* ^3^D_3_—*z* ${\;}^{5}F_{3}^{{}^{\circ}}$
3	7875.27	12694.49	*b* ^3^G_5_—*z* ${\;}^{3}H_{5}^{{}^{\circ}}$
10	7876.78	12692.05	*d* ^3^P_2_—*y* ${\;}^{3}D_{1}^{{}^{\circ}}$
200	7881.47	12684.50	*z* ${\;}^{5}G_{6}^{{}^{\circ}}$—*e* ^5^F_5_
400	7890.40	12670.14	*z* ${\;}^{3}F_{4}^{{}^{\circ}}$—*e* ^5^F_4_
30	7900.20	12654.43	*b* ^3^G_4_—*z* ${\;}^{3}H_{5}^{{}^{\circ}}$
9	7905.17	12646.47	*c* ^3^P_2_—*z* ${\;}^{3}D_{1}^{{}^{\circ}}$
20	7906.10	12644.98	*z* ${\;}^{7}P_{4}^{{}^{\circ}}$—*ƒ* ^3^F_4_
10	7908.73	12640.78	*b* ^3^G^5^—*y* ${\;}^{3}F_{4}^{{}^{\circ}}$
1	7909.84	12639.00	*z* ${\;}^{3}P_{2}^{{}^{\circ}}$—*e* ^3^D_2_
7	7917.53	12626.73	*z* ${\;}^{7}P_{2}^{{}^{\circ}}$—*e* ^3^F_2_
60	7922.97	12618.06	*d* ^3^F_2_—*y* ${\;}^{3}F_{3}^{{}^{\circ}}$
400	7924.45	12615.70	*z* ${\;}^{5}G_{5}^{{}^{\circ}}$—*ƒ* ^3^F_4_
4	7930.36	12606.30	*z* ${\;}^{3}P_{1}^{{}^{\circ}}$—F_1_
3	7941.08	12589.28	*c* ^3^P_1_—*z* ${\;}^{5}P_{2}^{{}^{\circ}}$
10	7947.97	12578.37	*z* ${\;}^{5}G_{3}^{{}^{\circ}}$—*e* ^3^F_3_
200	7948.15	12578.08	*z* ${\;}^{3}F_{3}^{{}^{\circ}}$—*e* ^3^F_2_
6	7963.83	12553.32	*y* ${\;}^{3}D_{1}^{{}^{\circ}}$—*e* ^5^P_2_
200	7967.88	12546.94	*z* ${\;}^{3}F_{4}^{{}^{\circ}}$—*ƒ* ^3^F_4_
6	7994.63	12504.96	*x* ${\;}^{5}D_{2}^{{}^{\circ}}$—*e* ^5^P_2_
2	7999.28	12497.69	*a* ^3^H_4_—*z* ${\;}^{7}F_{4}^{{}^{\circ}}$
20	7999.79	12496.89	*c* ^3^P_2_—*y* ${\;}^{5}D_{3}^{{}^{\circ}}$
4	8009.64	12481.52	*z* ${\;}^{5}G_{3}^{{}^{\circ}}$—*e* ^5^F_4_
1	8028.28	12452.54	*a* ^3^D_2_—*z* ${\;}^{5}D_{3}^{{}^{\circ}}$
60	8036.68	12439.53	*d* ^3^P_2_—*x* ${\;}^{5}D_{3}^{{}^{\circ}}$
2	8043.36	12429.20	*c* ^3^F_4_—*z* ${\;}^{5}P_{3}^{{}^{\circ}}$
3	8049.79	12419.27	*d* ^3^P_2_—*z* ${\;}^{3}P_{1}^{{}^{\circ}}$
2	8050.29	12418.50	*x* ${\;}^{5}D_{1}^{{}^{\circ}}$—*e* ^7^D_1_
1*h*	8055.05	12411.16	*z* ${\;}^{3}P_{1}^{{}^{\circ}}$—*e* ^3^D_2_
3	8058.45	12405.92	*a* ^3^D_1_—*z* ${\;}^{5}D_{1}^{{}^{\circ}}$
10	8074.43	12381.37	*a* ^1^D_2_—*z* ${\;}^{5}F_{2}^{{}^{\circ}}$
2*h*	8083.23	12367.89	*b* ^3^D_3_—*x* ${\;}^{3}F_{4}^{{}^{\circ}}$
7	8089.47	12358.35	*z* ${\;}^{5}G_{3}^{{}^{\circ}}$—*ƒ* ^3^F_4_
20	8090.23	12357.19	*z* ${\;}^{7}F_{5}^{{}^{\circ}}$—*e* ^3^F_4_
2	8091.05	12355.94	*d* ^3^P_1_—*y* ${\;}^{3}P_{1}^{{}^{\circ}}$
4	8105.47	12333.96	*d* ^3^P_1_—*y* ${\;}^{3}P_{0}^{{}^{\circ}}$
150	8112.47	12323.31	*z* ${\;}^{3}G_{3}^{{}^{\circ}}$—*e* ^5^F_3_
1	8144.33	12275.11	*a* ^3^D_3_—*z* ${\;}^{5}D_{2}^{{}^{\circ}}$
15	8157.69	12255.00	*a* ^3^H_5_—*z* ${\;}^{3}G_{5}^{{}^{\circ}}$
20	8168.85	12238.26	*c* ^3^P_2_—*z* ${\;}^{3}F_{2}^{{}^{\circ}}$
15	8173.91	12230.69	*z* ${\;}^{7}F_{4}^{{}^{\circ}}$—*e* ^3^F_4_
30*h*	8181.99	12218.61	*y* ${\;}^{5}F_{5}^{{}^{\circ}}$—*e* ^5^G_6_
2	8185.82	12212.89	*d* ^3^P_1_—*y* ${\;}^{5}P_{1}^{{}^{\circ}}$
8*h*	8194.54	12199.89	*y* ${\;}^{5}F_{5}^{{}^{\circ}}$—*e* ^5^D_4_
5	8216.25	12167.66	*b* ^3^G_3_—*y* ${\;}^{3}F_{4}^{{}^{\circ}}$
50	8220.48	12161.40	*d* ^3^F_4_—*y* ${\;}^{3}G_{5}^{{}^{\circ}}$
2	8231.96	12144.44	*d* ^3^F_4_—*y* ${\;}^{3}F_{3}^{{}^{\circ}}$
4	8237.46	12136.33	*z* ${\;}^{3}D_{2}^{{}^{\circ}}$—*e* ^5^F_1_
15*h*	8239.21	12133.75	*y* ${\;}^{5}F_{5}^{{}^{\circ}}$—*ƒ* ^5^F_5_
4	8246.80	12122.58	*x* ${\;}^{5}D_{3}^{{}^{\circ}}$—*e* ^5^S_2_
3	8251.32	12115.94	*a* ^1^G_4_—*z* ${\;}^{5}F_{5}^{{}^{\circ}}$
400	8264.95	12095.96	*z* ${\;}^{7}F_{6}^{{}^{\circ}}$—*e* ^5^F_5_
3*h*	8269.02	12090.01	*x* ${\;}^{5}D_{2}^{{}^{\circ}}$—*e* ^3^D_2_
2	8281.00	12072.52	*a* ^1^D_2_—*z* ${\;}^{5}D_{1}^{{}^{\circ}}$
30	8281.98	12071.09	*z* ${\;}^{3}D_{3}^{{}^{\circ}}$—*e* ^3^F_3_
2	8296.45	12050.04	*y* ${\;}^{5}P_{2}^{{}^{\circ}}$—*f* ^7^D_2_
2	8297.23	12048.90	*y* ${\;}^{3}D_{3}^{{}^{\circ}}$—*ƒ* ^5^F_3_
2	8304.90	12037.78	*d* ^3^P_2_—*y* ${\;}^{3}D_{2}^{{}^{\circ}}$
2	8309.04	12031.78	*z* ${\;}^{3}S_{1}^{{}^{\circ}}$—*e* ^7^D_1_
2*h*	8320.13	12015.74	*y* ${\;}^{5}F_{4}^{{}^{\circ}}$—B_4_
1	8330.16	12001.27	*x* ${\;}^{5}D_{4}^{{}^{\circ}}$—*f* ^5^F_4_
250	8348.99	11974.21	*z* ${\;}^{3}D_{3}^{{}^{\circ}}$—*e* ^5^F_4_
7	8350.23	11972.43	*x* ${\;}^{5}D_{1}^{{}^{\circ}}$—*e* ^5^P_2_
200	8352.93	11968.56	*z* ${\;}^{3}D_{2}^{{}^{\circ}}$—*e* ^5^F_3_
1	8354.92	11965.71	*a* ^1^P_1_—*z* ${\;}^{3}D_{2}^{{}^{\circ}}$
4	8376.90	11934.31	*z* ${\;}^{3}G_{4}^{{}^{\circ}}$—*e* ^3^F_4_
1	8377.62	11933.29	*z* ${\;}^{7}F_{3}^{{}^{\circ}}$—*e* ^3^F_4_
3	8384.12	11924.03	*x* ${\;}^{5}D_{3}^{{}^{\circ}}$—*e* ^5^P_3_
10	8388.96	11917.15	*d* ^3^F_3_—*y* ${\;}^{3}F_{3}^{{}^{\circ}}$
1	8395.68	11907.62	*b* ^3^D_2_—*x* ${\;}^{3}F_{3}^{{}^{\circ}}$
15	8400.60	11900.64	*a* ^1^I_6_—*z* ${\;}^{3}I_{6}^{{}^{\circ}}$
80	8435.77	11851.03	*z* ${\;}^{3}D_{3}^{{}^{\circ}}$—*ƒ* ^3^F_4_
4	8440.42	11844.50	*a* ^1^D_2_—*z* ^5^F_3_
30	8448.69	11832.90	$\{\;\begin{array}{c} z{\;}^{7}P_{2}^{{}^{\circ}}—e{\;}^{5}F_{3}\\ d{\;}^{3}P_{2}—y{\;}^{3}D_{3}^{{}^{\circ}} \end{array}$
2	8454.92	11824.18	*a* ^3^D_3_—*z* ${\;}^{5}F_{4}^{{}^{\circ}}$
2	8456.70	11821.70	*x* ${\;}^{5}D_{2}^{{}^{\circ}}$—*e* ^5^S_2_
200	8473.66	11798.03	*z* ${\;}^{3}F_{2}^{{}^{\circ}}$—*e* ^3^F_2_
9	8480.60	11788.38	*z* ${\;}^{7}F_{5}^{{}^{\circ}}$—*e* ^5^F_5_
200	8483.56	11784.27	*z* ${\;}^{3}F_{3}^{{}^{\circ}}$—*e* ^5^F_3_
6	8490.45	11774.70	*a* ^3^H_5_—*z* ${\;}^{5}F_{4}^{{}^{\circ}}$
7	8494.06	11769.70	*z* ${\;}^{5}G_{4}^{{}^{\circ}}$—*e* ^3^F_3_
3	8497.94	11764.33	*a* ^1^F_3_—*y* ${\;}^{3}D_{2}^{{}^{\circ}}$
2	8503.42	11756.75	*d* ^3^F_3_—*z* ${\;}^{3}H_{4}^{{}^{\circ}}$
1	8506.01	11753.17	*a* ^3^D_1_—*z* ${\;}^{5}D_{2}^{{}^{\circ}}$
1*h*	8522.21	11730.82	*z* ${\;}^{7}P_{3}^{{}^{\circ}}$—*e* ^3^F_3_
5	8523.23	11729.42	*y* ${\;}^{3}D_{3}^{{}^{\circ}}$—*e* ^3^D_3_
9	8556.05	11684.43	*z* ${\;}^{3}D_{2}^{{}^{\circ}}$—*e* ^5^F_2_
1	8570.84	11664.26	*b* ^3^D_2_—*x* ${\;}^{3}D_{1}^{{}^{\circ}}$
4	8585.67	11644.12	*y* ${\;}^{5}P_{3}^{{}^{\circ}}$—*ƒ* ^5^D_4_
2	8593.20	11633.91	*z* ${\;}^{3}P_{3}^{{}^{\circ}}$—*e* ^5^F_4_
3	8609.02	11612.53	*a* ^1^G_4_—*z* ${\;}^{5}D_{4}^{{}^{\circ}}$
2	8616.83	11602.01	*z* ${\;}^{3}P_{2}^{{}^{\circ}}$—*e* ^5^D_2_
10	8637.05	11574.85	*z* ${\;}^{7}P_{4}^{{}^{\circ}}$—*e* ^3^F_4_
2	8649.99	11557.53	*x* ${\;}^{5}D_{1}^{{}^{\circ}}$—*e* ^3^D_2_
1	8656.55	11548.77	*z* ${\;}^{7}P_{2}^{{}^{\circ}}$—*e* ^5^F_2_
10	8658.95	11545.57	*z* ${\;}^{5}G_{5}^{{}^{\circ}}$—*e* ^3^F_4_
2	8693.15	11500.15	*z* ${\;}^{3}F_{3}^{{}^{\circ}}$—*e* ^5^F_2_
200	8710.84	11476.80	*z* ${\;}^{3}F_{4}^{{}^{\circ}}$—*e* ^3^F_4_
100	8724.97	11458.21	*c* ^3^P_2_—*z* ${\;}^{3}F_{3}^{{}^{\circ}}$
5	8741.88	11436.05	*c* ^3^P_1_—*y* ${\;}^{5}D_{2}^{{}^{\circ}}$
1	8754.37	11419.73	*a* ^1^D_2_—*z* ${\;}^{5}D_{2}^{{}^{\circ}}$
2	8757.45	11415.71	*b* ^3^D_2_—*x* ${\;}^{3}D_{3}^{{}^{\circ}}$
60	8777.36	11389.82	*z* ${\;}^{3}D_{1}^{{}^{\circ}}$—*e* ^3^F_2_
1	8839.92	11309.21	*c* ^3^F_3_—*y* ${\;}^{5}D_{2}^{{}^{\circ}}$
20	8856.89	11287.55	*c* ^3^P_1_—*z* ${\;}^{3}D_{1}^{{}^{\circ}}$
5	8867.64	11273.86	*c* ^3^P_2_—*z* ${\;}^{3}D_{2}^{{}^{\circ}}$
2	8873.76	11266.09	*a* ^3^H_6_—*z* ${\;}^{5}F_{5}^{{}^{\circ}}$
1	8876.55	11262.55	*z* ${\;}^{3}G_{3}^{{}^{\circ}}$—*e* ^3^F^3^
1	8893.29	11241.35	*y* ${\;}^{5}D_{2}^{{}^{\circ}}$—*e* ^3^F_2_
2	8896.66	11237.09	*c* ^3^F_2_—*z* ${\;}^{3}D_{1}^{{}^{\circ}}$
1	8944.57	11176.90	*x* ${\;}^{5}D_{3}^{{}^{\circ}}$—*ƒ* ^5^F_4_
3	8948.51	11171.98	*z* ${\;}^{3}F_{2}^{{}^{\circ}}$—*e* ^5^F_1_
5	8953.57	11165.66	*z* ${\;}^{3}G_{3}^{{}^{\circ}}$—*e* ^5^F_4_
2	9053.45	11042.48	*z* ${\;}^{3}G_{3}^{{}^{\circ}}$—*ƒ* ^3^F_4_
2	9066.50	11026.59	*d* ^3^P_1_—*z* ${\;}^{3}S_{1}^{{}^{\circ}}$
1	9079.09	11011.30	*c* ^3^F_3_—*y* ${\;}^{5}D_{3}^{{}^{\circ}}$
2*h*	9081.78	11008.04	*y* ${\;}^{3}P_{2}^{{}^{\circ}}$—*e* ^7^D_3_
50	9084.95	11004.20	*z* ${\;}^{3}F_{2}^{{}^{\circ}}$—*e* ^5^F_3_
8	9107.72	10976.68	*z* ${\;}^{5}G_{5}^{{}^{\circ}}$—*e* ^5^F_5_
10	9126.45	10954.16	*d* ^3^F_4_—*y* ${\;}^{3}F_{4}^{{}^{\circ}}$
4	9134.63	10944.35	*x* ${\;}^{5}D_{4}^{{}^{\circ}}$—B_4_
15	9155.72	10919.14	*c* ^3^P_2_—*z* ${\;}^{3}G_{3}^{{}^{\circ}}$
1	9156.72	10917.95	*a* ^3^H_4_—*z* ${\;}^{5}F_{4}^{{}^{\circ}}$
90	9165.22	10907.82	$\{\;\begin{array}{c} z{\;}^{3}D_{2}^{{}^{\circ}}—e{\;}^{3}F_{3}\\ z{\;}^{3}F_{4}^{{}^{\circ}}—e{\;}^{5}F_{5} \end{array}$
1	9169.71	10902.48	*z* ${\;}^{3}S_{1}^{{}^{\circ}}$—*e* ^5^S_2_
2	9186.16	10882.96	*b* ^3^D_2_—*y* ${\;}^{3}G_{3}^{{}^{\circ}}$
150	9189.20	10879.36	*c* ^3^P_1_—*z* ${\;}^{3}F_{2}^{{}^{\circ}}$
2	9216.46	10847.18	*y* ${\;}^{5}P_{3}^{{}^{\circ}}$—*e* ^5^G_3_
7	9232.01	10828.91	*c* ^3^F_2_—*z* ${\;}^{3}F_{2}^{{}^{\circ}}$
2	9253.42	10803.85	*d* ^3^F_2_—*y* ${\;}^{3}D_{1}^{{}^{\circ}}$
4*h*	9266.02	10789.16	*b* ^3^D_3_—*x* ${\;}^{3}D_{3}^{{}^{\circ}}$
3	9271.16	10783.18	*y* ${\;}^{5}P_{3}^{{}^{\circ}}$—*e* ^5^S_2_
200	9273.15	10780.86	*z* ${\;}^{3}D_{3}^{{}^{\circ}}$—*e* ^3^F_4_
5*h*	9280.75	10772.04	*z* ${\;}^{7}P_{2}^{{}^{\circ}}$—*e* ^3^F_3_
1*h*	9285.60	10766.41	*y* ${\;}^{5}P_{1}^{{}^{\circ}}$—*e* ^7^D_2_
1	9287.92	10763.72	*z* ${\;}^{3}D_{1}^{{}^{\circ}}$—*e* ^5^F_1_
1	9288.60	10762.93	*a* ^1^F_3_—*y* ${\;}^{5}F_{2}^{{}^{\circ}}$
10	9297.53	10752.59	*c* ^3^F_3_—*z* ${\;}^{3}F_{2}^{{}^{\circ}}$
2	9300.70	10748.93	*c* ^3^F_4_—*z* ${\;}^{3}F_{3}^{{}^{\circ}}$
1	9307.23	10741.39	*x* ${\;}^{3}F_{3}^{{}^{\circ}}$—*ƒ* ^5^D_2_
15	9319.87	10726.82	*d* ^3^F_3_—*y* ${\;}^{3}F_{4}^{{}^{\circ}}$
50	9322.77	10723.48	*z* ${\;}^{3}F_{3}^{{}^{\circ}}$—*e* ^3^F_3_
3	9325.77	10720.03	*z* ${\;}^{3}F_{2}^{{}^{\circ}}$—*e* ^5^F_2_
1	9341.10	10702.44	*y* ${\;}^{3}P_{1}^{{}^{\circ}}$—*e* ^7^D_1_
4*h*	9344.62	10698.41	*y* ${\;}^{3}D_{3}^{{}^{\circ}}$—*e* ^3^P_2_
15	9396.09	10639.81	*d* ^3^P_1_—*x* ${\;}^{5}D_{1}^{{}^{\circ}}$
20	9407.76	10626.61	*z* ${\;}^{3}F_{3}^{{}^{\circ}}$—*e* ^5^F_4_
20	9445.03	10584.68	*y* ${\;}^{5}P_{3}^{{}^{\circ}}$—*e* ^5^P_3_
1	9452.67	10576.12	*a* ^3^H_5_—*z* ${\;}^{5}F_{5}^{{}^{\circ}}$
4	9463.41	10564.12	*b* ^3^D_2_—*y* ${\;}^{3}P_{1}^{{}^{\circ}}$
6	9518.09	10503.43	*z* ${\;}^{3}F_{3}^{{}^{\circ}}$—*ƒ* ^3^F_4_
3	9535.45	10484.31	*y* ${\;}^{3}F_{4}^{{}^{\circ}}$—*e* ^3^G_4_
1	9538.48	10480.98	*y* ${\;}^{5}P_{2}^{{}^{\circ}}$—*e* ^5^S_2_
50	9539.87	10479.45	*z* ${\;}^{5}G_{4}^{{}^{\circ}}$—*e* ^3^F_4_
1	9557.05	10460.61	*a* ^1^D_2_—*z* ${\;}^{5}D_{3}^{{}^{\circ}}$
1	9565.95	10450.88	*c* ^3^P_2_—*z* ${\;}^{7}P_{3}^{{}^{\circ}}$
2	9569.01	10447.54	*y* ${\;}^{5}D_{2}^{{}^{\circ}}$—*e* ^5^F_3_
60	9585.26	10429.82	*x* ${\;}^{5}D_{4}^{{}^{\circ}}$—*e* ^5^D_4_
3	9587.00	10427.93	*y* ${\;}^{3}D_{3}^{{}^{\circ}}$—*e* ^5^D_3_
1	9588.83	10425.94	*b* ^3^H_4_—*y* ${\;}^{5}D_{3}^{{}^{\circ}}$
1	9593.28	10421.11	*b* ^3^D_2_—*y* ${\;}^{5}P_{1}^{{}^{\circ}}$
1	9603.68	10409.82	*a* ^3^H_4_—*z* ${\;}^{5}D_{3}^{{}^{\circ}}$
1*h*	9613.40	10399.29	*y* ${\;}^{5}P_{1}^{{}^{\circ}}$—*e* ^5^P_2_
3	9622.37	10389.60	*b* ^3^D_2_—*y* ${\;}^{3}P_{2}^{{}^{\circ}}$
15*h*	9646.43	10363.69	*x* ${\;}^{5}D_{4}^{{}^{\circ}}$—*ƒ* ^5^F_5_
30	9694.93	10311.84	*z* ${\;}^{3}D_{1}^{{}^{\circ}}$—*e* ^5^F_2_
3*h*	9722.59	10282.51	*y* ${\;}^{5}P_{2}^{{}^{\circ}}$—*e* ^5^P_3_
3*h*	9726.44	10278.44	*y* ${\;}^{3}F_{4}^{{}^{\circ}}$—*e* ^5^G_5_
2	9729.37	10275.34	*b* ^1^D_2_—*y* ${\;}^{5}D_{2}^{{}^{\circ}}$
4	9747.44	10256.29	$\{\;\begin{array}{c} b{\;}^{3}D_{3}—y{\;}^{3}G_{3}^{{}^{\circ}}\\ y{\;}^{3}P_{1}^{{}^{\circ}}—e{\;}^{5}P_{2} \end{array}$
2	9751.24	10252.30	*c* ^3^P_2_—*z* ${\;}^{5}S_{2}^{{}^{\circ}}$
1*h*	9778.20	10224.03	*a* ^1^H_5_—*z* ${\;}^{5}G_{5}^{{}^{\circ}}$
60	9791.77	10209.86	*c* ^3^F_4_—*z* ${\;}^{3}G_{3}^{{}^{\circ}}$
2*h*	9820.88	10179.60	*y* ${\;}^{5}P_{1}^{{}^{\circ}}$—F_1_
4*h*	9828.92	10171.27	*y* ${\;}^{3}F_{3}^{{}^{\circ}}$—*e* ^5^G_4_
2	9849.75	10149.76	*d* ^3^F_2_—*y* ${\;}^{3}D_{2}^{{}^{\circ}}$
150	9887.87	10110.63	*c* ^3^P_2_—*z* ${\;}^{3}D_{3}^{{}^{\circ}}$
2*h*	9906.39	10091.73	*x* ${\;}^{5}D_{3}^{{}^{\circ}}$—*e* ^3^P_2_
2*h*	9909.98	10088.07	*z* ${\;}^{5}P_{2}^{{}^{\circ}}$—*e* ^3^F_2_
3	9916.63	10081.31	*b* ^3^D_2_—*y* ${\;}^{3}F_{3}^{{}^{\circ}}$
1	9920.03	10077.85	*d* ^3^F_4_—*x* ${\;}^{5}D_{3}^{{}^{\circ}}$
5	9946.83	10050.70	*c* ^3^P_1_—*z* ${\;}^{7}P_{2}^{{}^{\circ}}$
15*h*	10024.99	9972.34	$\{\;\begin{array}{c} c{\;}^{3}F_{3}—z{\;}^{3}F_{3}^{{}^{\circ}}\\ z{\;}^{3}G_{3}^{{}^{\circ}}—e{\;}^{3}F_{4} \end{array}$
30	10082.93	9915.03	*c* ^3^P_1_—*z* ${\;}^{3}D_{2}^{{}^{\circ}}$
1*h*	10215.71	9786.16	*d* ^3^P_1_—*z* ${\;}^{3}P_{1}^{{}^{\circ}}$
8	10228.91	9773.53	*a* ^1^I_6_—*y* ${\;}^{3}G_{5}^{{}^{\circ}}$
3	10286.65	9718.67	*b* ^1^D_2_—*z* ${\;}^{3}F_{2}^{{}^{\circ}}$
2	10289.39	9716.09	*y* ${\;}^{5}P_{1}^{{}^{\circ}}$—*e* ^5^S_2_
8	10303.56	9702.72	*c* ^3^F_4_—*z* ${\;}^{5}G_{4}^{{}^{\circ}}$
40	10512.58	9509.81	*c* ^3^F_2_—*z* ${\;}^{3}G_{3}^{{}^{\circ}}$
4	10630.05	9404.72	$\{\;\begin{array}{c} d{\;}^{3}P_{1}—y{\;}^{3}D_{2}^{{}^{\circ}}\\ x{\;}^{3}D_{3}^{{}^{\circ}}—e{\;}^{5}P_{2} \end{array}$
6	10649.83	9387.25	*b* ^3^H_4_—*z* ${\;}^{3}F_{3}^{{}^{\circ}}$
3*h*	10815.28	9243.65	$\{\;\begin{array}{c} y{\;}^{3}F_{4}^{{}^{\circ}}—B_{4}\\ d{\;}^{3}F_{3}—y{\;}^{3}D_{3}^{{}^{\circ}} \end{array}$
8	11199.67	8926.39	*c* ^3^F_3_—*z* ${\;}^{5}G_{4}^{{}^{\circ}}$
3	11298.66	8848.18	*b* ^3^H_4_—*z* ${\;}^{3}G_{3}^{{}^{\circ}}$
6	11325.88	8826.92	*b* ^3^H_5_—*z* ${\;}^{5}G_{4}^{{}^{\circ}}$
3	11483.91	8705.45	*c* ^3^F_4_—*z* ${\;}^{3}F_{4}^{{}^{\circ}}$

**Table 4:** Strongest unclassified lines

Intensity	Wavelength	Wavenumber
		
	*A*	*cm*^−1^
150	2279.584	43854.11
80	2306.911	43334.68
90	2360.530	42350.42
200	2420.826	41295.67
100	2450.560	40794.65
150	2495.691	40056.98
125	2560.845	39037.91
100	2570.973	38884.13
100	2601.456	38428.53
80	2676.354	37353.16
250	2735.669	36543.32
125	2753.433	36307.57
100	2812.817	35541.08
75	2998.346	33342.00
75	3059.175	32679.05
80	3226.906	30980.49
100	3327.700	30042.14
125	3332.051	30002.92
150	3693.760	27064.98
100	4738.410	21098.23
80	4806.212	20800.59
200	4832.998	20685.31
125	4910.236	20359.94
125	4919.004	20323.64
100	4975.371	20093.40
300H	5213.440	19175.86
150	5275.087	18951.76
200H	5334.716	18739.93
100	5350.420	18684.93
600H	5361.792	18645.30
500H	5401.047	18509.78
150H	5439.218	18379.89
250H	5479.415	18245.05
500	5578.412	17921.27
100	5603.552	17840.87
90	6528.73	15312.68
100	7266.95	13757.14
300	7475.40	13373.53
100	7529.58	13277.30
80	7797.90	12820.44

## Footnotes

1 Figures in brackets indicate the literature references on page 216.
